# Immune response to SARS-CoV-2 after a booster of mRNA-1273: an open-label phase 2 trial

**DOI:** 10.1038/s41591-022-01739-w

**Published:** 2022-03-03

**Authors:** Laurence Chu, Keith Vrbicky, David Montefiori, Wenmei Huang, Biliana Nestorova, Ying Chang, Andrea Carfi, Darin K. Edwards, Judy Oestreicher, Holly Legault, Frank J. Dutko, Bethany Girard, Rolando Pajon, Jacqueline M. Miller, Rituparna Das, Brett Leav, Roderick McPhee

**Affiliations:** 1 Benchmark Research, Austin, TX USA; 2 Meridian Clinical Research, Norfolk, NE USA; 3 Department of Surgery and Duke Human Vaccine Institute, Durham, NC USA; 4 Moderna, Inc., Cambridge, MA USA

**Keywords:** RNA vaccines, RNA vaccines

## Abstract

Rising breakthrough infections of severe acute respiratory syndrome coronavirus 2 (SARS-CoV-2) in previously immunized individuals have raised concerns for the need for a booster vaccine dose to combat waning antibody levels and new variants. Here we report the results of the open-label, non-randomized part B of a phase 2 trial in which we evaluated the safety and immunogenicity of a booster injection of 50 µg of the coronavirus disease 2019 (COVID-19) vaccine mRNA-1273 in 344 adult participants immunized 6–8 months earlier with a primary series of two doses of 50 µg or 100 µg of mRNA-1273 (NCT04405076). Neutralizing antibody (nAb) titers against wild-type SARS-CoV-2 at 1 month after the booster were 1.7-fold (95% confidence interval (CI): 1.5, 1.9) higher than those at 28 days after the second injection of the primary series, which met the pre-specified non-inferiority criterion (primary immunogenicity objective) and might indicate a memory B cell response. The nAb titers against the Delta variant (B.1.617.2) (exploratory objective) at 1 month after the booster were 2.1-fold (95% CI: 1.8, 2.4) higher than those at 28 days after the second injection of the primary series. The seroresponse rate (95% CI (four-fold rise from baseline)) was 100% (98.7, 100.0) at 28 days after the booster compared to 98.3% (96.0, 99.4) after the primary series. The higher antibody titers at 28 days after the booster dose compared to 28 days after the second dose in the phase 3 COVE study were also observed in two assays for anti-spike IgG antibody measured by ELISA and by Meso Scale Discovery (MSD) Multiplex. The frequency of solicited local and systemic adverse reactions after the booster dose was similar to that after the second dose in the primary two-dose series of mRNA-1273 (50 µg or 100 µg); no new signals were observed in the unsolicited adverse events; and no serious adverse events were reported in the 1-month follow-up period. These results show that a booster injection of mRNA-1273 more than 6 months after completing the primary two-dose series is safe and elicited nAb titers that were statistically significantly higher than the peak titers detected after the primary vaccination series, suggesting that a booster dose of mRNA-1273 might result in increased vaccine effectiveness against infection and disease caused by SARS-CoV-2.

## Abstract (web summary)

A third dose of the COVID-19 vaccine mRNA-1273 is safe and boosts SARS-CoV-2 neutralizing antibody titers almost two-fold higher than the peak levels observed after completion of a two-dose series, highlighting the potential clinical benefit of a booster dose.

## Main

Several vaccines for SARS-CoV-2 have been authorized worldwide for emergency use, and the mRNA vaccines have been approved for the primary series by the US Food & Drug Administration (FDA) for immunization to prevent COVID-19 disease (mRNA-1273 for individuals ≥18 years of age and BNT162b2 for individuals ≥16 years of age)^1,2^. In several countries worldwide, BNT162b2 has been authorized for use for primary vaccination in individuals older than 5 years of age^3^, and mRNA vaccines have been authorized for primary vaccination in individuals older than 12 years of age. Both vaccines have also been authorized as a three-dose primary series for immunocompromised populations, and the use of boosters in adults (mRNA-1273 and BNT162b2), as well as in adolescents and some children (BNT162b2), has also recently been authorized.

SARS-CoV-2 vaccines have been administered worldwide to billions of people^1,4,5^ and have proven safe and very efficacious in preventing hospitalizations and deaths due to COVID-19 (refs. ^6–8^). mRNA-1273 is a lipid nanoparticle-encapsulated messenger RNA encoding the spike protein of the Wuhan-Hu-1 isolate with two proline mutations introduced to stabilize the spike protein into the pre-fusion conformation. Vaccination with two doses of mRNA-1273 has shown immune responses against SARS-CoV-2, efficacy against COVID-19 disease in adults and adolescents and an acceptable safety and tolerability profile in several clinical trials^6,9–12^. Based on a primary vaccine efficacy of 94.1% against COVID-19 after a median follow-up of 64 days^6^, as shown in the phase 3 COVE study, mRNA-1273 received emergency use authorization from the FDA in December 2020, for use in adults 18 years of age or older^2^. Recently, the final efficacy analysis of the blinded part of the COVE study showed a vaccine efficacy of 93.2% over 5.3 months of follow-up^13^.

There are concerns that the efficacy of the SARS-CoV-2 vaccines might decrease due to waning antibody levels and/or emerging viral variants. Variants of SARS-CoV-2 with amino acid changes in the spike protein and elsewhere in the viral genome are circulating around the world^14^. Variants such as Delta (B.1.617.2) caused substantial outbreaks of COVID-19 in 2021 and 2022; and the Omicron variant (B.1.1.529), a highly transmissible variant of concern, has increased in prevalence in 2022 (refs. ^15,16^). The Omicron variant causes many infections but fewer hospitalizations and less severe disease than previously circulating variants^17–20^. nAb titers against Omicron were low several months after the primary series with SARS-CoV-2 vaccines compared to the titers against the ancestral virus^21–29^. However, administration of a third dose of an mRNA vaccine has been shown to increase nAb titers against Omicron^21,29^. Furthermore, a third dose of an mRNA vaccine has been shown to decrease hospitalizations, death rates, symptomatic infections and emergency department and urgent care encounters during periods of Delta and Omicron infections^17,30–32^.

With respect to the Delta variant, a small study showed that immunity against variants waned in vaccinated individuals such that approximately one-half did not have detectable nAbs to the Delta variant 6 months after vaccination with mRNA-1273 (ref. ^33^). An interim analysis reported that a booster dose of 50 µg of mRNA-1273 increased nAb titers against the Beta, Gamma, Delta, Epsilon and Iota variants^33^. In a large observational study performed by the Mayo Clinic, the mRNA vaccines were less effective against SARS-CoV-2 infections at a time when the Delta variant was prevalent^34^. In an exploratory analysis of the ongoing COVE trial during the open-label phase, lower incidence rates of COVID-19 and fewer severe cases of COVID-19 cases were observed during July–August 2021, when the Delta variant was dominant, compared to participants vaccinated more recently^35^. A decline in mRNA vaccine effectiveness against SARS-CoV-2 infection was observed in nursing home residents during the time period of 21 June–1 August 2021, when the Delta variant was prevalent; in frontline healthcare workers; and in a real-world effectiveness study in Qatar^36–38^.

These findings with variants and waning immunity after two doses of mRNA vaccines suggest that a booster vaccine injection might be beneficial. We previously reported the results from the blinded portion (part A) of this phase 2 trial of mRNA-1273 at eight sites in the United States in which participants received two injections of placebo or 50 µg or 100 µg of mRNA-1273 (ref. ^33^). Preliminary results of part A showed robust immune responses through 1 month after the second injection of mRNA-1273 and an acceptable safety profile in healthy adults aged 18 years and older^33^. In part B, the open-label, interventional phase of this study, participants who received a two-dose (50 µg or 100 µg of mRNA-1273) primary series in part A were offered a single booster dose of mRNA-1273 (50 μg). Here we report the immunogenicity, safety and reactogenicity after the booster dose as well as antibody persistence before the booster dose (approximately 209 days after the primary series).

## Results

### Trial population

**Fig. 1:**
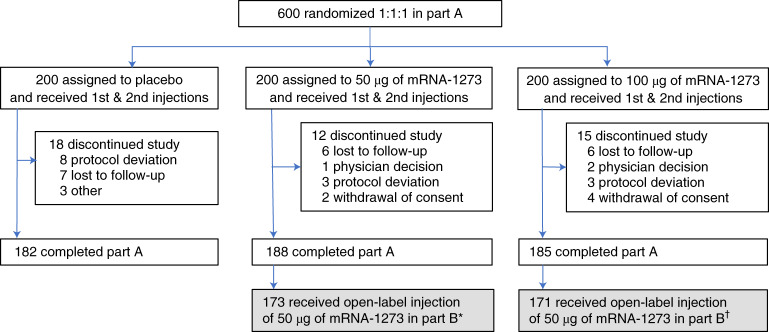
Trial profile for phase 2, parts A and B. Participants who received two doses of mRNA-1273 in part A were offered a booster injection of 50 µg of mRNA-1273 in part B. Completion of part A was defined as participants who completed 6 months of follow-up after the last injection received in part B (open-label phase). Data cutoff was 11 June 2021. *15 participants declined to receive a booster of 50 μg of mRNA-1273; ^†^14 participants declined to receive a booster of 50 μg of mRNA-1273.

Part A of this phase 2 trial (NCT04405076) comprised 600 participants who enrolled and received placebo or 50 µg or 100 µg of mRNA-1273 from 29 May 2020 to 8 July 2020. Of the 344 participants who enrolled and received a booster dose in part B from 28 January 2021 to 27 April 2021, 173 received two doses of 50 µg of mRNA-1273 and 171 received two doses of 100 µg of mRNA-1273 6–8 months earlier in part A (Fig. 1 and Supplementary Fig. 1). Immunogenicity and safety in this trial were compared to the primary series recipients in the immunogenicity cohort of the phase 3 COVE trial as a basis of immunobridging for the booster dose.

The baseline demographic characteristics of the participants who received a booster injection were generally similar between the groups that received a primary series of 50 µg or 100 µg of mRNA-1273 (Table 1). The phase 3 COVE trial had a greater representation of racial and ethnic groups as this was a major objective of phase 3 recruitment. In the current phase 2 trial, most participants were White and not Hispanic or Latino. The mean age of the participants in the groups that received the booster was 52.0 years in phase 2 part B and 54.5 years for those who received two doses in the phase 3 COVE trial. The time intervals (mean (s.d.; range)) between the second dose of mRNA-1273 during the primary series and the booster injection were 7.2 (0.6; 6.1–9.0) months for the group that received 50 µg of mRNA-1273 and 7.2 (0.6; 5.9–8.6) months for the group that received 100 µg of mRNA-1273 during the primary series.

**Table 1:** Demographics and characteristics (safety set)

Characteristic (%)	Phase 2 part B, 50-µg booster after 50-µg prime, *n* = 173	Phase 2 part B, 50-µg booster after 100-µg prime, *n* = 171	Phase 2 part B, 50-µg booster after 50- or 100-µg prime, *n* = 344	Phase 3 COVE after 100-µg prime *n* = 1,055
Age, y, mean (range)	52.0 (18–87)	52.0 (18–87)	52.0 (18–87)	54.5 (18–87)
Sex				
Male	49 (28.3)	67 (39.2)	116 (33.7)	560 (53.1)
Female	124 (71.7)	104 (60.8)	228 (66.3)	495 (46.9)
Race				
White	164 (94.8)	164 (95.9)	328 (95.3)	767 (72.7)
Black or African American	3 (1.7)	5 (2.9)	8 (2.3)	188 (17.8)
Asian	2 (1.2)	1 (0.6)	3 (0.9)	26 (2.5)
American Indian or Alaska Native	1 (0.6)	1 (0.6)	2 (0.6)	17 (1.6)
Native Hawaiian or other Pacific Islander, Multiracial, Other, Not reported, Unknown	3 (1.7)	0	3 (0.9)	57 (5.4)
Ethnicity				
Hispanic or Latino	10 (5.8)	10 (5.8)	20 (5.8)	334 (31.7)
Not Hispanic or Latino	162 (93.6)	161 (94.2)	323 (93.9)	717 (68.0)
Not reported or Unknown	1 (0.6)	0	1 (0.3)	4 (0.4)
Time interval between the second dose of mRNA-1273 during the primary series and the booster dose				
Mean (s.d.) (months^a^)	7.2 (0.6)	7.2 (0.6)	7.2 (0.6)	–
Range (months)	6.1–9.0	5.9–8.6	5.9–9.0	–
Body mass index (kg m^−^^2^)				
Mean (s.d.)	25.7 (3.3)	25.5 (3.2)	25.6 (3.2)	31.0 (7.8)

^a^Calculated with 30 days per month

Age was defined at the screening in part A. Percentages are based on the number of participants in the safety set.

### Safety

**Fig. 2:**
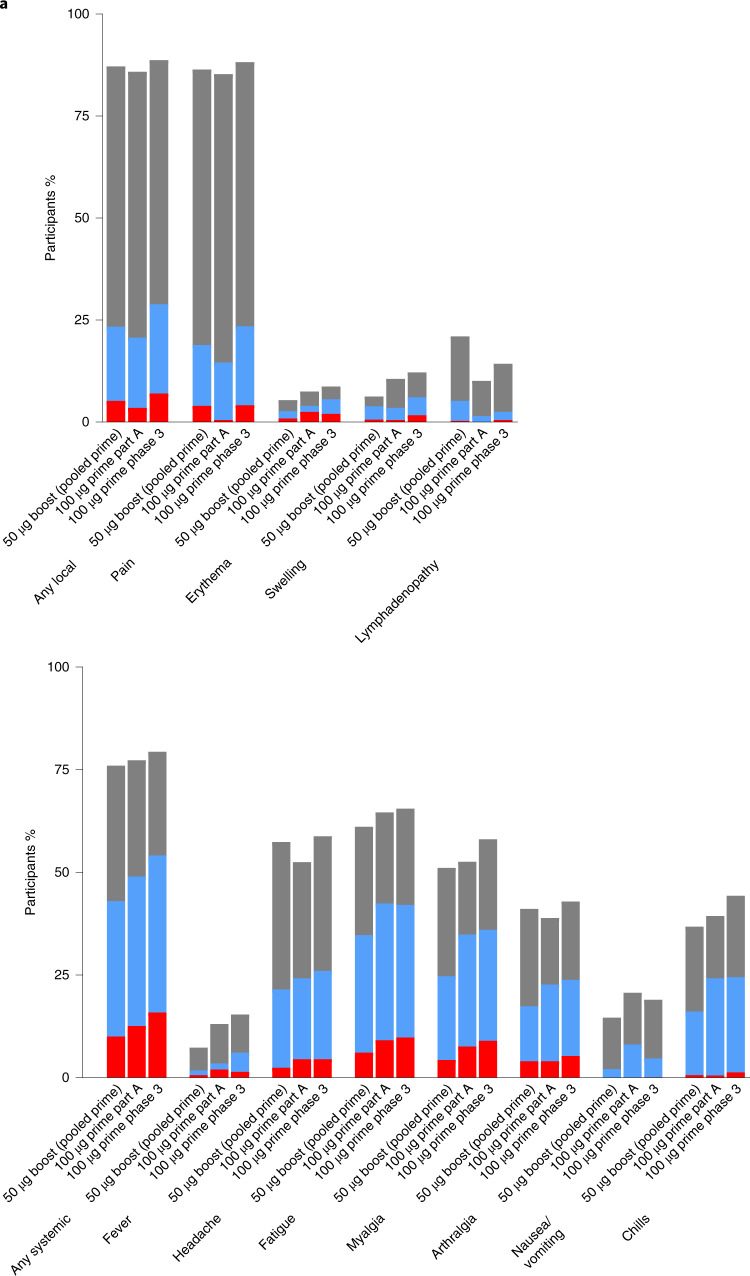
Solicited adverse reactions within 7 days after booster injection. **a**, Solicited local adverse reactions. **b**, Solicited systemic adverse reactions. The percentage of participants in the solicited safety set who reported local (**a**) or systemic (**b**) solicited adverse reactions is shown for 330 participants who received a booster dose of mRNA-1273 (50 μg) after a primary series of two doses of 50 µg or 100 µg of mRNA-1273 in part B; 198 participants who received a booster dose of mRNA-1273 (50 μg) after a primary series of two doses of 100 µg of mRNA-1273 in part B; and 14,691 participants who received two doses of 100 µg of mRNA-1273 in the phase 3 COVE trial. The percentages of participants who submitted any data for the adverse event within 7 days after the booster injection or the second dose during the primary series are shown. Grade 1 adverse reactions are indicated by gray bars, grade 2 adverse reactions by blue bars and grade 3 adverse reactions by red bars.

The percentages of participants with any solicited local or systemic adverse reactions within 7 days of the last injection were generally similar between the group that received a booster injection (part B) and the group in the phase 3 COVE trial that received two doses of mRNA-1273 (Fig. 2a,b and Supplementary Tables 1 and 2). The percentages of participants with any solicited local or systemic adverse reactions within 7 days of the last injection were also generally similar between the group that received a booster injection and the group that received two doses of mRNA-1273 during the blinded phase of this trial (part A) (Fig. 2a,b and Supplementary Tables 1 and 2). Most solicited local or systemic adverse reactions were mild (grade 1) or moderate (grade 2) (Fig. 2a,b). The incidence of any grade 3 solicited local or systemic adverse reaction after the booster injection were low (4.8%–12.9%) (Supplementary Tables 1 and 2). No grade 4 solicited local or systemic adverse events were reported after the booster injection.

The most common local adverse reaction was injection site pain, which was reported in 86.3% of those in the pooled group who received the 50 µg and 100 µg prime and in 88.3% of those in the phase 3 COVE trial (Supplementary Table 1). The most common grade 3 local adverse reaction was injection site pain in 3.6% of the participants who received a booster after the primary series of 100 µg of mRNA-1273 and in 4.1% of those in the phase 3 COVE trial (Supplementary Table 1). Incidences of local solicited adverse reactions in participants who received a booster injection were generally numerically similar to those in participants in the phase 3 COVE trial who received two doses of mRNA-1273 (Supplementary Table 1 and Fig. 2a). The incidences of local solicited adverse reactions in participants who received a booster injection were also similar to those in participants who received two doses of mRNA-1273 in this phase 2 trial (part A) (Supplementary Table 1 and Fig. 2a). However, lymphadenopathy was reported in 20.4% of participants who received a booster after the primary series of 100 µg of mRNA-1273 compared to 10.1% of participants in part A who received two doses of 100 µg of mRNA-1273 and 14.2% of participants in the phase 3 COVE trial after receiving the second dose of mRNA-1273 (Supplementary Table 1).

The most common systemic adverse reactions after the booster dose of mRNA-1273 were fatigue, headache and myalgia (Fig. 2b). Most solicited systemic adverse reactions after the booster injection were mild (grade 1) or moderate (grade 2) (Supplementary Table 2 and Fig. 2b). The most common grade 3 systemic adverse reaction after the booster dose was fatigue in 4.2% of the participants who received the primary series of 100 µg of mRNA-1273 (Fig. 2b and Supplementary Table 2). The incidences of systemic solicited adverse reactions were numerically similar in the group that received a booster injection and the group in the phase 3 COVE trial that received two doses of mRNA-1273 (Fig. 2b and Supplementary Table 2). The incidences of systemic solicited adverse reactions were also numerically similar in the group that received a booster injection and the group that received two doses of mRNA-1273 in this phase 2 trial (part A) (Fig. 2b and Supplementary Table 2).

The percentages of participants with any solicited local or systemic adverse reactions after receiving a booster injection were similar regardless of age group (≥18 to <55 years of age and ≥55 years of age; Supplementary Table 3). There were 39 (11.3%) unsolicited treatment-emergent adverse events (TEAEs) regardless of relationship to study vaccination as determined by the investigator up to 28 days after the booster injection. Thirteen (3.8%) unsolicited TEAEs were considered by the investigator to be related to study vaccination (Supplementary Table 4). None of these unsolicited TEAEs led to study discontinuation (Supplementary Table 4). There were 20 (5.8%) unsolicited treatment-emergent medically attended adverse events (MAAEs) regardless of relationship to study vaccination, as determined by the investigator. Two (0.6%) unsolicited treatment-emergent MAAEs were considered by the investigator to be related to study vaccination (Supplementary Table 4). No serious adverse events (SAEs) were reported up to 28 days after the booster injection.

### Immunogenicity

**Fig. 3:**
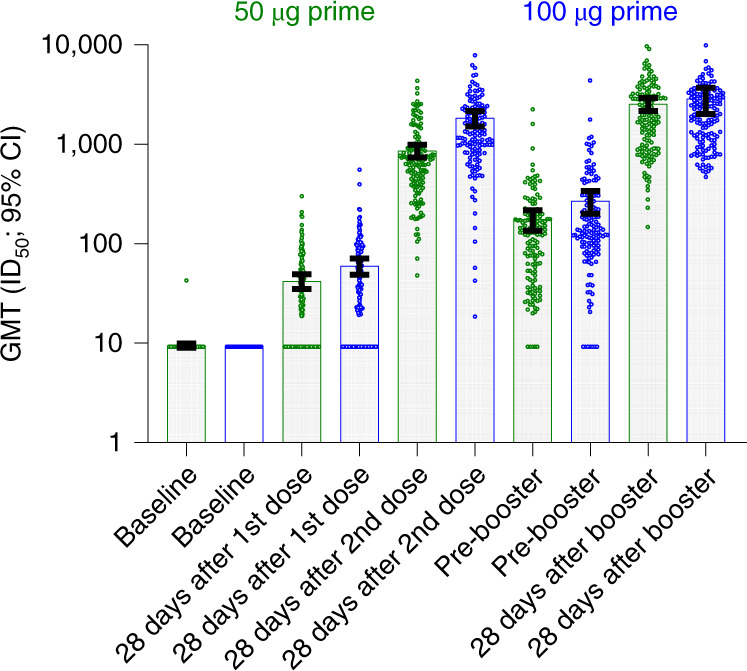
nAb titers (pseudovirus ID_50_; D614G) after the primary series and after a booster injection of 50 µg of mRNA-1273 (per-protocol set). The nAb titers in the pseudovirus assay against the D614G virus are shown for serum samples collected in part A at baseline, 28 days after the first dose of mRNA-1273, 28 days after the second dose of mRNA-1273 and in part B before the booster injection of 50 µg of mRNA-1273 (pre-booster) and 28 days after the booster injection. Titers from the group that received two priming doses of 50 µg of mRNA-1273 are shown in green, and those from the group that received two priming doses of 100 µg of mRNA-1273 are shown in blue. The dots show the results from individual serum samples. The tops of the bars show the GMTs. The whiskers were determined using the Tukey method. The tops of the whiskers show the 75th percentile minus the IQR (the difference between the 25th and 75th percentiles). The bottoms of the whiskers show the 25th percentile minus the IQR. Antibody values in the pseudovirus assay reported as below the LLOQ (18.5) were replaced by 0.5× LLOQ. Values that were greater than the ULOQ (45,118) were changed to the ULOQ if actual values were not available.

The geometric mean titers (GMTs; 95% CI) in the D614G pseudovirus nAb assay at day 57 (28 days after the second injection of mRNA-1273 during the primary series) were 629.2 (549.3, 720.8) in the group that received the primary series of 50 µg of mRNA-1273 and 1,268.0 (1,087.9, 1,477.8) in the group that received the primary series of 100 µg of mRNA-1273 (Fig. 3, Supplementary Fig. 2 and Supplementary Table 5). Before the administration of the booster (open-label day 1 (OL-D1); pre-booster), the titers of nAbs were 104.7 (88.3, 124.1) and 150.2 (125.7, 179.5) in the 50-µg and 100-µg groups, respectively (Fig. 3, Supplementary Fig. 5 and Supplementary Table 5). At OL-D29 (28 days after the 50-µg booster), the nAb titers were 1,834.3 (1,600.2, 2,102.6) in the 50-µg group and 1,951.7 (1,729.6, 2,202.4) in the 100-µg group (Fig. 3, Supplementary Fig. 5 and Supplementary Table 5). The GMTs (95% CIs) of nAb at 28 days after the booster (1,834.3 (1,600.2, 2102.6)) in the 50-µg group were higher than the titers of antibody at 28 days after the second dose of 50 µg of mRNA-1273 during the primary series (629.2 (549.3, 720.8)) (Fig. 3 and Supplementary Table 5). The GMTs (95% CIs) of nAb at 28 days after the booster (1,951.7 (1,729.6, 2,202.4) in the 100-µg group) were also higher than the titers of antibody at 28 days after the second dose of 100 µg of mRNA-1273 during the primary series (1,268.0 (1,087.9, 1,477.8)) (Fig. 3 and Supplementary Table 5). In addition, the GMTs after the second dose of mRNA-1273 were higher at 1 month after the second dose in the 100-µg group (1,268.0; 95% CI: 1,087.9, 1,477.8) compared to the 50-µg group (629.2; 95% CI: 549.3, 720.8) (Fig. 3 and Supplementary Table 5). The geometric mean ratios (GMRs) at 28 days after the booster compared to 28 days after the second dose in the primary series were 2.9 (2.6, 3.4) for the 50-µg group and 1.5 (1.3, 1.8) for the 100-µg group (Supplementary Table 5). The difference in the GMRs between the 50-µg and 100-µg groups is most likely due to differences in the GMTs in these two groups at 1 month after the second injection of mRNA-1273 (Fig. 3 and Supplementary Table 5). The interquartile range (IQR) (Q1–Q3) and the individual values for nAb titers at various time points for the 50-µg and 100-µg prime groups are shown in Supplementary Figs. 2 and 5.

The nAb 50% inhibitory dilution (ID_50_) titers at 28 days after the booster dose of mRNA-1273 in participants in part B of this study were also higher than those at 28 days after the second dose of mRNA-1273 in participants in the pivotal phase 3 COVE trial (Table 2). The GMR was 1.7 (95% CI: 1.5, 1.9) in a comparison of the pseudovirus nAb titers against D614G at 28 days after the booster dose to those in the phase 3 COVE trial at 28 days after the second dose (in which vaccine efficacy was demonstrated^5^) (Table 2). This GMR of 1.7 was above the pre-specified threshold of 1.0, and the lower bound of the 95% CI was greater than 0.67 (corresponding to a non-inferiority margin of 1.5). Therefore, the pre-specified criterion for non-inferiority was met for the GMT ratio. In a comparison of the pseudovirus nAb ID_50_ titers against the Delta variant at 28 days after the booster dose to those in the phase 3 COVE trial at 28 days after the second dose, the GMR was 2.1 (95% CI: 1.8, 2.4) (Table 2). The higher antibody titers at 28 days after the booster dose compared to 28 days after the second dose in the phase 3 COVE study were observed in three assays for D614G: pseudovirus nAbs, anti-spike IgG antibody by ELISA (VAC65) and anti-spike IgG antibody by MSD Multiplex (Supplementary Tables 6 and 7 and Supplementary Fig. 3). The GMTs (95% CI) for anti-spike IgG antibody against D614G by ELISA at 28 days after the booster were 651.8 (592.4, 717.1) compared to 1,080.4 (1,015.5, 1,149.4) at 28 days after the second 100-µg dose during the prime series (Supplementary Table 7; GM fold rise (95% CI): 1.7 (1.5, 1.9)). The age of the participants did not have a substantial effect on the immune response at 28 days after the booster dose (Supplementary Table 9).

**Table 2:** Pseudovirus nAb titers (ID_50_; against D614G or Delta) of mRNA-1273 after the booster compared to the phase 3 COVE primary series titers (per-protocol immunogenicity set)

Pseudovirus neutralization assay	D614G		Delta (B.1.617.2)	
	Phase 2 part B, 50-µg mRNA-1273 booster	Phase 3 COVE, 100-µg mRNA-1273, primary series	Phase 2 part B, 50-µg mRNA-1273 booster	Phase 3 COVE, 100-µg mRNA-1273, primary series
28 days after the booster (OL-D29, phase 2 part B) or 28 days after the second dose in phase 3 COVE				
*n*	295	1,053	295	580
GLSM	1,767.9	1,032.7	743.9	354.0
95% CI	1,586.4, 1,970.2	974.2, 1,094.7	663.7, 833.7	325.0, 385.5
GMR (phase 2 part B versus phase 3 COVE, model based)	1.7		2.1	
95% CI	1.5, 1.9		1.8, 2.4	

*n* indicates the number of individuals with no missing data at the corresponding time point. Antibody values reported as below the LLOQ were replaced by 0.5× LLOQ. Values greater than the ULOQ were replaced by the ULOQ if actual values are not available.

The log-transformed antibody titers were analyzed using an ANCOVA model with the group variable (phase 2 part B and phase 3 COVE) as fixed effect. The resulted least squares means, difference of least squares means and 95% CIs were back-transformed to the original scale for presentation.

Seroresponse rates (assay-specific definition) (95% CI) were 93.5% (90.1, 96.1) and 98.9% (98.0, 99.4) in the pooled phase 2 part B booster group (compared to pre-boost) and after the primary series in the phase 3 COVE trial, respectively (Table 3). The seroresponse rates (four-fold rise from baseline) (95% CI) were 90.1% (86.1, 93.3) and 98.4% (97.4, 99.1) in the booster group and after the primary series in the phase 3 COVE trial, respectively (Table 3). The seroresponse rates (four-fold rise from baseline) (95% CI) were 100% (98.7, 100.0) and 98.3% (96.0, 99.4) in the pooled phase 2 part B booster group and in the pooled phase 2 part A priming group (50 µg or 100 µg), respectively (Table 3). The seroresponse rates in the pseudovirus nAb assay were non-inferior for the phase 2 part B group after the booster injection compared to the phase 3 COVE trial after the primary series (Table 3), based on the assay-specific seroresponse definition. Given that the lower limit of the 95% CI for the group difference in seroresponse rates (four-fold rise from baseline) was greater than 0, the observed seroresponse rates were statistically significantly higher after the booster dose than after the second dose in part A of the phase 2 study, and the seroresponse rate difference met the pre-specified criterion for non-inferiority.

**Table 3:** Seroresponse rates by pseudovirus nAb (ID_50_; D614G) assay: phase 2 after booster compared to the phase 3 COVE primary series (per-protocol immunogenicity set)

Statistic	Seroresponse rate per assay-specific definition^a^		Seroresponse rate per four-fold definition^b^		Seroresponse rate per four-fold rise from baseline definition^c^	
	Phase 2 part B, 50-µg booster^d^ (*n* = 295)	Phase 3 COVE, 100 µg, primary series (*n* = 1,055)	Phase 2 part B, 50-µg booster^d^ (*n* = 295)	Phase 3 COVE, 100-µg, primary series (*n* = 1,055)	Phase 2 part B, 50-µg booster^e^ (*n* = 294)	Phase 2 part A, after dose 2 of primary series (*n* = 294)
N1	294	1,050	294	1,050	289	289
Participants achieving seroresponse, *n* (seroresponse rate %)	275 (93.5)	1,038 (98.9)	265 (90.1)	1,033 (98.4)	289 (100.0)	284 (98.3)
95% CI^f^	90.1, 96.1	98.0, 99.4	86.1, 93.3	97.4, 99.1	98.7, 100.0	96.0, 99.4
Difference in seroresponse rate^g^ (phase 2 part B versus phase 3 COVE) (%)	−5.3		−8.2		1.7	
95% CI^h^	−8.8, −2.9		−12.2, −5.2		0.4, 4.0	

N1, number of participants with non-missing data at both post-baseline time point of interest and baseline.

For participants who received the primary series in phase 3 COVE, seroresponse was defined based on the fold rise at day 57 (28 days after the second dose of mRNA-1273) compared to the baseline titer (before the first dose of the primary dose).

^a^Seroresponse specific to the ID_50_ titer in the D614G pseudovirus nAb assay at a participant level was defined as a change from below the LLOQ to equal to or above the LLOQ or at least a 3.3-fold rise if the baseline was equal to or above the LLOQ.

^b^Seroresponse at participant level was defined as a change of titer in the D614G pseudovirus nAb assay from below the LLOQ to equal to or above 4× LLOQ or a four times or higher ratio in participants with titers above the LLOQ.

^c^Seroresponse at participant level was defined as a change of titer in the D614G pseudovirus nAb assay from below the LLOQ at baseline (before Dose 1) to equal to or above 4× LLOQ or a four times or higher ratio in participants with titers above the LLOQ at baseline (before Dose 1).

^d^For participants who received a booster vaccination in phase 2 part B, seroresponse was defined based on the fold rise at OL-D29 (28 days after the booster dose of mRNA-1273) compared to the pre-booster titer (OL-D1; at least 6 months after completion of the primary series).

^e^For participants who received a booster vaccination in phase 2 part B, seroresponse was defined based on the fold rise at OL-D29 (28 days after the booster dose of mRNA-1273) compared to the baseline titer (before the first dose of the primary dose).

^f^95% CI was calculated using the Clopper–Pearson method.

^g^For the four-fold rise from baseline definition, the difference in seroresponse rate was the fold rise in phase 2 part B at OL-D29 (28 days after the booster dose of mRNA-1273) compared to 28 days after the second dose during the primary series in phase 2 part A.

^h^95% CI was calculated using the Miettinen–Nurminen (score) confidence limits.

In the group previously primed with two doses of 100-µg mRNA-1273, the nAb GMT versus the Delta variant was 47.9 (95% CI: 39.7, 57.8) before the booster and 827.8 (95% CI: 738.5, 927.9) at 28 days after the booster (Supplementary Table 8). Over 90% of booster recipients in the pooled group (92.2%; 95% CI: 88.5–95.0%; *n* = 293) met the definition of a seroresponse to the Delta variant using a four-fold increase from pre-booster baseline. Administration of the mRNA-1273 booster (50 µg) induced a 17.3-fold rise in neutralizing titers against the Delta variant compared to pre-booster titers in the 100-µg prime group (geometric mean fold rise (GMFR) = 17.3; 95% CI: 14.4, 20.8; Supplementary Table 8). Titers in the 100-µg prime group 28 days after the booster dose were 2.4-fold lower against the Delta variant (827.8; Supplementary Table 8) than against the D614G virus (1951.7; Supplementary Table 5). This difference in GMTs against the Delta variant and D614G virus is similar to the 2.9-fold difference (GMTs = 354.0 against Delta and 1,032.7 against D614G; Table 2) seen 28 days after the primary series of two doses of 100-µg mRNA-1273 in the phase 3 COVE study.

## Discussion

In this study, the administration of a booster dose of 50-µg mRNA-1273 to participants approximately 6–8 months after a primary series of two doses of 50 µg or 100 µg of mRNA-1273 resulted in a safety profile that was similar to that observed in the phase 3 COVE trial after receipt of the second dose of mRNA-1273. After receiving the booster injection of mRNA-1273, the most common local adverse reaction was pain, and the most common systemic adverse reactions were fatigue, headache and myalgia. The incidence and severity of local and systemic adverse reactions after the booster were similar to that observed after two doses of mRNA-1273 in part A of this study and in the phase 3 COVE trial^6,39^. Both co-primary immunogenicity objectives were met for the pooled group that received a booster (part B) compared to the group in the phase 3 COVE trial that received two doses of mRNA-1273. nAb titers (pseudovirus) against the D614G strain of virus at 28 days after the booster injection were higher than the titers at 28 days after the second dose of mRNA-1273 during the primary series in the COVE study. This higher level of antibody after the booster injection compared to that obtained after the second injection is suggestive of a robust memory response, likely due to stimulation of memory B cells^40–43^. It is also important to note that the results in this study using a validated pseudovirus neutralization assay showed that the GMTs after the second dose of mRNA-1273 were higher at 1 month after the second dose in the 100-µg group compared to the 50-µg group (Fig. 3 and Supplementary Table 5). Previous results using a qualified live virus nAb assay showed similar GMTs at 1 month after the second dose of 100 µg compared to 50 µg (~1,640)^12^. These different results between the 100-µg and 50-µg groups in this study are most likely due to differences in the dynamic ranges of the nAb assays.

SARS-CoV-2 mRNA vaccines appear to have retained their effectiveness over time in preventing hospitalization and severe disease due to Omicron, but the efficacy to prevent asymptomatic infection or mild symptomatic disease might have decreased since the emergence of the Delta and Omicron variants^17,30,33,34,36,44–46^ The GMFR (day 29 post-booster titer compared to pre-booster titer) achieved by the mRNA-1273 booster, measured by the Delta variant pseudovirus assay (19.0; 95% CI: 16.7, 21.5; Supplementary Table 8), points to the ability of the mRNA-1273 vaccine booster to enhance the breadth of nAb responses to both the original strain of SARS CoV-2 and other variants. Just as booster mRNA-1273 stimulated nAb titers against the original strain (GMTs of 1,892.7 at 28 days after the booster compared to 125.7 before the booster; Supplementary Table 5), a booster injection of mRNA-1273 was able to broaden and increase nAb titers against the Delta variant, highlighting the potential critical benefits of a mRNA-1273 booster dose. It is not clear whether a booster specific for one of more variants of concern will be necessary to achieve vaccine effectiveness against Omicron or future variants.

There are several limitations to the results of this study. This study was designed to assess the safety and immune response, but not the vaccine effectiveness, of a third dose of mRNA-1273 administered at least 6 months after the initial priming series. Although the optimal timing of a third dose has not been established, the data from this trial provide important information to address the potential need for a third booster dose in the event of waning vaccine effectiveness. Although nAb responses have been correlated to vaccine effectiveness, a correlate of protection has not been defined for Omicron or other variants^47–50^. Also, this study did not examine variant-specific booster vaccines or immune responses to variants of concern other than for Delta. Although the effect of the time interval between the second dose of mRNA-1273 in the primary series and the booster dose on antibody titers was not assessed, the time intervals between the second injection and the booster dose were similar in this study. Finally, this study showed an increase in antibody titers to the spike protein of SARS-CoV-2 but did not examine T cell memory or quantify memory B cells.

Although the data supporting the timing of when booster doses of mRNA vaccines against SARS-CoV-2 should be administered are still evolving, the results from this study provide evidence that a third dose of mRNA-1273 administered at least 6 months after the primary series is safe and effective in amplifying immune responses, as indicated by the statistically significantly higher nAb titers observed after the 50-µg booster dose as compared to titers elicited after the second dose of 100 µg of mRNA-1273 in the primary series. A booster dose of mRNA-1273 has the potential to extend the durability of vaccine efficacy and persistence of the nAb response, although this was not assessed in this study. The increased antibody responses to the Delta variant suggest that a third dose of mRNA-1273 might provide improved protection against this variant of concern. Extrapolation of these findings to other variants, including Omicron, requires further study.

## Methods

### Study design

This phase 2 study (NCT04405076) enrolled 600 participants to receive placebo or 50 µg or 100 µg of mRNA-1273 (randomized 1:1:1; Fig. 1a) in two cohorts of participants—those ≥18 to <55 years old (cohort 1) and those ≥55 years old (cohort 2)—in the observer-blinded and placebo-controlled part of the study (part A; Fig. 1). The full trial protocol is available online at *Nature Medicine*. This phase 2 trial was conducted in accordance with the International Council for Technical Requirements for Registration of Pharmaceuticals for Human Use, Good Clinical Practice guidance and applicable government regulations. All participants provided written informed consent.

Preliminary immunogenicity and safety results were previously reported^1^. Previously, the primary efficacy endpoint for mRNA-1273 against COVID-19 was met in the phase 3 efficacy study (COVE study)^2^, and 554 participants were unblinded in part B at least 5.9 months or more after enrollment in part A of the study. In total, 173 or 171 participants who had initially received two injections of 50 µg or 100 µg of mRNA-1273, respectively, then received a single booster of 50 µg of mRNA-1273.

### Trial participants

Eligible participants in part A were male or female, 18 years of age or older at screening and in good general health according to the investigator. For part B, participants must have been previously enrolled in the mRNA-1273 phase 2 study. Exclusion criteria were pregnancy or breastfeeding, acute illness or febrile (body temperature ≥38.0 °C/100.4 °F) 24 hours before or at screening or current treatment with investigational agents for prophylaxis against COVID-19.

### Randomization and unblinding

There were two age cohorts in this phase 2 study: participants ≥18 to <55 years old in cohort 1 and participants ≥55 years old in cohort 2. Within each age cohort, approximately 300 participants were randomized in a 1:1:1 ratio to receive 50 µg of mRNA-1273, 100 µg of mRNA-1273 or placebo in part A. The randomization was performed in a blinded manner using a centralized Interactive Response Technology. Vaccine dose preparation and administration during part A were performed by unblinded pharmacy personnel who did not participate in any other aspects of the study. A limited number of the sponsor team and the clinical research organization (CRO) were unblinded to enable the primary analysis at 1 month after the second dose of mRNA-1273 in part A. All study staff, participants, the CRO and sponsor personnel remained blinded to dosing assignment until the study was unblinded, upon implementation of part B of the study, following emergency use authorization of mRNA-1273 in the United States.

Participants with negative baseline SARS-CoV-2 status (*n* = 1,080) were randomly selected from the phase 3 COVE trial participants in the mRNA‑1273 group to form an immunogenicity subset that was subsequently used as the historical comparator arm for the phase 2 part B immunobridging analysis. Of the 1,080 selected participants from the phase 3 COVE trial mRNA-1273 group, 25 were further excluded from the per-protocol immunogenicity subset for the following reasons: had HIV infection (18 participants), received dose two outside of the pre-specified window (five participants), did not receive dose two per schedule (one participant) or had major protocol deviations (one participant). Thus, 1,055 participants were included in the per-protocol immunogenicity subset from the phase 3 COVE trial.

### Trial vaccine

The mRNA-1273 vaccine is a lipid nanoparticle containing an mRNA that encodes the SARS-CoV-2 spike glycoprotein of the Wuhan-HU-1 isolate^1,3^. The placebo and the mRNA-1273 vaccine were administered in the deltoid as an intramuscular injection according to a two-dose regimen in part A, with the first dose given on day 1 and the second on day 29 (28 days after dose 1). In part B, 50 µg of mRNA-1273 was administered intramuscularly in the deltoid as a single booster injection at OL-D1 (pre-booster) to the treatment groups originally vaccinated with either dose regimen of mRNA-1273. The volume administered in both part A and part B in each injection was 0.5 ml containing 50 µg or 100 µg of mRNA-1273 or saline (placebo).

### Study outcomes

Details regarding the design of part A of the study were previously published^1^. The primary safety objective of part B was to evaluate the safety and reactogenicity of 50 µg of mRNA-1273 administered as a single booster dose 6 months or more after a priming series of 50 µg or 100 µg of mRNA-1273. The primary safety endpoints were solicited local and systemic adverse reactions through 7 days after each injection; unsolicited TEAEs through 28 days after each injection; and MAAEs and SAEs throughout the entire study period.

The primary immunogenicity objective was to evaluate the immunogenicity of 50 µg of mRNA-1273 administered as a single booster dose administered at least 6 months after a two-dose priming series with 50 µg or 100 µg of mRNA-1273 as compared to 100 µg of mRNA-1273 administered as two doses 28 days apart in the pivotal phase 3 efficacy and safety study (COVE), as assessed by the level of SARS-CoV-2-specific nAbs. The ID_50_ titers were the primary focus of this analysis. Both ID_50_s and ID_80_s have been correlated with protection^4^. ID_50_s and ID_80_s are two highly correlated reportable read-outs from the same assays and samples. The ID_50_ provides a practical advantage of allowing for a wider linearity range and, thus, was preferred for this analysis. ID_80_s and ID_50_s have similar GMFRs in pseudovirus neutralization assays before the booster and 28 days after the booster (Supplementary Table 10). The conclusions using ID_80_s were consistent with those using ID_50_s.

The co-primary endpoints for non-inferiority were (1) GMTs of serum nAb and (2) seroresponse rates for nAb based on the pseudovirus nAb assay. The secondary immunogenicity objective was to evaluate the immunogenicity of 50 µg of mRNA-1273 vaccine administered as a single booster dose as assessed by the titers of broadly neutralizing antibody (bAb). Levels of SARS-CoV-2-specific bAbs were measured by ELISA and a SARS-CoV-2 MSD 3-PLEX assay on OL-D1 (pre-booster) and OL-29 (28 days after the booster injection). Seroresponse was defined in three ways: (1) seroresponse (specific to the ID_50_ titer in the pseudovirus nAb assay) was defined as a change from below the LLOQ at pre-booster (or pre-dose 1) to equal to or above the LLOQ at 28 days after the booster (or 28 days after the primary series) or at least a 3.3-fold rise at 28 days after the booster (or 28 days after the primary series) if the pre-booster (or pre-dose 1) titer was equal to or above LLOQ; (2) seroresponse (four-fold rise) was defined as a change of titer from below the LLOQ at pre-booster (or pre-dose 1) to equal to or above 4× LLOQ at 28 days after the booster (or 28 days after the primary series) or a four times or higher ratio in participants with titers above the LLOQ at pre-booster (or pre-dose 1); and (3) seroresponse (four-fold rise from baseline) was defined as a change of titer from below the LLOQ at baseline (pre-dose 1 in the primary series) to equal to or above 4× LLOQ at 28 days after the booster (or 28 days after the primary series) or a four times or higher ratio in participants with titers above the LLOQ at pre-dose 1. Definition (3) was applied only to participants in the phase 2 study (primary series in part A and booster in part B).

A comparison of the safety, reactogenicity and immunogenicty after the booster dose of mRNA-1273 in the 330 participants in part B to that in the 14,691 participants in the COVE trial who received two doses of mRNA-1273 was pre-specified in the Analysis Plan (‘Analysis Plan of immune response to a single 50 μg mRNA-1273 booster dose (P201 Part B) and to mRNA-1273 100 μg primary series (P301)’, version 2.0, 6 August 2021).

### Safety assessment

Solicited local and systemic adverse reactions were recorded daily by participants in an electronic diary during the 7 days after vaccine administration. Any solicited adverse reaction that persisted beyond day 7 was reported in the electronic diary until resolution. Oral body temperatures were measured daily. If applicable, the size of injection site erythema and swelling/induration were measured and recorded. In part B, trained site personnel called trial participants every 4 weeks to assess safety beginning 3 months after the booster dose.

### Immunogenicity assessments

#### SARS-CoV-2 spike-pseudotyped virus neutralization assay

This validated assay quantifies SARS-CoV-2 nAbs by using lentivirus particles that express SARS-CoV-2 full-length spike proteins (Wuhan-Hu-1 isolate including the amino acid change of D614G in the spike protein or the Delta variant (B.1.617.2; AY.3; Wuhan-Hu-1 isolate containing spike mutations T19R, G142D, Δ156- 157, R158G, L452R, T478K, D614G, P681R and D950N)) on their surface and contain a firefly luciferase reporter gene for quantitative measurements of infection by relative light units (RLU)^5^. During assay development, it was quickly demonstrated that the D614G variant behaved in the pseudo-neutralization assay in an indistinguishable manner when compared to the original Wuhan spike protein. The pseudovirus neutralization assay was formally optimized; qualified and validated for accuracy, sensitivity, specificity, linearity, range, precision, limits of quantitation and robustness^6^; and approved by the FDA. The assay uses a full-length spike protein with no cytoplasmic tail deletions. The spike protein in the assay does not contain the two proline mutations in the vaccine spike that stabilize the protein in a pre-fusion conformation.

The pseudoviruses were applied to transduced 293T cells expressing high levels of ACE2 (293T/ACE2 cells), with or without pre-incubation with antibodies (control antibodies or serum samples); the presence of nAbs reduced infection and resulted in lower RLUs. Serial dilution of antibodies or serum samples were used to produce a dose–response curve. Neutralization was measured as the serum dilution at which the RLU was reduced by 50% (ID_50_) or 80% (ID_80_) relative to mean RLU in virus control wells (cells + virus but no control antibody or sample) after subtraction of the mean RLU in cell control wells (cells only).

### SARS-CoV-2 MSD 3-PLEX assay

This quantitative electrochemiluminescence (ECL) method is an indirect binding ECL method designed to detect SARS-CoV-2 antibodies (SARS-CoV-2 full-length spike (Wuhan-Hu-1 isolate including D614G), nucleocapsid (N) and receptor-binding domain (RBD) antibodies) in human serum. The assay is based on the MSD technology that employs capture molecule MultiSPOT microtiter plates fitted with a series of electrodes. Using an MSD MESO SECTOR S 600 detection system, an electrical current was applied to the custom microtiter plates, leading to a light emission by SULFO-TAGTM through a series of oxidation-reduction reactions involving ruthenium and tripropylamine (TPA). A plate reader measured the intensity of emitted light to provide quantitative measures of analytes in samples.

For this bio-assay, a ten-spot custom SARS-CoV-2 3-PLEX plate coated with SARS-CoV-2 antigens (S (containing D614G), N and RBD) was used. Anti-SARS-CoV-2 antibodies present in the test sample bound to the antigen-coated plates and formed antibody–antigen complexes. These complexes were detected by adding SULFO-TAGTM-labeled antibodies, which bind to the antibody–antigen complexes. Addition of TPA in a buffer solution resulted in ECL that was measured in RLU using the MSD SECTOR S 600 plate reader. Antibody concentrations were determined by interpolating their ECL response using the standard curve generated from a serially diluted reference standard.

### SARS-CoV-2 S-2P IgG ELISA

This validated assay uses microtiter plates coated with commercially available SARS-CoV-2 full-length spike glycoprotein (Wuhan-Hu-1 isolate including D614G). Serum containing the SARS-CoV-2 IgG antibody was added to the plates. Bound antigen–antibody complex was detected using purified goat anti-human IgG horseradish peroxidase conjugate. Color development occurred with the addition of 3,3′,5,5′-tetramethylbenzidine substrate, and color intensity was measured spectrophotometrically (450 nm). The intensity of the color was directly proportional to the IgG antibody concentration. Quantitation of the human IgG antibody to SARS-CoV-2 or antibody concentration (AU ml^−1^) was determined by interpolation from a standard curve analyzed on each assay plate.

### Statistical analyses

There was no hypothesis testing in this study. With respect to the sample size, the number of proposed participants was considered sufficient to provide a descriptive summary of the safety and immunogenicity of different dose levels of mRNA-1273 in the primary series.

The results for the two groups that received a booster injection after a primary series of two doses of 50 µg or 100 µg of mRNA-1273 were expected to be similar and have been combined for the immunogenicity analysis to increase the statistical power for comparisons to the historical control from the phase 3 COVE trial.

The safety analyses were descriptive without pre-specified statistical criteria. The safety set for part B booster included all participants who were randomized in part A and received a booster injection during part B. The solicited safety set for part B booster consisted of all participants who were randomized to mRNA-1273 (50 µg or 100 µg) in part A, received a booster injection during part B and contributed any solicited adverse reaction data (that is, reported at least one post-baseline solicited safety assessment in part B). The solicited safety set was used for the analyses of solicited adverse reactions. The per-protocol set for part B booster consisted of all part B booster participants who had both pre-booster and post-booster immunogenicity assessments at OL-D1 (pre-booster) and OL-D29 (28 days after the booster injection)); who did not have evidence of past or current SARS-CoV-2 infection at OL-D1 for part B, where SARS-CoV-2 infection was defined as a positive RT–PCR test for SARS-CoV-2 and/or a positive serology test based on bAbs specific to SARS-CoV-2 nucleocapsid (as measured by Roche Elecsys Anti-SARS-CoV-2 assay); and who had no major protocol deviations that affected immune response during the period corresponding to the immunogenicity analysis objective in part B. The per-protocol immunogenicity set for the booster in part B served as the primary population for the analysis of immunogenicity data in part B.

The GMTs of bAb or nAb titers with corresponding 95% CIs are provided at each time point. The GMFR of bAb or nAb titers and the corresponding 95% CIs are also provided. The 95% CIs were calculated based on the t-distribution of the log-transformed values or the difference in the log-transformed values for GMT and GMFR, respectively, and then back-transformed to the original scale for presentation. For calculation of GMTs and GMFRs, antibody values reported as below the LLOQ were replaced by 0.5× LLOQ. Values that were greater than the upper limit of quantification (ULOQ) were converted to the ULOQ if the actual values were not available. Missing results were not imputed.

To assess the magnitudes of the differences in immune response 28 days after a single booster dose of 50-μg mRNA-1273 and the immune response 28 days after the completion of the primary series of 100-μg mRNA-1273 in the phase 3 COVE study, an analysis of covariance (ANCOVA) model was used. The model included log-transformed antibody titers at D29 after booster in this phase 2 study and D57 in the COVE study as the dependent variable and treatment groups (50-μg mRNA-1273 booster in phase 2, 100-μg primary series in COVE) as the explanatory variable, adjusting for age groups (<65 years and ≥65 years—age groups used in the COVE study). The geometric least squares mean (GLSM) and corresponding two-sided 95% CI for the antibody titers for each treatment group are provided. The GLSM and the corresponding 95% CI results in log-transformed scale estimated from the model were back-transformed to obtain these estimates in the original scale. GMR, estimated by the ratio of GLSM and the corresponding two-sided 95% CI, was provided to assess the treatment difference.

The primary immunogenicity objective in part B was considered met if the non-inferiority based on both GMTs and seroresponse rate at 28 days after the booster in part B compared with 28 days after the second dose in the phase 3 COVE trial was demonstrated, at a two‑sided alpha of 0.05. The null hypotheses based on GMTs and seroresponse rate and the criterion of success included: non-inferiority based on the GMR (28 days after the booster in part B versus 28 days after the second dose in the phase 3 COVE trial) was pre-defined with a non-inferiority margin of 1.5 and a point estimate of GMR ≥ 1; and non-inferiority based on difference in seroresponse rate (28 days after the booster in part B minus 28 days after the second dose in the phase 3 COVE trial) was pre-defined with a non-inferiority margin of 10%.

All analyses were conducted using SAS version 9.4 or higher.

### Reporting Summary

Further information on research design is available in the Nature Research Reporting Summary linked to this article.

## Online content

Any methods, additional references, Nature Research reporting summaries, source data, extended data, supplementary information, acknowledgements, peer review information; details of author contributions and competing interests; and statements of data and code availability are available at 10.1038/s41591-022-01739-w.

## Supplementary information

Supplementary Information Supplementary Information: List of Investigators, Supplementary Tables 1–10, Supplementary Figs. 1–5, Protocol Amendment Summary of Changes and References

Reporting Summary

Supplementary Data A phase 2a, randomized, observer-blind, placebo-controlled, dose-confirmation study to evaluate the safety, reactogenicity and immunogenicity of mRNA-1273 SARS-CoV-2 vaccine in adults aged 18 years and older

## Data Availability

As the trial is ongoing, access to patient-level data presented in this article (antibody assays, safety and reactogenicity) and supporting clinical documents with external researchers who provide methodologically sound scientific proposals will be available upon reasonable request and subject to review once the trial is complete. Such requests can be made to Moderna Inc., 200 Technology Square, Cambridge, MA 02139. A materials transfer and/or data access agreement with the sponsor will be required for accessing shared data. All other relevant data are presented in the paper. The protocol is available as online supplementary material to this article. ClinicalTrials.gov: NCT04405076.
