# Supplementary material for: Immune response to SARS-CoV-2 after a booster of mRNA-1273: an open-label phase 2 trial
Source: Nat Med. 2022 Mar 3;28(5):1042–9. doi: 10.1038/s41591-022-01739-w (PMC9117133; doi:10.1038/s41591-022-01739-w)
Supplement: Supplementary file 1 — Supplementary Information: List of Investigators, Supplementary Tables 1–10, Supplementary Figs. 1–5, Protocol Amendment Summary of Changes and References [file 41591_2022_1739_MOESM1_ESM.pdf]

nature portfolio

https://doi.org/10.1038/s41591-022-01739-w

Supplementary information

Immune response to SARS-CoV-2 after a booster of mRNA-1273: an open-label phase 2 trial

In the format provided by the authors and unedited

Supplementary Information

Table of Contents	Pg.

List of Investigators………………………………………………………………………………………………. 3

Supplementary Table 1. Solicited Local Adverse Reactions Reported Within 7 Days After 50 µg Booster in Phase 2 versus After the 2nd Injection in the Primary Series of Phase 2 Part A or Phase 3 COVE (Solicited Safety Set)………………………………………………………... 4

Supplementary Table 2: Solicited Systemic Adverse Reactions Reported Within 7 Days After 50 µg Booster in Phase 2 versus After the 2nd Injection in the Primary Series of Phase 2 Part A or Phase 3 COVE (Solicited Safety Set)……………………………………………….. 5

Supplementary Table 3: Solicited Adverse Reactions Within 7 Days After the Booster injection by Age Groups– Solicited Safety Set……………………………………………………… 7

Supplementary Table 4: Unsolicited Treatment-emergent Adverse Events (TEAEs) up to 28 days after Booster Injection………………………………………………………………………………… 9

Supplementary Table 5: Neutralizing Antibody Titers (Pseudovirus ID50 versus D614G) after the Primary Series and a Booster Injection of 50 µg of mRNA-1273 (Per-protocol Set)…… 10

Supplementary Table 6: Comparison of Antibody Responses in Three Assays Versus D614G After Boosting with Those After Completion of Primary Series……………………………… 11

Supplementary Table 7: Spike Binding IgG Antibody versus D614G by ELISA (VAC65) After the Primary Series and a 50 µg Booster Injection – Per-protocol Immunogenicity Subset …….. 13

Supplementary Table 8: Neutralizing Antibody Titers (Pseudovirus ID50 versus the Delta Variant) after the Primary Series and a Booster Injection of 50 µg of mRNA-1273 (Per-protocol Set)……………………………………………………………………………………………… 14

Supplementary Table 9: Summary of Geometric Means of PsVNA ID50 Titers and Ratios by Age Group.. 15

Supplementary Table 10: Comparison of Pseudovirus Neutralization ID50 and ID80 Titers... 16

Supplementary Figure 1: Participant Flow Diagram …………………………………………………… 17

Supplementary Figure 2: Neutralizing Antibody Titers (Pseudovirus ID50) After the Primary Series and After a Booster Injection of 50 µg of mRNA-1273 (Per-protocol Set).. 18

Supplementary Figure 3: Spike-binding IgG Antibody Titers (VAC65 ID50; D614G) After the Primary Series and After a Booster Injection of 50 µg of mRNA-1273 (Per-protocol Set)……. 20

Supplementary Figure 4: Box and Whisker Plot of Spike-binding IgG Antibody Titers (VAC65 ID50; D614G) After the Primary Series and After a Booster Injection of 50 µg of mRNA-1273 (Per-protocol Set)………………………………………………………………………………. 22

Supplementary Figure 5: Neutralizing Antibody Titers (Pseudovirus ID50; D614G) Before and After a Booster Injection of 50 µg of mRNA-1273……………………………………… 23

Protocol Amendment Summary of Changes…….………………………………………………………… 24

References……………………………………………………………………………………………………. 40

List of Investigators

Investigator	Institution	Location
Laurence Chu	Benchmark Research	Austin, Texas
Paul Bradley	Meridian Clinical Research	Savannah, Georgia
Keith Vbricky	Meridian Clinical Research	Norfolk, Nebraska
David Ensz	Meridian Clinical Research	Sioux City, Iowa
John Ervin	Alliance for Multispecialty Research, LLC	Kansas City, Missouri
Richard Glover	Alliance for Multispecialty Research, LLC	Newton, Kansas
Darrell Herrington	Benchmark Research	San Angelo, Texas
Barton Williams	Trial Management Associates	Wilmington, North Carolina

Supplementary Table 1: Solicited Local Adverse Reactions Reported Within 7 Days After 50 µg Booster in Phase 2 versus After the 2nd Injection in the Primary Series of Phase 2 Part A or Phase 3 COVE (Solicited Safety Set)

	mRNA-1273				
	Phase 2, Part B, 50 µg Prime + 50 µg Booster (N=163) n (%)	Phase 2, Part B, 100 µg Prime + 50 µg Booster (N=167) n (%)	Phase 2, Part B, (Pooled 50 and 100 µg Prime), 50 µg Booster (N=330) n (%)	Phase 2, Part A, 100 µg Prime (Dose 2) (N=198) n (%)	Phase 3 COVE, 100 µg Prime (Dose 2) (N= 14691) n (%)
**Any Local AR, N1**	162	167	329	198	14688
**Any**	144 (88.9)	143 (85.6)	287 (87.2)	170 (85.9)	13029 (88.7)
**Grade 1**	102 (63.0)	108 (64.7)	210 (63.8)	129 (65.2)	8789 (59.8)
**Grade 2**	33 (20.4)	27 (16.2)	60 (18.2)	34 (17.2)	3217 (21.9)
**Grade 3**	9 (5.6)	8 (4.8)	17 (5.2)	7 (3.5)	1023 (7.0)
**Pain, N1**	162	167	329	198	14688
**Any**	144 (88.9)	140 (83.8)	284 (86.3)	169 (85.4)	12964 (88.3)
**Grade 1**	111 (68.5)	111 (66.5)	222 (67.5)	140 (70.7)	9508 (64.7)
**Grade 2**	26 (16.0)	23 (13.8)	49 (14.9)	28 (14.1)	2850 (19.4)
**Grade 3**	7 (4.3)	6 (3.6)	13 (4.0)	1 (0.5)	606 (4.1)
**Erythema (Redness), N1**	162	167	329	198	14687
**Any**	10 (6.2)	8 (4.8)	18 (5.5)	15 (7.6)	1274 (8.7)
**Grade 1**	4 (2.5)	5 (3.0)	9 (2.7)	7 (3.5)	456 (3.1)
**Grade 2**	4 (2.5)	2 (1.2)	6 (1.8)	3 (1.5)	531 (3.6)
**Grade 3**	2 (1.2)	1 (0.6)	3 (0.9)	5 (2.5)	287 (2.0)
**Swelling (Hardness), N1**	162	167	329	198	14687
**Any**	12 (7.4)	9 (5.4)	21 (6.4)	21 (10.6)	1807 (12.3)
**Grade 1**	4 (2.5)	4 (2.4)	8 (2.4)	14 (7.1)	900 (6.1)
**Grade 2**	7 (4.3)	4 (2.4)	11 (3.3)	6 (3.0)	652 (4.4)
**Grade 3**	1 (0.6)	1 (0.6)	2 (0.6)	1 (0.5)	255 (1.7)
**Lymphadenopathy, N1**	162	167	329	198	14687
**Any**	35 (21.6)	34 (20.4)	69 (21.0)	20 (10.1)	2092 (14.2)
**Grade 1**	22 (13.6)	30 (18.0)	52 (15.8)	17 (8.6)	1735 (11.8)
**Grade 2**	13 (8.0)	3 (1.8)	16 (4.9)	3 (1.5)	289 (2.0)
**Grade 3**	0	1 (0.6)	1 (0.3)	0	68 (0.5)

Supplementary Table 1: N1 = Number of exposed subjects who submitted any data for the event. Percentages are based on the number of exposed subjects who submitted any data for the event (N1).

Supplementary Table 2: Solicited Systemic Adverse Reactions Reported Within 7 Days After 50 µg Booster in Phase 2 versus After the 2nd Injection in the Primary Series of Phase 2 Part A or Phase 3 COVE (Solicited Safety Set)

	mRNA-1273				
	**Phase 2, Part B, 50 µg Prime + 50 µg Booster (N=163) n (%)**	**Phase 2, Part B, 100 µg Prime + 50 µg Booster N=167 n (%)**	**Phase 2, Part B, 50 µg Booster (Pooled 50 and 100 µg Prime) (N=330) n (%)**	**Phase 2, Part A, 100 µg Prime (Dose 2) N=198 n (%)**	**Phase 3 COVE, 100 µg Prime (Dose 2) N=14691 n (%)**
**Any Systemic AR, N1**	163	167	330	198	14690
**Any**	127 (77.9)	126 (75.4)	253 (76.7)	153 (77.3)	11678 (79.5)
**Grade 1**	49 (30.1)	60 (35.9)	109 (33.0)	56 (28.3)	3717 (25.3)
**Grade 2**	56 (34.4)	53 (31.7)	109 (33.0)	72 (36.4)	5611 (38.2)
**Grade 3**	21 (12.9)	12 (7.2)	33 (10.0)	25 (12.6)	2336 (15.9)
**Grade 4**	0	0	0	0	14 (<0.1)
Fever, N1	162	166	328	198	14682
Any	13 (8.0)	11 (6.6)	24 (7.3)	38 (13.1)	2276 (15.5)
Grade 1	12 (7.4)	6 (3.6)	18 (5.5)	19 (9.6)	1363 (9.3)
Grade 2	1 (0.6)	3 (1.8)	4 (1.2)	3 (1.5)	697 (4.7)
Grade 3	0	2 (1.2)	2 (0.6)	4 (2.0)	203 (1.4)
Grade 4	0	0	0	0	13 (<0.1)
Headache, N1	162	167	329	198	14687
Any	97 (59.9)	92 (55.1)	189 (57.4)	104 (52.5)	8637 (58.8)
Grade 1	57 (35.2)	61 (36.5)	118 (35.9)	56 (28.3)	4815 (32.8)
Grade 2	34 (21.0)	29 (17.4)	63 (19.1)	39 (19.7)	3156 (21.5)
Grade 3	6 (3.7)	2 (1.2)	8 (2.4)	9 (4.5)	666 (4.5)
Fatigue, N1	162	167	329	198	14687
Any	103 (63.6)	98 (58.7)	201 (61.1)	128 (64.6)	9607 (65.4)
Grade 1	40 (24.7)	47 (28.1)	87 (26.4)	44 (22.2)	3431 (23.4)
Grade 2	50 (30.9)	44 (26.3)	94 (28.6)	66 (33.3)	4743 (32.3)
Grade 3	13 (8.0)	7 (4.2)	20 (6.1)	18 (9.1)	1433 (9.8)
Myalgia, N1	162	167	329	198	14687
Any	86 (53.1)	82 (49.1)	168 (51.1)	104 (52.5)	8529 (58.1)
Grade 1	40 (24.7)	47 (28.1)	87 (26.4)	35 (17.7)	3242 (22.1)
Grade 2	37 (22.8)	30 (18.0)	67 (20.4)	54 (27.3)	3966 (27.0)
Grade 3	9 (5.6)	5 (3.0)	14 (4.3)	15 (7.6)	1321 (9.0)

Arthralgia, N1	162	167	329	198	14687
Any	66 (40.7)	69 (41.3)	135 (41.0)	77 (38.9)	6303 (42.9)
Grade 1	35 (21.6)	43 (25.7)	78 (23.7)	32 (16.2)	2809 (19.1)
Grade 2	23 (14.2)	21 (12.6)	44 (13.4)	37 (18.7)	2719 (18.5)
Grade 3	8 (4.9)	5 (3.0)	13 (4.0)	8 (4.0)	775 (5.3)
Nausea/Vomiting, N1	162	167	329	198	14687
Any	29 (17.9)	19 (11.4)	48 (14.6)	41 (20.7)	2794 (19.0)
Grade 1	25 (15.4)	16 (9.6)	41 (12.5)	25 (12.6)	2094 (14.3)
Grade 2	4 (2.5)	3 (1.8)	7 (2.1)	16 (8.1)	678 (4.6)
Grade 3	0	0	0	0	21 (0.1)
Grade 4	0	0	0	0	1 (<0.1)
Chills, N1	162	167	329	198	14687
Any	62 (38.3)	59 (35.3)	121 (36.8)	78 (39.4)	6500 (44.3)
Grade 1	32 (19.8)	36 (21.6)	68 (20.7)	30 (15.2)	2907 (19.8)
Grade 2	28 (17.3)	23 (13.8)	51 (15.5)	47 (23.7)	3402 (23.2)
Grade 3	2 (1.2)	0	2 (0.6)	1 (0.5)	191 (1.3)

Supplementary Table 2: N1 = Number of exposed subjects who submitted any data for the event. NR = not reported.

Percentages are based on the number of exposed subjects who submitted any data for the event (N1).

Supplementary Table 3: Solicited Adverse Reactions Within 7 Days After the Booster injection by Age Groups– Solicited Safety Set

Adverse Reaction n (%)	≥18 to <55 yrs			≥55 yrs		
	50 µg Prime N=73	100 µg Prime N=79	Pooled 50 µg + 100 µg Prime N=152	50 µg Prime N=90	100 µg Prime N=88	Pooled 50 µg + 100 µg Prime N=178
Solicited AR, N1	73	79	152	90	88	178
Any Solicited AR	68 (93.2)	73 (92.4)	141 (92.8)	84 (93.3)	78 (88.6)	162 (91.0)
Grade 1	33 (45.2)	30 (38.0)	63 (41.4)	32 (35.6)	43 (48.9)	75 (42.1)
Grade 2	24 (32.9)	33 (41.8)	57 (37.5)	34 (37.8)	26 (29.5)	60 (33.7)
Grade 3	11 (15.1)	9 (11.4)	20 (13.2)	18 (20.0)	9 (10.2)	27 (15.2)
Any Solicited Local AR, N1	72	79	151	90	88	178
Any Solicited Local AR	66 (91.7)	69 (87.3)	135 (89.4)	78 (86.7)	74 (84.1)	152 (85.4)
Grade 1	49 (68.1)	48 (60.8)	97 (64.2)	53 (58.9)	60 (68.2)	113 (63.5)
Grade 2	13 (18.1)	17 (21.5)	30 (19.9)	20 (22.2)	10 (11.4)	30 (16.9)
Grade 3	4 (5.6)	4 (5.1)	8 (5.3)	5 (5.6)	4 (4.5)	9 (5.1)
Local AR, Pain, N1	72	79	151	90	88	178
Pain	66 (91.7)	68 (86.1)	134 (88.7)	78 (86.7)	72 (81.8)	150 (84.3)
Grade 1	51 (70.8)	50 (63.3)	101 (66.9)	60 (66.7)	61 (69.3)	121 (68.0)
Grade 2	11 (15.3)	15 (19.0)	26 (17.2)	15 (16.7)	8 (9.1)	23 (12.9)
Grade 3	4 (5.6)	3 (3.8)	7 (4.6)	3 (3.3)	3 (3.4)	6 (3.4)
Erythema, N1	72	79	151	90	88	178
Erythema	4 (5.6)	5 (6.3)	9 (6.0)	6 (6.7)	3 (3.4)	9 (5.1)
Grade 1	2 (2.8)	2 (2.5)	4 (2.6)	2 (2.2)	3 (3.4)	5 (2.8)
Grade 2	2 (2.8)	2 (2.5)	4 (2.6)	2 (2.2)	0	2 (1.1)
Grade 3	0	1 (1.3)	1 (0.7)	2 (2.2)	0	2 (1.1)
Swelling, N1	72	79	151	90	88	178
Swelling	5 (6.9)	5 (6.3)	10 (6.6)	7 (7.8)	4 (4.5)	11 (6.2)
Grade 1	3 (4.2)	3 (3.8)	6 (4.0)	1 (1.1)	1 (1.1)	2 (1.1)
Grade 2	2 (2.8)	2 (2.5)	4 (2.6)	5 (5.6)	2 (2.3)	7 (3.9)
Grade 3	0	0	0	1 (1.1)	1 (1.1)	2 (1.1)
Axillary Swelling, N1	72	79	151	90	88	178
Axillary Swelling	17 (23.6)	22 (27.8)	39 (25.8)	18 (20.0)	12 (13.6)	30 (16.9)
Grade 1	11 (15.3)	18 (22.8)	29 (19.2)	11 (12.2)	12 (13.6)	23 (12.9)
Grade 2	6 (8.3)	3 (3.8)	9 (6.0)	7 (7.8)	0	7 (3.9)
Grade 3	0	1 (1.3)	1 (0.7)	0	0	0
Any Systemic AR, N1	73	79	152	90	88	178
Any Systemic AR	55 (75.3)	59 (74.7)	114 (75.0)	72 (80.0)	67 (76.1)	139 (78.1)
Grade 1	24 (32.9)	23 (29.1)	47 (30.9)	25 (27.8)	37 (42.0)	62 (34.8)
Grade 2	22 (30.1)	29 (36.7)	51 (33.6)	34 (37.8)	24 (27.3)	58 (32.6)
Grade 3	8 (11.0)	6 (7.6)	14 (9.2)	13 (14.4)	6 (6.8)	19 (10.7)
Fever, N1	72	79	151	90	87	177

Fever	4 (5.6)	6 (7.6)	10 (6.6)	9 (10.0)	5 (5.7)	14 (7.9)
Grade 1	4 (5.6)	4 (5.1)	8 (5.3)	8 (8.9)	2 (2.3)	10 (5.6)
Grade 2	0	1 (1.3)	1 (0.7)	1 (1.1)	2 (2.3)	3 (1.7)
Grade 3	0	1 (1.3)	1 (0.7)	0	1 (1.1)	1 (0.6)
Headache, N1	72	79	151	90	88	178
Headache	41 (56.9)	45 (57.0)	86 (57.0)	56 (62.2)	47 (53.4)	103 (57.9)
Grade 1	23 (31.9)	28 (35.4)	51 (33.8)	34 (37.8)	33 (37.5)	67 (37.6)
Grade 2	16 (22.2)	16 (20.3)	32 (21.2)	18 (20.0)	13 (14.8)	31 (17.4)
Grade 3	2 (2.8)	1 (1.3)	3 (2.0)	4 (4.4)	1 (1.1)	5 (2.8)
Fatigue, N1	72	79	151	90	88	178
Fatigue	44 (61.1)	46 (58.2)	90 (59.6)	59 (65.6)	52 (59.1)	111 (62.4)
Grade 1	18 (25.0)	18 (22.8)	36 (23.8)	22 (24.4)	29 (33.0)	51 (28.7)
Grade 2	22 (30.6)	25 (31.6)	47 (31.1)	28 (31.1)	19 (21.6)	47 (26.4)
Grade 3	4 (5.6)	3 (3.8)	7 (4.6)	9 (10.0)	4 (4.5)	13 (7.3)
Myalgia, N1	72	79	151	90	88	178
Myalgia	37 (51.4)	37 (46.8)	74 (49.0)	49 (54.4)	45 (51.1)	94 (52.8)
Grade 1	20 (27.8)	19 (24.1)	39 (25.8)	20 (22.2)	28 (31.8)	48 (27.0)
Grade 2	13 (18.1)	15 (19.0)	28 (18.5)	24 (26.7)	15 (17.0)	39 (21.9)
Grade 3	4 (5.6)	3 (3.8)	7 (4.6)	5 (5.6)	2 (2.3)	7 (3.9)
Arthralgia, N1	72	79	151	90	88	178
Arthralgia	24 (33.3)	34 (43.0)	58 (38.4)	42 (46.7)	35 (39.8)	77 (43.3)
Grade 1	13 (18.1)	20 (25.3)	33 (21.9)	22 (24.4)	23 (26.1)	45 (25.3)
Grade 2	7 (9.7)	12 (15.2)	19 (12.6)	16 (17.8)	9 (10.2)	25 (14.0)
Grade 3	4 (5.6)	2 (2.5)	6 (4.0)	4 (4.4)	3 (3.4)	7 (3.9)
Nausea/ vomiting, N1	72	79	151	90	88	178
Nausea / vomiting	17 (23.6)	12 (15.2)	29 (19.2)	12 (13.3)	7 (8.0)	19 (10.7)
Grade 1	15 (20.8)	12 (15.2)	27 (17.9)	10 (11.1)	4 (4.5)	14 (7.9)
Grade 2	2 (2.8)	0	2 (1.3)	2 (2.2)	3 (3.4)	5 (2.8)
Grade 3	0	0	0	0	0	0
Chills, N1	72	79	151	90	88	178
Chills	28 (38.9)	30 (38.0)	58 (38.4)	34 (37.8)	29 (33.0)	63 (35.4)
Grade 1	15 (20.8)	20 (25.3)	35 (23.2)	17 (18.9)	16 (18.2)	33 (18.5)
Grade 2	12 (16.7)	10 (12.7)	22 (14.6)	16 (17.8)	13 (14.8)	29 (16.3)
Grade 3	1 (1.4)	0	1 (0.7)	1 (1.1)	0	1 (0.6)

Supplementary Table 3: N1=Number of exposed participants who submitted any data for the event. Percentages are based on the number of exposed participants who submitted any data for the event (N1).

Supplementary Table 4: Unsolicited Treatment-emergent Adverse Events (TEAEs) up to 28 days after Booster Injection

	50 µg Prime, Booster (N=173) n (%)	100 µg Prime, Booster (N=171) n (%)	50 µg and 100 µg Prime, Booster (N=344) n (%)
Unsolicited TEAEs regardless of relationship to study vaccination			
All	17 (9.8)	22 (12.9)	39 (11.3)
Serious	0	0	0
Fatal	0	0	0
Medically-attended	8 (4.6)	12 (7.0)	20 (5.8)
Leading to study discontinuation	0	0	0
Severe	0	0	0
Unsolicited TEAEs related to study vaccination			
All	6 (3.5)	7 (4.1)	13 (3.8)
Serious	0	0	0
Fatal	0	0	0
Medically-attended	0	2 (1.2)	2 (0.6)
Leading to study discontinuation	0	0	0
Severe	0	0	0

Supplementary Table 4: A treatment-emergent adverse event was defined as any event not present before exposure to study vaccination or any event already present that worsens in intensity or frequency after exposure. Percentages are based on the number of participants in the safety set.

Supplementary Table 5: Neutralizing Antibody Titers (Pseudovirus ID50 versus D614G) after the Primary Series and a Booster Injection of 50 µg of mRNA-1273 (Per-protocol Set)

	50 µg Prime N=146	100 µg Prime N=149	50 µg and 100 µg Prime N=295
Baseline (Day 1), n*	146	148	294
GMT	9.4	9.3	9.3
95% CI	9.2, 9.5	NE	9.2, 42.7
Day 29 (28 Days after 1st injection), n	144	146	290
GMT	27.3	36.4	31.6
95% CI	23.3, 31.8	30.8, 43.1	28.1, 35.4
Day 57 (28 Days after 2nd injection), n	143	146	289
GMT	629.2	1268.0	896.5
95% CI	549.3, 720.8	1087.9, 1477.8	803.4, 1000.4
Open-label, Day 1 (OL-D1; Pre-Booster), n*	145	149	294
GMT	104.7	150.2	125.7
95% CI[†]	88.3, 124.1	125.7, 179.5	111.0, 142.3
Open-label Day 29 (OL-D29; 28 Days After Booster), n[‡]	146	149	295
GMT	1834.3	1951.7	1892.7
95% CI	1600.2, 2102.6	1729.6, 2202.4	1728.8, 2072.2
Comparison of OL-D29 after the booster to Day 57, n	143	146	289
GM Fold Rise	2.9	1.5	2.1
95% CI	2.6, 3.4	1.3, 1.8	1.9, 2.3

Supplementary Table 5: Antibody values reported as below the lower limit of quantification (LLOQ; 18.5) were replaced by 0.5 x LLOQ. Values that were greater than the upper limit of quantification (ULOQ; 45118) were converted to the ULOQ if actual values were not available.
*n=Number of subjects with non-missing baseline results.
[†]95% Confidence Intervals were calculated based on the t-distribution of the log-transformed values or the difference in the log-transformed values for GMT and GMFR, respectively, then back transformed to the original scale.
[‡] Number of subjects in the Per-Protocol Set with non-missing data at the corresponding visit.

Supplementary Table 6: Comparison of Antibody Responses in Three Assays Versus D614G After Boosting with Those After Completion of Primary Series

	Boosting with 50 µg of mRNA-1273 after primary series* [Phase 2 Part B]	Primary Series of Two Injections of 100 µg of mRNA-1273 [Phase 3 COVE random sub-cohort]
**Pseudovirus Neutralizing Antibody (ID50)**		
Baseline, n	294	1052
Baseline, GMT (95% CI)	125.7 (111.0, 142.3)	9.6 (9.4, 9.9)
GMT at 28 Days after boosting or at 28 Days after second injection of mRNA-1273 during primary series (95% CI)	1892.7 (1728.8, 2072.2)	1081.1 (1019.8, 1146.1)
Seroresponse Rate†, n	294	1050
% (95% CI)	90.1 (86.1, 93.3)	98.4 (97.4, 99.1)
Difference in seroresponse rates†, % (95% CI)	-8.2 (-12.2, -5.2)	
**Anti-Spike ELISA (VAC65)**		
Baseline, n	290	1052
Baseline GMT (95% CI)	97.4 (89.4, 106.1)	0.7 (0.7, 0.8)
GMT at 28 Days after boosting or at 28 Days after second injection of mRNA-1273 during primary series (95% CI)	1074.7 (1024.1, 1127.8)	694.8 (664.8, 726.1)
Seroresponse Rate, % (95% CI)	94.7 (91.4, 97.0)	99.6 (99.0, 99.9)
Difference in seroresponse rates, % (95% CI)†	-4.9 (-8.2, -2.8)	
**Anti-Spike IgG Antibody by MSD MULTIPLEX**		
Baseline, n	290	1046
Baseline, GMT (95% CI)	34,827.3 (31,546.4, 38, 449.5)	114.8 (110.8, 119.0)
28 Days after boosting or at 28 Days after second injection of mRNA-1273 during primary series, n	289	1035
GMT (95% CI)	569,716.1 (536,434.6, 605,062.5)	316,448.3 (300,071.4, 333,719.0)
Seroresponse Rate, n	284	1027
% (95% CI)	98.2 (95.9, 99.4)	99.4 (98.7, 99.8)
Difference in seroresponse rates, % (95% CI)†	-1.2 (-3.5, 0.0)	

Supplementary Table 6: GMT: Geometric Mean Titer, Observed.

* Overall: Combined 50 µg and 100 µg prime groups

† Seroresponse at participant level was defined as a change of titer from below the lower limit of quantification (LLOQ) to equal to or above 4 times the LLOQ, or a 4-times or higher ratio in participants with titers above LLOQ. The difference in seroresponse rates was Boosting minus Primary Series.

For participants who received the primary series, seroresponse was defined based on the fold-rise from baseline titer (prior to first dose of primary dose). For participants who received a booster vaccination,

seroresponse was defined based on the fold-rise from pre-booster titer (at least 6 months after completion of the primary series).

Supplementary Table 7: Spike Binding IgG Antibody versus D614G by ELISA (VAC65) After the Primary Series and a 50 µg Booster Injection – Per-protocol Immunogenicity Subset

	50 µg Prime N=185	100 µg Prime N=189	50 µg and 100 µg Prime N=374
Baseline (Day 1), n*	185	189	374
GMT	0.7	0.67	0.7
95% CI	0.6, 0.8	0.6, 0.7	0.6, 0.7
28 days after 1st injection, n	183	189	372
GMT	59.4	81.7	69.8
95% CI	52.4, 67.4	72.1, 92.5	63.9, 76.4
14 days after 2nd injection, n	176	180	356
GMT	716.6	835.7	774.5
95% CI	655.8, 782.9	765.7, 912.1	727.6, 824.5
28 days after 2nd injection, n	176	174	350
GMT	516.7	651.8	580.0
95% CI	469.9, 568.2	592.4, 717.1	541.9, 620.8
Day 209, n	170	174	344
GMT	95.7	129.4	111.5
95% CI	82.7, 110.7	112.1, 149.5	100.6, 123.6
Pre-booster, n	144	146	290
GMT	86.4	109.6	97.4
95% CI	76.9, 97.0	96.8, 124.2	89.4, 106.1
Day 28 After Booster, n	142	147	289
GMT	1068.8	1080.4	1074.7
95% CI	991.6, 1152.1	1015.5, 1149.4	1024.1, 1127.8
Comparison of 28 days after booster to 28 days after primary series, n	142	145	287
GM Fold Rise	2.1	1.7	1.9
95% CI	1.9, 2.2	1.5, 1.9	1.7, 2.0

Supplementary Table 7: GMT = Geometric Mean Titer. CI = Confidence intervals.
*n = Number of participants in the Per-Protocol Set with non-missing data at baseline and the corresponding visit.
Antibody values reported as below the lower limit of quantification (LLOQ) were replaced by 0.5 x LLOQ. Values that were greater than the upper limit of quantification (ULOQ) were converted to the ULOQ if actual values are not available. Percentages are based on the number of participants in the Per-Protocol Set with non-missing data at baseline and the corresponding visit. 95% Confidence Intervals were calculated based on the t-distribution of the log-transformed values or the difference in the log-transformed values for GMT and GMFR, respectively, then back transformed to the original scale for presentation.

Supplementary Table 8: Neutralizing Antibody Titers (Pseudovirus ID50 versus the Delta Variant) after the Primary Series and a Booster Injection of 50 µg of mRNA-1273 (Per-protocol Set)

	50 µg Prime N=146	100 µg Prime N=149	50 µg and 100 µg Prime N=295
OL-Day 1 (Pre-booster), n*	144	149	293
GMT	37.1	47.9	42.3
95% CI	31.3, 44.2	39.7, 57.8	37.2, 48.0
OL-Day 29 (28 Days after booster), n‡	146	149	295
GMT	779.5	827.8	803.5
95% CI	670.1, 906.8	738.5, 927.9	731.4, 882.7
Comparison of OL-D29 to OL-D1			
GM Fold Rise	20.9	17.3	19.0
95% CI	17.5, 24.9	14.4, 20.8	16.7, 21.5

Supplementary Table 8: Antibody values reported as below the lower limit of quantification (LLOQ; 18.5) were replaced by 0.5 x LLOQ. Values that were greater than the upper limit of quantification (ULOQ; 45118) were converted to the ULOQ if actual values were not available.
*n=Number of participants with non-missing results at pre-booster.
†95% Confidence Intervals were calculated based on the t-distribution of the log-transformed values or the difference in the log-transformed values for GMT and GMFR, respectively, then back transformed to the original scale.
‡ Number of participants in the Per-Protocol Set with non-missing data at the corresponding visit.

Supplementary Table 9: Summary of Geometric Means of PsVNA ID50 Titers and Ratios by Age Group

Age Group	≥18-<65 years		≥65 years	
	28 days post booster N=219	28 days post dose 2 in primary series N=218	28 days post booster N=76	28 days post dose 2 in primary series N=76
n*	214	214	75	75
GMT (95% CI)	1940.9 (1748.3, 2154.7)	985.5 (870.0, 1116.3)	1754.1 (1448.3, 2124.3)	684.3 (548.3, 854.0)
GMR (28 days post booster vs. 28 days post dose 2 in primary series) [95% CI]	2.0 [1.7, 2.2]		2.6 [2.1, 3.1]	
Age Group	≥18-<55 years		≥55 years	
	28 days post booster N=131	28 days post dose 2 in primary series N=131	28 days post booster N=164	28 days post dose 2 in primary series N=163
n	130	130	159	159
GMT (95% CI)	2025.6 (1789.8, 2295.4)	1090.9 (949.5, 1253.3)	1786.9 (1565.1, 2040.3)	763.6 (650.1, 896.9)
GMR (28 days post booster vs. 28 days post dose 2 in primary series) [95% CI]	1.9 [1.6, 2.2]		2.3 [2.0, 2.7]	

Supplementary Table 9: GMT=geometric mean titer; 95% CI=95% confidence interval; GMR=geometric mean titer ratio; Per-protocol Immunogenicity Set

*n = Number of participants in the Per-Protocol immunogenicity Set with non-missing data at 28 days after dose 2 in the primary series and 28 days post-booster.

Supplementary Table 10: Comparison of Pseudovirus Neutralization ID50 and ID80 Titers

	PsVNA ID50		PsVNA ID80	
	50 µg Prime N=146	100 µg Prime N=149	50 µg Prime N=146	100 µg Prime N=149
Pre-booster				
n*	145	149	145	149
GMT [95% CI]	104.7 [88.3, 124.1]	150.2 [125,7, 179.5]	39.6 [33.8, 46.5]	55.7 [47.5, 65.3]
28 Days After Booster				
n	146	149	146	149
GMT [95% CI]	1834.3 [1600.2, 2102.6]	1951.7 [1729.6, 2202.4]	696.1 [609.2, 795.4]	736.6 [661.0, 820.8]
GMFR [95% CI]	17.5 [14.9, 20.6]	13.0 [11.0, 15.3]	17.6 [15.1, 20.4]	13.2 [11.5, 15.3]

Supplementary Table 10: GMT=geometric mean titer; 95% CI=95% confidence interval; GMFR=geometric mean fold rise in titer; Per-protocol Immunogenicity Set

*n = Number of participants in the Per-Protocol immunogenicity Set with non-missing data at pre-booster and 28 days after the booster.

Supplementary Figure 1: Participant Flow Diagram

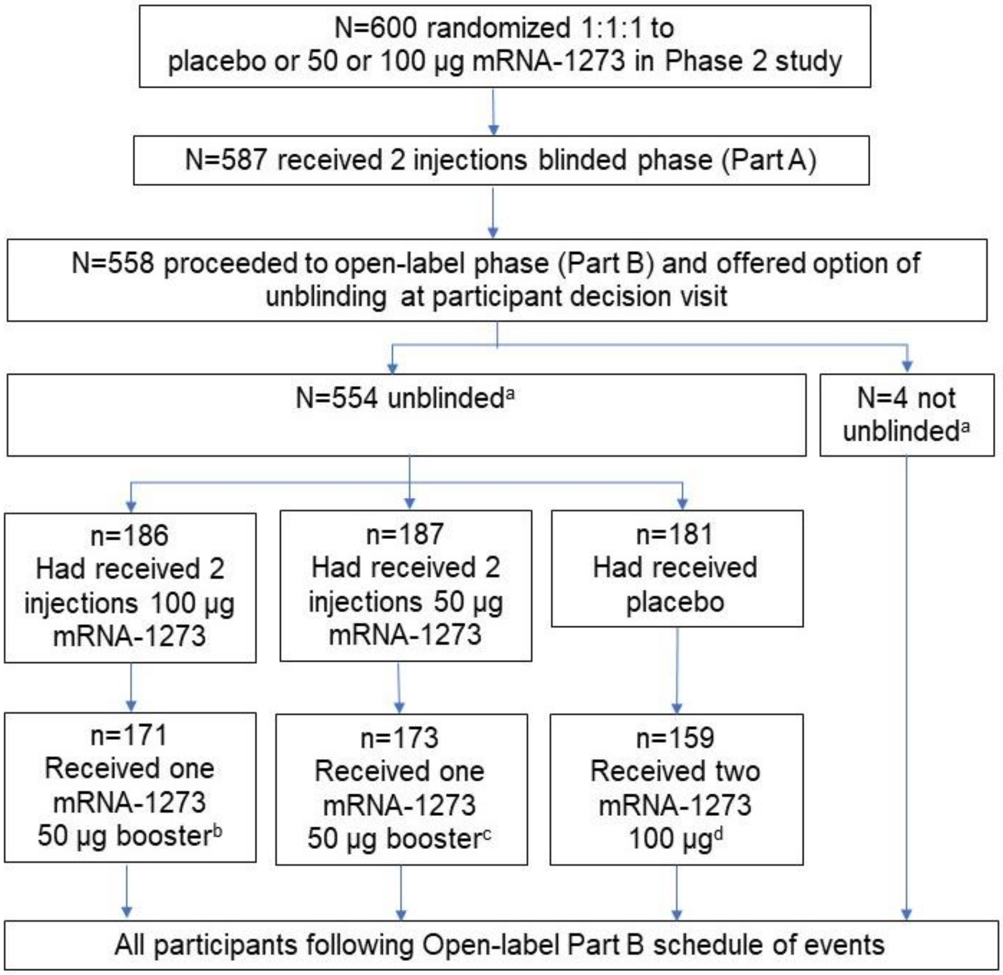

Supplementary Figure 1. In Part B, participants who had received 2 injections of 50 µg or 100 µg mRNA-1273 or placebo completed the blinded phase (Part A) and went on to receive a single open-label booster dose of 50 µg mRNA-1273 or two doses of 100 µg of mRNA-1273.

[a]Unblinded or not unblinded to assigned treatment in Part A blinded phase.

[b]15 participants declined to receive a booster of 50 µg mRNA-1273.

[c]14 participants declined to receive a booster of 50 µg mRNA-1273.

[d]22 participants declined to receive mRNA-1273 in Part B.

Supplementary Figure 2: Neutralizing Antibody Titers (Pseudovirus ID50; D614G) After the Primary Series and After a Booster Injection of 50 µg of mRNA-1273 (Per-protocol Set)

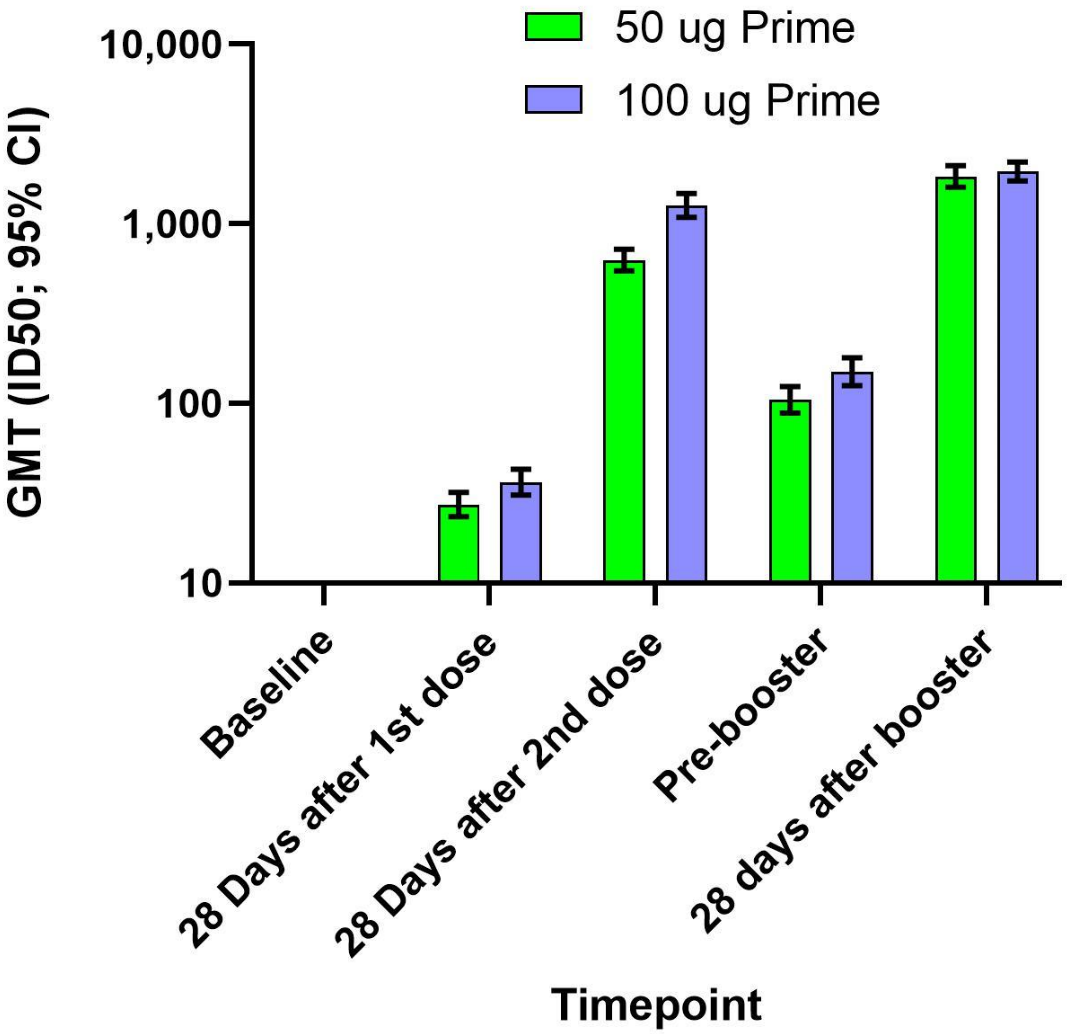

Supplementary Figure 2: The geometric mean titers (GMTs) for neutralizing antibodies against the D614G virus in the pseudovirus assay (tops of rectangular bars) and 95% confidence intervals (95% CIs; whiskers) are shown for serum samples collected in Part A at baseline, 28 days after the first dose of mRNA-1273, 28 days after the second dose of mRNA-1273, and in Part B before the booster injection of 50 µg of mRNA-1273 (Pre-booster) and 28 days after the booster injection. Results from the group that received two priming doses of 50 µg of mRNA-1273 are shown in green, and those from the group that received two priming doses of 100 µg of mRNA-1273 are shown in blue. Antibody values in the pseudovirus assay reported as below

the lower limit of quantification (LLOQ; 18.5) were replaced by 0.5 x LLOQ. Values that were greater than the upper limit of quantification (ULOQ; 45118) were changed to the ULOQ if actual values were not available. 95% confidence intervals were calculated based on the t-distribution of the log-transformed values or the difference in the log-transformed values for GMT, and then back transformed to the original scale.

Supplementary Figure 3: Spike-binding IgG Antibody Titers (VAC65 ID50; D614G) After the Primary Series and After a Booster Injection of 50 µg of mRNA-1273 (Per-protocol Set)

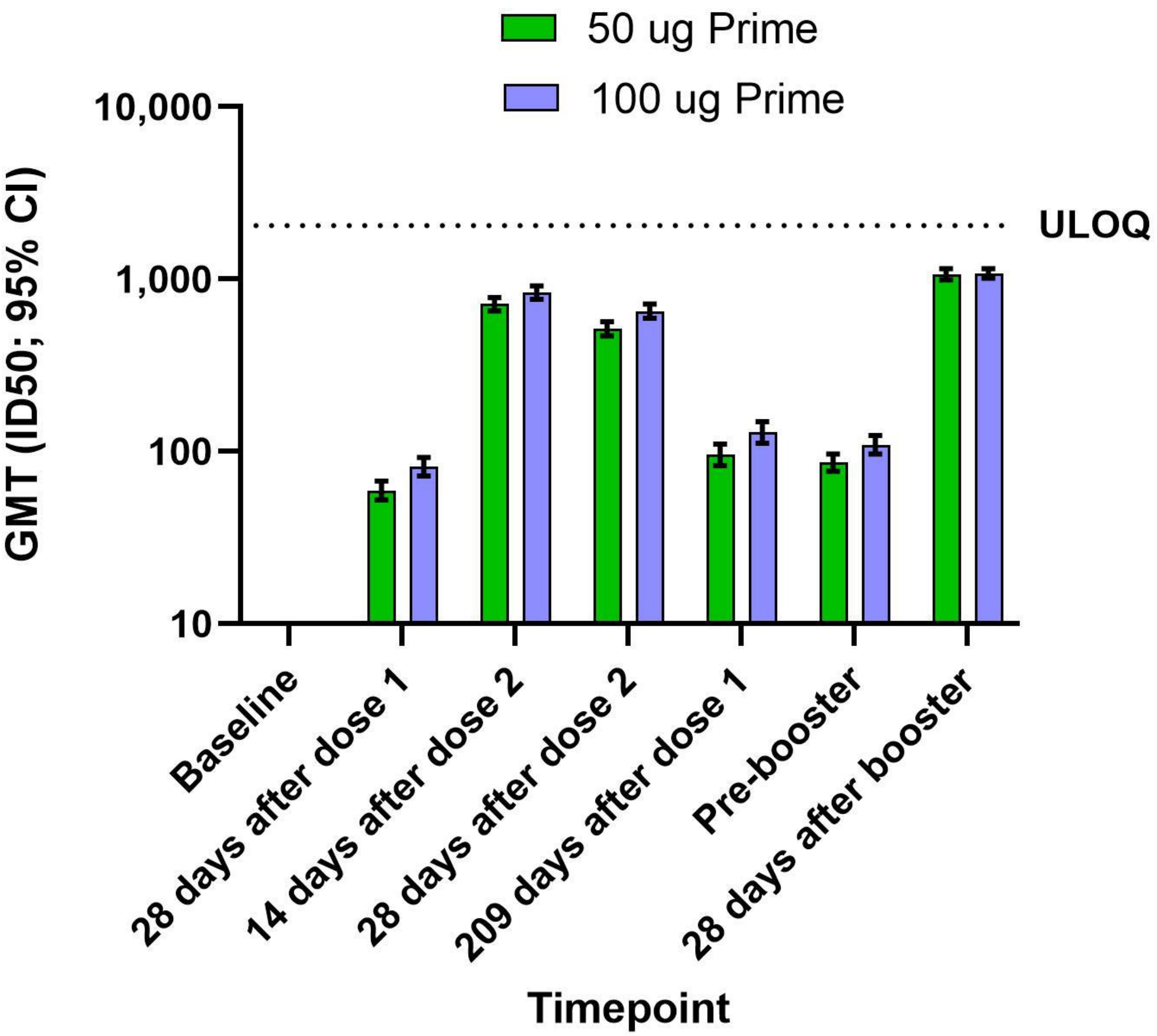

Supplementary Figure 3: The geometric mean titers (GMTs) and 95% confidence intervals (95% CIs) against the D614G virus in the spike-binding IgG antibody assay of serum samples collected in Part A at 28 days after the first dose of mRNA-1273, 14 days after the second dose of mRNA-1273, 28 days after the second dose of mRNA-1273, and in Part B before the booster injection of 50 µg of mRNA-1273 (Pre-booster) and 28 days after the booster injection. Results from the group that received two priming doses of 50 µg of mRNA-1273 are shown in green, and those from the group that received two priming doses of 100 µg of mRNA-1273 are shown in blue. Values reported as below the lower limit of quantification (LLOQ; 1) were replaced by 0.5 x LLOQ. Values that were greater than the upper limit of quantification (ULOQ; 2052) were

changed to the ULOQ if actual values were not available. 95% Confidence Intervals were calculated based on the t-distribution of the log-transformed values or the difference in the log-transformed values for GMT, then back transformed to the original scale.

Supplementary Figure 4: Box and Whisker Plot of Spike-binding IgG Antibody Titers (VAC65 ID50; D614G) After the Primary Series and After a Booster Injection of 50 µg of mRNA-1273 (Per-protocol Set)

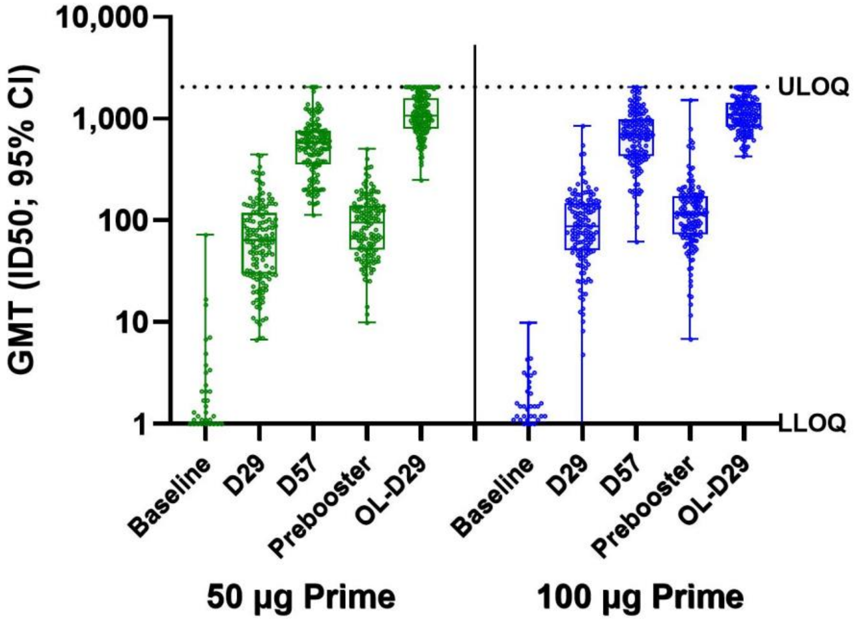

Supplementary Figure 4: The geometric mean titers (GMTs) and 95% confidence intervals (95% CIs) against the D614G virus in the spike-binding IgG antibody assay of serum samples collected in Part A at 28 days after the first dose of mRNA-1273, 14 days after the second dose of mRNA-1273, 28 days after the second dose of mRNA-1273, and in Part B before the booster injection of 50 µg of mRNA-1273 (Pre-booster) and 28 days after the booster injection. Values reported as below the lower limit of quantification (LLOQ; 1) were replaced by 0.5 x LLOQ. Values that were greater than the upper limit of quantification (ULOQ; 2052) were changed to the ULOQ if actual values were not available. 95% Confidence Intervals were calculated based on the t-distribution of the log-transformed values or the difference in the log-transformed values for GMT, then back transformed to the original scale.

Supplementary Figure 5: Neutralizing Antibody Titers (Pseudovirus ID50; D614G) Before and After a Booster Injection of 50 µg of mRNA-1273

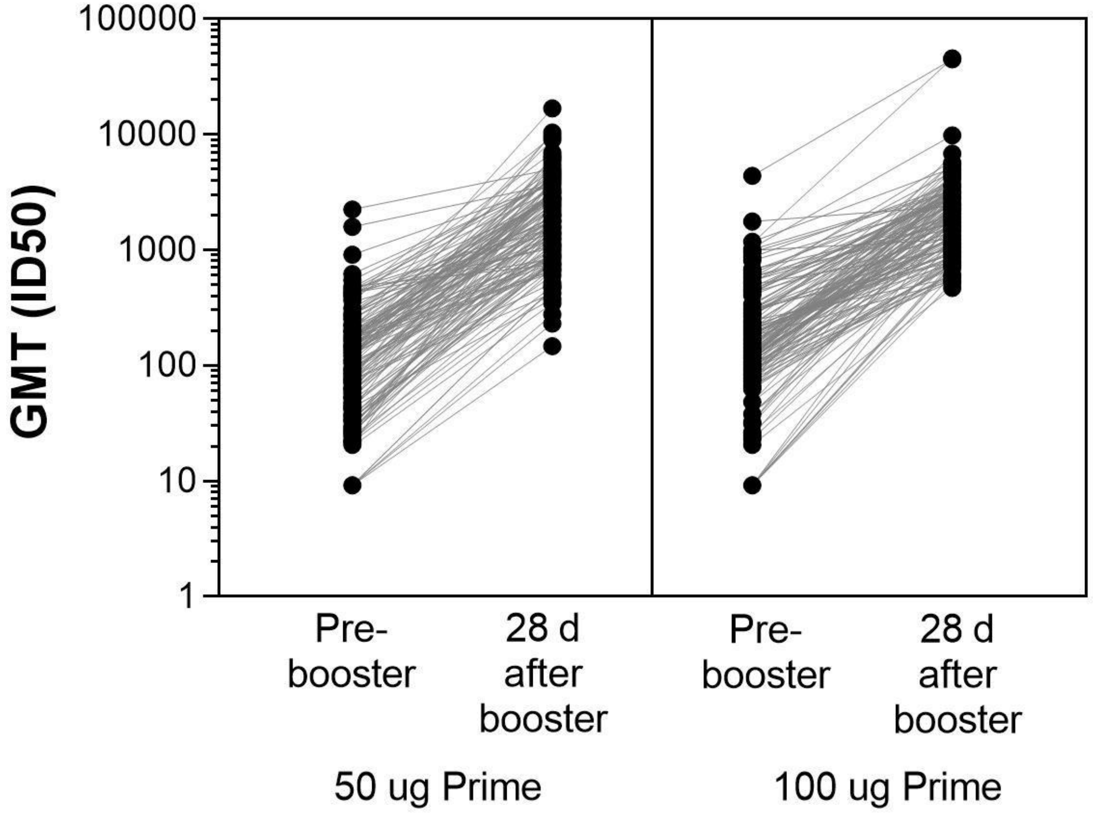

Supplementary Figure 5: GMT=geometric mean titer. The GMTs (ID50) for pseudovirus (D614G) neutralizing antibody titers pre-booster and 28 days after the booster for individual participants are shown by symbols connected by lines.

Protocol Amendment Summary of Changes

DOCUMENT HISTORY

Document	Date
Amendment 6	28 Apr 2021
Amendment 5	19 Feb 2021
Amendment 4	15 Jan 2021
Amendment 3	02 Sep 2020
Amendment 2	01 Jul 2020
Amendment 1	18 May 2020
Original Protocol	22 Apr 2020

Summary of Major Changes in Protocol Amendment 6:

Section # and Name	Description of Change	Brief Rationale
Synopsis and Section 4.7.6 (End of Study Analysis)	End of Part A clarified	To clarify that an analysis will be performed at the conclusion of Part A
Synopsis and Section 4.7.2 (Analysis at End of Blinded part A Only)	Added an analysis of safety and immunogenicity at the end of Part A	To clarify that an analysis of safety and immunogenicity will be performed on all participants upon completion of Part A ofthe study.

Amendment 5, 19 Feb 2021
Main Rationale for the Amendment:

There is an urgent need for vaccination strategies against SARS-CoV2 that induce broader protection that includes variants such as B.1.351 to decrease morbidity and mortality. ModernaTX, Inc. is developing a mRNA vaccine (mRNA-1273.351) that is similar to the mRNA-1273 vaccine available under the Emergency Use Authorization (EUA), but in which the mRNA encodes for mutations included in the S protein of the B.1.351 variant.

This protocol amendment will add Part C to the protocol, which will be an amendment to investigate the proof of concept of a single dose booster of two dose levels of the mRNA-1273.351 variant and a mixture formulation of mRNA-1273/mRNA-1273.351 administered to approximately 60 participants who received primary vaccination during the mRNA-1273-P301 COVE study. The COVE study participants will be offered enrollment in this new site-specific sub study, Part C of mRNA-1273-P201, based on pre-determined eligibility criteria. If they choose to enroll in this protocol amendment, the participants will be discontinued from the mRNA-1273-P301 COVE study. The participants would have had to be originally randomized to the mRNA-1273 group and have previously received 2 doses of mRNA-1273, 28 days apart, to be enrolled in this amendment. The unblinding visit should also have occurred. In this protocol amendment, enrolled participants will be allocated 1:1:1 to receive a single intramuscular injection of mRNA-1273.351 (20 µg or 50 µg) or mRNA-1273/mRNA-1273.351 mixture (50 µg) as a booster injection.

The summary of changes table provided here describes the major changes made in Amendment 5 relative to Amendment 4, including the sections modified and the corresponding rationales. The synopsis of Amendment 5 has been modified to correspond to changes in the body of the protocol.

Summary of Major Changes in Protocol Amendment 5:

Section # and Name	Description of Change	Brief Rationale
Title Page, Protocol Approval Page, Headers, Protocol Amendment Summary of Changes	Updated the protocol version and date.	To reflect the new version and date of the protocol.

Protocol Synopsis – Objectives and Section 2.7 (Primary Objectives [Part C, Open Label]), Section 2.8 (Secondary Immunogenicity Objective [Part C, Open Label]), and Section 2.9 (Exploratory Objectives [Part C, Open Label]	The description of the Part C primary, secondary, and exploratory objectives was added to the Protocol Synopsis. The description of Part C was included in Section 2 (Study Objectives). Section 2.7, Section 2.8, and Section 2.9 were added to encompass the addition of Part C primary, secondary, and exploratory objectives.	The addition of Part C was the main purpose of this amendment.
Protocol Synopsis – Study Design and Methodology and Section 3.1 (General Study Design)	A description of the general design of Part C was added to the synopsis. The description was also added to Section 3.1 for consistency.	The addition of Part C was the main purpose of this amendment. Language was included for consistency and clarity.
Protocol Synopsis – Study Design and Methodology and Section 3.1.3 (Part C, Open-Label Interventional Phase of mRNA-1273.351 and mRNA-1273/mRNA-1273.351 Mixture Booster Vaccines)	A further description of the methodology for Part C was included in the synopsis and Section 3.1.3 for consistency and clarity. Figure 4 was added to Section 3.1.3 as a visual aid for the structure of Part C.	The addition of Part C was the main purpose of this amendment. Language was included for consistency and clarity.
Protocol Synopsis – Safety Assessments and Section 3.4.7 (Safety Assessments)	Applicable safety assessments for Part C were indicated in the safety sections of the synopsis and body for consistency.	The addition of Part C was the main purpose of this amendment.
Protocol Synopsis – Immunogenicity assessments, Section 3.4.5 (Immunogenicity Assessments)	Part C immunogenicity assessments were included for clarity.	The addition of Part C was the main purpose of this amendment. Language added for clarity.
Protocol Synopsis – Investigational Product, Dosage, and Route of Administration and Section 3.3.3 (Identity of Investigational Product)	The investigational product, dosage, and route of administration information was provided for Part C.	The addition of Part C was the main purpose of this amendment. Language was included for clarity.
Protocol Synopsis – Investigational Product, Dosage, and Route of Administration and Section 3.3.5 (Blinding)	Specified that Part B and Part C of the study will be open label.	The addition of Part C was the main purpose of this amendment. Language was added for clarity and consistency.

Section # and Name	Description of Change	Brief Rationale
Protocol Synopsis – Sample Size and Section 4.5 (Sample Size Determination)	Clarified that patients in Part A will have the opportunity to enter Part B provided they meet the eligibility criteria.<br>A description of the Part C sample size and enrollment process was provided.	Language was updated for clarity. The description for Part C was included as the addition of Part C was the main purpose of this amendment.
Protocol Synopsis – Statistical Methods and Section 4.6 Statistical Methods.	Indicated that Part C data may be presented separately, as appropriate.<br>Specified when analyses were specific to Part A and Part B.<br>Indicated that Part C will be similarly analyzed (when not specific to Part A and Part B).	Language was included as the addition of Part C was the main purpose of this amendment. Other language updated for clarity and consistency.
Protocol Synopsis – Study Analyses and Section 4.7.3 (Interim Study Analysis for Open-Label Part C Only)	Indicated that an interim analysis of the safety and immunogenicity data in Part C of the study may be performed after participants have completed OL-D8, OL-D15, OL-Day 29 and/or the OL-Day 57 study procedures.<br>Section 4.7.3 added, and language included for consistency and clarity.	The addition of Part C was the main purpose of this amendment. Language was included for clarity.
Section 3.1.6 (Inclusion Criteria)	Section 3.1.6 heading was created.<br>Inclusion criterion #7 was updated to #6 as there is no inclusion criterion #7.<br>A separate set of inclusion criteria for Part C was included. Headers separating Parts A and B from Part C were created.	Headings were created for clarity and readability.<br><br>Numbering was updated for clarity and accuracy.<br><br>Part C inclusion criteria was added to clarify what participants will be eligible for Part C.
Section 3.1.7 (Exclusion Criteria)	A separate set of exclusion criteria for Part C was included. Headers separating Parts A and B from Part C were created.	Part C exclusion criteria was added to clarify which participants will not be eligible for Part C. Headers were created for clarity and readability.
Section 3.3.1 (Method of Assigning Participants to Dosing Groups [Part A and Part C])	Table 2 – Dose Group Assignment (Part C) added to clarify groups, investigational product, and number of participants.	The addition of Part C was the main purpose of this amendment. The table was added for clarity.

Section # and Name	Description of Change	Brief Rationale
Section 3.3.2 (Investigational Product Administration)	Indicated that the Part C investigational product will be administered as a single intramuscular injection into the deltoid muscle on the day of the participants consent. Each injection will have variable dose volumes; the 20-µg dose of mRNA-1273.351 is 0.2 mL, the 50-µg dose of mRNA-1273.351 is 0.5 mL, and the 50-µg dose of pharmacy prepared mixture of mRNA-1273/mRNA-1273.351 is 0.5 mL.	The addition of Part C was the main purpose for this amendment. This language was included for clarity.
Section 3.3.2.1 (Pause Rules)	Indicated that the pause rules described in this section are not applicable to Part C. Clarified for Part C, that the sponsor will continue to inform the SMC of any occurrence of the events in Table 3.	The addition of Part C was the main purpose for this amendment. This language was included for clarity.
Section 3.3.2.2 (Contraindications to Subsequent Injection)	Updated how long the patient will be encouraged to continue study participation from 12 months to 6 months.	Existing safety data across mRNA-1273 studies suggest that a safety follow up of 6 months after last vaccination is acceptable.
Section 3.3.4.2 (Packaging and Labeling)	Additional packaging and labeling information for Part C was included.	The addition of Part C was the main purpose of this amendment.
Section 3.3.4.3 (Storage)	Confirmed that the mRNA-1273 vaccine in Part C must be stored at -25°C to -15°C. The mRNA-1273.351 vaccine used in Part C must be stored at -90°C to -60°C (-130°F to -76°F).	The addition of Part C was the main purpose of this amendment and other additional language was for clarity.
Section 3.8.5.6 (Definition and Reporting of Adverse Events Consistent With Anaphylaxis)	Section 3.8.5.6 describes the definitions and reporting procedures for Adverse Events (AEs) consistent with anaphylaxis.	This text is being added to all mRNA-1273 protocols based on recent reports of anaphylaxis in the post-Emergency Use Authorization setting.
Section 4.3.3 (Part C, Open Label)	Updated Section 4.3 to include Part C primary safety endpoints, primary immunogenicity endpoints, secondary immunogenicity endpoint, and exploratory endpoints.	The addition of Part C was the main purpose of this amendment.

Section # and Name	Description of Change	Brief Rationale
Section 7.1 (Appendix 1: Schedule of Events) – Table 11 (Part B: Open-label Schedule of Events: ONLY for Participants Receiving 2 Doses of mRNA-1273 28 Days Apart)	"Blood for vaccine immunogenicity[4]" updated to "Blood for serology[9] and immunogenicity[4]"<br>Added Footnote 9 "Blood sample for serology will be collected only at the Participant Decision Visit."	Language updated and footnote added for clarity and specificity of serology timing.

Section # and Name	Description of Change	Brief Rationale
Section 7.1 (Appendix 1: Schedule of Events) – Table 12 (Part B and Part C: Open-label Schedule of Events (Participants Receiving a Booster Dose)[9]	Table title updated to remove “ONLY for” and “single.”<br>“Blood for vaccine immunogenicity[5]” updated to “Blood for serology[10] and immunogenecity[5]”<br>Updated note language to indicate the following, “Participants who decline receiving booster mRNA-1273, decline unblinding, or decline to receive 2 doses of mRNA-1273 in Part B will not receive study vaccination at OL-D1 (or OL-D29), will not perform a pregnancy test, or eDiary activation at OL-D1.”<br>Added Footnote 10 “Blood sample for serology will be collected only at the Participant Decision Visit (OL-D1).”<br>Addition of Booster injection for Part C under OL-D57.<br>Added footnote 11 “In Part C, an additional booster injection may be added approximately 56 days after the first boost at OL-D1. This additional booster dose will be triggered following review of immunogenicity data up to OL-D15 of the initial mRNA1273.351(20 and 50 µg) and mRNA-1273/mRNA-1273.351 mixture injections.”<br>Added footnote 12 “If the additional booster dose is given at OL-D57, an eDiary for reactogenicity will be completed for 7 days post-injection. Additionally, unsolicited AEs will continue to be collected for 28 days post last injection.”	Title update made as this schedule of events applies to participants who choose to decline unblinding, but wish to remain in the study, decide to unblind but decline to receive further vaccination (either a single mRNA-1273 booster or 2 doses of mRNA-1273, 28 days apart). Language updates made for clarity and specificity of serology timing.<br>An additional booster injection may be added approximately 56 days after the first boost at OL-D1. This will be determined following review of immunogenicity data up to OL-D15.<br>Additional footnotes included for clarity.

Amendment 4, 15 Jan 2021

Main Rationale for the Amendment:

Following authorization of a COVID-19 vaccine under an Emergency Use Authorization (EUA), this study amendment is designed to transition to Part B, the Open-Label Interventional Phase (Figure 3). Transitioning the study to Part B, Open-Label Interventional Phase permits all ongoing study participants to (a) be informed of the availability and eligibility criteria of any COVID-19 vaccine made available under an EUA, and (b) the option to offer all ongoing study participants who request unblinding an opportunity to schedule a study visit to know their original group assignment (placebo vs. mRNA-1273 [50µg or 100µg vaccine]).

Part B, Open-label Interventional Phase, also provides the opportunity for study participants who previously received placebo, to request to receive 2 doses of the mRNA-1273 (100 µg) vaccine. Participants who originally received 1 or 2 doses of mRNA-1273 (50µg or 100µg vaccine) during Part A, will have the opportunity to request to receive a single booster dose of mRNA-1273 (50 µg).

The summary of changes table provided here describes the major changes made in Amendment 4 relative to Amendment 3, including the sections modified and the corresponding rationales. The synopsis of Amendment 4 has been modified to correspond to changes in the body of the protocol.

Summary of Major Changes in Protocol Amendment 4:

Section # and Name	Description of Change	Brief Rationale
Title Page, Protocol Approval Page, Headers, Protocol Amendment Summary of Changes	Updated the protocol version and date	To reflect the new version and date of the protocol
Synopsis (Objectives) and Section 2 (Study Objectives)	Added objectives for Part B, Open Label	Added to specifically enumerate the objectives for the Open-Label part of the study, allowing for changes to study design and dosing.

Section # and Name	Description of Change	Brief Rationale
Synopsis (Study Design), Section 3.1.2 (Part B, Open-Label Interventional Phase), Section 7.1 (Appendix 1: Schedule of Events)	Added a “Participant Decision Clinic Visit”; instructions for transitioning to Part B, Open Label; Schedule of Events for the Participant Decision Clinic Visit; and Schedules of Events for Part B, Open-Label procedures.	This Participant Decision Clinic Visit provides the opportunity for study site personnel to discuss with and offer to participants, the choice to be unblinded, as well as offering to participants who originally received placebo, the choice to receive active vaccination with mRNA-1273 and possible vaccination against COVID-19, as well as offering those who received mRNA-1273 during Part A, the choice to receive a booster injection of mRNA-1273.<br>The new Schedules of Events distinguish Part B, Open Label from Part A, Blinded, since all participants will transition to Part B. The Part B Schedules of Events provide procedural instructions for participants who will receive 2 mRNA-1273 injections and for those who will receive 1 mRNA-1273 injection in Part B.
Section 1.2 (Nonclinical Studies in Development of mRNA-1273) and Section 1.3 (Clinical Studies With Lipid Nanoparticle mRNA Vaccines)	Updated status of nonclinical studies, as well as ongoing clinical studies, including this study (mRNA-1273-P201) and the Phase 3 Study mRNA-1273-P301.	The status of the 3 clinical studies (one Phase 1, one Phase 2a, and the Phase 3 study) has changed since Amendment 3.<br>In addition, the results of the interim analyses in the Phase 3 study of the primary efficacy endpoint (prevention of COVID-19 infection), a major secondary endpoint (prevention of severe COVID-19), and safety and reactogenicity endpoints are now available and are provided here. These results provide the justification for offering participants the opportunity to receive active investigational product (mRNA-1273) and the potential benefit of vaccination against COVID-19.

Section # and Name	Description of Change	Brief Rationale
Section 4.3.2 (Statistical Analyses, Part B, Open Label)	Addition of Part B, open-label statistical analyses	Lists the Part B, open-label endpoints. Major differences from the Part A endpoints/analyses were elimination of assessment of laboratory values, and distinguishing identical endpoints for 50 µg and 100 µg of mRNA-1273.
Section 3.4.5 (Blinding)	The study site staff, investigators, study monitors, and participants will remain blinded until the conclusion of the study.<br>Changed to: The study site staff, investigators, study monitors, and participants will remain blinded until the initiation of Open-Label Part B.	Previous language is no longer applicable, since the study will be unblinded for Part B.
Section 3.4.2.1 (Pause Rules)	The pause-triggering rules that have been in effect for Part A, will not be applicable for Part B; however, participants will continue to be monitored for the pause rule criteria.	In December 2020, after review of both safety and efficacy data observed to date, the FDA granted Emergency Use Authorization (EUA) to Moderna's mRNA-1273 vaccine. Given this, the current pause rules as outlined cannot be applied effectively, as it is more likely that any serious safety signal would emerge from the ongoing large public vaccination campaigns. Moderna will still monitor for safety events in this study and will report to the Safety Monitoring Committee as appropriate.
Section 4.7.2 (Interim Analysis for Open-Label Part B Only)	Added an option IA following completion of OL-Day 29 and/or OL-Day 57 study procedures.	The IA would help inform of the benefit of participant receiving a booster dose.

Amendment 3, 02 Sep 2020

The main purpose of this amendment was to clarify that data can be analyzed in multiple batches based on availability of participants who have reached the Day 57 visit. The summary of changes table describes the major changes made in Amendment 3 relative to Amendment 2, including the sections modified and the corresponding rationales. Minor editorial or formatting changes are not included in this summary table.

Summary of Major Changes in Protocol Amendment 3:

Section # and Name	Description of Change	Brief Rationale
Title page, Signature page, Synopsis, and header	Updated the protocol version and date	Reflect the new version and date of the protocol.
Synopsis, Section 3.1 Study Design	Deleted repeated text about Safety Monitoring Committee review before expansion in Cohort 2.	Editorial removal of redundant text.
Synopsis,<br>Section 3.4.5 Blinding,<br>Section 4.1 Blinding and Responsibility for Analyses,<br>Section 4.7.1 Primary Study Analysis	Added information about potential participant populations to be included in the primary analysis of safety and immunogenicity after completion of Day 57 procedures.	Clarification that data can be analyzed in multiple batches based on availability of participants who have reached the Day 57 visit.
Synopsis, Section 4.6.2 Safety Analyses	Revisions to clarify that separate summaries of Grade 3 or higher solicited ARs are not planned.	Clarification of planned safety analyses.
Section 3.5.2 Use of Electronic Diaries,<br>Section 7.1 Appendix 1: Schedule of Events (Table 7)	Added clarification about site follow-up of relevant safety events from eDiary entries (includes revisions to Footnote 12).	Clarification that follow-up by telephone of relevant safety events from eDiary entries is not the same as scheduled safety follow-up telephone calls.
Section 7.1 Appendix 1: Schedule of Events (Table 7)	The acceptable window around the Day 29 visit has been clarified as + 7 days with no negative visit window.	Correction to reflect the minimum interval between vaccine administrations of 28 days.
Section 7.1 Appendix 1: Schedule of Events (Table 7)	The footnotes and footnote numbering have been updated to accommodate the footnotes that were added with Amendment 2.	Editorial clarification.
Section 7.1 Appendix 1: Schedule of Events (Table 7)	Footnote 4 has been revised to clarify that study days for safety follow-up are relative to Day 1 vaccine administration.	Editorial clarification.
Section 7.1 Appendix 1: Schedule of Events (Table 7)	Footnote 5 has been revised to explain how to handle potential visit window overlap related to Visit 8.	Editorial clarification.
Section 7.1 Appendix 1: Schedule of Events (Table 7)	Footnote 10 has been revised to clarify the timing of nasopharyngeal swab samples on vaccination days.	Editorial clarification.

Abbreviations: AE = adverse event; AR = adverse reaction; MAAE = medically attended adverse event; SAE = serious adverse event.

Amendment 2, 01 Jul 2020

Main Rationale for the Amendment:

The main purpose of this amendment was to change the statistical analysis plan by removing interim analyses and defining the Primary Study Analysis and EOS Analysis. The summary of changes table describes the major changes made in Amendment 2 relative to Amendment 1, including the sections modified and the corresponding rationales. Minor editorial or formatting changes are not included in this summary table.

Summary of Major Changes in Protocol Amendment 2:

Section # and Name	Description of Change	Brief Rationale
Title page, Signature page, and header	Updated the protocol version and date	Reflect the new version and date of the protocol.
Synopsis, Section 3.1 Study Design, Section 3.5.2 Use of Electronic Diaries, Section 3.5.3 Safety Telephone Calls, Section 7.1 Appendix 1: Schedule of Events (including text, Table 7, and footnotes to Table 7)	Added eDiary questionnaires to the procedure for safety follow-up after the Day 57 visit, with completion of eDiary questionnaires alternating with safety telephone calls approximately every 2 weeks after the Day 57 visit.	Reduce the burden on study site personnel of completing safety follow-up by telephone.
Synopsis, Section 3.1 Study Design, Section 3.5.3 Safety Telephone Calls, Section 7.1 Appendix 1: Schedule of Events (footnote 12)	Added exposure to someone with known COVID-19 or SARS-CoV-2 infection and participant experience of COVID-19 symptoms to the list of events queried during scheduled safety telephone calls.	Improve surveillance for incidence of COVID-19 during the study.
Synopsis, Section 3.1 Study Design	End of Study definition was amended.	Minor clarification to define the End of Study.
Synopsis, Section 3.4.5 Blinding, Section 4.1 Blinding and Responsibility for Analyses, Section 4.7 Study Analyses, Section 4.7.1 Primary Study Analysis, Section 4.7.2 End of Study Analysis, Section 6.4 Clinical Study Reports	Added descriptions of the Primary Study Analysis and End of Study Analysis and respective clinical study reports, replacing descriptions of interim analyses and reports. The synopsis contains a new section.	Eliminate interim analyses in favor of a focus on the primary analysis.
Synopsis, Section 4.6 Statistical Methods	Stated that all analyses will be performed by treatment group overall (for the 2 cohorts combined) and for the 2 cohorts separately, unless specified otherwise.	Previous versions of the protocol had not included the overall analysis in statement of the standard scope of analysis.
Synopsis, Section 4.6.3 Immunogenicity Analyses	For the primary immunogenicity endpoint, geometric mean titer was changed to geometric mean.	Assays for bAb are under development. The reported values may or may not be titers; hence the protocol wording has been modified.

Section # and Name	Description of Change	Brief Rationale
Section 3.5.1 Assessment for SARS-CoV-2 Infection	Added instructions for asymptomatic patients who have a confirmed SARS-CoV-2 infection.	To clarify the steps for the investigator to follow when a participant is confirmed to have SARS-CoV-2 infection but is asymptomatic.
Section 3.5.8.8 Assessment of Severity	Decoupled life-threatening and Grade 4 in the severity assessment.	To adhere to CDISC guidance and align with case report form page.
Section 3.5.8.8 Assessment of Severity	Added clarification on when an AE is defined as serious.	To clarify when an AE is defined as serious.

Abbreviations: AE = adverse event; bAb = binding antibody; CDISC = Clinical Data Interchange Standards Consortium; eDiary = electronic diary; SARS-Cov-2 = Severe Acute Respiratory Syndrome coronavirus that causes COVID-19.

Amendment 1, 18 May 2020

Main Rationale for the Amendment:

The main purpose of this amendment was to incorporate the following modifications requested by the FDA Center for Biologics Evaluation and Research:

• Enhance monitoring of participants who are confirmed to have SARS-CoV-2 infection.

• Include a convalescent visit for participants with confirmed SARS-CoV-2 infection.

• Explore the mRNA-1273 vaccine efficacy in preventing asymptomatic SARS-CoV-2 infection.

• Update the Month 7 and Month 13 visits to Day 209 and Day 394, respectively, to extend the follow-up to a full 12-month period after the second injection on Day 29 (Month 1).

• Decrease the highest dose of mRNA-1273 in the study from 250 µg to 100 µg.

The summary of changes table describes the major changes made in Amendment 1, including the sections modified and the corresponding rationale. Minor editorial or formatting changes are not included in this summary table.

Summary of Major Changes in Protocol Amendment 1:

Section # and Name	Description of Change	Brief Rationale
Title page, Signature page, and header	Updated protocol version and date.	Revised version and date of protocol.
Title page, Signature page, and header	Updated the protocol title.	Revised to reflect the current purpose of the study.
Synopsis and Section 2.3 Exploratory Objectives	Added an exploratory objective to evaluate the effect of the mRNA-1273 vaccine on the incidence of SARS-CoV-2 infection.	Request from the Health Authority.

Section # and Name	Description of Change	Brief Rationale
Synopsis,<br>Section 2.3 Exploratory Objectives, Section 4.3.3 Exploratory Endpoints,<br>Section 4.6.4 Exploratory Analyses	Revised wording for the exploratory objective/endpoint regarding spike protein-specific serum immunoglobulin class and subclass and neutralizing antibody in serum	Editorial change.
Synopsis, Section 3.1 Study Design, Study Flow Schema (Figure 1), Sentinel and Expansion Cohort Schema (Figure 2),<br>Section 3.1.1 Rationale for Dose Selection, 3.4.1 Method of Assigning Participants to Dosing Groups. Dose Group Assignment (Table 1),<br>3.4.2 Investigational Product Administration, 4.5 Sample Size Determination	Decreased the highest dose of mRNA-1273 in the study from 250 µg to 100 µg.	Decreased based on the preliminary findings of the Phase 1 DMID study.
Synopsis and<br>Section 3.1 Study Design	Deleted collection of nasopharyngeal swab samples at the Screening Visit (Day 0).	Editorial update for consistency with Schedule of Events (Table 7).
Synopsis and<br>Section 3.1 Study Design	Deleted the number of visits at which participants will have blood samples collected.	Editorial update to avoid confusion as blood samples will be collected at different visits for safety and vaccine immunogenicity assessments.
Synopsis; Section 3.1 Study Design, Section 3.5.6 Blood Sampling Volumes (Table 3), Section 3.5.7 Safety Assessments,<br>Section 3.5.8.6 Eliciting and Documenting Adverse Events,<br>Section 4.3.1.2 Primary Immunogenicity Endpoint,<br>Section 4.3.2 Secondary Endpoints,<br>Section 4.7 Interim Analyses,<br>Section 6.4 Clinical Study Reports, and<br>Section 7.1 Appendix 1: Schedule of Events (Table 7)	Updated Month 7 and Month 13 visits to Day 209 and Day 394, respectively, to allow for 6-month and 12-month intervals, respectively, after the second injection on Day 29 (Month 1).	Request from the Health Authority.
Synopsis, Section 3.1 Study Design, and<br>Section 7.1 Appendix 1: Schedule of Events (Table 7)	Updated the biweekly safety telephone calls from Day 211 through Day 351 to Day 223 through Day 377.	Consequent to the change made to the Day 209 Visit (Request from the Health Authority).

Section # and Name	Description of Change	Brief Rationale
Synopsis, Section 3.1 Study Design,<br>Section 3.1.2 Rationale for Study Design,<br>Section 3.5.1 Assessment for SARS-CoV-2 Infection, and<br>Section 7.1 Appendix 1: Schedule of Events (Table 7)	Updated nasal swab to nasopharyngeal swab.	Clarified the type of swab to be performed.
Section 3.1.1 Rationale for Dose Selection	Updated enrollment and preliminary safety data from the ongoing Phase 1 DMID study.	Updated based on the preliminary findings of the Phase 1 DMID study.
Section 3.2.1 Inclusion Criteria	Updated inclusion criterion #7 to exclude sperm donations through 3 months after the last injection.	Update to align with the informed consent form on refraining male participants from sperm donation through 3 months after the last injection based on IRB feedback to the ICF.
Section 3.3.2 Handling Withdrawal From the Study	Updated the scheduled end of study assessments at Day 394 (Month 13) to allow for a 12-month interval after the second vaccination on Day 29 (Month 1).	Request from the Health Authority.
Section 3.4.5 Blinding	Updated the method to maintain the blind of the dosing assignment from opaque sleeve to blinding label.	Operational change in cases for which opaque sleeves are not used.
Section 3.5.1 Assessment for SARS-CoV-2 Infection	• Added more intense monitoring of participants who are confirmed to have SARS-CoV-2 infection (ie, notification of the participant's primary care physician by the investigator and recording of confirmed SARS-CoV-2 infection as an MAAE along with relevant concomitant medications and details about severity, seriousness, and outcome).<br>• Added a convalescent visit with blood collection after diagnosis of SARS-CoV-2 infection.	Request from the Health Authority.
Section 3.5.1 Assessment for SARS-CoV-2 Infection and Section 3.5.8.2 Medically Attended Adverse Event	Deleted "or COVID-19."	Editorial update for internal consistency.
Section 4.3.3 Exploratory Endpoints	Included a new exploratory endpoint to evaluate the effect of the mRNA-1273 vaccine on the incidence of SARS-CoV-2 infection.	Request from the Health Authority.

Section # and Name	Description of Change	Brief Rationale
Section 7.1 Appendix 1: Schedule of Events (Table 7)	Deleted that Day 0 and Day 1 visits may be combined the same day.	Editorial update of template text, which did not apply to this protocol.
	Corrected sequential footnote numbering in the schedule of events (Table 7).	Editorial update.
	Included a header row titled "Days Since Most Recent Vaccination."	Update to clarify that the visits are relative to the most recent injection.

Abbreviation: DMID = Division of Microbiology and Infectious Diseases; ICF = informed consent form; IRB = Institutional Review Board; MAAE = medically attended adverse event; SARS-Cov-2 = Severe Acute Respiratory Syndrome coronavirus that causes COVID-19.

REFERENCES

1 Chu, L. *et al.* A preliminary report of a randomized controlled phase 2 trial of the safety and immunogenicity of mRNA-1273 SARS-CoV-2 vaccine. *Vaccine* **39**, 2791-2799, doi:10.1016/j.vaccine.2021.02.007 (2021).

2 Baden, L. R. *et al.* Efficacy and Safety of the mRNA-1273 SARS-CoV-2 Vaccine. *N Engl J Med* **384**, 403-416, doi:10.1056/NEJMoa2035389 (2021).

3 Jackson, L. A. *et al.* An mRNA Vaccine against SARS-CoV-2 - Preliminary Report. *N Engl J Med* **383**, 1920-1931, doi:10.1056/NEJMoa2022483 (2020).

4 Gilbert, P. B. *et al.* Immune correlates analysis of the mRNA-1273 COVID-19 vaccine efficacy clinical trial. *Science*, https://www.science.org/doi/10.1126/science.abm3425 (2021).

5 Shen, X. *et al.* SARS-CoV-2 variant B.1.1.7 is susceptible to neutralizing antibodies elicited by ancestral spike vaccines. *Cell Host Microbe* **29**, 529-539 e523, doi:10.1016/j.chom.2021.03.002 (2021).

6 FDA. *Bioanalytical Method Validation: Guidance for Industry*, <https://www.fda.gov/files/drugs/published/Bioanalytical-Method-Validation-Guidance-for-Industry.pdf> (2018).
