# Supplementary material for: Immune response to SARS-CoV-2 after a booster of mRNA-1273: an open-label phase 2 trial
Source: Nat Med. 2022 Mar 3;28(5):1042–9. doi: 10.1038/s41591-022-01739-w (PMC9117133; doi:10.1038/s41591-022-01739-w)
Supplement: Supplementary file 2 — Reporting Summary [file 41591_2022_1739_MOESM2_ESM.pdf]

Corresponding author(s): Roderick McPhee, M.D., Ph.D.

Last updated by author(s): Feb. 3, 2022

Reporting Summary

Nature Portfolio wishes to improve the reproducibility of the work that we publish. This form provides structure for consistency and transparency in reporting. For further information on Nature Portfolio policies, see our Editorial Policies and the Editorial Policy Checklist.

Statistics

For all statistical analyses, confirm that the following items are present in the figure legend, table legend, main text, or Methods section.

n/a	Confirmed	
☐	☒	The exact sample size (*n*) for each experimental group/condition, given as a discrete number and unit of measurement
☐	☒	A statement on whether measurements were taken from distinct samples or whether the same sample was measured repeatedly
☐	☒	The statistical test(s) used AND whether they are one- or two-sided<br>*Only common tests should be described solely by name; describe more complex techniques in the Methods section.*
☒	☐	A description of all covariates tested
☒	☐	A description of any assumptions or corrections, such as tests of normality and adjustment for multiple comparisons
☐	☒	A full description of the statistical parameters including central tendency (e.g. means) or other basic estimates (e.g. regression coefficient) AND variation (e.g. standard deviation) or associated estimates of uncertainty (e.g. confidence intervals)
☐	☒	For null hypothesis testing, the test statistic (e.g. F, t, r) with confidence intervals, effect sizes, degrees of freedom and P value noted<br>*Give P values as exact values whenever suitable.*
☒	☐	For Bayesian analysis, information on the choice of priors and Markov chain Monte Carlo settings
☒	☐	For hierarchical and complex designs, identification of the appropriate level for tests and full reporting of outcomes
☒	☐	Estimates of effect sizes (e.g. Cohen's *d*, Pearson's *r*), indicating how they were calculated

Our web collection on statistics for biologists contains articles on many of the points above.

Software and code

Policy information about availability of computer code

Data collection	*Provide a description of all commercial, open source and custom code used to collect the data in this study, specifying the version used OR state that no software was used.*
Data analysis	All analyses were conducted using SAS Version 9.4 or higher. Prism (GraphPad Software, Inc.) was used to develop figures.

For manuscripts utilizing custom algorithms or software that are central to the research but not yet described in published literature, software must be made available to editors and reviewers. We strongly encourage code deposition in a community repository (e.g. GitHub). See the Nature Portfolio guidelines for submitting code & software for further information.

Data

Policy information about availability of data

All manuscripts must include a data availability statement. This statement should provide the following information, where applicable:

- Accession codes, unique identifiers, or web links for publicly available datasets
- A description of any restrictions on data availability
- For clinical datasets or third party data, please ensure that the statement adheres to our policy

As the trial is ongoing, access to patient-level data presented in this article (antibody assays, safety and reactogenicity) and supporting clinical documents with external researchers who provide methodologically-sound scientific proposals will be available upon request and subject to review once the trial is complete. Such requests can be made to Moderna Inc., 200 Technology Square, Cambridge, MA 02139. A materials transfer and/or data access agreement with the sponsor will be required for accessing shared data. All other relevant data are presented in the paper. The protocol is available as online supplementary material to this article. Clintrials.gov. NCT04405076.

Field-specific reporting

Please select the one below that is the best fit for your research. If you are not sure, read the appropriate sections before making your selection.

☒ Life sciences ☐ Behavioural & social sciences ☐ Ecological, evolutionary & environmental sciences

For a reference copy of the document with all sections, see nature.com/documents/nr-reporting-summary-flat.pdf

Life sciences study design

All studies must disclose on these points even when the disclosure is negative.

Sample size	There was no hypothesis testing in this study. With respect to sample size, the number of proposed participants was considered sufficient to provide a descriptive summary of the safety and immunogenicity of different doise levels of mRNA-1273 in the primary series.
Data exclusions	No data was excluded from analyses.
Replication	Antibody assays were run in triplicate.
Randomization	During Part A, the blinded part of the study, within each age cohort, approximately 300 participants were randomly assigned in 1:1:1 ratio to receive mRNA-1273 50 ug, mRNA-1273 100 ug, or placebo. The randomization was blinded using a centralized Interactive Response Technology (IRT), in accordance with pregenerated randomization schedules.
Blinding	Part B of the study was open label. In Part B, participants who received placebo in Part A of the study had the option to receive 2 injections of open-label mRNA-1273. Participants who received 1 or 2 doses of 50 ug or 100 ug mRNA-1273 in Part A were offered a single booster dose of mRNA-1273 (50 ug) in Part B.Vaccine dose preparation and administration during Part A were performed by unblinded pharmacy personnel who did not participate in any other aspects of the study. A limited number of the sponsor team and clinical research organization (CRO) were unblinded to enable the primary analysis at one month after the second dose of mRNA-1273 in Part A. All study staff, participants, CRO and sponsor personnel remained blinded to dosing assignment until the study was unblinded, upon implementation of Part B of the study, following Emergency Use Authorization of mRNAS-1273 in the United States.<br>Part A was observer blind. The investigators, study staff, study participants, site monitors and study personnel (or their designees) were blinded to the investigational product administered until study end or initiation of Part B, with the following exceptions: unblinded pharmacy personnel, unblinded site monitors, and a limited number of unblinded sponsor and clinical reseacrh organization personnel who performed the primary study analysis and prepared the Clinical Study Report. Part B of the study was open label.

Reporting for specific materials, systems and methods

We require information from authors about some types of materials, experimental systems and methods used in many studies. Here, indicate whether each material, system or method listed is relevant to your study. If you are not sure if a list item applies to your research, read the appropriate section before selecting a response.

Materials & experimental systems

n/a	Involved in the study	
☐	☒	Antibodies
☐	☒	Eukaryotic cell lines
☒	☐	Palaeontology and archaeology
☒	☐	Animals and other organisms
☐	☒	Human research participants
☐	☒	Clinical data
☒	☐	Dual use research of concern

Methods

n/a	Involved in the study	
☒	☐	ChIP-seq
☒	☐	Flow cytometry
☒	☐	MRI-based neuroimaging

Antibodies

Antibodies used	MSD® SULFO-TAG Labeled Anti-Human Antibody from Meso Scale Discovery, Gaithersburg, MD 20877, USA was used in the MSD assay.
Validation	*Describe the validation of each primary antibody for the species and application, noting any validation statements on the manufacturer's website, relevant citations, antibody profiles in online databases, or data provided in the manuscript.*

Eukaryotic cell lines

Policy information about cell lines

Cell line source(s)	293T cell line stably overexpressing the human ACE2 cell surface receptor protein. These cells were named 293T-hACE2.MF

Cell line source(s)	and were shown to be mycoplasma free. Manufacturer: Drs. Mike Farzan and Huihui Mu at Scripps Clinic and Research Foundation, La Jolla, California.
Authentication	*Describe the authentication procedures for each cell line used OR declare that none of the cell lines used were authenticated.*
Mycoplasma contamination	The 293T cell line has been shown to be free of mycoplasma.
Commonly misidentified lines (See ICLAC register)	*Name any commonly misidentified cell lines used in the study and provide a rationale for their use.*

Human research participants

Policy information about studies involving human research participants

Population characteristics	Please see Table 1 in the main text of the manuscript. The majority of participants were White and not Hispanic or Latinx in this phase 2 study and the phase 3 COVE trials, but there were higher percentages of Black and Hispanic or Latinx participants in the latter. The mean age of the participants in the groups that received the booster were 52.0 years in the phase 2 study Part B and 54.5 years for those who received 2 doses in the phase 3 COVE trial.
Recruitment	The blinded portion of the P201 clinical trial recruited healthy volunteer participants from the general poipulation across 8 clinical trial sites throughout the United S?tates. Following Moderna's Emergency Use Authorization in December 2020, the P201 study protocol was amended to allow participants from the study to be unblinded during a Participant Decision Visit, and move to the Part B open label part of the study.
Ethics oversight	The study protocol was approved by Advarra, Inc., Columbia, MD.

Note that full information on the approval of the study protocol must also be provided in the manuscript.

Clinical data

Policy information about clinical studies

All manuscripts should comply with the ICMJE guidelines for publication of clinical research and a completed CONSORT checklist must be included with all submissions.

Clinical trial registration	NCT04405076
Study protocol	Online at Nature Medicine.
Data collection	Data were collected at 8 US sites. Investigator sites: Benchmark Research, 3100 Red River St, Suite 1, Austin, TX 78705; Meridian Clinical Research, 340 Eisenhower Drive, Suite 1200, Savannah, GA 31406; Meridian Clinical Research, 1410 North 13th Street, Suite 5, Norfolk, NE 68701; Meridian Clinical Research, 4802 Sunnybrook Dr., Sioux City, IA 51106; Alliance for Multispecialty Research, 700 Medical Drive, Suite 110, Newton, Kansas 67114; Trial Management Associates, 3806 Peachtree Avenue, Suite 200, Wilmington, NC 28403; Alliance for Multispecialty Research, LLC, 1010 Carondelet Drive, Suite 426,Kansas City, MO 64114; Benchmark Research -San Angelo, 3605 Executive Drive, San Angelo, TX 76904 The phase 2 Part A trial consisted of a total of 600 participants who received placebo, 50 µg or 100 µg of mRNA-1273 from May 29, 2020 to July 8, 2020. Of the 344 participants who received a booster dose in Part B from January 28, 2021 to April 27, 2021, 173 received two doses of 50 µg of mRNA-1273 and 171 received two doses of 100 µg of mRNA-1273 6 to 8 months earlier in Part A.
Outcomes	From Methods in Supplementary Information: The primary safety objective of Part B was to evaluate the safety and reactogenicity of 50 µg of mRNA-1273 administered as a single booster dose 6 months or more after a priming series of 50 µg or 100 µg of mRNA 1273. The primary safety endpoints were solicited local and systemic adverse reactions (ARs) through 7 days after each injection, unsolicited treatment-emergent adverse events (TEAEs) through 28 days after each injection, medically-attended AEs (MAAEs) and serious AEs (SAEs) throughout the entire study period. The primary immunogenicity objective was to evaluate the immunogenicity of 50 µg of mRNA-1273 administered as a single booster dose administered at least 6 months after a two-dose priming series with 50 or 100 µg of mRNA-1273 as compared to 100 µg of mRNA-1273 administered as 2 doses 28 days apart in the pivotal phase 3 efficacy and safety study (COVE), as assessed by the level of SARS-CoV-2-specific neutralizing antibody (nAb). The coprimary endpoints for noninferiority were: (i) Geometric mean (GM) titers of serum nAb and (ii) Seroresponse rates for nAb based on the pseudovirus neutralizing antibody assay. The secondary immunogenicity objective was to evaluate the immunogenicity of 50 µg of mRNA-1273 vaccine administered as a single booster dose as assessed by the titers of bAb. Levels of SARS-CoV-2-specific bAb were measured by enzyme-linked immunosorbent assay (ELISA) and a SARS-CoV-2 Meso-Scale Discovery (MSD) 3-PLEX assay on Open-label Day 1 ([OL-Dl]; pre-boost) and Open-label Day 29 ([OL-Day 29]; 28 days after the booster injection). Seroresponse was defined in 3 ways: i) seroresponse (specific to the IDS0 titer in the pseudovirus neutralizing antibody assay) was defined as a change from below the lower limit of quantification (LLOQ) at pre-booster (or pre-dose 1) to equal or above LLOQ at 28 days after the booster (or 28 days after the primary series), or at least a 3.3-fold rise at 28 days after the booster (or 28 days after the primary series) if pre-booster (or pre-dose 1) titer was equal to or above LLOQ; ii) Seroresponse (4-fold rise) was defined as a change of titer from below the LLOQ at pre-booster (or pre-dose 1) to equal to or above 4 x LLOQ at 28 days after the booster (or 28 days after the primary series), or a 4-times or higher ratio in participants with titers above the LLOQ at pre-booster (or pre-dose 1); and iii) Seroresponse (4-fold rise from baseline) was defined as a change of titer from below the LLOQ at baseline (pre-dose 1 in the primary series) to equal to or above 4 x LLOQ at 28 days after the booster (or 28 days after the primary series), or a 4-times or higher ratio in participants with titers above the LLOQ at pre-dose 1. Definition iii) was only applied to participants in the Phase 2 study (primary series in Part A and booster in Part B).
