# Supplementary material for: Immune response to SARS-CoV-2 after a booster of mRNA-1273: an open-label phase 2 trial
Source: Nat Med. 2022 Mar 3;28(5):1042–9. doi: 10.1038/s41591-022-01739-w (PMC9117133; doi:10.1038/s41591-022-01739-w)
Supplement: Supplementary file 3 — A phase 2a, randomized, observer-blind, placebo-controlled, dose-confirmation study to evaluate the safety, reactogenicity and immunogenicity of mRNA-1273 SARS-CoV-2 vaccine in adults aged 18 years and older [file 41591_2022_1739_MOESM3_ESM.pdf]

CLINICAL STUDY PROTOCOL

A PHASE 2a, RANDOMIZED, OBSERVER-BLIND, PLACEBO-CONTROLLED, DOSE-CONFIRMATION STUDY TO EVALUATE THE SAFETY, REACTOGENICITY, AND IMMUNOGENICITY OF MRNA-1273 SARS-COV-2 VACCINE IN ADULTS AGED 18 YEARS AND OLDER

IND NUMBER: 19745
PROTOCOL NUMBER: mRNA-1273-P201

**Sponsor:**	ModernaTX, Inc.<br>200 Technology Square<br>Cambridge, MA 02139
**Sponsor Contact:**	Roderick McPhee, MD, PhD<br>Director, Clinical Development<br>ModernaTX, Inc.<br>200 Technology Square<br>Cambridge, MA 02139<br>Telephone: 1-617-301-1186<br>e-mail: Roderick.McPhee@modernatx.com
**Amendment Number:**	6
**Date of Amendment 6:**	28 Apr 2021
**Date of Amendment 5:**	19 Feb 2021
**Date of Amendment 4:**	15 Jan 2021
**Date of Amendment 3:**	02 Sep 2020
**Date of Amendment 2:**	01 Jul 2020
**Date of Amendment 1:**	18 May 2020
**Date of Original Protocol:**	22 Apr 2020

CONFIDENTIAL

The concepts and information contained in this document or generated during the study are considered proprietary and may not be disclosed in whole or in part without the expressed, written consent of ModernaTX, Inc.

The study will be conducted according to E6(R2) Good Clinical Practice: International Council for Harmonisation (E6(R2) Good Clinical Practice: Integrated Addendum to ICH E6(R1).

Signature Page

**PROTOCOL TITLE:**	A Phase 2a, Randomized, Observer-Blind, Placebo-Controlled, Dose-Confirmation Study to Evaluate the Safety, Reactogenicity, and Immunogenicity of mRNA-1273 SARS-CoV-2 Vaccine in Adults Aged 18 Years and Older
**PROTOCOL NUMBER:**	mRNA-1273-P201
**AMENDMENT NUMBER:**	6
**AMENDMENT 6 DATE:**	28 Apr 2021

See esignature & date signed on last of the document

_______________________________________ _______________________

Roderick McPhee, MD, PhD Date

Director, Clinical Development, Infectious Disease

ModernaTX, Inc.

Investigator Protocol Agreement Page

I agree to conduct the study as outlined in the protocol entitled "A Phase 2a, Randomized, Observer-Blind, Placebo-Controlled, Dose-Confirmation Study to Evaluate the Safety, Reactogenicity, and Immunogenicity of mRNA-1273 SARS-CoV-2 Vaccine in Adults Aged 18 Years and Older" in accordance with the guidelines and all applicable government regulations including US Title 21 of the Code of Federal Regulations Part 54. I have read and understand all sections of the protocol.

______________________________ ______________________
Signature of Investigator Date

____
Printed Name of Investigator

Protocol Amendment Summary of Changes

DOCUMENT HISTORY	
**Document**	**Date**
Amendment 6	28 Apr 2021
Amendment 5	19 Feb 2021
Amendment 4	15 Jan 2021
Amendment 3	02 Sep 2020
Amendment 2	01 Jul 2020
Amendment 1	18 May 2020
Original Protocol	22 Apr 2020

Amendment 6, 22 Apr 2021: Current Amendment

Main Rationale for the Amendment:

This protocol amendment will add an analysis at the end of Part A. An analysis of safety and immunogenicity data will be performed after all participants have completed Part A of the study. All data collected in Part A of the study will be cleaned (ie, data that are as clean as possible) and locked and a report may be generated as needed.

The summary of changes table provided here describes the major changes made in Amendment 6 relative to Amendment 5, including the sections modified and the corresponding rationales. The synopsis of Amendment 6 has been modified to correspond to changes in the body of the protocol.

Summary of Major Changes in Protocol Amendment 6:

Section # and Name	Description of Change	Brief Rationale
Synopsis and Section 4.7.6 (End of Study Analysis)	End of Part A clarified	To clarify that an analysis will be performed at the conclusion of Part A
Synopsis and Section 4.7.2 (Analysis at End of Blinded part A Only)	Added an analysis of safety and immunogenicity at the end of Part A	To clarify that an analysis of safety and immunogenicity will be performed on all participants upon completion of Part A of the study.

IRB and Regulatory Authority Approval

A copy of this amended protocol will be sent to the institutional review board (IRB) and regulatory authority.

The changes described in this amended protocol require IRB approval prior to implementation. In addition, if the changes herein affect the informed consent, sites are required to update and submit a revised informed consent for approval that incorporates the changes described in this amended protocol.

Table of Contents

Signature Page ... 2

Investigator Protocol Agreement Page ... 3

Protocol Amendment Summary of Changes ... 4

IRB and Regulatory Authority Approval ... 5

List of Tables ... 11

List of Figures ... 11

Protocol Synopsis ... 12

List of Abbreviations ... 26

1 INTRODUCTION ... 29

1.1 Background ... 29

1.2 Nonclinical Studies in Development of mRNA-1273 ... 31

1.3 Clinical Studies With Lipid Nanoparticle mRNA Vaccines ... 31

2 STUDY OBJECTIVES ... 34

2.1 Primary Objectives (Part A, Blinded) ... 34

2.1.1 Primary Safety Objective ... 34

2.1.2 Primary Immunogenicity Objective ... 34

2.2 Secondary Objective (Part A, Blinded) ... 34

2.3 Exploratory Objectives (Part A, Blinded) ... 34

2.4 Primary Objectives (Part B, Open Label) ... 35

2.4.1 Primary Safety Objective ... 35

2.4.2 Primary Immunogenicity Objective ... 35

2.5 Secondary Objective (Part B, Open Label) ... 35

2.6 Exploratory Objectives (Part B, Open Label) ... 35

2.7 Primary Objectives (Part C, Open Label) ... 36

2.7.1 Primary Safety Objective ... 36

2.7.2 Primary Immunogenicity Objective ... 36

2.8 Secondary Immunogenicity Objective (Part C, Open Label) ... 36

2.9 Exploratory Objectives (Part C, Open Label) ... 36

3 INVESTIGATIONAL PLAN ... 37

3.1 General Study Design ... 37

3.1.1 Part A, the Blinded Phase ... 37
3.1.2 Part B, Open-Label Interventional Phase ... 41
3.1.3 Part C, Open-Label Interventional Phase of mRNA-1273.351 and mRNA-1273/mRNA-1273.351 Mixture Booster Vaccines ... 43
3.1.4 Rationale for Dose Selection ... 44
3.1.5 Rationale for Study Design ... 45
3.1.6 Inclusion Criteria ... 46
3.1.6.1 Inclusion criterion specific for Parts A and B: ... 47
3.1.6.2 Inclusion criterion specific for Part C: ... 48
3.1.7 Exclusion Criteria ... 49
3.1.7.1 Exclusion Criteria (Part A and Part B) ... 49
3.1.7.2 Exclusion Criteria (Part C) ... 51
3.1.8 Screen Failures (Part A: Blinded Phase Only) ... 53
3.1.9 Participant Restrictions During the Study ... 53
3.1.9.1 General and Dietary ... 53
3.2 Withdrawal of Participants From the Study or Study Dosing ... 53
3.2.1 Participant Withdrawal From the Study ... 53
3.2.2 Handling Withdrawal From the Study ... 54
3.2.3 Lost to Follow-up ... 54
3.2.4 Replacements (Part A, Blinded Phase Only) ... 54
3.2.5 Participant Withdrawal From Study Dosing ... 54
3.3 Study Dosing Groups ... 55
3.3.1 Method of Assigning Participants to Dosing Groups (Part A and Part C) ... 55
3.3.2 Investigational Product Administration ... 57
3.3.2.1 Pause Rules ... 58
3.3.2.2 Contraindications to Subsequent Injection ... 59
3.3.3 Identity of Investigational Product ... 60
3.3.4 Management of Investigational Product ... 61
3.3.4.1 Delivery and Receipt ... 61
3.3.4.2 Packaging and Labeling ... 61
3.3.4.3 Storage ... 62
3.3.4.4 Investigational Product Accountability ... 62
3.3.4.5 Handling and Disposal ... 63
3.3.5 Blinding ... 63
3.3.6 Breaking the Blind ... 64
3.3.7 Dosing Compliance ... 64
3.3.8 Prior and Concomitant Medications ... 65

3.3.8.1 Prior Medications and Therapies ... 65
3.3.8.2 Concomitant Medications and Therapies ... 65
3.4 Study Procedures ... 66
3.4.1 Assessment for SARS-CoV-2 Infection ... 67
3.4.2 Use of Electronic Diaries ... 68
3.4.3 Safety Telephone Calls ... 70
3.4.4 Safety Laboratory Assessments ... 71
3.4.5 Immunogenicity Assessments ... 71
3.4.6 Blood Sampling Volumes ... 72
3.4.7 Safety Assessments ... 73
3.4.8 Safety Definitions ... 74
3.4.8.1 Adverse Event ... 74
3.4.8.2 Medically Attended Adverse Event ... 75
3.4.8.3 Serious Adverse Event ... 75
3.4.8.4 Solicited Adverse Reactions ... 76
3.4.8.5 Pregnancy ... 80
3.4.8.6 Definition and Reporting of Adverse Events Consistent with Anaphylaxis ... 81
3.4.8.7 Eliciting and Documenting Adverse Events ... 82
3.4.8.8 Reporting Adverse Events ... 82
3.4.8.9 Assessment of Severity ... 83
3.4.8.10 Assessment of Causality ... 84
3.4.8.11 Follow-up of Adverse Events ... 84
3.4.9 Vital Sign Measurements ... 84
3.4.10 Physical Examinations ... 85
4 STATISTICAL ANALYSIS PLAN ... 86
4.1 Blinding and Responsibility for Analyses ... 86
4.2 Hypothesis Testing ... 87
4.3 Analysis Endpoints ... 87
4.3.1 Part A, Blinded ... 87
4.3.1.1 Primary Endpoints ... 87
4.3.1.2 Secondary Endpoints ... 87
4.3.1.3 Exploratory Endpoints ... 88
4.3.2 Part B, Open Label ... 88
4.3.2.1 Primary Endpoints (Part B, Open Label) ... 88
4.3.2.2 Secondary Endpoints (Part B, Open Label) ... 89

4.3.2.3 Exploratory Endpoints (Part B, Open Label) 89
4.3.3 Part C, Open Label 90
4.3.3.1 Primary Endpoints (Part C, Open Label).................................... 90
4.3.3.2 Secondary Immunogenicity Endpoint 90
4.3.3.3 Exploratory Endpoints (Part C, Open Label) 91
4.4 Analysis Populations.. 91
4.4.1 Randomized Set 91
4.4.2 Solicited Safety Set... 91
4.4.3 Safety Set 91
4.4.4 Full Analysis Set 92
4.4.5 Per-Protocol Set 92
4.5 Sample Size Determination.. 92
4.6 Statistical Methods... 93
4.6.1 Summary of Baseline Characteristics and Demographics 93
4.6.2 Safety Analyses.. 94
4.6.3 Immunogenicity Analyses 95
4.6.4 Exploratory Analyses... 96
4.7 Study Analyses... 97
4.7.1 Primary Study Analysis for Blinded Part A Only 97
4.7.2 Analysis at End of Blinded Part A Only 97
4.7.3 Interim Study Analysis for Open-Label Part B Only.................................... 97
4.7.4 Interim Study Analysis for Open-Label Part C Only.................................... 97
4.7.5 Additional Study Analyses 97
4.7.6 End of Study Analysis 98
4.8 Data Quality Assurance.. 98
5 INVESTIGATOR OBLIGATIONS.. 99
5.1 Confidentiality 99
5.2 Institutional Review 99
5.3 Participant Consent 99
5.4 Study Reporting Requirements... 100
5.5 Financial Disclosure and Obligations 100
5.6 Investigator Documentation.. 101
5.7 Study Conduct.. 101
5.8 Data Collection 102
5.8.1 Case Report Forms and Source Documents 102

5.9 Adherence to Protocol 102
5.10 Reporting Adverse Events 102
5.11 Investigator's Final Report 102
5.12 Records Retention 103
5.13 Publications 103
6 STUDY MANAGEMENT 104
6.1 Monitoring 104
6.1.1 Safety Monitoring Committee 104
6.1.2 Monitoring of the Study 104
6.1.3 Inspection of Records 105
6.2 Management of Protocol Amendments and Deviations 105
6.2.1 Modification of the Protocol 105
6.2.2 Protocol Deviations 105
6.3 Study Termination 105
6.4 Clinical Study Reports 106
7 APPENDICES 107
7.1 Appendix 1: Schedule of Events 107
7.2 Appendix 2: Toxicity Grading Scale Tables 118
7.3 Appendix 3: Protocol Amendment History 123
7.3.1 Amendment 5, 19 Feb 2021 123
7.3.2 Amendment 4, 15 Jan 2021 129
7.3.3 Amendment 3, 02 Sep 2020 132
7.3.4 Amendment 2, 01 Jul 2020 133
7.3.5 Amendment 1, 18 May 2020 134
8 REFERENCE LIST 138

List of Tables

Table 1: Dose Group Assignment (Part A) 56

Table 2: Dose Group Assignment (Part C) 56

Table 3: Pause Rule Criteria, Events, and Thresholds 58

Table 4: Maximum Blood Sampling Volumes per Participant by Visit (Part A, Blinded) . 73

Table 5: Maximum Blood Sampling Volumes per Participant by Visit (Part B and Part C Open-label) 73

Table 6: Solicited Adverse Reactions and Grades 78

Table 7: 95% Confidence Interval for One Participant with AE and the Lowest Detectable Incidence Rate at 95% Probability in Selected Sample Size 93

Table 8: Analysis Strategy for Safety Parameters 95

Table 9: Schedule of Events (Part A, Blinded Phase only) 108

Table 10: Part B: Participant Decision Clinic Visit 111

Table 11: Part B: Open-label Schedule of Events: ONLY for Participants Receiving 2 Doses of mRNA-1273 28 Days Apart 112

Table 12: Part B and Part C: Open-label Schedule of Events (Participants Receiving a Booster Dose)[9] 115

Table 13: Tables for Clinical Abnormalities 118

Table 14: Tables for Laboratory Abnormalities 121

List of Figures

Figure 1: Study Flow Schema (Part A, Blinded Phase) 38

Figure 2: Sentinel and Expansion Cohort Schema (Part A, Blinded Phase) 39

Figure 3: Part B, Open-Label Schema 41

Figure 4: Part C, Open-Label Schema for Boosting with mRNA-1273.351 and mRNA-1273/mRNA-1273.351 Mixture 43

Protocol Synopsis

**Protocol Number:**	**mRNA-1273-P201**
**Title:**	A Phase 2a, Randomized, Observer-Blind, Placebo-Controlled, Dose-Confirmation Study to Evaluate the Safety, Reactogenicity, and Immunogenicity of mRNA-1273 SARS-CoV-2 Vaccine in Adults Aged 18 Years and Older
**Study Phase:**	2
**Study Sites:**	Approximately 10 study sites in the United States or its territories.
**Objectives:**	**PART A (BLINDED)**

Primary:

- To evaluate the safety and reactogenicity of 2 dose levels of mRNA-1273 vaccine, each administered in 2 doses 28 days apart
- To evaluate the immunogenicity of 2 dose levels of mRNA-1273 vaccine, each administered in 2 doses 28 days apart, as assessed by the level of specific binding antibody (bAb)

Secondary:

- To evaluate the immunogenicity of 2 dose levels of mRNA-1273 vaccine, each administered in 2 doses 28 days apart, as assessed by the titer of neutralizing antibody (nAb)

Exploratory:

- To profile spike (S) protein-specific serum immunoglobulin (Ig) class and subclass and nAb in serum
- To describe the ratio or profile of specific bAb relative to nAb in serum
- To describe initial immunogenicity responses following the first dose (Day 1) and prior to the second dose (Day 29)
- To characterize the clinical profile and immune response of participants infected by SARS-CoV-2
- To evaluate the effect of the mRNA-1273 vaccine on the incidence of SARS-CoV-2 infection

PART B (OPEN LABEL)

Primary:

- To evaluate the safety and reactogenicity of 50 µg of mRNA-1273 vaccine administered as a single booster dose or 100 µg of mRNA-1273 administered as 2 doses 28 days apart
- To evaluate the immunogenicity of 50 µg of mRNA-1273 vaccine administered as a single booster dose or 100 µg of mRNA-1273 administered as 2 doses 28 days apart, as assessed by the level of specific bAb

Secondary:

- To evaluate the immunogenicity of 50 µg of mRNA-1273 vaccine administered as a single booster dose or 100 µg of mRNA-1273 administered as 2 doses 28 days apart, as assessed by the titer of nAb

Exploratory:

- To profile S protein-specific serum Ig class and subclass and nAb in serum
- To describe the ratio or profile of specific bAb relative to nAb in serum
- To describe initial immunogenicity responses at various time points following a booster dose, where applicable
- To characterize the clinical profile and immune response of participants infected by SARS-CoV-2
- To evaluate the effect of the mRNA-1273 vaccine on the incidence of SARS-CoV-2 infection

PART C (OPEN LABEL)

Primary:

- To evaluate the safety and reactogenicity of 2 dose levels (20 and 50 µg) of mRNA-1273.351 or one dose level of a mixture of 25 µg mRNA-1273 and 25 µg mRNA-1273.351 (mRNA-1273/mRNA-1273.351 mixture) (50 µg total) vaccine administered as a single booster dose after completing a two-dose vaccination schedule of mRNA-1273 at least 6 months earlier
- To evaluate the immunogenicity of 2 dose levels (20 and 50 µg) of mRNA-1273.351 or one dose level of mRNA-1273/mRNA-1273.351 mixture (50 µg total) vaccine administered as a single booster dose after completing a two-dose vaccination schedule of mRNA-1273 at least 6

months earlier, as assessed by the level of specific nAb directed against the S2P antigen derived from B.1.351 variant

Secondary:

- To evaluate the immunogenicity of 2 dose levels (20 and 50 µg) of mRNA-1273.351 or one dose level of mRNA-1273/mRNA-1273.351 mixture (50 µg total) vaccine administered as a single booster dose after completing a two-dose vaccination schedule of mRNA-1273 at least 6 months earlier, as assessed by the level of specific bAb directed against the S2P antigen derived from B.1.351 variant

Exploratory:

- To profile S protein-specific serum Ig class and subclass and nAb in serum
- To describe the ratio or profile of specific bAb relative to nAb in serum
- To describe initial immunogenicity responses at various time points for 6 months following the first and second booster dose, as applicable
- To characterize the clinical profile and immune response of participants infected by SARS-CoV-2
- To evaluate the effect of the mRNA-1273.351 and mRNA-1273/mRNA-1273.351 mixture vaccines on the incidence of SARS-CoV-2 infection
- To further assess the immune response to vaccines containing the B.1.351 and the D614G variant sequences

Study Design and Methodology:

This is a three-part Phase 2a study: Part A, Part B, and Part C. Participants in Part A, the Blinded Phase of this study, are blinded to their treatment assignment. Part B, the Open-Label Interventional Phase of this study, is designed to offer participants who received placebo in Part A of this study the option to receive 2 injections of open-label mRNA-1273. Participants who received 1 or 2 doses of 50 µg or 100 µg mRNA-1273 in Part A will be offered a single booster dose of mRNA-1273 (50 µg) in Part B. Part C will be a proof-of-concept rollover study of approximately 60 participants, who are currently enrolled in Moderna's Phase 3 mRNA-1273-P301 COVE study, have already been unblinded, and have previously received 2 doses of mRNA-1273 at least 6 months earlier. Upon enrollment into Part C of this study, they will receive a single IM injection of mRNA-1273.351 (20 µg or 50 µg) or mRNA-1273/mRNA-1273.351 mixture (50 µg total) at least 6 months after receiving the second vaccination in the mRNA-1273-P301 COVE study. At enrollment into this study, their participation in mRNA-1273-P301 COVE study will be terminated.

Part A, the Blinded Phase:

The Blinded Phase of this study is randomized, observer-blind, and placebo-controlled, with adult participants at least 18 years of age.

Two dose levels (50 µg and 100 µg) will be evaluated in this study, based in part on initial safety data from the Phase 1 Division of Microbiology and Infectious Diseases (DMID) study of mRNA-1273. The study will include 2 age cohorts: Cohort 1 with 300 participants (≥ 18 to < 55 years old) and Cohort 2 with 300 participants (≥ 55 years old). Approximately 600 participants will receive either mRNA-1273 vaccine or saline placebo control according to a 1:1:1 randomization ratio; ie, within each age cohort, 100 participants will receive mRNA-1273 50 µg, 100 participants will receive mRNA-1273 100 µg, and 100 participants will receive saline placebo.

The study will be initiated with a parallel enrollment of all 300 participants in Cohort 1 (≥ 18 to < 55 years old) and a sentinel group of 50 participants in Cohort 2 (≥ 55 years old) receiving study treatment. Before initiating study treatment of the remaining participants in Cohort 2, safety data through Day 7 from the sentinel group of Cohort 2 and all available data from Cohort 1 will be reviewed by the Safety Monitoring Committee (SMC).

If no safety concerns are found, expansion enrollment (N=250) of Cohort 2 will proceed.

Part A of the study comprises up to 10 scheduled study site visits: Screening, Day 1, Day 8, Day 15, Day 29 (Month 1), Day 36, Day 43,

Day 57 (Month 2), Day 209 (Month 7), and Day 394 (Month 13) or initiation of Part B, whichever is earlier. There are also scheduled participant contacts approximately every 2 weeks after Day 57 to collect medically attended adverse events (MAAEs), adverse events (AEs) leading to withdrawal, serious adverse events (SAEs), concomitant medications associated with these events, receipt of non-study vaccinations, exposure to someone with known COVID-19 or SARS-CoV-2 infection, and participant experience of COVID-19 symptoms. Every 4 weeks from Day 71 through Day 183 and from Day 223 through Day 363, each participant will complete a questionnaire in an electronic diary (eDiary) that will be reviewed by study site personnel. Safety telephone calls will occur every 4 weeks from Day 85 through Day 197 and from Day 237 through Day 377. The study duration for Part A will be approximately 14 months for each participant: a screening period of up to 1 month and a study period of 13 months that includes the first dose of vaccine on Day 1 and the second dose on Day 29. The participant's final visit will be on Day 394 (Month 13), 12 months after the second dose of vaccine on Day 29 (Month 1), or initiation of Part B, whichever is earlier.

To test for the presence of SARS-CoV-2, nasopharyngeal swab samples will be collected at Day 1, Day 29, and Day 57. During the course of the study, participants meeting pre-specified disease criteria that suggest possible SARS-CoV-2 infection will be asked to contact the study site to arrange for a prompt, thorough, and careful assessment.

Each participant will receive 2 injections of mRNA-1273 or placebo by 0.5 mL intramuscular (IM) injection on Day 1 and Day 29. Vaccine accountability, dose preparation, and vaccine administration will be performed by unblinded pharmacy personnel who will not participate in any other aspects of the study during Part A, the Blinded Phase. The remainder of the study staff, all participants, and Sponsor personnel (or its designees) will remain blinded to dosing assignment.

All participants will be followed for safety and reactogenicity and provide pre- and post-injection blood specimens for immunogenicity through 12 months after the last dose of investigational product.

The end of Part A is defined as the earlier of Visit 9, the completion of the last participant's last visit, or initiation of Part B. Participants are considered to have completed Part A of the study if they complete the final visit on Day 394 (Month 13) or initiated Part B of the study.

At each dosing visit, participants will be instructed (Day 1) or reminded (Day 29) how to document and report solicited adverse reactions (ARs) within a provided eDiary. Solicited ARs will be assessed for 7 days (the day of injection and the following 6 days) after each injection and

unsolicited AEs will be assessed for 28 days after each injection; SAEs and MAAEs will be assessed throughout the study.

Participants will have blood sampled at scheduled study site visits during the study for safety and immunogenicity assessments or other medical concerns according to the investigator's judgment. In addition, participants may have blood sampled at unscheduled visits for acute respiratory symptoms.

Part B, the Open-Label Interventional Phase:

Part B, the Open-Label Interventional Phase of the study, is prompted by the authorization of a COVID-19 vaccine under Emergency Use Authorization (EUA). Transitioning the study to Part B permits all ongoing study participants to be informed of the availability and eligibility criteria of any COVID-19 vaccine made available under an Emergency Use Authorization (EUA) and the option to offer all ongoing study participants an opportunity to schedule a Participant Decision Visit to know their original treatment assignment (placebo vs. mRNA-1273 [50 µg or 100 µg] vaccine).

Part B, the Open-Label Interventional Phase of the study, also provides the opportunity for study participants who previously received placebo to actively request to receive 2 doses of mRNA-1273 (100 µg) vaccine. In addition, all participants who previously received 1 or 2 doses (50 µg or 100 µg) of the mRNA-1273 vaccine will be able to receive a single 50 µg booster dose of mRNA-1273. All study participants will receive a Notification Letter summarizing the basis for a COVID-19 vaccine to receive an EUA and will be asked to schedule a Participant Decision Clinic Visit.

At the Participant Decision Clinic Visit, all participants will:

- Be given the option to be unblinded as to their original group assignment (placebo vs. mRNA-1273 vaccine [50 µg or 100 µg]),
- Be counselled about the importance of continuing other public health measures to limit the spread of disease including social distancing, wearing a mask, and hand-washing,
- Sign a revised ICF, and
- Provide a nasopharyngeal (NP) swab for RT-PCR for SARS-CoV-2 and a blood sample for serology and immunogenicity.

NOTE: After the Participant Decision Clinic Visit, all participants will proceed to the open label, Part B of the study, and will follow the Part B Schedule of Events as follows:

- **Participants received placebo in Part A and consent to unblinding and to receiving 2 doses of mRNA-1273 in Part B**: These participants will proceed to Part B.
- **Participants received 1 dose of mRNA-1273 in Part A (regardless of the initial dose level received) and consent to unblinding and to receiving booster injection of mRNA-1273 in Part B**: These participants will proceed to Part B.
- **Participants received 2 doses of mRNA-1273 in Part A and consent to unblinding and to receiving booster injection of mRNA-1273 dose in Part B:** These participants will proceed to Part B (note that these participants will receive 3 injections of mRNA-1273 during the course of the study).
- **Participants received 2 doses of mRNA-1273 in Part A and consent to unblinding, but decline receiving another booster injection of mRNA-1273 in Part B:** These participants will proceed to Part B.
- **Participants decline breaking of blind:** These participants will proceed to Part B.

PART C, the Open-Label Interventional Phase of mRNA-1273.351 and mRNA-1273/mRNA-1273.351 mixture booster vaccines:

Part C, the Open-Label Interventional Phase of the study of mRNA-1273.351 and mRNA-1273/mRNA-1273.351 mixture booster vaccines, is prompted by the need to pro-actively prepare for vaccination strategies that induce a broader protection, including variants such as B.1.351. This proof-of-concept study design will evaluate the safety and immunogenicity of two dose levels of mRNA-1273.351 (20 µg and 50 µg), and mRNA-1273/mRNA-1273.351 mixture (50 µg total), given as a single IM injection booster. Upon meeting eligibility criteria, approximately 60 participants from Moderna's Phase 3 mRNA-1273-P301 COVE study will be enrolled into this Part C to receive a single IM injection of mRNA-1273.351 (20 µg or 50 µg) or a single IM injection of mRNA-1273/mRNA-1273.351 mixture (50µg). An additional booster dose may be added approximately 56 days after the first boost at OL-D1. This additional booster dose will be triggered following review of immunogenicity data up to OL-D15 of the initial mRNA-1273.351 (20 µg and 50 µg) and mRNA-1273/mRNA-1273.351 mixture injections.

Study Population:

Participants (males and females 18 years of age or older at time of consent) will be included in the study if they are in good health according to the assessment of the investigator and can comply with study procedures. Negative pregnancy tests will be required at Screening and before vaccine administration for female participants of childbearing potential. The full lists of inclusion and exclusion criteria are provided in the body of the protocol.

Safety Assessments:

Safety assessments will include monitoring and recording of the following for each participant:

- Solicited local and systemic ARs that occur during the 7 days following each injection (ie, the day of injection and 6 subsequent days); solicited ARs will be recorded daily using eDiaries. (Part A, Blinded Phase, Part B, Open Label, and Part C, Open Label).
- Unsolicited AEs observed or reported during the 28 days following each injection (ie, the day of injection and 27 subsequent days); unsolicited AEs are AEs that are not included in the protocol-defined solicited ARs. (Part A, Blinded Phase, Part B, Open Label, and Part C, Open Label).
- AEs leading to discontinuation from dosing and/or study participation from Day 1 through Day 394 (Part A) and OL-Month 6 or 7 (Part B and Part C) or withdrawal from the study.
- MAAEs from Day 1 through Day 394 (Part A) and OL-Month 6 or 7 (Part B and Part C) or withdrawal from the study.
- SAEs from Day 1 through Day 394 (Part A) and OL-Month 6 or 7 (Part B and Part C) or withdrawal from the study.
- Results of safety laboratory tests (Part A).
- Vital sign measurements (Part A, Part B, and Part C).
- Physical examination findings (Part A, Part B, and Part C).
- Assessments for SARS-CoV-2 infection from Day 1 through study completion (Part A, Part B, and Part C).

**Immunogenicity Assessments:**	Immunogenicity assessments will include the following in Part A and Part B:<br>• Serum bAb level against SARS-CoV-2 as measured by enzyme-linked immunosorbent assay (ELISA) specific to the SARS-CoV-2 spike protein<br>• Serum nAb titer against SARS-CoV-2 as measured by pseudovirus and/or live virus neutralization assays<br>In Part C, serum samples for immunogenicity assessment will be analyzed for:<br>• Serum nAb against SARS-CoV-2 as measured by pseudovirus (and/or live virus) using the B.1.351 and the D614G variant sequences of SARS-CoV-2 S protein (and/or variant strains)<br>• Serum bAb against SARS-CoV-2 as measured by ELISA against the B.1.351 and the D614G variant sequences of SARS-CoV-2 S protein
**Investigational Product, Dosage, and Route of Administration:**	mRNA-1273 vaccine, dosed in Part A and Part B, is a lipid nanoparticle (LNP) dispersion of an mRNA encoding the prefusion stabilized S protein of SARS-CoV-2 formulated in LNPs composed of 4 lipids (1 proprietary and 3 commercially available). The mRNA-1273 vaccine is provided as a sterile liquid for injection, white to off white dispersion in appearance, at a concentration of 0.5 mg/mL in 20 mM Tris buffer containing 87 mg/mL sucrose and 10.7 mM sodium acetate at pH 7.5.<br>The mRNA-1273 vaccine dosed in Part C is provided at a concentration of 0.2 mg/mL in 20 mM Tris buffer containing 87 mg/mL sucrose and 4.3 mM sodium acetate at pH 7.5.<br>mRNA-1273.351 vaccine, dosed in Part C, is a LNP dispersion, with an mRNA encoding the prefusion stabilized S protein of SARS-CoV-2 variant B1.351. The mRNA is formulated in LNPs composed of 4 lipids (1 proprietary and 3 commercially available). The mRNA-1273.351 vaccine in Part C is in 20 mM Tris buffer containing 10.7 mM of sodium acetate at pH 7.5 and provided at a concentration of 0.5 mg/mL. mRNA-1273 and mRNA-1273.351 vaccines will be mixed at the clinical site to enable dosing of the mRNA-1273/mRNA-1273.351 (50 µg total) arm.<br>The placebo is 0.9% sodium chloride (normal saline) injection, United States Pharmacopeia (USP).<br>Investigational product will be administered as an IM injection into the deltoid muscle on a 2-dose injection schedule on Day 1 and Day 29 in Part A and on Open Label (OL) Days 1 and 29 in Part B

(for participants receiving 2 doses of mRNA-1273), with a 28-day interval between doses. Participants receiving a single booster dose of mRNA-1273 in Part B will receive their dose on OL-D1. Each injection will have a volume of 0.5 mL and contain mRNA-1273 50 µg, mRNA-1273 100 µg, or saline placebo. Upon consent to roll over, participants in Part C will receive a single booster dose of mRNA-1273.351 (20 µg or 50 µg) or mRNA-1273/mRNA-1273.351 mixture (50 µg total). Preferably, the vaccine should be administered into the nondominant arm.

Unblinded pharmacy personnel, who will not participate in any other aspect of the study during Part A, the Blinded Phase, will perform vaccine accountability, dose preparation, and vaccine administration. In Part B, study site personnel who were blinded during the Blinded Phase, will be unblinded at the Participant Decision Visit.

Part B and C of the study will be open label.

Sample Size:

There is no hypothesis testing in this study. The number of proposed participants is considered sufficient to provide a descriptive summary of the safety and immunogenicity of different dose levels of mRNA-1273.

In the blinded Part A, approximately 600 participants will be randomly assigned in a 1:1:1 ratio to mRNA-1273 50 µg, mRNA-1273 100 µg, or placebo. A total of 400 participants will receive mRNA-1273, 200 participants in each dose level, or 100 participants in each age cohort and dose level. A sample size of 400 has at least a 95% probability to observe at least 1 participant with an AE at a true 0.75% AE rate. Participants who received any injection in Part A will have the opportunity to enter Part B provided that they meet the eligibility criteria.

In the open-label Part C, approximately 60 participants from Moderna’s Phase 3 mRNA-1273-P301 COVE study will be enrolled and will be assigned to receive a single IM injection of mRNA-1273.351 (20 µg or 50 µg) or mRNA-1273/mRNA-1273.351 mixture (50 µg total). The enrollment and vaccination in each study arm will be sequential as follows. The first 20 participants will be enrolled and dosed on OL-D1 with the mRNA-1273.351 50 µg dose. Upon completion of the first 50 µg dose arm, 20 participants will be enrolled in the second arm and will receive the mRNA-1273/mRNA-1273.351 mixture (50 µg total dose). Following completion of the second arm, 20 participants will be enrolled and dosed with the mRNA-1273.351 20 µg dose.

**Statistical Methods:**	**General Considerations:** Data from Part A and Part B will be presented separately, as applicable. All analyses will be performed by treatment group overall (for the 2 cohorts combined) and for the 2 cohorts separately, unless specified otherwise. Data from Part C may be presented separately, as appropriate.<br><br>For categorical variables, frequencies and percentages will be presented. Continuous variables will be summarized using descriptive statistics (number of participants, mean, median, standard deviation, minimum, and maximum).<br><br>**Safety:** Safety and reactogenicity will be assessed by clinical review of all relevant parameters including solicited ARs (local and systemic events), unsolicited AEs, SAEs, MAAEs, AEs leading to discontinuation, safety laboratory test results, vital signs, and physical examination findings.<br><br>Solicited ARs and unsolicited AEs will be coded by system organ class and preferred term according to the Medical Dictionary for Regulatory Activities (MedDRA) for Adverse Reaction Terminology. The Toxicity Grading Scale for Healthy Adult and Adolescent Volunteers Enrolled in Preventive Vaccine Clinical Trials is used in this study with modification for rash, solicited ARs, and vital signs.<br><br>Rash will be graded in the following manner:<br>• Grade 0 = no rash<br>• Grade 1 = localized without associated symptoms<br>• Grade 2 = maculopapular rash covering < 50% body surface area<br>• Grade 3 = urticarial rash covering > 50% body surface area<br>• Grade 4 = generalized exfoliative, ulcerative, or bullous dermatitis<br><br>All safety analyses will be based on the Safety Set, except summaries of solicited ARs, which will be based on the Solicited Safety Set. All safety analyses for Part A and Part B will be provided by age cohort unless otherwise specified.<br><br>The number and percentage of participants with any solicited local AR, with any solicited systemic AR, and with any solicited AR during the 7-day follow-up period after each injection will be summarized. A 2-sided 95% exact confidence interval (CI) using the Clopper-Pearson method will be provided for the percentage of participants with any solicited AR.<br><br>Number and percentage of participants with unsolicited AEs, SAEs, MAAEs, Grade 3 or higher AEs, and AEs leading to discontinuation from study vaccine or participation in the study will be summarized.

Number of events of solicited ARs, unsolicited AEs/SAEs, and MAAEs will be reported in summarization tables accordingly.

For all other safety parameters, descriptive summary statistics will be provided.

For treatment-emergent safety laboratory tests results, the raw values and change from baseline values will be summarized by age cohort (for Part A and Part B), treatment group, and visit at each timepoint.

The number and percentage of participants who have chemistry, hematology, coagulation, and vital signs results below or above the laboratory normal ranges will be tabulated by timepoint.

Demographic variables (eg, age, height, weight, and body mass index (BMI) and baseline characteristics will be summarized by treatment group for each age cohort (when appropriate) by descriptive statistics (mean, standard deviation for continuous variables, and number and percentage for categorical variables).

Data from Part C will be analyzed similarly.

Immunogenicity: The analyses of immunogenicity will be based on the Per-Protocol (PP) Set. For each age cohort (for Part A and Part B), if the number of participants in the Full Analysis Set (FAS) and PP Set differ (defined as the difference divided by the total number of participants in the PP Set) by more than 10%, supportive analyses of immunogenicity may be conducted using the FAS.

For the primary immunogenicity endpoint (secondary immunogenicity in Part C), geometric mean (GM) of specific bAb with corresponding 95% CI at each timepoint and geometric mean fold-rise (GMFR) of specific bAb with corresponding 95% CI at each post-baseline timepoint over pre-injection baseline at Day 1 will be provided by treatment group and age cohort (for Part A and Part B). Descriptive summary statistics including median, minimum, and maximum will also be provided.

For the secondary immunogenicity endpoint (primary immunogenicity endpoint for Part C), geometric mean titer (GMT) of specific nAb with corresponding 95% CI at each timepoint and GMFR of specific nAb with corresponding 95% CI at each post-baseline timepoint over pre-injection baseline at Day 1 will be provided by treatment group and age cohort (for Part A and Part B). Descriptive summary statistics including median, minimum, and maximum will also be provided. For summarizations of GMT values, antibody values reported as below the lower limit of quantification (LLOQ) will be replaced $0.5 \times$ LLOQ. Values that are greater than the upper limit of quantification (ULOQ) will be converted to the ULOQ.

	The number and percentage of participants with fold-rise ≥ 2, fold-rise ≥ 3, and fold-rise ≥ 4 of serum SARS-CoV-2-specific nAb titers and participants with seroconversion from baseline will be provided with 2-sided 95% CI using the Clopper-Pearson method at each post-baseline timepoint. Seroconversion at a participant level is defined as a change of nAb titer from below the LLOQ to equal to or above LLOQ, or a 4-times or higher titer ratio in participants with pre-existing nAb titers.<br><br>Exploratory analyses of each dose level of mRNA-1273 versus placebo (in Part A) on bAb and nAb levels/titers may be performed. Further details will be described in the Statistical Analysis Plan.<br><br>Data from Part C will be analyzed similarly.
**Study Analyses**	A primary analysis of safety and immunogenicity data will be performed after participants have completed Day 57 study procedures for blinded Part A only. This primary analysis may be performed when all participants in Cohort 1 and the Cohort 2 sentinel group have completed Day 57 study procedures and/or when all participants in Cohort 1 and Cohort 2 have completed Day 57 study procedures. All data relevant to the primary study analysis through Day 57 visit will be cleaned (ie, data that are as clean as possible) and locked. A limited number of Sponsor and clinical research organization personnel will be unblinded to perform the primary study analysis and prepare a final Clinical Study Report (CSR), including individual listings. The study site staff, investigators, study monitors, and participants will remain blinded until initiation of the Open-Label Part B of the study.<br><br>This protocol amendment will add an analysis at the end of Part A. An analysis of safety and immunogenicity data will be performed after all participants have completed Part A of the study. All data collected in Part A of the study will be cleaned (ie, data that are as clean as possible) and locked and a report may be generated.<br><br>An interim analysis of the safety and immunogenicity data in Part B of the study may be performed after participants have completed the OL-Day 29 and/or the OL-Day 57 study procedures. This analysis may be performed when all participants have completed OL-Day 29 and/or OL-Day 57 study procedures, or on a subset of participants who received a single booster dose at OL-Day 1 when they have completed OL-Day29 and/or OL-Day 57 study procedures. All data collected up to OL-D29 and/or OL-D57 in Part B, will be cleaned (ie, data that are as clean as possible) and locked, and a report may be generated.<br><br>An interim analysis of the safety and immunogenicity data in Part C of the study may be performed after participants have completed the OL-D8, OL-D15, OL-Day 29 and/or the OL-Day 57 study procedures.

All data collected up to OL-D29 and/or OL-D57 in Part C, will be cleaned (i.e., data that are as clean as possible) and locked, and a report may be generated.

Additional analyses may be performed to support regulatory requests, as appropriate.

The EOS analysis of all endpoints will be performed after all participants have completed Month 13 (Blinded Part A), or OL-Month 6 and OL-Month 7 (Open-Label Part B), or OL-Month 6 (Part C) study procedures and after the database is cleaned and locked. Results of this analysis will be presented in an EOS CSR, including individual listings.

Date of Protocol Amendment 5: 22 Apr 2021

List of Abbreviations

Abbreviation	Definition
AE	adverse event
ALP	alkaline phosphatase
ALT	alanine aminotransferase
AR	adverse reaction
AST	aspartate aminotransferase
bAb	binding antibody
BMI	body mass index
BUN	blood urea nitrogen
CBC	complete blood count
CBER	Center for Biologics and Evaluation Research
CDC	US Centers for Disease Control and Prevention
CFR	Code of Federal Regulations
CI	confidence interval
CMV	cytomegalovirus
CONSORT	Consolidated Standards of Reporting Trials
CoV	coronavirus
CRO	contract research organization
CSR	clinical study report
DHHS	Department of Health and Human Services
DMID	Division of Microbiology and Infectious Diseases
DSPC	1,2-distearoyl-sn-glycero-3-phosphocholine
eCRF	electronic case report form
EDC	electronic data capture
eDiary	electronic diary
ELISA	enzyme-linked immunoabsorbent assay
EOS	end of study
EUA	Emergency Use Authorization
FAS	full analysis set
FDA	Food and Drug Administration
FSH	follicle-stimulating hormone
GCP	Good Clinical Practice
GLP	Good Laboratory Practice
GM	geometric mean

Abbreviation	Definition
GMFR	geometric mean fold-rise
GMP	Good Manufacturing Practice
GMT	geometric mean titer
HCP	healthcare practitioner
hDPP4	dipeptidyl peptidase 4
HIV	human immunodeficiency virus
hMPV	human metapneumovirus
IB	investigator's brochure
ICF	informed consent form
ICH	International Council for Harmonisation
Ig	immunoglobulin
IM	intramuscular
IRB	institutional review board
IRT	interactive response technology
LLOQ	lower limit of quantification
LNP	lipid nanoparticle
MAAE	medically attended adverse event
MedDRA	Medical Dictionary for Regulatory Activities
MERS-CoV	Middle East Respiratory Syndrome coronavirus
mRNA	messenger RNA
nAb	neutralizing antibody
NIAID	National Institute of Allergy and Infectious Diseases
NOAEL	no adverse effect level
OL	open-label
PCR	polymerase chain reaction
PEG2000-DMG	1-monomethoxypolyethyleneglycol-2,3-dimyristylglycerol with polyethylene glycol of average molecular weight 2000
PIV3	parainfluenza virus type 3
PP	per-protocol
PT	prothrombin time
PTT	partial thromboplastin time
S	spike
S-2P	spike protein with 2 proline residues introduced for stability in a prefusion conformation

Abbreviation	Definition
SAE	serious adverse event
SAP	statistical analysis plan
SARS-CoV	Severe Acute Respiratory Syndrome coronavirus
SM-102	heptadecan-9-yl 8-((2-hydroxyethyl) (6-oxo-6-(undecyloxy)hexyl) amino) octanoate
SMC	Safety Monitoring Committee
SOP	standard operating procedure
TEAE	treatment-emergent adverse event
ULN	upper limit of normal
ULOQ	upper limit of quantification
USP	United States Pharmacopeia
VE	vaccine efficacy
VRC	Vaccine Research Center
WHO	World Health Organization

1 INTRODUCTION

1.1 Background

Coronaviruses (CoVs) are a large family of viruses that cause illness ranging from the common cold to more severe diseases, such as Middle East Respiratory Syndrome (MERS-CoV) and Severe Acute Respiratory Syndrome (SARS-CoV). Coronaviruses are zoonotic, meaning they are transmitted between animals and people.

An outbreak of the CoV disease (COVID-19) caused by SARS-CoV-2 began in Wuhan, Hubei Province, China in December 2019 and has spread throughout China and to over 200 other countries and territories, including the United States (WHO 2020). A CoV ribonucleic acid was quickly identified in some of these patients.

As of 20 Apr 2020, the World Health Organization (WHO) reported more than 2,314,621 confirmed cases and 157,847 deaths globally and have therefore made the assessment that COVID-19 can be characterized as a pandemic (WHO 2020). As of 20 Apr 2020, the US Centers for Disease Control and Prevention (CDC) reported 746,625 confirmed and probable cases of COVID-19 in all 50 states and 5 jurisdictions, with 39,083 attributed and probable deaths (CDC 2020a). The CDC have reported that the highest risk of disease burden is in older adults and populations with certain underlying comorbid conditions such as heart disease, diabetes, and lung disease (CDC 2020b).

There is currently no vaccine against SARS-CoV-2. Global efforts to evaluate novel antivirals and therapeutic strategies to treat severe SARS-CoV-2 infections have intensified, but no proven therapeutic currently exists. Therefore, there is an urgent public health need for rapid development of novel interventions to prevent the spread of this disease.

ModernaTX, Inc. has developed a rapid-response, proprietary vaccine platform based on a messenger RNA (mRNA) delivery system. The platform is based on the principle and observations that cells in vivo can take up mRNA, translate it, and then express protein viral antigen(s) on the cell surface. The delivered mRNA does not enter the cellular nucleus or interact with the genome, is nonreplicating, and is expressed transiently. mRNA vaccines have been used to induce immune responses against infectious pathogens such as cytomegalovirus (CMV) (NCT03382405), human metapneumovirus (hMPV) and parainfluenza virus type 3 (PIV3) (NCT03392389), and influenza virus (NCT03076385 and NCT03345043).

The Sponsor is using its mRNA-based platform to develop a novel lipid nanoparticle (LNP)-encapsulated mRNA-based vaccine against SARS-CoV-2 (mRNA-1273). mRNA-1273 encodes for the full-length spike (S) protein of SARS-CoV-2, modified to introduce 2 proline residues to

stabilize the S protein (S-2P) into a prefusion conformation. The CoV S protein mediates attachment and entry of the virus into host cells (by fusion), making it a primary target for neutralizing antibodies that prevent infection (Johnson et al 2016; Wang et al 2015; Wang et al 2018; Chen et al 2017; Corti et al 2015; Yu et al 2015; Kim et al 2019; Widjaja et al 2019). It has been confirmed that the stabilized SARS-CoV-2 S-2P expresses well and is in the prefusion conformation (Wrapp et al 2020).

Recently, SARS-CoV-2 variants with mutations in the S protein have emerged. A variant first identified in South Africa (B.1.351) is associated with increased transmission, higher viral burden, and possibly increased mortality in infected persons (Tegally et al 2020). To date, four SARS-CoV-2 vaccines, all based on the Wuhan-sequence of the S protein, have shown reduced activity against the B.1.351 variant (Wu et al 2021; Wang et al 2021). Sera from individuals vaccinated with mRNA-based vaccines had a 6-to-9-fold reduction in neutralizing activity against a B.1.351-matched pseudovirion relative to a Wuhan-matched pseudovirion. More recently, pivotal vaccine efficacy studies testing both viral vector and adjuvanted protein vaccines had lower efficacy in regions where B.1.351 was known to be circulating (Callaway and Mallapaty 2021; Cohen 2021).

There is an urgent need for vaccination strategies that induce broader protection that includes variants such as B.1.351 to decrease morbidity and mortality. ModernaTX, Inc. is developing a mRNA vaccine (mRNA-1273.351) that is similar to the mRNA-1273 vaccine available under the Emergency Use Authorization (EUA), but in which the mRNA encodes for the S protein of the B.1.351 variant.

Nonclinical studies have demonstrated that CoV S proteins are immunogenic and S protein-based vaccines, including those based on mRNA delivery platforms, are protective in animals. Prior clinical studies of vaccines targeting related CoVs and other viruses have demonstrated that mRNA-based vaccines are safe and immunogenic. It is therefore anticipated that mRNA-1273 will generate robust immune responses to the SARS-CoV-2 S protein.

The mRNA-1273 vaccine is currently being evaluated for safety and immunogenicity in a dose-ranging Phase 1 study (NCT04283461) sponsored and conducted by the Division of Microbiology and Infectious Diseases (DMID) of the National Institute of Allergy and Infectious Diseases (NIAID). Two dose levels will be chosen for evaluation in this Phase 2 study, based on the data from the Phase 1 DMID study (Section 3.1.2). The development of this vaccine is being accelerated as, if it is demonstrated safe and immunogenic, it may be used to address the current COVID-19 outbreak as a result of the uniquely rapid and scalable manufacturing process for mRNA-1273.

1.2 Nonclinical Studies in Development of mRNA-1273

Nonclinical studies have demonstrated that CoV S proteins are immunogenic and S protein-based vaccines, including those based on mRNA delivery platforms, are protective in animals. Prior clinical studies of vaccines targeting related CoVs and other viruses have demonstrated that mRNA-based vaccines are safe and immunogenic. It is therefore anticipated that mRNA-1273 will generate robust immune responses to the SARS-CoV-2 S protein and will be well tolerated. In addition, mRNA-1273 has shown preliminary evidence of protection against SARS-CoV-2 in a murine model of infection (data on file).

In support of development of mRNA-1273 for prophylaxis against SARS-CoV-2 infection, nonclinical immunogenicity, biodistribution, and safety studies have been completed with similar mRNA-based vaccines formulated in LNPs containing SM-102 (heptadecan-9-yl 8 ((2 hydroxyethyl)(6 oxo 6-(undecyloxy)hexyl)amino)octanoate), the novel proprietary lipid used in the mRNA-1273 LNP formulation. Non-clinical studies with mRNA-1273.351 and the mRNA-1273/mRNA-1273.351 mixture are also planned in mice, Syrian golden hamsters, and non-human primates.

A detailed review of nonclinical experience with mRNA-1273 vaccine is provided in the investigator's brochure (IB).

1.3 Clinical Studies With Lipid Nanoparticle mRNA Vaccines

The mRNA-1273 vaccine is currently being evaluated for safety and immunogenicity in the dose-ranging Phase 1 DMID study of healthy adults at least 18 years of age (Section 3.1.2). The Phase 1 DMID study is an open-label dose-ranging study of mRNA-1273 in healthy adult male and non-pregnant female participants in 3 age groups: age 18 to 55 years, inclusive (45 participants); age 56 to 70 years, inclusive (30 participants); and ≥ 71 years (30 participants). Each participant will receive an intramuscular (IM) injection (0.5 mL) of mRNA-1273 on Days 1 and 29 in the deltoid muscle and will be followed for 13 months after the second injection.

As of 26 October 2020, 120 participants were enrolled. All participants received the first injection of mRNA-1273, and 116 participants received the second injection. Three mRNA-1273 dose levels (25, 100, and 50 µg) administered 28 days apart have been assessed in participants 18 to 55 years, 56 to 70 years, and ≥ 71 years of age. The mRNA-1273 250 µg dose was not evaluated in participants 56 to 70 years and ≥ 71 years of age due to reactogenicity observed in 4 participants in the 250 µg (18 to 55 years) dose cohort. Across all age groups, solicited adverse reactions (ARs) were predominantly mild or moderate in severity and most commonly included fatigue, chills,

headache, myalgia, and pain at the injection site. The incidence and severity of solicited ARs were generally dose dependent and increases in incidence and severity were generally observed after the second injection. The incidence of systemic reactions increased after the second injection, particularly at the highest (250 µg) dose. Four (27%) participants in the 250 µg dose cohort reported at least 1 severe solicited AR after the second injection, including feverishness, fatigue, fever, headache, myalgia, nausea, and erythema/redness. No serious adverse events (SAEs) have been reported through Day 119, and no pause rules were triggered during the study.

This Phase 2a study (mRNA-1273-P201), conducted by the Sponsor under IND 19745, will expand the safety and immunogenicity database by testing 2 two dose levels (50 µg and 100 µg) in 400 adults. As of 05 November 2020, 200 (100%) participants each in the mRNA-1273 50 µg group, mRNA-1273 100 µg group, and placebo group received the first dose, and 195 (97.5%) participants in the mRNA-1273 50 µg group, 198 participants (99.0%) in the mRNA-1273 100 µg group, and 194 (97.0%) participants in the placebo group received the second injection (mRNA-1273-P201). In this study, 2 mRNA-1273 dose levels (50 and 100 µg) or placebo administered 28 days apart were assessed in participants ≥ 18 to < 55 years and ≥ 55 years of age. No protocol-specified pause rules were met during the study. In addition, the study initiated with a sentinel group of 50 participants ≥ 55 years of age; after review of the safety data by the independent SMC, the cohort was expanded to include the remaining 250 participants ≥ 55 years of age.

Additionally, in the Phase 3 study (mRNA-1273-P301) conducted by the Sponsor under IND 19745, as of October 25, 2020, a total of 30,350 participants have been dosed in the Blinded Phase of the study (15,184 received mRNA-1273 and 15,165 received placebo). The protocol-defined interim analysis was conducted in November 2020, which indicated that the primary efficacy endpoint in Study mRNA-1273-P301 was met: mRNA-1273 prevented COVID-19 starting 14 days after the second dose of the mRNA-1273 vaccine, based on a total of 95 adjudicated cases accrued (5 cases in the mRNA-1273 group and 90 cases in the placebo group). The vaccine efficacy (VE) was 94.5% (95% CI: 86.5%, 97.8%; one-sided p value < 0.0001), rejecting the null hypothesis of VE ≤ 30% and achieving the prespecified efficacy boundary based on the 1-sided nominal alpha of 0.0047 using the Lan-DeMets O'Brien-Fleming spending function. The mRNA-1273 vaccine was effective in preventing severe COVID-19, with 11 cases in the placebo group and 0 cases in the mRNA-1273 group. In addition, the vaccine was efficacious in preventing COVID-19 regardless of prior SARS-CoV-2 infection for cases starting 14 days after the second injection (VE of 93.5% based on hazard ratio).

In this study, the safety and reactogenicity of mRNA-1273 100 µg compared with placebo administered 28 days apart were assessed in participants 18 years of age and older at increased risk for acquiring COVID-19 based on occupation or location and living circumstances. Reactogenicity (solicited local and/or systemic ARs) was observed in the majority of participants in the mRNA-1273 group and generally increased after the second injection. The rates of local and systemic ARs were higher in the mRNA-1273 group than in the placebo group after each injection. The majority of solicited ARs in the mRNA-1273 group were grade 1 to grade 2 in severity and generally resolved within 3 days or less. The incidence of unsolicited TEAEs, severe TEAEs, and medically attended adverse events (MAAEs) during the 28 days after injection was also generally similar in participants who received mRNA-1273 and those who received placebo.

Deaths and SAEs were generally reported at a similar incidence in the mRNA-1273 and placebo groups. There was no evidence of enhanced disease, as fewer cases of severe COVID-19 and COVID-19 were observed in participants who received mRNA-1273 than in those who received placebo.

2 STUDY OBJECTIVES

2.1 Primary Objectives (Part A, Blinded)

2.1.1 Primary Safety Objective

The primary safety objective is to evaluate the safety and reactogenicity of 2 dose levels of mRNA-1273 vaccine, each administered in 2 doses 28 days apart.

2.1.2 Primary Immunogenicity Objective

The primary immunogenicity objective is to evaluate the immunogenicity of 2 dose levels of mRNA-1273 vaccine, each administered in 2 doses 28 days apart, as assessed by the level of specific binding antibody (bAb).

2.2 Secondary Objective (Part A, Blinded)

The secondary objective is to evaluate the immunogenicity of 2 dose levels of mRNA-1273 vaccine, each administered in 2 doses 28 days apart, as assessed by the titer of neutralizing antibody (nAb).

2.3 Exploratory Objectives (Part A, Blinded)

The exploratory objectives are the following:

- To profile S protein-specific serum immunoglobulin (Ig) class and subclass and nAb in serum
- To describe the ratio or profile of specific bAb relative to nAb in serum
- To describe initial immunogenicity responses following the first dose (Day 1) and prior to the second dose (Day 29)
- To characterize the clinical profile and immune response of participants infected by SARS-CoV-2
- To evaluate the effect of the mRNA-1273 vaccine on the incidence of SARS-CoV-2 infection

2.4 Primary Objectives (Part B, Open Label)

2.4.1 Primary Safety Objective

The primary safety objective is to evaluate the safety and reactogenicity of 50 µg of mRNA-1273 vaccine administered as a single booster dose or 100 µg of mRNA-1273 administered as 2 doses 28 days apart.

2.4.2 Primary Immunogenicity Objective

The primary immunogenicity objective is to evaluate the immunogenicity of 50 µg of mRNA-1273 vaccine administered as a single booster dose or 100 µg of mRNA-1273 administered as 2 doses 28 days apart, as assessed by the level of specific bAb.

2.5 Secondary Objective (Part B, Open Label)

The secondary objective is to evaluate the immunogenicity of 50 µg of mRNA-1273 vaccine administered as a single booster dose or 100 µg of mRNA-1273 100µg administered as 2 doses 28 days apart, as assessed by the titer of nAb.

2.6 Exploratory Objectives (Part B, Open Label)

The exploratory objectives are the following:

- To profile S protein-specific serum Ig class and subclass and nAb in serum
- To describe the ratio or profile of specific bAb relative to nAb in serum
- To describe initial immunogenicity responses at various time points following a booster dose, where applicable
- To characterize the clinical profile and immune response of participants infected by SARS-CoV-2
- To evaluate the effect of the mRNA-1273 vaccine on the incidence of SARS-CoV-2 infection

2.7 Primary Objectives (Part C, Open Label)

2.7.1 Primary Safety Objective

The primary safety objective is to evaluate the safety and reactogenicity of 2 dose levels (20 and 50 µg) of mRNA-1273.351 or one dose level of a mixture of 25 µg mRNA-1273 and 25 µg mRNA-1273.351 (mRNA-1273/mRNA-1273.351 mixture) (50 µg total) vaccine administered as a single booster dose after completing a two-dose vaccination schedule of mRNA-1273 at least 6 months earlier.

2.7.2 Primary Immunogenicity Objective

The primary immunogenicity objective is to evaluate the immunogenicity of 2 dose levels (20 and 50 µg) of mRNA-1273.351 or one dose level of mRNA-1273/mRNA-1273.351 mixture (50 µg total) vaccine administered as a single booster dose after completing a two-dose vaccination schedule of mRNA-1273 at least 6 months earlier, as assessed by the level of specific nAb directed against the S2P antigen derived from B.1.351 variant.

2.8 Secondary Immunogenicity Objective (Part C, Open Label)

The secondary immunogenicity objective is to evaluate the immunogenicity of 2 dose levels (20 and 50 µg) of mRNA-1273.351 or one dose level of mRNA-1273/mRNA-1273.351 mixture (50 µg total) vaccine administered as a single booster dose after completing a two-dose vaccination schedule of mRNA-1273 at least 6 months earlier, as assessed by the level of specific bAb directed against the S2P antigen derived from B.1.351 variant.

2.9 Exploratory Objectives (Part C, Open Label)

The exploratory objectives are the following:

- To profile S protein-specific serum Ig class and subclass and nAb in serum
- To describe the ratio or profile of specific bAb relative to nAb in serum
- To describe initial immunogenicity responses at various time points for 6 months following the first and second booster dose, as applicable
- To characterize the clinical profile and immune response of participants infected by SARS-CoV-2
- To evaluate the effect of the mRNA-1273.351 and mRNA-1273/mRNA-1273.351 mixture vaccines on the incidence of SARS-CoV-2 infection
- To further assess the immune response to vaccines containing the B.1.351 and the D614G variant sequences

3 INVESTIGATIONAL PLAN

3.1 General Study Design

This is a three-part, Phase 2a study: Part A, Part B, and Part C. Participants in Part A, the Blinded Phase of the study, are blinded to their treatment assignment. Given that the primary efficacy endpoint for mRNA-1273 against COVID-19 was met in a separate Phase 3 efficacy study (COVE study) conducted by the Sponsor, this Phase 2a study will move to Part B, Open Label Interventional Phase.

Part B, the Open-label Interventional Phase of this study is designed to offer participants who received placebo in Part A of this study an option to receive 2 injections of open-label mRNA-1273 (Figure 3). Participants who received 1 or 2 injections of mRNA-1273 (50 µg or 100 µg) in Part A of this study will proceed to Part B, open label, and will be offered a single booster dose of mRNA-1273 (50 µg). This study will be conducted in compliance with the protocol, Good Clinical Practice (GCP), and all applicable regulatory requirements.

Part C will be a proof-of-concept rollover study of approximately 60 participants, who are currently enrolled in Moderna's Phase 3 mRNA-1273-P301 COVE study and have previously received 2 doses of mRNA-1273 at least 6 months earlier. Upon enrollment into Part C of this study, they will receive a single IM injection of mRNA-1273.351 (20 µg or 50 µg) or mRNA-1273/mRNA-1273.351 mixture (50 µg total) at least 6 months after receiving the second vaccination in the mRNA-1273-P301 COVE study. At enrollment into this study, their participation in mRNA-1273-P301 COVE study will be terminated.

3.1.1 Part A, the Blinded Phase

The Blinded Phase of this study is a randomized, observer-blind, and placebo-controlled, with adult participants at least 18 years of age. The study schematic is presented in Figure 1 and the Schedule of Events is presented in Table 9.

Two dose levels, 50 µg and 100 µg, will be evaluated in this study, based in part on initial safety data from the Phase 1 DMID study of mRNA-1273. The study will include 2 age cohorts: Cohort 1 with 300 participants (≥ 18 to < 55 years old) and Cohort 2 with 300 participants (≥ 55 years old). Approximately 600 participants will receive either mRNA-1273 vaccine or saline placebo control according to a 1:1:1 randomization ratio; ie, within each age cohort, 100 participants will receive mRNA-1273 50 µg, 100 participants will receive mRNA-1273 100 µg, and 100 participants will receive saline placebo (Figure 1).

The study will be initiated with a parallel enrollment of all 300 participants in Cohort 1 (≥ 18 to < 55 years old) and a sentinel group of 50 participants in Cohort 2 (≥ 55 years old) receiving study treatment (Figure 2). Before initiating study treatment of the remaining participants in Cohort 2, safety data through Day 7 from the sentinel group of Cohort 2 and all available data from Cohort 1 will be reviewed by the Safety Monitoring Committee (SMC; Section 6.1.1).

If no safety concerns are found, expansion enrollment (N=250) of Cohort 2 will proceed.

Figure 1: Study Flow Schema (Part A, Blinded Phase)

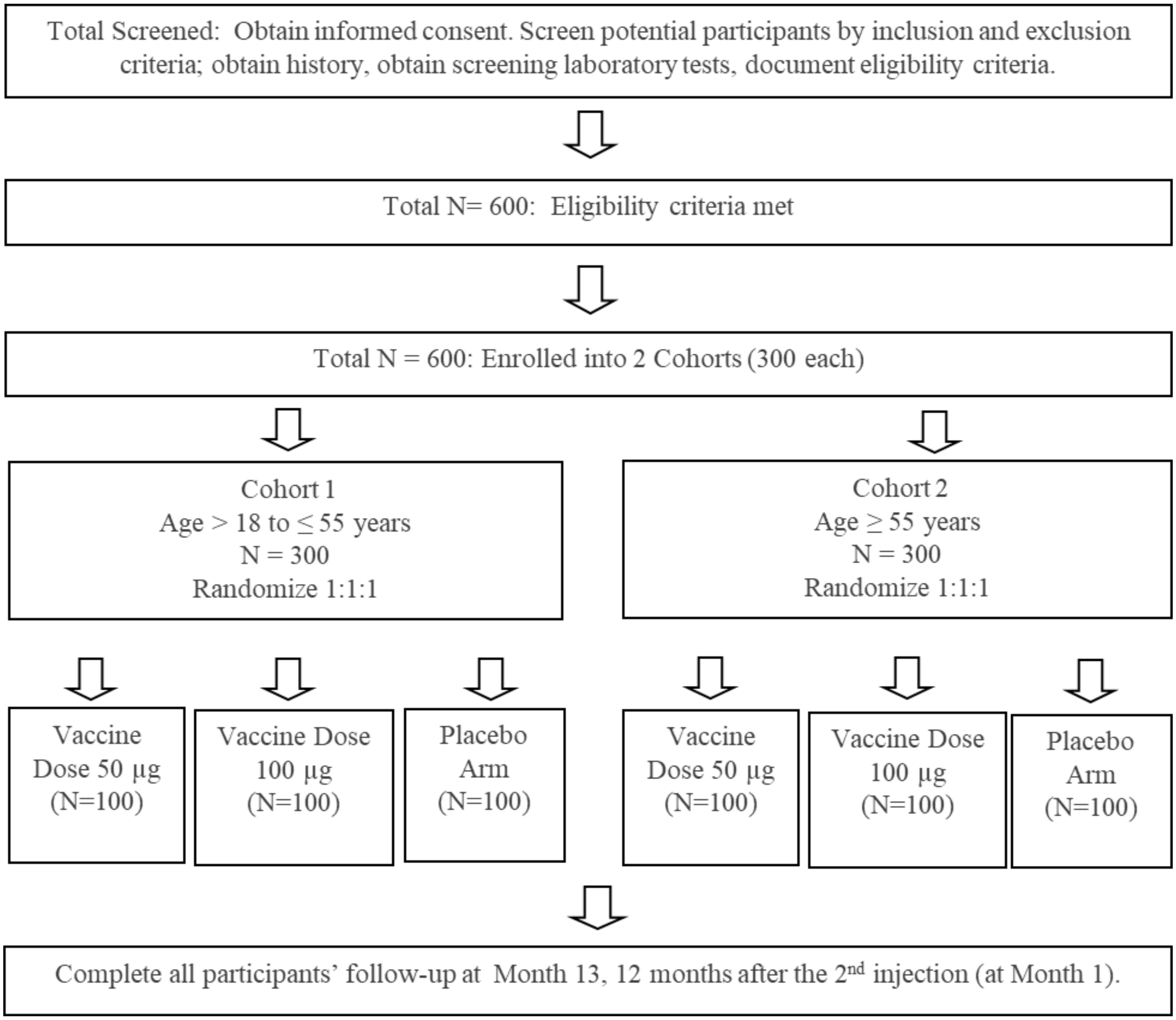

Figure 2: Sentinel and Expansion Cohort Schema (Part A, Blinded Phase)

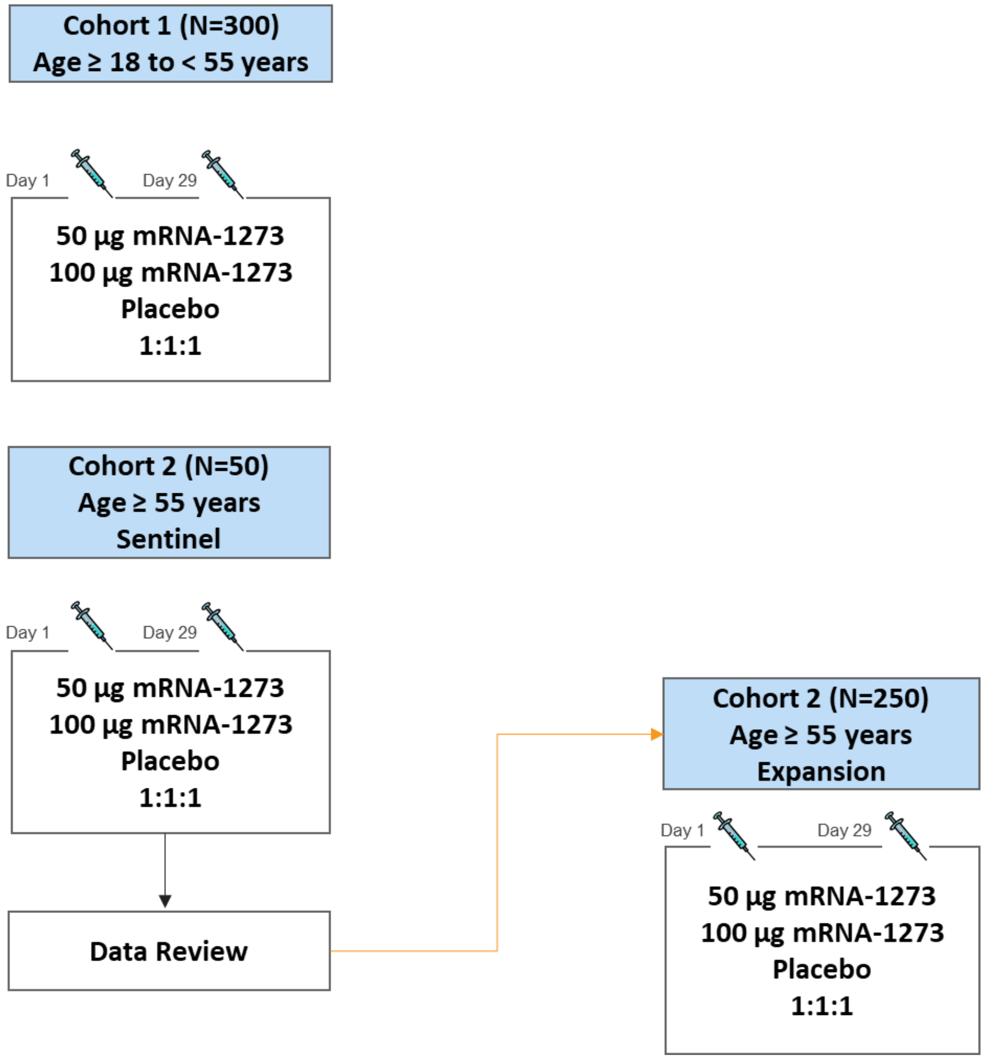

Part A, Blinded Phase of the study comprises up to 10 scheduled study site visits: Screening, Day 1, Day 8, Day 15, Day 29 (Month 1), Day 36, Day 43, Day 57 (Month 2), Day 209 (Month 7), and Day 394 (Month 13) or initiation of Part B, whichever is earlier. There are also scheduled participant contacts approximately every 2 weeks after Day 57 to collect MAAEs, AEs leading to withdrawal, SAEs, concomitant medications associated with these events, receipt of non-study vaccinations, exposure to someone with known COVID19 or SARS-CoV-2 infection, and participant experience of COVID-19 symptoms (Table 9). Every 4 weeks from Day 71 through Day 183 and from Day 223 through Day 363, each participant will complete a questionnaire in an electronic diary (eDiary) that will be reviewed by study site personnel. Safety telephone calls will occur every 4 weeks from Day 85 through Day 197 and from Day 237 through Day 377. The study duration will be approximately 14 months for each participant: a screening period of up to 1 month

and a study period of 13 months that includes the first dose of vaccine on Day 1 and the second dose on Day 29. The participant's final visit will be on Day 394 (Month 13), 12 months after the second dose of vaccine on Day 29 (Month 1), or at the initiation of Part B, whichever is earlier.

To test for the presence of SARS-CoV-2, nasopharyngeal swab samples will be collected at Day 1, Day 29, and Day 57. During the course of the study, participants meeting pre-specified disease criteria that suggest possible SARS-CoV-2 infection will be asked to contact the study site to arrange for a prompt, thorough, and careful assessment (Section 3.4.1).

Each participant will receive 2 injections of mRNA-1273 or placebo by 0.5 mL IM injection on Day 1 and Day 29. Vaccine accountability, dose preparation, and vaccine administration will be performed by unblinded pharmacy personnel who will not participate in any other aspects of the study during Part A, the Blinded Phase. The remainder of the study staff, all participants, and Sponsor personnel (or its designees) will remain blinded to dosing assignment (Section 3.3.5).

All participants will be followed for safety and reactogenicity and provide pre- and post-injection blood specimens for immunogenicity through 12 months after the last dose of investigational product. Section 4.7 describes the planned study analyses.

The end of Part A is defined as the earlier of Visit 9, the completion of the last participant's last visit, or initiation of Part B. Participants are considered to have completed Part A of the study if they complete the final visit on Day 394 (Month 13) or initiated Part B of the study.

The end of study (EOS) is defined as date of last visit of Part A, Part B or Part C of the study, whichever occurs later. Participants are considered to have completed the study if they complete the final visit on Day 394 (Month 13), 12 months after the second injection on Day 29 (Month 1).

At each dosing visit, participants will be instructed (Day 1) or reminded (Day 29) how to document and report solicited ARs within a provided eDiary. Solicited ARs will be assessed for 7 days (the day of injection and the following 6 days) after each injection and unsolicited AEs will be assessed for 28 days after each injection; SAEs and MAAEs will be assessed throughout the study.

Participants will have blood sampled at scheduled study visits during the study for safety and immunogenicity assessments or other medical concerns, according to the investigator's judgment. In addition, participants may have blood sampled at unscheduled visits for acute respiratory symptoms.

Detailed information on all statistical analysis of data is presented in Section 4.6.2.

3.1.2 Part B, Open-Label Interventional Phase

Part B, the Open-Label Interventional Phase of the study, is prompted by the authorization of a COVID-19 vaccine under an EUA. Transitioning the study to Part B permits all ongoing study participants to be informed of the availability and eligibility criteria of any COVID-19 vaccine made available under an EUA and the option to offer all ongoing study participants an opportunity to schedule a Participant Decision Visit to know their original treatment assignment (placebo vs. mRNA-1273 [50 µg or 100 µg] vaccine).

Part B, the Open-Label Interventional Phase of the study, provides the opportunity for study participants who previously received placebo to actively request to receive 2 doses of mRNA-1273 (100 µg) vaccine. In addition, all participants who previously received 1 or 2 injections of mRNA-1273 (50 µg or 100 µg) vaccine will be able to receive a single booster dose of mRNA-1273 (50 µg). All study participants will receive a Notification Letter summarizing the basis for a COVID-19 vaccine to receive an EUA and will be asked to schedule a Participant Decision Clinic Visit.

Figure 3: Part B, Open-Label Schema

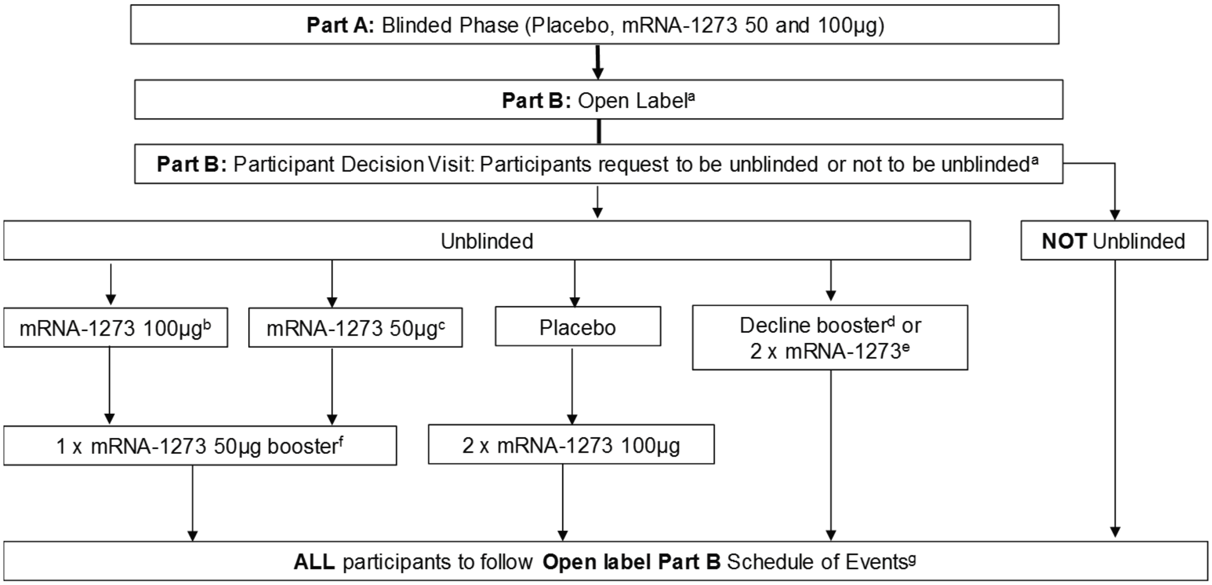

[a] All participants will proceed to Part B, Open-Label Interventional Phase; begins with the Participant Decision Visit.
[b] Participants who received 2 injections of mRNA-1273 100 µg during Blinded Part A.
[c] Participants who received 2 injections of mRNA-1273 50 µg during Blinded Part A.
[d] Participants who received 2 injections of mRNA-1273 in Part A and decline booster injection in Part B.
[e] Participants who received placebo in Part A and decline 2 injections of mRNA-1273 in Part B.
[f] Participants who received 2 injections of mRNA-1273 (50 µg or 100 µg) in Part A will receive one booster injection of mRNA-1273 50 µg in Part B.
[g] See Table 10 for directions for Part B schedule of events based on Participant status at end of Participant Decision Visit.

At the Participant Decision Clinic Visit, all participants will:

- Be given the option to be unblinded as to their original group assignment (placebo vs. mRNA-1273 vaccine [50 µg or 100 µg]),
- Be counselled about the importance of continuing other public health measures to limit the spread of disease including social distancing, wearing a mask, and hand-washing,
- Sign a revised ICF, and
- Provide a nasopharyngeal (NP) swab for RT-PCR for SARS-CoV-2 and a blood sample for serology and immunogenicity.

After the Participant Decision Clinic Visit (Table 10), all participants will proceed to the open-label Part B of the study and will follow the Part B Schedule of Events (Table 11 or Table 12) as follows (Figure 3):

- **Participants received placebo in Part A and consent to unblinding and to receiving 2 doses of mRNA-1273 in Part B**: These participants will proceed to Part B and follow the schedule of events (SOE) in Table 11.
- **Participants received 1 dose of mRNA-1273 in Part A (regardless of the initial dose level received) and consent to unblinding and to receiving another booster injection of mRNA-1273 in Part B**: These participants will proceed to Part B and follow the SOE in Table 12.
- **Participants received 2 doses of mRNA-1273 in Part A and consent to unblinding and to receiving another booster injection of mRNA-1273 dose in Part B:** These participants will proceed to Part B and follow the SOE in Table 12 (note that these participants will receive 3 injections of mRNA-1273 during the course of the study).
- **Participants received 2 doses of mRNA-1273 in Part A and consent to unblinding, but decline receiving another booster injection of mRNA-1273 in Part B:** These participants will proceed to Part B and follow the schedule of events (SOE) in Table 12.
- **Received 1 dose of blinded study medication in Part A and decline unblinding:** These participants will proceed to Part B and follow the SOE in Table 12.
- **Participants decline unblinding:** These participants will proceed to Part B and follow the SOE in Table 12.

  Note: **After participant proceeds to the open-label Part B of the study at the Participant Decision Clinic Visit (Table 10), Part A schedule of events (Table 9) will no longer be followed and will become obsolete.**

3.1.3 Part C, Open-Label Interventional Phase of mRNA-1273.351 and mRNA-1273/mRNA-1273.351 Mixture Booster Vaccines

Part C, the Open-Label Interventional Phase of the study of mRNA-1273.351 and mRNA-1273/mRNA-1273.351 mixture booster vaccines, is prompted by the need to proactively prepare for vaccination strategies that induce a broader protection, including variants such as B.1.351. This part will be a proof-of-concept rollover design, where participants in Moderna's Phase 3 mRNA-1273-P301 COVE study, who have previously received 2 doses of mRNA-1273 at least 6 months previously, will be enrolled into Part C of this study. Upon consent to roll over, participants in Part C will receive a single booster dose of either mRNA-1273.351 (20 µg or 50 µg), or mRNA-1273/mRNA-1273.351 mixture (50 µg total). An additional booster dose may be added approximately 56 days after the first boost at OL-D1. This additional booster dose will be triggered following review of immunogenicity data up to OL-D15 of the initial mRNA-1273.351 (20 µg and 50 µg) and mRNA-1273/mRNA-1273.351 mixture injections. The enrollment and vaccination in each study arm will be sequential. The first 20 participants will be enrolled and dosed on OL-D1 with the mRNA-1273.351 50 µg dose. Upon completion of the first 50 µg dose arm, 20 participants will be enrolled in the second arm and will receive the mRNA-1273/mRNA-1273.351 mixture (50 µg total dose). Following completion of the second arm, 20 participants will be enrolled and dosed with the mRNA-1273.351 20 µg dose.

Figure 4: Part C, Open-Label Schema for Boosting with mRNA-1273.351 and mRNA-1273/mRNA-1273.351 Mixture

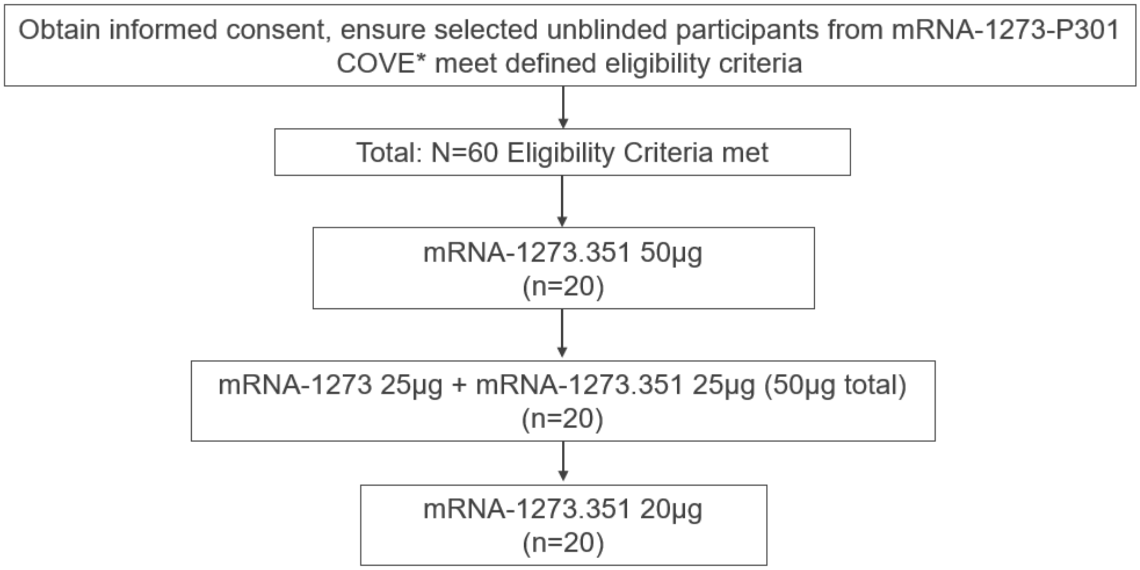

Note: *Only COVE study participants who are unblinded and have received 2 doses of mRNA-1273 at least 6 months earlier; in addition, the participants will have to meet all other specified eligibility criteria to be rolled over into Part C of the mRNA-1273-P201 study.

3.1.4 Rationale for Dose Selection

In the Blinded Part A of this study, the 2 dose levels of mRNA-1273 tested in participants will be 50 µg and 100 µg, based on assessment of available safety and immunogenicity data from the Phase 1 DMID study and Phase 1 studies of mRNA-1647 and mRNA-1443 (Section 1).

The Phase 1 DMID study, an open-label dose-ranging study of mRNA-1273 in healthy adult male and non-pregnant female participants in 3 age groups: age 18 to 55 years, inclusive (45 participants); age 56 to 70 years, inclusive (30 participants); and ≥ 71 years (30 participants) is currently ongoing. Participants in each age cohort will be randomly assigned to 1 of 3 dose levels of mRNA-1273: 25 µg, 100 µg, and 250 µg. Each participant will receive an IM injection (0.5 mL) of mRNA-1273 on Days 1 and 29 in the deltoid muscle and will be followed for 12 months after the second injection.

As of 14 May 2020, 15 participants in each of the 3 dose levels of the 18 to 55-year age cohort received at least 1 dose of mRNA-1273. Recruitment of participants in the 2 older age cohorts is ongoing. There have been no SAEs and no triggering of study pause rules. A review of preliminary solicited local and systemic ARs in participants in the 18 to 55-year age cohort after the second injection showed 3 participants in the 100 µg dose group who reported severe local ARs (Grade 3 erythema and induration) and 3 participants in the 250 µg dose group who reported severe systemic ARs (fever, fatigue, feverishness, myalgia, and nausea). These ARs resolved within 24 hours and were not assessed as serious.

The 50 µg and 100 µg doses proposed for this Phase 2a study fall within the doses being evaluated in the Phase 1 DMID study. Specifically, in Part B of the amended study, the decision to provide a booster dose to those participants who received two doses of 50 or 100 µg is, in part, to address the waning neutralizing antibody titers seen in the Phase 1 DMID study beginning 4 months after the first vaccination (Widge et al 2021). Furthermore, the choice of a 50 µg dose level as a booster is supported by data from Phase 2 studies indicating that the immune response is largely indistinguishable between 50 and 100 µg.

In Part C of the amended study, participants will receive a single booster dose of either mRNA-1273.351 (20 µg or 50 µg) or mRNA-1273/mRNA-1273.351 mixture (50 µg total; 25 µg of mRNA-1273 and 25 µg of mRNA-1273.351). The dose ranging will help assess the potential for cross-priming by the variant vaccine. In addition, the inclusion of the choice of the 20-µg dose level as a booster is intended to help assess the stability of the 50 µg dose of the vaccine at the limits of the anticipated expiration.

3.1.5 Rationale for Study Design

The 2 age cohorts in this Phase 2a study, ≥ 18 to < 55 years old and ≥ 55 years old, were established to better understand the relationships among dose, tolerability, and immunogenicity in different age groups, one being healthy older adults. The older cohort in this Phase 2a study corresponds to the 2 older age cohorts in the Phase 1 DMID study.

Because there are currently no licensed SARS-CoV-2 vaccines available, 0.9% sodium chloride will be used as a placebo control for the safety and immunogenicity assessments. Consequently, the mRNA-1273 vaccine and placebo injections will look different, so administration will be blinded in Part A of the study (Section 3.3.5).

The Phase 1 DMID study is small (105 participants at 3 dose levels) and does not incorporate a placebo. Having a sample size of 600 participants in this Phase 2a study and including a placebo will help to improve understanding of AEs.

With SARS-CoV-2 expected to be circulating in the general population during the study, all participants will provide pre-injection blood samples and post-injection blood samples for antibody analysis through 12 months after the last dose of investigational product. In addition, participants will have nasopharyngeal swab samples collected at Day 1 and Day 29, before the injections, and at Day 57. Furthermore, with any signs or symptoms or MAAE suggesting SARS-CoV-2 infection in a participant, an additional nasopharyngeal swab sample and a blood sample will be taken to confirm the diagnosis of SARS-CoV-2 via serology and polymerase chain reaction (PCR). Additionally, clinical information will be carefully collected to evaluate the severity of the clinical case.

Since it is possible that participants are naturally exposed to SARS-CoV-2 through community exposure, the nasopharyngeal swab samples collected before study injection may help discriminate between natural infection and vaccine-induced antibody responses, should such discrimination be needed.

Following authorization of a COVID-19 vaccine under EUA, this study amendment is designed to transition to Part B, the Open-Label Interventional Phase (Figure 3). Transitioning the study to Part B, Open-Label Interventional Phase, permits all ongoing study participants to (a) be informed of the availability and eligibility criteria of any COVID-19 vaccine made available under an EUA and (b) the option to offer all ongoing study participants who request unblinding an opportunity to schedule a study visit to know their original group assignment (placebo vs. mRNA-1273 [50 µg or 100 µg vaccine]). Part B, Open-Label Interventional Phase, also provides the opportunity for study participants who previously received placebo to request to receive 2 doses of mRNA-1273 (100 µg) vaccine. Participants who originally received mRNA-1273

(50 µg or 100 µg vaccine) will have the opportunity to request to receive a single booster dose of mRNA-1273 (50 µg).

As SARS-CoV-2 variants with mutations in the S protein have emerged, we are learning about the clinical significance to ongoing global vaccination efforts based on strains derived from the Wuhan virus. A variant first identified in South Africa (B.1.351) is associated with increased transmission, higher viral burden, and possibly increased mortality in infected persons (Tegally et al 2020). The development and testing of vaccines targeting this SARS-CoV-2 variant is urgently needed.

mRNA-1273.351, like mRNA-1273, encodes the prefusion stabilized S protein of SARS-CoV-2. However, the mRNA of mRNA-1273.351 incorporates the key mutations present in the B.1.351 strain of the virus. Part C of this study will evaluate the immunological benefit of boosting participants previously vaccinated with mRNA-1273 (mRNA-1273-P301 COVE study) with the B.1.351 strain-specific S protein (mRNA-1273.351) and the mRNA-1273/mRNA-1273.351 mixture, which incorporates 25 µg of each of the Wuhan -1 and B.1.315 representative sequences, for a total dose of 50 µg.

Healthy male or female participants will be enrolled at study sites in the US or its territories.

3.1.6 Inclusion Criteria

Each participant must meet all of the following criteria during the screening period and at Day 1/OL-D1, unless noted otherwise, to be enrolled in this study:

Male or female, 18 years of age or older at the time of consent (Screening Visit, Day 0). For **Part B**, participants must have been previously enrolled in the mRNA-1273 P201 study. For **Part C**, participants must have been previously enrolled in the mRNA-1273-P301 COVE study, must have received two doses of mRNA-1273 in Part A of that study (ie, already unblinded and aware of their actual treatment), with their second dose at least 6 months prior to enrollment in this part, and must be currently enrolled and compliant in that study (ie, not have withdrawn or discontinued early).

Understands and agrees to comply with the study procedures and provides written informed consent.

According to the assessment of the investigator, is in good general health and can comply with study procedures.

3.1.6.1 Inclusion criterion specific for Parts A and B:

1. Female participants of nonchildbearing potential may be enrolled in the study. Nonchildbearing potential is defined as surgically sterile (history of bilateral tubal ligation, bilateral oophorectomy, hysterectomy) or postmenopausal (defined as amenorrhea for ≥ 12 consecutive months prior to Screening (Day 0) without an alternative medical cause). A follicle-stimulating hormone (FSH) level may be measured at the discretion of the investigator to confirm postmenopausal status.
2. Female participants of childbearing potential may be enrolled in the study if the participant fulfills all the following criteria:
    - Has a negative pregnancy test at Screening (Day 0) and on the day of the first injection (Day 1).
    - Has practiced adequate contraception or has abstained from all activities that could result in pregnancy for at least 28 days prior to the first injection (Day 1).
    - Has agreed to continue adequate contraception through 3 months following the second injection (Day 29).
    - Is not currently breastfeeding.

    Adequate female contraception is defined as consistent and correct use of a Food and Drug Administration (FDA) approved contraceptive method in accordance with the product label. For example:

    - Barrier method (such as condoms, diaphragm, or cervical cap) used in conjunction with spermicide
    - Intrauterine device
    - Prescription hormonal contraceptive taken or administered via oral (pill), transdermal (patch), subdermal, or IM route
    - Sterilization of a female participant’s monogamous male partner prior to entry into the study

    Note: periodic abstinence (eg, calendar, ovulation, symptothermal, post-ovulation methods) and withdrawal are not acceptable methods of contraception.
3. Male participants engaging in activity that could result in pregnancy of sexual partners must agree to practice adequate contraception and refrain from sperm donation from the time of the first injection and through 3 months after the last injection.

Adequate contraception for male participants is defined as:

- Monogamous relationship with a female partner using an intrauterine device or hormonal contraception (described above)
- Use of barrier methods and spermicide
- History of surgical sterilization
- Male participants with partners who have become pregnant prior to Screening are eligible to participate in the study.

3.1.6.2 Inclusion criterion specific for Part C:

1. Female participants of nonchildbearing potential may be enrolled in the study. Nonchildbearing potential is defined as surgically sterile (history of bilateral tubal ligation, bilateral oophorectomy, hysterectomy) or postmenopausal (defined as amenorrhea for ≥ 12 consecutive months prior to Screening (Day 0) without an alternative medical cause). The FSH level may be measured at the discretion of the investigator to confirm postmenopausal status.

2. Female participants of childbearing potential may be enrolled in the study if the participant fulfills all the following criteria:

   - Has a negative pregnancy test at Part C OL-Day 1 and on the day of injection.
   - Has practiced adequate contraception or has abstained from all activities that could result in pregnancy for at least 28 days prior to the injection (OL-Day 1).
   - Has agreed to continue adequate contraception through 3 months following the second injection (OL-Day 57), as applicable.
   - Is not currently breastfeeding.

3. Adequate female contraception is defined as consistent and correct use of a Food and Drug Administration (FDA) approved contraceptive method in accordance with the product label. For example:

   - Barrier method (such as condoms, diaphragm, or cervical cap) used in conjunction with spermicide
   - Intrauterine device

    - Prescription hormonal contraceptive taken or administered via oral (pill), transdermal (patch), subdermal, or IM route
    - Sterilization of a female participant's monogamous male partner prior to entry into the study
  - Note: periodic abstinence (eg, calendar, ovulation, symptothermal, post-ovulation methods) and withdrawal are not acceptable methods of contraception.

4. Adequate contraception for male participants is defined as:
    - Monogamous relationship with a female partner using an intrauterine device or hormonal contraception (described above)
    - Use of barrier methods and spermicide
    - History of surgical sterilization
    - Male participants with partners who have become pregnant prior to Screening are eligible to participate in the study.
5. Healthy adults or adults with pre-existing medical conditions who are in stable condition. A stable medical condition is defined as disease not requiring significant change in therapy or hospitalization for worsening disease during the 3 months before enrollment.
6. For Part C, participants must have provided SARS-CoV 2 serology samples at Day 1, Day 29, Day 57, and at the Participant Decision Visit (OL-D1) in Moderna's COVE study.

3.1.7 Exclusion Criteria

3.1.7.1 Exclusion Criteria (Part A and Part B)

Participants meeting any of the following criteria for Parts A and B at the Screening Visit (Day 0) or at Day 1, unless noted otherwise, will be excluded from the study:

1. Pregnant or breastfeeding.
2. Is acutely ill or febrile 24 hours prior to or at the Screening Visit (Day 0). Fever is defined as a body temperature ≥ 38.0°C/100.4°F. Participants meeting this criterion may be rescheduled within the relevant window periods. Afebrile participants with minor illnesses can be enrolled at the discretion of the investigator.
3. Current treatment with investigational agents for prophylaxis against COVID-19.

4. Has a medical, psychiatric, or occupational condition that may pose additional risk as a result of participation, or that could interfere with safety assessments or interpretation of results according to the investigator's judgment.
5. Is a healthcare worker or a member of an emergency response team.
6. Current use of any inhaled substance (eg, tobacco or cannabis smoke, nicotine vapors).
7. History of chronic smoking (≥ 1 cigarette a day) within 1 year of the Screening Visit (Day 0).
8. History of illegal substance use or alcohol abuse within the past 2 years. This exclusion does not apply to historical cannabis use that was formerly illegal in the participant's state but is legal at the time of Screening.
9. Known history of hypertension, or systolic blood pressure > 150 mm Hg in participants in Cohort 1 (≥ 18 to < 55 years old) or systolic blood pressure > 160 mm Hg in participants in Cohort 2 (≥ 55 years old) at the Screening Visit (Day 0).
10. Known history of hypotension or systolic blood pressure < 85 mm Hg at the Screening Visit (Day 0).
11. Diabetes mellitus.
12. Diagnosis of chronic pulmonary disease (eg, chronic obstructive pulmonary disease, asthma).
13. Chronic cardiovascular disease.
14. Resides in a nursing home.
15. Grade 1 or higher toxicity on clinical safety laboratory testing at the Screening Visit (Day 0).
16. Current or previous diagnosis of immunocompromising condition, immune-mediated disease, or other immunosuppressive condition.
17. Received systemic immunosuppressants or immune-modifying drugs for > 14 days in total within 6 months prior to the Screening Visit (Day 0) (for corticosteroids ≥ 20 mg/day of prednisone equivalent). Topical tacrolimus is allowed if not used within 14 days prior to the Screening Visit (Day 0).
18. Anticipating the need for immunosuppressive treatment at any time during participation in the study.

19. Positive serology for hepatitis B virus surface antigen, hepatitis C virus antibody, or human immunodeficiency virus (HIV) type 1 or 2 antibodies identified at the Screening Visit (Day 0).
20. History of anaphylaxis, urticaria, or other significant AR requiring medical intervention after receipt of a vaccine.
21. Bleeding disorder considered a contraindication to IM injection or phlebotomy.
22. Diagnosis of malignancy within previous 10 years (excluding non-melanoma skin cancer).
23. Has received or plans to receive a licensed vaccine ≤ 28 days prior to the first injection (Day 1) or plans to receive a licensed vaccine within 28 days before or after any study injection. Licensed influenza vaccines may be received more than 14 days before or after any study injection.
24. Receipt of systemic immunoglobulins or blood products within 3 months prior to the Screening Visit (Day 0) or plans for receipt during the study.
25. Has donated ≥ 450 mL of blood products within 28 days prior to the Screening Visit (Day 0) or plans to donate blood products during the study.
26. Participated in an interventional clinical study, other than mRNA-1273-P301 COVE, within 28 days prior to the Screening Visit (Day 0) or plans to do so while participating in this study.
27. Is an immediate family member or household member of study personnel.

3.1.7.2 Exclusion Criteria (Part C)

Participants meeting any of the following criteria for Part C at OL-D1, unless noted otherwise, will be excluded from the study:

1. Is acutely ill or febrile 24 hours prior to or at the Screening Visit (Day 0). Fever is defined as a body temperature ≥ 38.0°C/100.4°F. Participants meeting this criterion may be rescheduled within the relevant window periods. Afebrile participants with minor illnesses can be enrolled at the discretion of the investigator.
2. Current treatment with investigational agents for prophylaxis against COVID-19.
3. Has a medical, psychiatric, or occupational condition that may pose additional risk as a result of participation, or that could interfere with safety assessments or interpretation of results according to the investigator’s judgment.

4. History of illegal substance use or alcohol abuse within the past 2 years. This exclusion does not apply to historical cannabis use that was formerly illegal in the participant's state but is legal at the time of Screening.
5. Known history of hypotension or systolic blood pressure < 85 mm Hg at the Screening Visit (Day 0).
6. Current or previous diagnosis of immunocompromising condition, immune-mediated disease, or other immunosuppressive condition.
7. Received systemic immunosuppressants or immune-modifying drugs for > 14 days in total within 6 months prior to the Screening Visit (Day 0) (for corticosteroids ≥ 20 mg/day of prednisone equivalent). Topical tacrolimus is allowed if not used within 14 days prior to the Screening Visit (Day 0).
8. Anticipating the need for immunosuppressive treatment at any time during participation in the study.
9. History of anaphylaxis, urticaria, or other significant AR requiring medical intervention after receipt of a vaccine.
10. Bleeding disorder considered a contraindication to IM injection or phlebotomy.
11. Has received or plans to receive a licensed vaccine ≤ 28 days prior to the first injection (Day 1) or plans to receive a licensed vaccine within 28 days before or after any study injection. Licensed influenza vaccines may be received more than 14 days before or after any study injection.
12. Receipt of systemic immunoglobulins or blood products within 3 months prior to the Screening Visit (Day 0) or plans for receipt during the study.
13. Has donated ≥ 450 mL of blood products within 28 days prior to the Screening Visit (Day 0) or plans to donate blood products during the study.
14. Participated in an interventional clinical study, other than mRNA-1273-P301 COVE, within 28 days prior to the Screening Visit (Day 0) or plans to do so while participating in this study.
15. Is an immediate family member or household member of study personnel.
16. Is SARS-CoV-2 positive by RT-PCR (central or local testing) at baseline or at any time during the mRNA-1273-P301 COVE study regardless of the presence or absence of symptoms consistent with COVID-19.
17. Had any SAE in the mRNA-1273-P301 COVE study.

3.1.8 Screen Failures (Part A: Blinded Phase Only)

Screen failures are defined as participants who consent to participate in the study but are not subsequently randomly assigned to study intervention. A minimal set of screen failure information is required to ensure transparent reporting of screen failure participants to meet the Consolidated Standards of Reporting Trials (CONSORT) publishing requirements and to respond to queries from regulatory authorities. Minimal information includes demography, screen failure details, eligibility criteria, and any SAE. In the event an eligible participant was not enrolled as a result of a cohort being full and the participant having surpassed their 28-day screening period, the investigator may rescreen the participant for enrollment by assigning the participant a new identification number and repeating all screening procedures (Section 3.2.4).

3.1.9 Participant Restrictions During the Study

3.1.9.1 General and Dietary

Participants must not eat or drink anything hot or cold within 10 minutes before oral temperature is taken.

3.2 Withdrawal of Participants From the Study or Study Dosing

3.2.1 Participant Withdrawal From the Study

Participants can withdraw consent and discontinue from the study at any time, for any reason, without prejudice to further treatment the participant may need to receive.

If participant desires to withdraw from the study because of an AE, the investigator will try to obtain agreement to follow up with the participant until the event is considered resolved or stable and will then complete the EOS electronic case report form (eCRF).

Potential reasons for withdrawing a participant from the study include the following:

- SAE
- AE (non-SAE)
- Protocol violation (specify)
- Consent withdrawal (document reason); this includes participants who, at the Participant Decision Visit, withdraw consent from continuing in the study.
- Lost to follow-up
- Other (specify)

3.2.2 Handling Withdrawal From the Study

When a participant withdraws or is withdrawn from the study, the reason(s) for withdrawal will be recorded by the investigator on the relevant page of the eCRF. These participants will be requested to complete the EOS assessments at the study completion visit as outlined in the respective SoE for each study part.

3.2.3 Lost to Follow-up

A participant will be considered lost to follow-up if he or she repeatedly fails to return for scheduled visits without stating an intention to withdraw consent and is unable to be contacted by the study site. The following actions must be taken if a participant fails to return to the study site for a required study visit:

- The site must attempt to contact the participant and reschedule the missed visit as soon as possible, counsel the participant on the importance of maintaining the assigned visit schedule and ascertain whether the participant wishes to and/or should continue in the study.
- Before a participant is deemed lost to follow-up, the investigator or designee must make every effort to regain contact with the participant (where possible, 3 telephone calls and, if necessary, a certified letter to the participant's last known mailing address or local equivalent methods). These contact attempts (eg, dates of telephone calls and registered letters) should be documented in the participant's study source document.
- Should the participant continue to be unreachable, he/she will be considered to have withdrawn from the study.
- A participant should not be considered lost to follow-up until due diligence has been completed. Date of withdrawal/lost to follow-up should be the date of last contact with the participant where safety status of the participant was assessed (eg, study site visit, telephone call).

3.2.4 Replacements (Part A, Blinded Phase Only)

Any participant who is withdrawn, who is significantly outside the allowed injection window, or who is lost to follow-up from the study may be replaced at the Sponsor's discretion.

3.2.5 Participant Withdrawal From Study Dosing

Every reasonable attempt will be made to follow up with participants for safety throughout the entire study period, even if further injection is withheld or the participant misses one or more visits.

Unless consent is withdrawn, a participant who withdraws or is withheld from receiving the second dose of study vaccine will remain in the study and complete all scheduled visits and assessments.

The investigator, in consultation with the Sponsor's medical monitor, may withhold a participant from further injection if the participant experiences any of the following:

- Becomes pregnant
- Develops, during the course of the study, symptoms or conditions listed in the exclusion criteria (Section 3.1.7)
- Experiences an AE (other than reactogenicity) after injection that is considered by the investigator to be related to investigational product (Section 3.4.8.10) and is of Grade 3 (severe) or greater severity (Appendix 2)
- Experiences an AE or SAE that, in the judgment of the investigator, requires study vaccine withdrawal due to its nature, severity, or required treatment, regardless of the causal relationship to vaccine
- Experiences a clinically significant change in clinical laboratory test results, vital sign measurements, or general condition that, in the judgment of the investigator, requires vaccine withdrawal
- Experiences anaphylaxis clearly attributed to study vaccine
- Experiences generalized urticaria related to the study vaccine

The reason(s) for withdrawal from further injection will be recorded.

3.3 Study Dosing Groups

3.3.1 Method of Assigning Participants to Dosing Groups (Part A and Part C)

There are 2 age cohorts in Part A of this study: participants ≥ 18 to < 55 years old in Cohort l and participants ≥ 55 years old in Cohort 2. During Part A, the Blinded Phase of the study, within each age cohort, approximately 300 participants will be randomly assigned in 1:1:1 ratio to receive mRNA-1273 50 µg, mRNA-1273 100 µg, or placebo. The randomization will be in a blinded manner using a centralized Interactive Response Technology (IRT), in accordance with pre-generated randomization schedules. Only the unblinded pharmacy personnel (Section 3.3.5) will have controlled access to which arm the participant is randomly assigned.

Part C will be open label and the IRT system will not be utilized. The enrollment and vaccination in each study arm will be sequential. The first 20 participants will be enrolled and dosed on OL-

D1 with the mRNA-1273.351 50 µg dose. Upon completion of the first 50 µg dose arm, 20 participants will be enrolled in the second arm and will receive the mRNA-1273/mRNA-1273.351 mixture (50 µg total dose). Following completion of the second arm, 20 participants will be enrolled and dosed with the mRNA-1273.351 20 µg dose. In addition to the single dose of investigational product at OL-D1 (Day 1), a second booster dose of mRNA-1273.351 (20 µg or 50 µg) or mRNA 1273/mRNA-1273.351 mixture may be administered approximately 56 days after the first dose. The administration of a second dose of IP will be based on the magnitude of the immune response on either OL-Day 15 or OL-Day 29 after a single dose. Specifically, if the immune response is lower than the response observed after two priming doses of mRNA-1273 in study P201 or P301, a second dose of either mRNA-1273.351 or mRNA-1273/mRNA 1273.351 mixture may be considered.

Dose group assignment in each cohort and stratification within each cohort for Part A and Part C is summarized in Table 1 and Table 2, respectively.

Table 1: Dose Group Assignment (Part A)

Cohort	Treatment Groups	Investigational Product	Number of Participants
Cohort 1<br>≥ 18 to < 55 years old	mRNA-1273 Arm	mRNA-1273 50 µg	100
	mRNA-1273 Arm	mRNA-1273 100 µg	100
	Placebo Arm	Placebo	100
Cohort 2<br>≥ 55 years old	mRNA-1273 Arm	mRNA-1273 50 µg	100
	mRNA-1273 Arm	mRNA-1273 100 µg	100
	Placebo Arm	Placebo	100
**Total**			**600**

Table 2: Dose Group Assignment (Part C)

Treatment Groups	Investigational Product	Number of Participants
mRNA-1273.351 Arm	mRNA-1273.351 20 µg	20
mRNA-1273.351 Arm	mRNA-1273.351 50 µg	20
mRNA-1273/mRNA-1273.351 mixture Arm	25 µg mRNA-1273 and 25 µg mRNA-1273.351 mixture (50 µg total)	20
**Total**		**60**

3.3.2 Investigational Product Administration

In the Blinded Part A of the study, investigational product will be administered as an IM injection into the deltoid muscle on a 2-dose injection schedule on Day 1 and Day 29, with a 28-day interval between doses. Each injection will have a volume of 0.5 mL and contain mRNA-1273 50 µg, mRNA-1273 100 µg, or saline placebo. Preferably, vaccine should be administered into the nondominant arm.

In the open-label Part B of the study, mRNA-1273 vaccine will be administered as an IM injection into the deltoid muscle following the injection schedule for each group based on the product received: participants who received 1 or 2 doses of mRNA-1273 (50 µg and 100 µg) will receive a single booster dose of mRNA-1273 50 µg on OL-Day 1 in Part B (Table 11); participants who received placebo in Part A will receive 2 doses of mRNA-1273 100 µg on open-label (OL)-Day 1 and OL-Day 29 of Part B (Table 11). In addition, participants who received only 1 dose of mRNA-1273 (50 µg or 100 µg) in Part A, will be receive a single booster dose of mRNA-1273 (50µg) on OL-D1 in Part B (Table 12).

In the open-label Part C of the study, the investigational product will be administered as a single IM injection into the deltoid muscle on the day of the participant's consent (OL-D1). Each injection will have variable dose volumes; the 20-µg dose of mRNA-1273.351 is 0.2 mL, the 50-µg dose of mRNA-1273.351 is 0.5 mL, and the 50-µg dose of pharmacy prepared mixture of mRNA-1273/mRNA-1273.351 is 0.5 mL.

In Part A, the investigational product will be prepared for injection as a single 0.5 mL dose for each participant based on the cohort and randomization assignment, as detailed in the mRNA-1273-P201 Pharmacy Manual. In Part B, the investigational product will be prepared for injection as a single 0.5 mL dose volumes for each participant as detailed in the mRNA-1273-P201 Pharmacy Manual. In Part C, the investigational product will be prepared for injection as a single dose with varied volumes for each participant as detailed in mRNA1273-P201 Pharmacy Manual addendum for Part C. Unblinded pharmacy personnel, who will not participate in any other aspect of the study during Part A, the Blinded Phase, will perform investigational product accountability, dose preparation, and investigational product administration. The investigator will designate an unblinded clinical team member to provide oversight to the administration of investigational product so that it proceeds according to the procedures stipulated in this study protocol and the mRNA-1273-P201 Pharmacy Manual. Study-specific training will be provided.

At each visit when investigational product is administered, participants will be monitored for a minimum of 30 minutes after administration. Assessments will include vital sign measurements and monitoring for local or systemic reactions (Schedule of Events, Table 9).

Eligibility for subsequent investigational product injection is determined by following the criteria outlined in Section 3.3.2.2.

The study site will be appropriately staffed, staff will be trained on emergency resuscitation, and will have stocked rescue medications (such as epinephrine, steroids, antihistamines, and intravenous fluids) should any severe reaction (eg, anaphylaxis or urticaria) occur that requires immediate intervention.

The rules for pausing dosing are provided in Section 3.3.2.1.

3.3.2.1 Pause Rules

During Part A of the study, the investigators, study medical monitor, and Sponsor will monitor for events that could trigger a study pause (Table 3). Although these pause rules are not applicable during Part B and Part C, participants will continue to be monitored for the events in Table 3, and the Sponsor will be notified if any of these events occur.

Table 3: Pause Rule Criteria, Events, and Thresholds

Pause Rule Criterion	Event	Participant Threshold for Triggering Study Pause
1	Any death due to SARS-CoV-2 infection	≥ 1
2	Any SAE or Grade 4 AE that cannot be reasonably attributed to a cause other than injection	≥ 3
3	ICU admissions in Cohort 1 due to SARS-CoV-2 infection	≥ 3
4	ICU admissions in Cohort 2 due to SARS-CoV-2 infection	≥ 6

Abbreviations: AE = adverse event; ICU = intensive care unit; SAE = serious adverse event; SARS-Cov-2 = severe acute respiratory syndrome coronavirus that causes COVID-19.

If any of the thresholds for a study pause is met during Part A, the Sponsor will immediately suspend further enrollment and/or study dosing by notifying all investigators. Such a suspension will remain in force until the threshold event is adjudicated by the Safety Monitoring Committee (SMC; Section 6.1.1).

The investigator or designee is responsible for reporting to the Sponsor, via the electronic data capture (EDC) system within 24 hours of observation, each event potentially meeting any pause rule criterion. The Sponsor will inform the SMC (Section 6.1.1) of any event potentially meeting any pause rule criterion. The SMC will review all available study data to adjudicate such events in accordance with the SMC charter.

The Sponsor will also actively monitor the following and provide them to the SMC for review as they become available:

- Instances of study halting rules triggered in the Phase 1 DMID study (NCT04283461)
- Histopathological data suggestive of vaccine-enhanced disease in ongoing nonclinical studies

The Sponsor will notify the Center for Biologics and Evaluation Research (CBER) within 48 hours in the event of a study pause. In the event of a study pause, all safety and immunogenicity assessments will continue per protocol. The window allowance for injection visits may be extended by an additional 7 days for affected participants at the discretion of the Sponsor.

During Part B and Part C of the study, the Sponsor will continue to inform the SMC of the occurrence of any of the events in Table 3. The SMC will review all available relevant study data to adjudicate such events in accordance with the SMC charter.

3.3.2.2 Contraindications to Subsequent Injection

Prior to receiving a second injection, participants will be reassessed to ensure that they continue to meet eligibility requirements as outlined below. For Part C, if the option to administer a second injection is exercised, prior to receiving a second injection, participants will be reassessed to ensure they continue to meet eligibility requirements as outlined below.

The following events in a participant constitute absolute contraindications to any further administration of the investigational product to that participant. If any of these events occur during the study, the participant must not receive additional doses of vaccine but will be encouraged to continue study participation for safety through 6 months following last injection (Section 3.2.5).

- Symptomatic COVID-19 at the time of a second injection. If COVID-19 is suspected on or prior to administration of a second injection, further administration of investigational product must be withheld, as applicable, until COVID-19 test results are available.
- Anaphylaxis or systemic hypersensitivity reaction following the administration of vaccine.
- Any SAE judged by investigator or Sponsor to be related to study vaccine.
- Pregnancy
- Any clinically significant medical condition that, in the opinion of the investigator, poses an additional risk to the participant if he/she continues to participate in the study.

The following events constitute contraindications to administration of study vaccine at certain points in time, and if any of these events occur at the time scheduled for injection, the participant may be injected at a later date, within the time window specified in the Schedule of Events (Section 7.1), or the participant may be withdrawn from dosing at the discretion of the investigator (Section 3.2.5):

- Acute moderate or severe infection with or without fever at the time of injection
- Fever, defined as body temperature ≥ 38.0°C (100.4°F) at the time of injection

Participants with a minor illness without fever, as assessed by the investigator, can be administered investigational product. Participants with a fever of 38.0°C (100.4°F) or higher will be contacted within the time window acceptable for participation and reevaluated for eligibility.

3.3.3 Identity of Investigational Product

The mRNA-1273 vaccine dosed in Part A and Part B of this study is an LNP dispersion of an mRNA encoding the prefusion stabilized S protein of SARS-CoV-2. The mRNA-1273.351 vaccine dosed in Part C of this study is an LNP dispersion of an mRNA encoding the prefusion stabilized S protein of SARS-CoV-2 variant B1.351. The mRNA-1273/mRNA-1273.351 mixture vaccine dosed in Part C of this study utilizes a mixture of mRNA-1273 and mRNA-1273.351 drug products to administer a single dose containing both vaccines. All vaccines are formulated in LNPs composed of 4 lipids (1 proprietary and 3 commercially available): the proprietary ionizable lipid SM-102; cholesterol; 1,2-distearoyl-sn-glycero-3-phosphocholine (DSPC); and 1-monomethoxypolyethyleneglycol-2,3-dimyristylglycerol with polyethylene glycol of average molecular weight 2000 (PEG2000-DMG).

The mRNA-1273, mRNA-1273.351, and mRNA-1273/mRNA-1273.351 mixture vaccines are provided as sterile liquids for injection, white to off white dispersion in appearance. The mRNA-1273 vaccine dosed in Part A and Part B is in 20 mM Tris buffer containing 87 mg/mL sucrose and 10.7 mM sodium acetate at pH 7.5 and is provided at a concentration of 0.5 mg/mL. The mRNA-1273 vaccine dosed in Part C is in 20 mM Tris buffer containing 4.3 mM of sodium acetate at pH 7.5 and is provided at a concentration of 0.5 mg/mL. The mRNA-1273.351 vaccine in Part C is in 20 mM Tris buffer containing 10.7 mM of sodium acetate at pH 7.5 and provided at a concentration of 0.2 mg/mL. mRNA-1273 and mRNA-1273.351 vaccines will be mixed at the clinical site to enable dosing of the mRNA-1273/mRNA-1273.351 (50 µg total) arm.

The placebo is 0.9% sodium chloride (normal saline) injection, USP.

3.3.4 Management of Investigational Product

3.3.4.1 Delivery and Receipt

The Sponsor or designee is responsible for the following:

- Supplying the investigational product
- Confirming the appropriate labeling of mRNA-1273, mRNA-1273.351, and mRNA-1273/mRNA-1273.351 mixture Injection, so that it complies with the legal requirements of the US

The investigator is responsible for acknowledging the receipt of the investigational product by a designated staff member at the site, including the following:

- Confirming that the investigational product was received in good condition
- Confirming that the temperature during shipment from the Sponsor to the investigator's designated storage location was appropriate
- Confirming whether the Sponsor has authorized the investigational product for use
- Ensuring the appropriate dose level of mRNA-1273, mRNA-1273.351, and mRNA-1273/mRNA-1273.351 mixture Injection is properly prepared using aseptic technique

Further description of the investigational product and instructions for the receipt, storage, preparation, administration, accountability, and destruction of the investigational product are described in the mRNA-1273-P201 Pharmacy Manual addendum for Part C.

3.3.4.2 Packaging and Labeling

The Sponsor will provide the investigator and study site with adequate quantities of mRNA-1273, mRNA-1273.351, and mRNA-1273/mRNA-1273.351 mixture. The sterile vaccine product used in Part A and Part B is packaged in a 2-mL glass vial with a 0.6-mL fill volume. The mRNA-1273 vaccine used in Part C is packaged in a 10-mL vial with a 6.3-mL fill volume. The mRNA-1273.351 vaccine used in Part C is packaged in a 2-mL vial with a 0.8-mL fill volume. The vaccines will have all required labeling per regulations and will be supplied to the pharmacy in an unblinded manner. Each vial will be individually labeled for future participant identification purposes.

The mRNA-1273 and mRNA-1273.351 injections will be packaged and labeled in accordance with the standard operating procedures (SOPs) of the Sponsor or of its designee, Code of Federal Regulations Title 21 (CFR), Good Manufacturing Practice (GMP) guidelines, International

Council for Harmonisation (ICH) Good Clinical Practice (GCP) guidelines, guidelines for Quality System Regulations, and applicable regulations.

The Sponsor or Sponsor's designee will supply the 0.9% sodium chloride injection for use as both a placebo and a diluent to mRNA-1273 and mRNA-1273.351. The 0.9% sodium chloride bears a commercial label and does not contain study-specific identification.

3.3.4.3 Storage

The mRNA-1273 vaccine in Part A and Part B must be stored at -90°C to -60°C (-130°F to -76°F). The mRNA-1273 vaccine in Part C must be stored at -25°C to -15°C. The mRNA-1273.351 vaccine used in Part C must be stored at -90°C to -60°C (-130°F to -76°F). Freezers must be in a secure area with limited access (unblinded pharmacy staff only) and protected from moisture and light until it is prepared for administration (Section 3.3.2). The freezer should have automated temperature recording and a 24-hour alert system in place that allows for rapid response in case of freezer malfunction. There must be an available back-up freezer. The freezers must be connected to a back-up generator. In addition, vaccine accountability study staff (eg, the unblinded pharmacy personnel) are required to keep a temperature log to establish a record of compliance with these storage conditions. The site is responsible for reporting any mRNA-1273 and mRNA-1273.351 vaccine that was not temperature controlled during shipment or during storage to the unblinded site monitor. Such vaccines will be retained for inspection by the unblinded monitor and disposed of according to approved methods.

The 0.9% sodium chloride injection (USP) should be stored at 20ºC to 25ºC (68ºF to 77ºF) in a restricted access area.

3.3.4.4 Investigational Product Accountability

It is the investigator's responsibility that the unblinded pharmacy personnel maintain accurate records in an investigational product accountability log of receipt of all investigational product, inventory at the site, dispensing of mRNA-1273, mRNA-1273.351, and placebo, study injections, and return to the Sponsor or alternative disposition of used/unused products.

An unblinded site monitor will review the inventory and accountability log during site visits and after the completion of treatment. Additional details are found in the mRNA-1273-P201 Pharmacy Manual (Part A and Part B) and the mRNA-1273-P201 Pharmacy Manual Addendum for Part C.

3.3.4.5 Handling and Disposal

An unblinded site monitor will reconcile the investigational product during the conduct and at the end of the study for compliance. Once fully reconciled at the site at the end of the study, the investigational product can be destroyed at the investigational site or at a Sponsor-selected third party, as appropriate.

Investigational product may be destroyed at the study site only if permitted by local regulations and authorized by the Sponsor. A Certificate of Destruction must be completed and sent to the Sponsor or designee.

3.3.5 Blinding

See Section 3.1.2 regarding the addition of a participant decision visit as part of this protocol amendment.

Part A of this study is observer blind. The investigator, study staff, study participants, site monitors, and Sponsor personnel (or its designees) will be blinded to the investigational product administered until study end or initiation of Part B, with the following exceptions:

- Unblinded pharmacy personnel (of limited number) will be assigned to vaccine accountability procedures and will prepare and administer mRNA-1273 (or placebo) to all participants. These pharmacy personnel will have no study functions other than study vaccine management, documentation, accountability, preparation, and administration. They will not be involved in participant evaluations and will not reveal the identity of investigational product to either the participant or the blinded study site personnel involved in the conduct of the study unless this information is necessary in the case of an emergency.
- Unblinded site monitors, not involved in other aspects of monitoring, will be assigned as the investigational product accountability monitors. They will have responsibilities to ensure that sites are following all proper investigational product accountability, preparation, and administration procedures.
- A primary analysis of safety and immunogenicity data will be performed after participants have completed Day 57 study procedures. This primary analysis may be performed when all participants in Cohort 1 and the Cohort 2 sentinel group have completed Day 57 study procedures and/or when all participants in Cohort 1 and Cohort 2 have completed Day 57 study procedures. All data relevant to the primary study analysis through Day 57 will be cleaned (ie, data that are as clean as possible) and locked. A limited number of Sponsor and clinical research organization (CRO) personnel will be unblinded to perform the primary study analysis and prepare a final Clinical Study Report (CSR), including individual

listings. The study site staff, investigators, study monitors, and participants will remain blinded until the initiation of Open-Label Part B.

In Part A, the dosing assignment will be concealed by having the unblinded pharmacy personnel prepare the investigational product in a secure location that is not accessible or visible to other study staff. A blinding label over the syringe used for injection will maintain the blind at the time of injection, as the doses containing mRNA-1273 will look different than placebo. Only delegated unblinded site staff will conduct the injection procedure. Once the injection is completed, only the blinded study staff will perform further assessments and interact with the participants. Access to the randomization code will be strictly controlled at the pharmacy.

Part B and Part C of the study will be open label.

3.3.6 Breaking the Blind

A participant or participants may be unblinded in the event of an SAE or other severe event, or if there is a medical emergency requiring the identity of the product to be known to properly treat a participant. If a participant becomes seriously ill or pregnant during the study, the blind will be broken if knowledge of the administered vaccine will affect that participant's dosing options. In the event of a medical emergency requiring identification of the vaccine administered to an individual participant, the investigator will make every attempt to contact the Sponsor medical lead to explain the need for opening the code within 24 hours of opening the code. The investigator will be responsible for documenting the time, date, reason for the code break, and the names of the personnel involved.

In addition to the aforementioned situations where the blind may be broken, the data will also be unblinded to a statistical team at specified time points for the primary study analysis as outlined in Section 4.7.

3.3.7 Dosing Compliance

All doses of investigational product will be administered at the study site under direct observation of unblinded pharmacy personnel and appropriately recorded (date and time) in the eCRF. Unblinded pharmacy personnel will confirm that the participant has received the entire dose of vaccine. If a participant does not receive vaccine or does not receive all of the planned doses, the reason for the missed dose will be recorded.

Participants who miss the second injection due to noncompliance with the visit schedule and not due to a safety pause will still be required to follow the original visit and testing schedule as described in the protocol. Unless consent is withdrawn, a participant who withdraws or is withheld

from receiving the second dose of study vaccine will remain in the study and complete all safety and immunogenicity assessments required through the scheduled EOS.

The study site is responsible for ensuring participants comply with the study windows allowed. If a participant misses a visit, every effort should be made to contact the participant and complete a visit within the defined visit window (Part A Table 9, Part B Table 11, and Table 12, and Part C Table 12). If a participant does not complete a visit within the time window, that visit will be classified as a missed visit and the participant will continue with subsequent scheduled study visits. All safety requirements of the missed visit will be captured and included in the subsequent visit (eg, clinical laboratory testing, eDiary review for reactogenicity, immunologic testing, as applicable).

3.3.8 Prior and Concomitant Medications

3.3.8.1 Prior Medications and Therapies

Information about prior medications (including any prescription or over-the-counter medications, vaccines, or blood products) taken by the participant within the 28 days before providing informed consent (or as designated in the inclusion/exclusion requirements) will be recorded in the participant's eCRF.

3.3.8.2 Concomitant Medications and Therapies

At each study visit, study site staff must question the participant regarding any medications taken and vaccinations received by the participant and record the following information in the eCRF:

- All non-study vaccinations administered within the period starting 28 days before the first study injection.
- All concomitant medications and non-study vaccinations taken through 28 days after each injection. Antipyretics and analgesics taken prophylactically (ie, taken in the absence of any symptoms in anticipation of an injection reaction) will be recorded as such.
- Any concomitant medications relevant to or for the treatment of an SAE or a MAAE.
- Participant will be asked in the eDiary if they have taken any antipyretic or analgesic to treat or prevent fever or pain within 7 days after each study injection, including the day of injection. Reported antipyretic or analgesic medications should be recorded in the source document by the site staff during the post-injection study visits or via other participant interactions (eg, telephone calls).

The use of the following concomitant medications and/or vaccines will not require withdrawal of the participant from the study but may determine a participant's eligibility to receive a second dose or evaluability in the per-protocol analysis (analysis sets are described in Section 4.4):

- Any investigational or nonregistered product (drug or vaccine) other than the study vaccine used during the study period.
- Immunosuppressants or other immune-modifying drugs administered chronically (ie, more than 14 days in total) during the study period. For corticosteroids, this will mean that prednisone ≥ 20 mg/day or the equivalent is not permitted. Inhaled, nasal, and topical steroids are allowed.
- Long-acting immune-modifying drugs administered at any time during the study period (eg, infliximab).
- A licensed vaccine administered during the period from 28 days before through 28 days after each study injection, except for any licensed influenza vaccine that was administered more than 14 days before or after any study injection.
- Immunoglobulins and/or any blood products administered during the study period.

Concomitant medications (including vaccinations) will be coded using the WHO Drug Dictionary. If a participant takes a prohibited drug therapy, the investigator and the CRO's medical monitor will make a joint decision about continuing or withholding further injection of the participant based on the time the medication was administered, the drug's pharmacology and pharmacokinetics, and whether use of the medication will compromise the participant's safety or interpretation of the data. It is the investigator's responsibility to ensure that details regarding the concomitant medications are adequately recorded in the eCRF.

3.4 Study Procedures

Before performing any study procedures, all potential participants will sign an informed consent form (ICF) (as detailed in Section 5.3). Participants will undergo study procedures at the time points specified in the Schedule of Events (Table 9).

After participant proceeds to the open-label Part B of the study at the Participant Decision Clinic Visit (Table 10), Part A schedule of events (Table 9) will no longer be followed and become obsolete.

Upon the participant's decision to end participation in Moderna's mRNA-1273-P301 COVE study, and consent to enrolling into Part C of this study, they will follow study procedures as specified in the Schedule of Events (Table 12).

A participant also can be seen for an unscheduled visit at any time during the study. An unscheduled visit may be prompted by reactogenicity issues or new or ongoing AEs. The site also has the discretion to make reminder telephone calls or send text messages to inform the participant about visits, review eDiary requirements, or follow up on ongoing or outstanding issues.

In accordance with "FDA Guidance on Conduct of Clinical Trials of Medical Products during COVID-19 Public Health Emergency" (DHHS 2020), investigators may convert study site visits to telemedicine visits with the approval of the Sponsor. Such action should be taken to protect the safety and well-being of study participants and study site staff or to comply with state or municipal mandates.

3.4.1 Assessment for SARS-CoV-2 Infection

Study participants will have nasopharyngeal swab samples collected for SARS-CoV-2 testing at time points specified in the Schedule of Events (Part A Table 9 and Part B Table 10, Table 11 and Table 12, and Part C Table 12).

For Part A, Part B, and Part C of this study, a study illness visit or a consultation will be arranged within 24 hours or as soon as possible to collect a nasopharyngeal swab sample to ascertain the presence of SARS-CoV-2 via PCR if a participant experiences any of the following:

- Signs or symptoms of SARS-CoV-2 infection as defined by the CDC (CDC 2020c)
- Exposure to an individual confirmed to be infected with SARS-CoV-2
- MAAE suggesting a SARS-CoV-2 infection

Additionally, clinical information will be carefully collected to evaluate the severity of the clinical case. All findings will be recorded in the eCRF.

If scheduled, a study site illness visit may include assessments such as medical history, physical examination, blood sampling for clinical laboratory testing, and nasopharyngeal swab sampling for viral PCR (including multiplex PCR for respiratory viruses including SARS-CoV-2) to evaluate the severity of the clinical case. Radiologic imaging studies may be conducted. Blood samples will be collected at all illness visits for potential future immunologic assessment of SARS-CoV-2 infection.

If participants are confirmed to have SARS-CoV-2 infection, the investigator will notify the participant's primary care physician of the diagnosis. Additionally, a convalescent visit will be scheduled approximately 28 days after diagnosis. At this visit, a blood sample will be collected for potential future immunologic assessment of SARS-CoV-2 infection.

If participants are confirmed to have SARS-CoV-2 infection, and are asymptomatic, the investigator will notify the participant's primary care physician and local health authority, as per local regulations. If the participant had known exposure to COVID-19 (eg, exposure to someone with confirmed COVID-19 disease), it will be captured in the COVID-19 exposure form, and the participant will be discontinued from future study treatment only, and will continue to follow all other study assessments as outlined in the protocol. Additionally, a convalescent visit will be scheduled approximately 28 days after diagnosis. At this visit, a blood sample will be collected for potential immunologic assessment of SARS-CoV-2 infection.

Any confirmed SARS-CoV-2 infection occurring in participants, except asymptomatic infection diagnosed at Day 1, will be captured as an MAAE along with relevant concomitant medications and details about severity, seriousness, and outcome.

3.4.2 Use of Electronic Diaries

At the time of consent, the participants must confirm they will be willing to complete an eDiary using either an application downloaded to their smartphone or using a device that is provided at the time of enrollment. Before enrollment on Day 1, the participant will be instructed to download the eDiary application or will be provided an eDiary device to record solicited ARs (Section 3.4.8.4) beginning on Day 1 or at the Participant Decision Visit in the open-label Part B of the study.

In Part A, at each injection visit, participants will be instructed (Day 1) or reminded (Day 29) on thermometer usage to measure body temperature, ruler usage to measure injection site erythema and swelling/induration (hardness), and self-assessment for localized axillary swelling or tenderness on the same side as the injection arm.

In Part B and Part C, at the respective injection visit, participants will be reminded on thermometer usage to measure body temperature, ruler usage to measure injection site erythema and swelling/induration (hardness), and self-assessment for localized axillary swelling or tenderness on the same side as the injection arm.

At each injection visit, participants will record data into the eDiary starting approximately 30 minutes after injection under supervision of the study site staff to ensure successful entry of assessments. The site staff will perform any retraining as necessary. Study participants will continue to record data in the eDiary after they leave the study site, preferably in the evening and at the same time each day, on the day of injection and for 6 days following injection.

Participants will record the following data in the eDiary:

- Solicited local and systemic reactogenicity ARs, as defined in Section 3.4.8.4, that occur on the day of each vaccine administration and during the 7 days after vaccine administration (ie, the day of injection and 6 subsequent days). Any solicited AR that is ongoing beyond Day 7 will be reported in the eDiary until resolution. Adverse reactions recorded in diaries beyond Day 7 should be reviewed by study site staff either during the next scheduled telephone call or at the next study site visit (Table 9, Table 11, and Table 12).
- Daily oral body temperature measurement should be performed at approximately the same time each day using the thermometer provided by the study site. If body temperature is taken more than once in a given day, only the highest temperature reading should be recorded.
- Measurement, as applicable, for solicited local ARs (injection site erythema and swelling/induration); the size measurements will be performed using the ruler provided by the study site.
- Participants will be queried by the eDiary whether any medications were taken to treat or prevent pain or fever on a day of injection or for the 6 subsequent days.

The eDiary will be the only source documents allowed for solicited systemic or local ARs (including body temperature measurements). Participants will be instructed to complete eDiary entries daily. The participant will have a limited window on the following day to complete assessments for the previous day; quantitative temperature recordings and measurement of any injection site erythema or swelling/induration reported on the following day may be excluded from the analyses of solicited ARs.

Any new safety information reported during safety telephone calls or at site visits (including a solicited reaction) that is not already captured in the eDiary will be described in the source documents as a verbally reported event. Any AR reported in this manner must be described as an unsolicited event and therefore entered on the AE eCRF.

Study site staff will review eDiary data with participants at the Day 8 and Day 36 visits in the blinded Part A of the study. In the open-label Part B of the study, eDiary data will be reviewed in person on open-label (OL) Day 8 for participants receiving a single booster (Table 12) and via phone call 7 days after each vaccination for those participants receiving 2 doses of 100 µg mRNA-1273 (Table 11). In Part C of the study, eDiary data will be reviewed in person on open-label Day 8 (Table 12).

The eDiary will also be used after the Day 57 visit (including OL-Day 57 in Part B and Part C) to capture the occurrence of AEs, MAAEs, SAEs, or AEs leading to study withdrawal, every 4 weeks from Day 71 through Day 183 in Part A (Table 9), from OL-Day 71 through OL- Day 155 in Part B (Table 11 and Table 12) and Part C (Table 12), and from Day 223 through Day 363 in Part A only (Table 9).

The eDiary will prompt the participant to complete an eDiary questionnaire that collects the following data:

- Changes in health since last completing the questionnaire or since last in contact with the study site.
- Known exposure to someone with known COVID-19 or SARS-CoV-2 infection.
- Any experience of symptoms of COVID-19.
- Any MAAEs or SAEs.

If an eDiary record results in identification of relevant safety events according to the study period or of symptoms of COVID-19, the site will follow up with the participant via telephone and assess the need for an unscheduled visit.

Completion of eDiary questionnaires will alternate with safety telephone calls (Section 3.4.3) as the procedure for safety follow-up approximately every 2 weeks after the Day 57 (Table 9) or the OL-Day 57 visit (Table 11 and Table 12).

3.4.3 Safety Telephone Calls

A safety telephone call is a telephone call made to the participant by trained site personnel. This call will follow a script, which will facilitate the collection of relevant safety information.

In blinded Part A, safety telephone calls are scheduled to occur every 4 weeks from Day 85 through Day 197 and from Day 237 through Day 377 (Table 9).

In the open-label Part B, the safety telephone calls are scheduled to occur every 4 weeks (Table 11 and Table 12).

In the open-label Part C, the safety telephone calls are scheduled to occur every 4 weeks (Table 12).

The participant will be interviewed according to the script about occurrence of AEs, MAAEs, SAEs, AEs leading to study withdrawal, concomitant medications associated with those events, and any non-study vaccinations (Section 3.4.8.6). In addition, study personnel will collect information on known participant exposure to someone with known COVID-19 or SARS-CoV-2

infection and on participant experience of COVID-19 symptoms. All safety information collected from the telephone contact must be documented in source documents as described by the participant and not documented on the script used for the safety telephone contact.

3.4.4 Safety Laboratory Assessments

In the blinded Part A of the study, laboratory tests will be performed by the central laboratory unless otherwise specified. Screening safety laboratory tests will include complete blood count with differential, alanine aminotransferase (ALT), aspartate aminotransferase (AST), total and direct bilirubin, alkaline phosphatase (ALP), blood urea nitrogen/creatinine, prothrombin time (PT), and partial thromboplastin time (PTT). These safety laboratory tests are to be repeated at Day 29 and Day 57 only for Cohort 2 (≥ 55 years of age).

Additional tests include the following:

In both Parts A and B, a point-of-care urine pregnancy test will be performed at the Screening Visit (Day 0) or Participant Decision Visit (OL-Day 1) and before each vaccine administration. In Part C, a point-of-care urine pregnancy test will be performed at the OL-Day 1 prior to vaccine administration.

At any time, a pregnancy test either via blood or point-of-care urine can be performed, at the discretion of the investigator.

- If not documented in a female participant's medical records, an FSH test may be performed at the Screening Visit (Day 0, Part A, Blinded Phase only), as necessary and at the discretion of the investigator, to confirm postmenopausal status.
- Hepatitis B surface antigen, hepatitis C virus antibody, and HIV virus (types 1 and 2) antibody at the Screening Visit (Day 0, Part A, Blinded Phase only).

3.4.5 Immunogenicity Assessments

Blood samples for immunogenicity assessments will be collected at the time points indicated in the Schedule of Events for each study part, Table 9 for the blinded Part A, and Table 10, Table 11 and Table 12 for open-label Part B, and Table 12 for Part C. On Day 1 and Day 29 (Table 9), and on OL-Day 1 (Table 12) and on OL-Day 1 and OL-Day 29 (Table 11), blood samples for immunogenicity assessment will be collected before administration of vaccine. The following analytes will be measured:

- Serum bAb level against SARS-CoV-2 as measured by enzyme-linked immunosorbent assay (ELISA) specific to the SARS-CoV-2 S protein

- Serum nAb titer against SARS-CoV-2 as measured by pseudovirus and/or live virus neutralization assays

In Part C, serum samples for immunogenicity assessment will be analyzed for the following:

- Serum nAb against SARS-CoV-2 as measured by pseudovirus (and/or live virus) using the B.1.351 and the D614G variant sequences of SARS-CoV-2 S protein (and/or variant strains)
- Serum bAb against SARS-CoV-2 as measured by ELISA against the B.1.351 and the D614G variant sequences of SARS-CoV-2 S protein

Sample aliquots will be designed to ensure that backup samples are available and that adequate vial volumes may allow further testing needs. The actual time and date of each sample collected will be recorded in the eCRF, and unique sample identification will be utilized to maintain the blind at the laboratory at all times and to allow for automated sample tracking and housing. Handling and preparation of the samples for analysis, as well as shipping and storage requirements, will be provided in a separate study manual.

The ELISA and measurement of nAb titers will be performed in a laboratory designated by the Sponsor.

For participants who provide consent (Section 5.3), serum from immunogenicity testing may be used for future research, which may be performed at the discretion of the Sponsor to further characterize the immune response to SARS-CoV-2, additional assay development, and the immune response across the coronaviruses.

3.4.6 Blood Sampling Volumes

In Part A, the maximum planned volumes of blood sampled per participant are 66 mL for 1 day, 182 mL for 28 days, and 398 mL for the complete study (Table 4).

Table 4: Maximum Blood Sampling Volumes per Participant by Visit (Part A, Blinded)

Study Visit Day	D0	D1	D15	D29	D43	D57	D209	D394	Total
Safety laboratory tests	16 mL			16[1] mL		16[1] mL			48 mL
Immunogenicity assays		50 mL	50 mL	50 mL	50 mL	50 mL	50 mL	50 mL	350 mL
**Total**	**16 mL**	**50 mL**	**50 mL**	**66 mL**	**50 mL**	**66 mL**	**50 mL**	**50 mL**	**398 mL**

Abbreviation: D = Day.
[1]Only participants in Cohort 2 will have blood sampled for safety laboratory tests at Day 29 and Day 57.

In Parts B and C, the maximum planned volumes of blood sampled per participant are 20 mL per visit, 80 mL for 28 days, and a total of 120 mL (Table 5).

Table 5: Maximum Blood Sampling Volumes per Participant by Visit (Part B and Part C Open-label)

Study Visit Day	OL-D1	OL-D8	OL-D15	OL-D29	OL-D57	OL-M6-7[1]	Total
Immunogenicity assays	20 mL	20 mL	20 mL	20 mL	20 mL	20 mL	**120 mL**

Abbreviation: OL = Open-Label; D = Day; M = month.
[1] OL-M6-7 is calculated based on the participant's last vaccination day, ie, for participants receiving a single booster dose this will be OL-M6, and for participants; who receive 2 injections, this visit will be based off of the day of second injection (ie, OL-Day 29), and will be OL-M7. In Part C, the last blood sample collection will be at OL-M6.

3.4.7 Safety Assessments

In Part A (blinded), Part B (open-label), and Part C (open-label), safety assessments will include monitoring and recording of the following for each participant:

- Solicited local and systemic ARs (Section 3.4.8.4) that occur during the 7 days following each injection (ie, the day of injection and 6 subsequent days). Solicited ARs will be recorded daily using eDiaries (Section 3.4.2).
- Unsolicited AEs observed or reported during the 28 days following each injection (ie, the day of injection and 27 subsequent days). Unsolicited AEs are AEs that are not included in the protocol-defined solicited ARs (Section 3.4.8.4).
- AEs leading to discontinuation from dosing and/or study participation from Day 1 through Day 394 (Part A), OL-Month 6 or 7 (Part B), and OL-Month 6 (Part C) or withdrawal from the study.
- MAAEs from Day 1 through Day 394 (Part A), OL-Month 6 or 7 (Part B), and OL-Month 6 (Part C) or withdrawal from the study.

- SAEs from Day 1 through Day 394 (Part A), OL-Month 6 or 7 (Part B), and OL-Month 6 (Part C) or withdrawal from the study.
- Results of safety laboratory tests (Part A).
- Vital sign measurements.
- Physical examination findings.
- Assessments for SARS-CoV-2 infection from Day 1 through study completion (Section 3.4.1).

3.4.8 Safety Definitions

3.4.8.1 Adverse Event

An AE is defined as any untoward medical occurrence associated with the use of a drug in humans, whether or not considered drug related.

A treatment-emergent AE (TEAE) is defined as any event not present before exposure to vaccine or any event already present that worsens in intensity or frequency after exposure.

Events Meeting the Adverse Event Definition

- Any abnormal laboratory test result (hematology, clinical chemistry, or PT/PTT) or other safety assessment (eg, electrocardiogram, radiological scan, vital sign measurement), including one that worsens from baseline and is considered clinically significant in the medical and scientific judgment of the investigator.
- Exacerbation of a chronic or intermittent pre-existing condition including either an increase in frequency and/or intensity of the condition.
- New conditions detected or diagnosed after mRNA-1273 vaccine administration even though it may have been present before the start of the study.

Events NOT Meeting the Adverse Event Definition

- Medical or surgical procedure (eg, endoscopy, appendectomy): the condition that leads to the procedure is the AE.
- Situations in which an untoward medical occurrence did not occur (social and/or convenience admission to a hospital).

An AR is any AE for which there is a reasonable possibility that the investigational product caused the AE (Section 3.4.8.4). For the purposes of investigational new drug safety reporting,

"reasonable possibility" means that there is evidence to suggest a causal relationship between the investigational product and the AE.

An unsolicited AE is any AE reported by the participant that is not specified as a solicited AR in the protocol or is specified as a solicited AR in the protocol but starts outside the protocol-defined post-injection period for reporting solicited ARs (ie, for the 7 days after each injection).

3.4.8.2 Medically Attended Adverse Event

An MAAE is an AE that leads to an unscheduled visit to a healthcare practitioner (HCP). This would include visits to a study site for unscheduled assessments (eg, rash assessment, abnormal laboratory follow-up, SARS-CoV-2 infection [Section 3.4.1]) and visits to HCPs external to the study site (eg, urgent care, primary care physician). Investigators will review unsolicited AEs for the occurrence of any MAAEs. All MAAEs must be fully reported on the MAAE page of the eCRF.

3.4.8.3 Serious Adverse Event

An AE (including an AR) is considered an SAE if, in the view of either the investigator or Sponsor, it results in any of the following outcomes:

- Death
  A death that occurs during the study or that comes to the attention of the investigator during the protocol-defined follow-up period must be reported to the Sponsor, whether or not it is considered related to study vaccine.
- Is life-threatening
  An AE is considered life-threatening if, in the view of either the investigator or the Sponsor, its occurrence places the participant at immediate risk of death. It does not include an AE that, had it occurred in a more severe form, might have caused death.
- Inpatient hospitalization or prolongation of existing hospitalization
  In general, inpatient hospitalization indicates the participant was admitted to the hospital or emergency ward for at least one overnight stay for observation and/or treatment that would not have been appropriate in the physician's office or outpatient setting. The hospital or emergency ward admission should be considered an SAE regardless of whether opinions differ as to the necessity of the admission. Complications that occur during inpatient hospitalization will be recorded as an AE; however, if a complication/AE prolongs hospitalization or otherwise fulfills SAE criteria, the complication/AE will be recorded as a separate SAE.

- Persistent or significant incapacity or substantial disruption of the ability to conduct normal life functions
  This definition is not intended to include experiences of relatively minor medical significance such as uncomplicated headache, nausea/vomiting, diarrhea, influenza, and accidental trauma (eg, sprained ankle) which may interfere with or prevent everyday life functions but do not constitute a substantial disruption.
- Congenital anomaly or birth defect
- Medically important event
  Medical judgment should be exercised in deciding whether SAE reporting is appropriate in other situations, such as important medical events that may not be immediately life-threatening or result in death or hospitalization but may jeopardize the participant or require medical or surgical intervention to prevent one of the other outcomes listed in the above definition. These events should usually be considered serious. Examples of such medical events include allergic bronchospasm requiring intensive treatment in an emergency room or at home, blood dyscrasias or convulsions that do not result in inpatient hospitalization, or the development of drug dependency or drug abuse.

3.4.8.4 Solicited Adverse Reactions

The term "reactogenicity" refers to the occurrence and intensity of selected signs and symptoms (ARs) occurring after vaccine administration. The eDiary (Section 3.4.2) will solicit participant reporting of ARs using a structured checklist. Participants will record such occurrences in an eDiary on the day of each vaccine administration and for the 6 days after a day of injection.

The following local ARs will be solicited by the eDiary: pain at injection site, erythema (redness) at injection site, swelling/induration (hardness) at injection site, and localized axillary swelling or tenderness ipsilateral to the injection arm.

The following systemic ARs will be solicited by the eDiary: headache, fatigue, myalgia (muscle aches all over the body), arthralgia (aching in several joints), nausea/vomiting, rash, body temperature (potentially fever), and chills.

The study site staff will contact the participant within 24 hours of becoming aware of the event if any of the following occurs within 7 days after study injection:

- Severe (Grade 3) local or systemic ARs (Table 6)
- Presence of any rash

The purpose of the contact is to assess the nature of AR, including assessment of potential pause rules. In the event that rash is reported, the participant will be asked to return to the study site for assessment by the investigator.

The investigator will review, confirm, and grade reactogenicity according to the grading scales presented in Table 6, modified from the Toxicity Grading Scale for Healthy Adult and Adolescent Volunteers Enrolled in Preventive Vaccine Clinical Trials (DHHS 2007).

If a solicited local or systemic AR continues beyond 7 days after injection, the participant will be prompted to capture solicited local or systemic ARs in the eDiary until resolution. Adverse reactions recorded in eDiaries beyond Day 7 should be reviewed either via telephone call or at the following study visit. All solicited ARs (local and systemic) will be considered causally related to injection.

Table 6: Solicited Adverse Reactions and Grades

Reaction	Grade 0	Grade 1	Grade 2	Grade 3	Grade 4*
Injection site pain	None	Does not interfere with activity	Repeated use of over-the-counter pain reliever > 24 hours or interferes with activity	Any use of prescription pain reliever or prevents daily activity	Requires emergency room visit or hospitalization
Injection site erythema (redness)	< 25 mm/ < 2.5 cm	25 - 50 mm/ 2.5 - 5 cm	51 - 100 mm/ 5.1 - 10 cm	> 100 mm/ > 10 cm	Necrosis or exfoliative dermatitis
Injection site swelling/induration (hardness)	< 25 mm/ < 2.5 cm	25 - 50 mm/ 2.5 - 5 cm	51 - 100 mm/ 5.1 - 10 cm	> 100 mm/ > 10 cm	Necrosis
Axillary (underarm) swelling or tenderness ipsilateral to the side of injection	None	No interference with activity	Repeated use of over-the-counter (non-narcotic) pain reliever > 24 hours or some interference with activity	Any use of prescription (narcotic) pain reliever or prevents daily activity	Requires emergency room visit or hospitalization
Headache	None	No interference with activity	Repeated use of over-the-counter pain reliever > 24 hours or some interference with activity	Significant; any use of prescription pain reliever or prevents daily activity	Requires emergency room visit or hospitalization
Fatigue	None	No interference with activity	Some interference with activity	Significant; prevents daily activity	Requires emergency room visit or hospitalization
Myalgia (muscle aches all over body)	None	No interference with activity	Some interference with activity	Significant; prevents daily activity	Requires emergency room visit or hospitalization
Arthralgia (joint aches in several joints)	None	No interference with activity	Some interference with activity	Significant; prevents daily activity	Requires emergency room visit or hospitalization

Reaction	Grade 0	Grade 1	Grade 2	Grade 3	Grade 4*
Nausea/vomiting	None	No interference with activity or 1-2 episodes/ 24 hours	Some interference with activity or > 2 episodes/24 hours	Prevents daily activity, requires outpatient intravenous hydration	Requires emergency room visit or hospitalization for hypotensive shock
Chills	None	No interference with activity	Some interference with activity not requiring medical intervention	Prevents daily activity and requires medical intervention	Requires emergency room visit or hospitalization
Fever (oral)	< 38.0°C<br>< 100.4°F	38.0 – 38.4°C<br>100.4 – 101.1°F	38.5 – 38.9°C<br>101.2 – 102.0°F	39.0 – 40.0°C<br>102.1 – 104.0°F	> 40.0°C<br>> 104.0°F
Rash*	No rash	Localized rash, without associated symptoms	Maculopapular rash, covering < 50% body surface area	Generalized urticarial, covering > 50% body surface area	Generalized exfoliative, ulcerative or bullous dermatitis, eg, Stevens-Johnson syndrome or erythema multiforme

* Grading for rash and Grade 4 events per Investigator assessment (with exception of fever).

Sources: Guidance for Industry – Toxicity Grading Scale for Heathy Adult and Adolescent Volunteers Enrolled in Preventive Vaccine Clinical Trials; tables for clinical abnormalities (DHHS 2007). Division of AIDS Grading the Severity of Adult and Pediatric Adverse Events (DHHS 2014).

In case of any rash episode observed within 7 days after study injection, the participant will be instructed to contact the study site within 24 hours. During participant evaluation, the investigator should categorize the rash as one of the following:

- Rash no longer present and history not consistent with urticaria.
- Rash no longer present but history is consistent with urticaria.
- Rash present but clinical findings are not consistent with urticaria. Alternative diagnosis should be specified as an AE.
- Rash present and clinical findings consistent with urticaria.

Any solicited AR that meets any of the following criteria must be entered into the participant's source document and must also be recorded as an AE in the participant's Adverse Event eCRF:

- Solicited local or systemic AR that results in a visit to an HCP (MAAE)
- Solicited local or systemic AR leading to the participant withdrawing from the study or the participant being withdrawn from the study by the investigator (AE leading to withdrawal)
- Solicited local or systemic AR lasting beyond 7 days post-injection
- Solicited local or systemic AR that leads to participant withdrawal from vaccine administration
- Solicited local or systemic AR that otherwise meets the definition of an SAE
- Solicited AR with a toxicity score of Grade 3 or greater

An unsolicited AE is any AE reported by the participant that is either not specified as a solicited AR in the protocol or is specified as a solicited AR in the protocol, but starts outside the protocol-defined post-injection period for reporting solicited ARs (ie, for the 7 days after each injection).

3.4.8.5 Pregnancy

Pregnancies occurring in participants after enrollment must be reported to Sponsor or designee within 72 hours of the site learning of its occurrence. If the participant agrees to submit this information, the pregnancy must be followed to determine the outcome, including spontaneous or voluntary termination, details of the birth, and the presence or absence of any birth defects, congenital abnormalities, or maternal and/or newborn complications. This follow-up should occur even if intended duration of the safety follow-up for the study has ended. Pregnancy report forms will be distributed to the study site to be used for this purpose. The investigator must immediately

(within 24 hours of awareness) report to the Sponsor any pregnancy resulting in an abnormal outcome according to the procedures described for SAEs (Section 3.4.8.8).

3.4.8.6 Definition and Reporting of Adverse Events Consistent with Anaphylaxis

All suspected cases of anaphylaxis should be recorded as MAAEs and reported as an SAE, based on criteria for medically important event, unless the event meets other serious criteria. As an SAE the event should be reported to the Sponsor or designee immediately and in all circumstances within 24 hours as per Section 3.4.8.8 Reporting AEs. The investigator will submit any updated anaphylaxis case data to the Sponsor within 24 hours of it being available. For reporting purposes, a participant who displays signs/symptoms consistent with anaphylaxis as shown below should be reported as a potential case of anaphylaxis. This is provided as general guidance for investigators and is based on the Brighton Collaboration case definition (Ruggeberg et al 2007).

Anaphylaxis is an acute hypersensitivity reaction with multi-organ-system involvement that can present as, or rapidly progress to, a severe life-threatening reaction. It may occur following exposure to allergens from a variety of sources.

Anaphylaxis is a clinical syndrome characterized by:

- sudden onset AND
- rapid progression of signs and symptoms AND
- involving two or more organ systems, as follows:

Skin/mucosal: urticaria (hives), generalized erythema, angioedema, generalized pruritus with skin rash, generalized prickle sensation, red and itchy eyes

Cardiovascular: measured hypotension, clinical diagnosis of uncompensated shock, loss of consciousness or decreased level of consciousness, evidence of reduced peripheral circulation

Respiratory: bilateral wheeze (bronchospasm), difficulty breathing, stridor, upper airway swelling (lip, tongue, throat, uvula, or larynx), respiratory distress, persistent dry cough, hoarse voice, sensation of throat closure, sneezing, rhinorrhea

Gastrointestinal: diarrhea, abdominal pain, nausea, vomiting

3.4.8.7 Eliciting and Documenting Adverse Events

The investigator is responsible for ensuring that all AEs and SAEs are recorded in the eCRF and reported to the Sponsor. Unsolicited AEs will be captured from Day 1 through 28 days after each dose up to Day 57 visit in Part A (Table 9), up to OL-Day 57 visit in Part B (Table 11 and Table 12), and up to 28 days after the last injection in Part C (Table 12). Both MAAEs and SAEs will be captured from blinded (Part A) and open-label (Part B) Day 1 throughout entire study duration (Day 394 for Part A, or 6 months post-last vaccination for Part B and C for all participants), as specified in the respective SOEs (Table 9, Table 11, and Table 12). Any AEs occurring before receipt of the vaccine will be analyzed separately from TEAEs.

At every study site visit or telephone contact, participants will be asked a standard question to elicit any medically-related changes in their well-being according to the scripts provided. Participants will also be asked if they have been hospitalized, had any accidents, used any new medications, changed concomitant medication regimens (both prescription and over-the-counter medications), or had any non-study vaccinations.

In addition to participant observations, data from clinical laboratory test results, physical examination findings, or other documents relevant to participant safety classified as an AE will be documented on the AE page of the eCRF.

3.4.8.8 Reporting Adverse Events

The investigator is responsible for reporting all AEs that are observed or reported during the study, regardless of their relationship to vaccine or their clinical significance. If there is any doubt as to whether a clinical observation is an AE, the event should be reported.

All unsolicited AEs reported or observed during the study will be recorded on the AE page of the eCRF. Information to be collected includes cohort, type of event, time of onset, investigator-specified assessment of severity and relationship to vaccine, time of resolution of the event, seriousness, as well as any required treatment or evaluations, and outcome. The unsolicited AEs resulting from concurrent illnesses, reactions to concurrent illnesses, reactions to concurrent medications, or progression of disease states must also be reported. All AEs will be followed until they are resolved or stable or judged by the investigator to be not clinically significant. The Medical Dictionary for Regulatory Activities (MedDRA) will be used to code all unsolicited AEs.

Any medical condition that is present at the time that the participant is screened but does not deteriorate should not be reported as an unsolicited AE. However, if it deteriorates at any time during the study, it should be recorded as an unsolicited AE.

Any AE considered serious by the investigator or that meets SAE criteria (Section 3.4.8.3) must be reported to the Sponsor immediately (within 24 hours of becoming aware of the SAE). The investigator will assess whether there is a reasonable possibility that the vaccine caused the SAE. The Sponsor will be responsible for notifying the relevant regulatory authorities of any SAE as outlined in the 21 US CFR Parts 312 and 320. The investigator is responsible for notifying the institutional review board (IRB) directly.

If the eCRF is unavailable at the time of the SAE, the following contact information is to be used for SAE reporting:

- SAE Mailbox: Safety_Moderna@iqvia.com
- SAE Hotline (USA and Canada): +1-866-599-1341
- SAE Fax line (USA and Canada): +1-866-599-1342

3.4.8.9 Assessment of Severity

An event is defined as “serious” when it meets at least one of the predefined outcomes as described in the definition of an SAE (Section 3.4.8.3), NOT when it is rated as severe.

The severity (or intensity) of an AR or AE refers to the extent to which it affects the participant’s daily activities and will be classified as mild (Grade 1), moderate (Grade 2), severe (Grade 3), or Grade 4 using the following criteria:

- Mild (Grade 1): These events do not interfere with the participant’s daily activities.
- Moderate (Grade 2): These events cause some interference with the participant’s daily activities but do not require medical intervention.
- Severe (Grade 3): These events prevent the participant’s daily activity and require medical intervention.
- Grade 4 (solicited ARs): These events require an emergency room visit or hospitalization.

Changes in the severity of an AE should be documented to allow an assessment of the duration of the event at each level of intensity to be performed. An AE characterized as intermittent requires documentation of onset and duration of each episode.

The Toxicity Grading Scale for Healthy Adult and Adolescent Volunteers Enrolled in Preventive Vaccine Clinical Trials (DHHS 2007) will be used to categorize local and systemic solicited ARs (Table 6), clinical laboratory test results, and vital sign measurements observed during this study. Specific criteria for clinical and laboratory abnormalities are presented in Appendix 2 (Table 13 and Table 14, respectively) and will be graded if outside of the reference range for the laboratory utilized.

3.4.8.10 Assessment of Causality

The investigator's assessment of an AE's relationship to vaccine is part of the documentation process but is not a factor in determining what is or is not reported in the study.

The investigator will assess causality (ie, whether there is a reasonable possibility that the vaccine caused the event) for all AEs and SAEs. The relationship will be characterized using the following classification:

- Not related: There is not a reasonable possibility of a relationship to the investigational product. Participant did not receive the investigational product OR temporal sequence of the AE onset relative to administration of the investigational product is not reasonable OR the AE is more likely explained by another cause than the investigational product.
- Related: There is a reasonable possibility of a relationship to the investigational product. There is evidence of exposure to the investigational product. The temporal sequence of the AE onset relative to the administration of the investigational product is reasonable. The AE is more likely explained by the investigational product than by another cause.

3.4.8.11 Follow-up of Adverse Events

All AEs, SAEs, and MAAEs must be reported in detail on the appropriate page of the eCRF and followed until the event is resolved or stable or judged by the investigator to be not clinically significant.

3.4.9 Vital Sign Measurements

Vital sign measurements will include systolic and diastolic blood pressure, heart rate, respiratory rate, and body temperature (preferred route is oral). The participant will be seated for at least 5 minutes before all measurements are taken. Vital signs will be measured at the time points indicated in the Schedule of Events (Table 9, Table 11, and Table 12). In all parts, blinded and open label, on Day 1 and Day 29 (as applicable), vital sign measurements will be collected once before vaccine administration and at least 30 minutes (Part A) and 30 minutes (Parts B and C) after vaccine administration (before participants are discharged from the study site).

Febrile participants at Day 1 and Day 29 (Parts A, B, and C) visits (fever is defined as a body temperature ≥ 38.0°C/100.4°F) may be rescheduled within the relevant window periods. Afebrile participants with minor illnesses may be injected at the discretion of the investigator.

When procedures overlap and are scheduled to occur at the same time point, the order of procedures should be vital sign measurements and then the blood collection.

If any of the vital sign measurements meet the toxicity grading criteria for clinical abnormalities (Table 13) of Grade 3 or greater, the abnormal value and grade will be documented on the AE page of the eCRF (unless there is another known cause of the abnormality that would result in an AE classification). The investigator will continue to monitor the participant with additional assessments until the vital sign value has reached the reference range, returns to the vital sign value at baseline, is considered stable, or until the investigator determines that follow-up is no longer medically necessary.

3.4.10 Physical Examinations

A symptom-directed physical examination, including height and weight, will be performed at scheduled time points as indicated in the Schedule of Events (Table 9, Table 11, and Table 12). The physical examination may include assessment of skin, head, ears, eyes, nose, throat, neck, thyroid, lungs, heart, cardiovascular, abdomen, lymph nodes, and musculoskeletal system/extremities. Any clinically significant finding identified during a study visit should be reported as a MAAE.

Symptom-directed physical examinations may be performed at other timepoints at the discretion of the investigator. On each injection day before injection and again 7 days after injection, the arm receiving the injection should be examined and the associated lymph nodes should be evaluated.

Body mass index will be calculated at the Screening Visit (Day 0) only.

4 STATISTICAL ANALYSIS PLAN

This section summarizes the planned statistical analysis strategy and procedures for the study. The details of statistical analysis will be provided in the statistical analysis plan (SAP), which will be finalized before the clinical database lock for the study and treatment unblinding. If, after the study has begun, but prior to any unblinding, changes are made to primary and/or key secondary objectives/hypotheses, or the statistical methods related to those hypotheses, then the protocol will be amended (consistent with ICH Guideline E9). Changes to other secondary or exploratory analyses made after the protocol has been finalized, along with an explanation as to when and why they occurred, will be listed in the SAP or CSR for the study. Ad hoc exploratory analyses, if any, will be clearly identified in the CSR.

4.1 Blinding and Responsibility for Analyses

Blinding during the Part A of the study will be managed as described in Section 3.3.5. The Sponsor Biostatistics department or designee will generate the randomized allocation schedule(s) for study treatment assignment. Randomization will be implemented via an IRT.

Details regarding unblinded SMC review are described in Section 6.1.

A limited number of Sponsor and CRO personnel will be unblinded to perform the primary analysis and prepare a final CSR after participants have completed Day 57 visit study procedures in Part A (Section 3.3.5). This primary analysis may be performed when all participants in Cohort 1 and the Cohort 2 sentinel group have completed Day 57 study procedures and/or when all participants in Cohort 1 and Cohort 2 have completed Day 57 study procedures. The study site staff, investigators, study monitors, and participants will remain blinded until the initiation of Part B.

Planned study analyses and data presentation for unblinded SMC review are described in Section 4.7 and Section 6.1, respectively. At each analysis, pre-identified Sponsor members will be unblinded to review treatment level results as defined in the study Data Blinding Plan. Unblinded data presentation or analysis for SMC review will be handled by the CRO unblinded team of statisticians and programmers, who are not involved in study design. A strict firewall between the CRO blinded and unblinded teams will be maintained during study conduct. Sponsor personnel who have access to review unblinded results will be documented. Sponsor and CRO personnel involved in the ongoing review and oversight of safety and immunogenicity will remain blinded until the initiation of Part B, as will study investigators and personnel at the study sites. The results of the primary analysis will not be shared with the investigators before completion of Part A of the study.

Part B and Part C will be open label.

4.2 Hypothesis Testing

There is no hypothesis testing in this study.

4.3 Analysis Endpoints

4.3.1 Part A, Blinded

4.3.1.1 Primary Endpoints

4.3.1.1.1 Primary Safety Endpoints

The primary safety objective will be evaluated by the following safety endpoints:

- Solicited local and systemic ARs through 7 days after each injection.
- Unsolicited AEs through 28 days after each injection.
- MAAEs through the entire study period.
- SAEs throughout the entire study period.
- Safety laboratory abnormalities at Day 29 and Day 57 (Cohort 2 only).
- Vital sign measurements and physical examination findings.

4.3.1.1.2 Primary Immunogenicity Endpoint

- Level of SARS-CoV-2-specific bAb measured by ELISA on Day 1, Day 29 (Month 1), Day 43, Day 57 (Month 2), Day 209 (Month 7), and Day 394 (Month 13), as applicable.

4.3.1.2 Secondary Endpoints

The secondary objective will be evaluated by the following endpoints:

- Titer of SARS-CoV-2-specific nAb on Day 1, Day 29 (Month 1), Day 43, Day 57 (Month 2), Day 209 (Month 7), and Day 394 (Month 13), as applicable.
- Seroconversion on Day 29 (Month 1), Day 43, Day 57 (Month 2), Day 209 (Month 7), and Day 394 (Month 13), as applicable.

4.3.1.3 Exploratory Endpoints

The exploratory endpoints are the following:

- Serum titers of S protein-specific binding Ig assessed by class and subclass and nAb in serum.
- Relative amounts or profiles of S protein-specific bAb and specific nAb levels/titers in serum.
- Clinical severity and immune response of participants infected by SARS-CoV-2.
- Number of cases and incidence of confirmed SARS-CoV-2 infection using an assay designed to detect non-vaccine antigens of SARS-CoV-2.

4.3.2 Part B, Open Label

4.3.2.1 Primary Endpoints (Part B, Open Label)

4.3.2.1.1 Primary Safety Endpoints

The primary safety objective will be evaluated by the following safety endpoints:

- Solicited local and systemic ARs through 7 days after each injection.
- Unsolicited AEs through 28 days after each injection.
- MAAEs through the entire study period.
- SAEs throughout the entire study period.
- Vital sign measurements and physical examination findings.

4.3.2.1.2 Primary Immunogenicity Endpoint

- For participants receiving 100 µg of mRNA-1273 as 2 doses 28 days apart: Level of SARS-CoV-2-specific bAb measured by ELISA on OL-Day 1, OL-Day 8, OL-Day 15, OL-Day 29 (OL-Month 1), OL-Day 57 (OL-Month 2), and OL-Day 209 (OL-Month 7).
- For participants receiving 50 µg of mRNA-1273 as a single booster: Level of SARS-CoV-2-specific bAb measured by ELISA on OL-Day 1, OL-Day 8, OL-Day 15, OL-Day 29 (OL-Month 1), OL-Day 57 (OL-Month 2), and OL-Day 181 (OL-Month 6).

4.3.2.2 Secondary Endpoints (Part B, Open Label)

The secondary objective will be evaluated by the following endpoints:

- For participants receiving 100 µg of mRNA-1273 as 2 doses 28 days apart: Titer of SARS-CoV-2-specific nAb on OL- Day 1, OL-Day 8, OL- Day 15, OL- Day 29 (OL-Month 1), OL- Day 57 (OL-Month 2), and OL- Day 209 (OL-Month 7).
- For participants receiving 50 µg of mRNA-1273 as a single booster: Titer of SARS-CoV-2-specific nAb on OL-Day 1, OL-Day 8, OL-Day 15, OL-Day 29 (OL-Month 1), OL-Day 57 (OL-Month 2), and OL-Day 181 (OL-Month 6).
- For participants receiving 100 µg of mRNA-1273 as 2 doses 28 days apart: Seroconversion on OL-Day 8, OL-Day 15, OL-Day 29 (OL-Month 1), OL-Day 57 (OL-Month 2), and OL-Day 209 (OL-Month 7).
- For participants receiving 50 µg of mRNA-1273 as a single booster: Seroconversion on OL-Day 8, OL-Day 15, OL-Day 29 (OL-Month 1), OL-Day 57 (OL-Month 2), and OL-Day 181 (OL-Month 6), and the proportion of participants with a ≥2-, ≥3-, or ≥4-fold rise in antibody titer from open-label baseline.

4.3.2.3 Exploratory Endpoints (Part B, Open Label)

The exploratory endpoints are the following:

- Serum titers of S protein-specific binding Ig assessed by class and subclass and nAb in serum.
- Relative amounts or profiles of S protein-specific bAb and specific nAb levels/titers in serum.
- Clinical severity and immune response of participants infected by SARS-CoV-2.
- Number of cases and incidence of confirmed SARS-CoV-2 infection using an assay designed to detect non-vaccine antigens of SARS-CoV-2.

4.3.3 Part C, Open Label

4.3.3.1 Primary Endpoints (Part C, Open Label)

4.3.3.1.1 Primary Safety Endpoints

The primary safety objective will be evaluated by the following safety endpoints:

- Solicited local and systemic ARs through 7 days after injection.
- Unsolicited AEs through 28 days after injection.
- MAAEs through the entire study period.
- SAEs throughout the entire study period.
- Vital sign measurements and physical examination findings.

4.3.3.1.2 Primary Immunogenicity Endpoints (Part C, Open Label)

The primary objective will be evaluated by the following endpoints:

- For participants receiving 2 dose levels (20 and 50 μg) of mRNA-1273.351 or one dose level (50 μg total) of mRNA-1273/mRNA-1273.351 mixture as a single booster: titer of serum neutralizing antibody against SARS-CoV2 as measured by pseudovirus (and/or live virus) using the B.1.351 and D614G variant sequences of SARS-CoV-2 protein (and/or variant strains) on OL-Day 1, OL-Day 8, OL-Day 15, and OL-Day 29 (OL-Month 1), OL-Day 57 (OL-Month 2), and OL-Day 181 (OL-Month 6).
- For participants receiving 2 dose levels (20 and 50 μg) of mRNA-1273.351 or one dose level (50 μg total) of mRNA-1273/mRNA-1273.351 mixture as a single booster: seroconversion on OL-Day 8, OL-Day 15, and OL-Day 29 (OL-Month 1), OL-Day 57 (OL Month 2), and OL-Day 181 (OL-Month 6) and the proportion of participants with a ≥2-, ≥3-, or ≥4-fold rise in antibody titer from the open-label baseline.

4.3.3.2 Secondary Immunogenicity Endpoint

- For participants receiving 2 dose levels (20 and 50 μg) of mRNA-1273.351 or one dose level (50 μg total) of mRNA-1273/mRNA-1273.351 mixture as a single booster: level of SARS-CoV-2-specific serum binding antibody against the B.1.351 and D614G variant sequences of SARS-CoV-2 protein on OL-Day 1, OL-Day 8, OL-Day 15, OL-Day 29 (OL-Month 1), OL-Day 57 (OL-Month 2), and OL-Day 181 (OL-Month 6).

4.3.3.3 Exploratory Endpoints (Part C, Open Label)

The exploratory endpoints are the following:

- Serum titers of S protein-specific binding Ig assessed by class and subclass and nAb in serum.
- Relative amounts or profiles of S protein-specific bAb and specific nAb levels/titers in serum.
- Clinical severity and immune response of participants infected by SARS-CoV-2.
- Number of cases and incidence of confirmed SARS-CoV-2 infection using an assay designed to detect non-vaccine antigens of SARS-CoV-2.

4.4 Analysis Populations

4.4.1 Randomized Set

The Randomized Set consists of all participants who are randomly assigned in the study, regardless of the participants' treatment status in the study. This analysis set will be used for analyses of Part A.

4.4.2 Solicited Safety Set

The Solicited Safety Set consists of all participants who are randomly assigned and received any study injection, and contribute any solicited AR data; ie, have at least one post-baseline solicited safety (eDiary) assessment. The Solicited Safety Set will be used for the analyses of solicited ARs and participants will be included in the treatment group corresponding to the study injection they actually received. This analysis set will be used for analyses in Part A, Part B and Part C.

4.4.3 Safety Set

The Safety Set consists of all randomly assigned participants who received any study injection. The Safety Set will be used for analysis of safety except for the solicited ARs. Participants will be included in the treatment group corresponding to the study injection they actually received for the analysis of safety data using the Safety Set. This analysis set will be used for analyses in Part A, Part B, and Part C.

4.4.4 Full Analysis Set

The Full Analysis Set (FAS) consists of all randomly assigned participants who a) receive any study injection, b) have baseline (Day 1 or OL-Day 1, as appropriate) data available for those analyses that require baseline data, and c) have at least one post-injection assessment for the analysis endpoint. Participants will be included in the treatment group to which they were randomly assigned. This analysis set will be used for analyses in Part A, Part B, and Part C.

4.4.5 Per-Protocol Set

The Per-Protocol (PP) Set consists of all FAS participants who meet all of the following criteria:

- Complied with the injection schedule
- Complied with the timings of immunogenicity blood sampling to have post-injection results available for at least one assay component corresponding to the immunogenicity analysis objective
- Did not have SARS-CoV-2 infection
- Have had no major protocol deviations that impact immune response during the period corresponding to the immunogenicity analysis objective

The PP Set will serve as the primary population for the analysis of immunogenicity data in Part A, Part B, and Part C of this study. Participants will be included in the treatment group to which they were randomly assigned.

4.5 Sample Size Determination

There is no hypothesis testing in this study. The number of proposed participants is considered sufficient to provide a descriptive summary of the safety and immunogenicity of different dose levels of mRNA-1273.

In the blinded Part A, approximately 600 participants will be randomly assigned in a 1:1:1 ratio to mRNA-1273 50 µg, mRNA-1273 100 µg, or placebo. A total of 400 participants will receive mRNA-1273, 200 participants in each dose level, or 100 participants in each age cohort and dose level. Table 7 presents the 95% confidence interval (CI) for 1 participant with an AE and the lowest AE rate detectable with at least 95% probability for each selected sample size. The 2-sided 95% CI was calculated using the Clopper-Pearson method for one proportion in SAS 9.4 software. The 2-sided 95% CI is estimated (0.01%, 1.4%) at sample size of 400 with 1 participant reporting an AE. Furthermore, a sample size of 400 has at least a 95% probability to observe at least

1 participant with an AE at a true 0.75% AE rate. Participants who received any injection in Part A will have the opportunity to enter Part B provided that they meet the eligibility criteria.

In the open-label Part C, approximately 60 participants from Moderna's mRNA-1273-P301 COVE study will be enrolled and will be assigned to receive a single IM injection of mRNA-1273.351 (20 µg or 50 µg) or mRNA-1273/mRNA-1273.351 mixture (50 µg total). The enrollment and vaccination in each study arm will be sequential. The first 20 participants will be enrolled and dosed on OL-D1 with the mRNA-1273.351 50 µg dose. Upon completion of the first 50 µg dose arm, 20 participants will be enrolled in the second arm and will receive the mRNA-1273/mRNA-1273.351 mixture (50 µg total dose). Following completion of the second arm, 20 participants will be enrolled and dosed with the mRNA-1273.351 20 µg dose.

Table 7: 95% Confidence Interval for One Participant with AE and the Lowest Detectable Incidence Rate at 95% Probability in Selected Sample Size

Sample Size Receiving mRNA-1273	Rate and 95% CI (%) at One Participant with AE			Lowest Detectible Rate (%) with ≥95% Probability
	AE Rate	Lower CI	Upper CI	
100	1.00	0.03	5.45	2.95
200	0.50	0.01	2.75	1.49
400	0.25	0.01	1.38	0.75

Abbreviations: AE = adverse event; CI = confidence interval.

4.6 Statistical Methods

Data from Part A, Part B, and Part C will be presented separately as applicable.

For Part A and Part B of the study, there are 2 age cohorts: Cohort 1 with 300 participants (≥ 18 to < 55 years old) and Cohort 2 with 300 participants (≥ 55 years old). All analyses will be performed by treatment group overall (for the 2 cohorts combined) and for the 2 cohorts separately, unless specified otherwise.

4.6.1 Summary of Baseline Characteristics and Demographics

Demographic variables (eg, age, height, weight, and BMI) and baseline characteristics will be summarized by treatment group for each age cohort (when appropriate) by descriptive statistics (mean, standard deviation for continuous variables, and number and percentage for categorical variables).

4.6.2 Safety Analyses

Safety and reactogenicity will be assessed by clinical review of all relevant parameters including solicited ARs (local and systemic events), unsolicited AEs, SAEs, MAAEs, AEs leading to discontinuation, safety laboratory test results (Part A only), vital signs, and physical examination findings.

Solicited ARs and unsolicited AEs will be coded by system organ class and preferred term according to the MedDRA for adverse reaction terminology. The Toxicity Grading Scale for Healthy Adult and Adolescent Volunteers Enrolled in Preventive Vaccine Clinical Trials (DHHS 2007) is used in this study with modification for rash, solicited ARs, and vital signs (Table 6).

Rash will be graded as:

- Grade 0 = no rash
- Grade 1 = localized without associated symptoms
- Grade 2 = maculopapular rash covering < 50% body surface area
- Grade 3 = urticarial rash covering > 50% body surface area
- Grade 4 = generalized exfoliative, ulcerative, or bullous dermatitis

All safety analyses will be based on the Safety Set, except summaries of solicited ARs which will be based on the Solicited Safety Set. All safety analyses will be provided by age cohort (for Part A and Part B) unless otherwise specified.

The number and percentage of participants with any solicited local AR, with any solicited systemic AR, and with any solicited AR during the 7-day follow-up period after each injection will be summarized. A 2-sided 95% exact CI using the Clopper-Pearson method will be provided for the percentage of participants with any solicited AR.

Number and percentage of participants with unsolicited AEs, SAEs, MAAEs, Grade 3 or higher AEs, and AEs leading to discontinuation from study vaccine or participation in the study will be summarized.

Number of events of solicited ARs, unsolicited AEs/SAEs, and MAAEs will be reported in summarization tables accordingly.

For all other safety parameters, descriptive summary statistics will be provided. Table 8 summarizes the analysis strategy for safety parameters.

Table 8: Analysis Strategy for Safety Parameters

Safety Endpoint	Number and Percentage of Participants, Number of Events	95% CI
Any Solicited AR (overall and by local, systemic)	X	X
Any Unsolicited AE	X	
Any SAE	X	
Any Unsolicited MAAE	X	
Any Unsolicited Treatment-Related AE	X	
Any Treatment-Related SAE	X	
Discontinuation due to AE	X	
Any Grade 3 and above AE	X	
Any Treatment-Related Grade 3 and above AE	X	

Abbreviations: AE = adverse event; AR = adverse reaction; CI = confidence interval; MAAE = medically attended adverse event; SAE = serious adverse event.
Notes: 95% CI using the Clopper-Pearson method, X = results will be provided. Unsolicited AEs will be summarized by system organ class and preferred term coded by the Medical Dictionary for Regulatory Activities.

For treatment-emergent safety laboratory tests results in Part A and Part B of the study, the raw values and change from baseline values will be summarized by age cohort, treatment group, and visit at each timepoint.

The number and percentage of participants who have chemistry, hematology, coagulation, and vital signs results below or above the laboratory normal ranges will be tabulated by timepoint.

Data from Part C will be analyzed similarly.

Further details will be described in the SAP.

4.6.3 Immunogenicity Analyses

The analyses of immunogenicity will be based on the PP Set. For each age cohort (for Part A and Part B), if the number of participants in the FAS and PP Set differ (defined as the difference divided by the total number of participants in the PP Set) by more than 10%, supportive analyses of immunogenicity may be conducted using the FAS.

For the primary immunogenicity endpoint (secondary immunogenicity endpoint in Part C) (Section 4.3.1.1.2), geometric mean (GM) of specific bAb with corresponding 95% CI at each timepoint and geometric mean fold-rise (GMFR) of specific bAb with corresponding 95% CI at each post-baseline timepoint over pre-injection baseline will be provided by treatment group and age cohort (for Part A and Part B). Descriptive summary statistics including median, minimum, and maximum will also be provided.

For the secondary immunogenicity endpoint (primary immunogenicity endpoint in Part C) (Section 4.3.1.2), geometric mean titer (GMT) of specific nAb with corresponding 95% CI at each timepoint and GMFR of specific nAb with corresponding 95% CI at each post-baseline timepoint over pre-injection baseline will be provided by treatment group and age cohort (for Part A and Part B). Descriptive summary statistics including median, minimum, and maximum will also be provided. For summarizations of GMT values, antibody values reported as below the LLOQ will be replaced by 0.5 × LLOQ. Values that are greater than the upper limit of quantification (ULOQ) will be converted to the ULOQ.

The number and percentage of participants with fold-rise ≥ 2, fold-rise ≥ 3, and fold-rise ≥ 4 of serum SARS-CoV-2-specific nAb titers and participants with seroconversion from baseline will be provided with 2-sided 95% CI using the Clopper-Pearson method at each post-baseline timepoint. Seroconversion at a participant level is defined as a change of nAb titer from below the LLOQ to equal to or above LLOQ, or a 4-times or higher titer ratio in participants with pre-existing nAb titers.

Exploratory analyses of each dose level of mRNA-1273 versus placebo (for Part A) on bAb and nAb levels/titers may be performed.

Data from Part C will be analyzed similarly for each strain included in each assay.

Details for immunogenicity analyses on data collected in the open-label phase will be provided in the SAP.

4.6.4 Exploratory Analyses

Exploratory analyses may include the following:

- Descriptive summaries of the relative proportions of S protein-specific serum Igs and nAb during the study. Subclass analysis of specific IgG may be performed.
- Descriptive summaries of the ratio or profile of specific bAb relative to nAb in serum during the study.
- Descriptive summaries of clinical profile and immunologic endpoints to characterize participants with SARS-CoV-2 infection during the study.

4.7 Study Analyses

4.7.1 Primary Study Analysis for Blinded Part A Only

A primary analysis of safety and immunogenicity data will be performed after participants have completed Day 57 study procedures. This primary analysis may be performed when all participants in Cohort 1 and the Cohort 2 sentinel group have completed Day 57 study procedures and/or when all participants in Cohort 1 and Cohort 2 have completed Day 57 study procedures. All data relevant to the primary study analysis through the Day 57 visit will be cleaned (ie, data that are as clean as possible) and locked. Results of this analysis will be presented in a final CSR, including individual listings.

4.7.2 Analysis at End of Blinded Part A Only

An analysis of safety and immunogenicity data will be performed after all participants have completed Part A of the study. All data collected in Part A of the study will be cleaned (ie, data that are as clean as possible) and locked and a report may be generated as needed.

4.7.3 Interim Study Analysis for Open-Label Part B Only

An interim analysis of the safety and immunogenicity data in Part B of the study may be performed after participants have completed the OL-Day 29 and/or the OL-Day 57 study procedures. This analysis may be performed when all participants have completed OL-Day 29 and/or OL-Day 57 study procedures, or on a subset of participants who received a single booster dose at OL-Day 1 when they have completed OL-Day 29 and/or OL-Day 57 study procedures. All data collected in Part B of the study will be cleaned (ie, data that are as clean as possible) and locked and a report may be generated as needed. Results of this analysis may be presented in a CSR.

4.7.4 Interim Study Analysis for Open-Label Part C Only

An interim analysis of the safety and immunogenicity data in Part C of the study may be performed after participants have completed the OL-D8, OL-D15, OL-Day 29 and/or the OL-Day 57 study procedures. All data collected in Part C of the study will be cleaned (ie, data that are as clean as possible) and locked and a report may be generated as needed. Results of this analysis may be presented in a CSR.

4.7.5 Additional Study Analyses

Additional analyses may be performed to support regulatory request as appropriate.

4.7.6 End of Study Analysis

The EOS analysis of all endpoints will be performed after all participants have completed Month 13 (Blinded Part A), OL-M6 and OL-M7 (Open-Label Part B), or OL-Month 6 (Part C) study procedures and after the database is cleaned and locked. Results of this analysis will be presented in an EOS CSR, including individual listings.

Additional information can be found in the SAP.

4.8 Data Quality Assurance

All aspects of the study will be monitored for compliance with applicable government regulations with respect to current ICH harmonized tripartite guideline E6(R2): GCP and current SOPs. The eCRFs will be utilized and accessed through iMedidata® via the internet. This EDC system is validated and compliant with US Title 21 of CFR Part 11. Each person involved with the study will have an individual identification code and password that allow for record traceability. There may be an internal quality review audit of the data and additional reviews by the clinical monitor.

Due to safety review requirements, study sites must follow the data entry and availability instructions provided by the Sponsor in the study readiness trainings. As a quality measure, timeliness of data entry and data query resolution will be followed closely. Other issues of data quality that may hinder safety review or pose a concern with patient safety will be brought to the attention of the Sponsor or CRO, with appropriate awareness to the SMC if needed.

5 INVESTIGATOR OBLIGATIONS

The following administrative items are meant to guide the investigator in the conduct of the study and may be pursuant to change based on industry and government SOPs, working practice documents, or guidelines. Changes will be reported to the IRB but will not result in protocol amendments.

5.1 Confidentiality

All laboratory specimens, evaluation forms, reports, and other records will be identified in a manner designed to maintain participant confidentiality. All records will be kept in a secure storage area with limited access. Clinical information will not be released without the written permission of the participant, except as necessary for monitoring and auditing by the Sponsor, its designee, relevant regulatory authority, or the IRB.

The investigator and all employees and coworkers involved with this study may not disclose or use for any purpose other than performance of the study, any data, record, or other unpublished, confidential information disclosed to those individuals for the purpose of the study. Prior written agreement from the Sponsor or its designee must be obtained for the disclosure of any said confidential information to other parties.

5.2 Institutional Review

Federal regulations and the ICH E6(R2) guidelines require that approval be obtained from an IRB before participation of human participants in research studies. Before study onset, the protocol, informed consent, advertisements to be used for the recruitment of study participants, and any other written information regarding this study to be provided to the participant must be approved by the IRB. Documentation of all IRB approvals and of the IRB compliance with the ICH E6(R2) guidelines will be maintained by the site and will be available for review by the Sponsor or its designee.

All IRB approvals should be signed by the IRB chairman or designee and must identify the IRB name and address, the clinical protocol by title or protocol number or both, and the date approval or a favorable opinion was granted.

5.3 Participant Consent

Written informed consent in compliance with US Title 21 CFR Part 50 shall be obtained from each participant before he or she enters the study or before any unusual or nonroutine procedure that involves risk to the participant is performed. If any institution-specific modifications to study-

related procedures are proposed or made by the site, the consent should be reviewed by the Sponsor or its designee or both before IRB submission. Once reviewed, the investigator will submit the ICF to the IRB for review and approval before the start of the study. If the ICF is revised during the course of the study, all active participating participants must sign the revised form.

Before recruitment and enrollment, each prospective participant will be given a full explanation of the study, be allowed to read the approved ICF, and be given answers to any questions. Once the investigator is assured that the participant understands the implications of participating in the study, the participant will be asked to give his or her consent to participate in the study by signing the ICF. Separate counseling and consent may be provided for HIV testing as applicable per local laws or regulations.

The ICF will also explain that excess serum from immunogenicity testing may be used for future research, which may be performed at the discretion of the Sponsor to further characterize the immune response to SARS-CoV-2, additional assay development, and the immune response across the coronaviruses.

The investigator or designee will provide a copy of the ICF to the participant. The original form shall be maintained in the participant's medical records at the site.

5.4 Study Reporting Requirements

By participating in this study, the investigator agrees to submit reports of SAEs to Sponsor according to the timeline and method outlined in this protocol. In addition, the investigator agrees to submit annual reports to his or her IRB as appropriate.

5.5 Financial Disclosure and Obligations

The investigator is required to provide financial disclosure information to allow the Sponsor to submit the complete and accurate certification or disclosure statements required under 21 CFR 54. In addition, the investigator must provide to the Sponsor a commitment to promptly update this information if any relevant changes occur during the course of the investigation and for 1 year following the completion of the study.

Neither the Sponsor, the CRO, nor the study site is financially responsible for further testing or treatment of any medical condition that may be detected during the screening process. In addition, in the absence of specific arrangements, neither the Sponsor, the CRO, nor the study site is financially responsible for further treatment of the disease under study.

5.6 Investigator Documentation

Prior to beginning the study, the investigator will be asked to comply with ICH E6(R2) 8.2 and Title 21 of the CFR by providing the following essential documents, including but not limited to:

- IRB approval,
- An original investigator-signed investigator agreement page of the protocol,
- Form FDA 1572, fully executed, and all updates on a new fully executed Form FDA 1572,
- Curriculum vitae for the principal investigator and each subinvestigator listed on Form FDA 1572. Current licensure must be noted on the curriculum vitae. The curriculum vitae will be signed and dated by the principal investigators and subinvestigators at study start-up, indicating that they are accurate and current,
- Financial disclosure information to allow the Sponsor to submit complete and accurate certification or disclosure statements required under 21 CFR 54. In addition, the investigators must provide to the Sponsor a commitment to promptly update this information if any relevant changes occur during the course of the investigation and for 1 year after the completion of the study,
- An IRB-approved ICF, samples of site advertisements for recruitment for this study, and any other written information about this study that is to be provided to the participant, and
- Laboratory certifications and reference ranges for any local laboratories used by the site, in accordance with 42 CFR 493.

5.7 Study Conduct

The investigator agrees that the study will be conducted according to the principles of ICH E6(R2). The investigator will conduct all aspects of this study in accordance with all national, state, and local laws or regulations. The study will be conducted in compliance with the protocol, current GCP guidelines – adopting the principles of the Declaration of Helsinki – and all applicable regulatory requirements.

5.8 Data Collection

5.8.1 Case Report Forms and Source Documents

As part of the responsibilities assumed by participating in the study, the investigator agrees to maintain adequate case histories for participants treated as part of the research under this protocol. The investigator agrees to maintain accurate eCRFs and source documentation as part of the case histories. These source documents may include laboratory reports and similar sources.

Electronic case report forms are accessed through iMedidata® via the internet. This EDC system is validated and compliant with 21 CFR 11. Each person involved with the study will have an individual identification code and password that allows for record traceability. Thus, the system, and subsequently any investigative reviews, can identify coordinators, investigators, and individuals who have entered or modified records, as well as the time and date of any modifications. There may be internal quality review audit of the data and additional reviews by the clinical monitor.

Each eCRF is presented as an electronic copy, allowing data entry by site personnel, who can add and edit data, add new participants, identify and resolve discrepancies, and view records. This system provides immediate direct data transfer to the database, as well as immediate detection of discrepancies, enabling site coordinators to resolve and manage discrepancies in a timely manner.

5.9 Adherence to Protocol

The investigator agrees to conduct the study as outlined in this protocol in accordance with ICH E6(R2) and all applicable guidelines and regulations.

5.10 Reporting Adverse Events

By participating in this study, the investigator agrees to submit reports of SAEs according to the timeline and method outlined in this protocol. In addition, the investigator agrees to submit annual reports to his or her IRB as appropriate. The investigator also agrees to provide the Sponsor with an adequate report, if applicable, shortly after completion of the investigator's participation in the study.

5.11 Investigator's Final Report

Upon completion of the study, the investigator, where applicable, should inform the institution; the investigator/institution should provide the IRB with a summary of the study's outcome, and the Sponsor and regulatory authority(ies) with any reports required.

5.12 Records Retention

Essential documents should be retained until at least 2 years after the last approval of a marketing application in an ICH region and until there are no pending or contemplated marketing applications in an ICH region or at least 2 years have elapsed since the formal discontinuation of clinical development of the vaccine. These documents should be retained for a longer period, however, if required by the applicable regulatory requirements or by an agreement with the Sponsor. It is the Sponsor's responsibility to inform the investigator/institution as to when these documents no longer need to be retained.

5.13 Publications

After completion of the study, the data may be considered for reporting at a scientific meeting or for publication in a scientific journal. In these cases, the Sponsor will be responsible for these activities and will work with the investigators to determine how the manuscript is written and edited, the number and order of authors, the publication to which it will be submitted, and other related issues. The Sponsor has final approval authority over all such issues.

Data are the property of the Sponsor and cannot be published without their prior authorization, but data and publication thereof will not be unduly withheld.

6 STUDY MANAGEMENT

6.1 Monitoring

Ongoing safety monitoring will be performed in a blinded manner by the CRO's medical monitor, the Sponsor's medical monitor, and the individual site investigators throughout the study.

6.1.1 Safety Monitoring Committee

Safety oversight will be under the direction of an SMC composed of external independent consultants with relevant expertise. Members of the SMC will be independent from the study conduct and free of conflict of interest. The SMC will meet on a regular basis to assess safety throughout the study conduct. The SMC will operate under the rules of an approved charter that will be written and reviewed at the organizational meeting of the SMC. Details regarding the SMC composition, responsibilities, procedures, and frequency of data review will be defined in its charter.

The SMC will convene on an ad hoc basis if any of the pause rules, described in Section 3.3.2.1, are met. The SMC will review all available unblinded study data to adjudicate any potential study pauses and make recommendations on further study conduct, including requesting additional information, recommending stopping the study, recommending changes to study conduct and/or the protocol, or recommending additional operational considerations due to safety issues that arise during the study.

6.1.2 Monitoring of the Study

The study monitor, as a representative of the Sponsor, is obligated to follow the study closely. In doing so, the monitor will visit the investigator and study facility at periodic intervals, in addition to maintaining necessary telephone and letter contact. The monitor will maintain current personal knowledge of the study through observation, review of study records and source documentation, and discussion of the conduct of the study with the investigator and staff. The monitor will be blinded to dose assignments. A separate unblinded study monitor will be responsible for vaccine accountability.

All aspects of the study will be carefully monitored by the Sponsor or its designee for compliance with applicable government regulation with respect to current ICH E6(R2) guidelines and SOPs.

6.1.3 Inspection of Records

The investigator and institution involved in the study will permit study-related monitoring, audits, IRB review, and regulatory inspections by providing direct access to all study records. In the event of an audit, the investigator agrees to allow the Sponsor, their representatives, the FDA, or other regulatory agency access to all study records.

The investigator should promptly notify the Sponsor of any audits scheduled by any regulatory authorities and promptly forward copies of any audit reports received to the Sponsor.

6.2 Management of Protocol Amendments and Deviations

6.2.1 Modification of the Protocol

Any changes in this research activity, except those necessary to remove an apparent, immediate hazard to the participant, must be reviewed and approved by the Sponsor or designee. Amendments to the protocol must be submitted in writing to the investigator's IRB for approval before participants are enrolled into an amended protocol.

6.2.2 Protocol Deviations

A protocol deviation is any change, divergence, or departure from the study design or procedures of a study protocol. A protocol deviation may be a reason to remove data from an analysis set at the time of analysis. Major protocol deviations are defined as exclusionary from the analysis according to the protocol objectives and endpoints. Protocol deviations will be documented by the study monitor throughout the course of monitoring visits. The investigator will be notified in writing by the monitor of such deviations.

After a participant proceeds to the open-label Part B of the study at the Participant Decision Clinic Visit (Table 10), Part A schedule of events (Table 9) will no longer be followed and become obsolete.

6.3 Study Termination

Although the Sponsor has every intention of completing the study, they reserve the right to discontinue it at any time for clinical or administrative reasons.

The EOS is defined as the date on which the last participant completes the last visit (includes the EOS Visit and any additional long-term follow-up). Any additional long-term follow-up that is required to monitor the resolution of a finding or AE may be reported through an amendment to the CSR.

6.4 Clinical Study Reports

Whether the study is completed or prematurely terminated, the Sponsor will ensure that CSRs are prepared and provided to the regulatory agency(ies) as required by the applicable regulatory requirement(s). The Sponsor will also ensure that CSRs in marketing applications meet the standards of the ICH harmonized tripartite guideline E3: Structure and Content of Clinical Study Reports.

Where required by applicable regulatory requirements, an investigator signatory will be identified for the approval of the CSRs. The investigator will be provided reasonable access to statistical tables, figures, and relevant reports and will have the opportunity to review complete study results.

The final CSR will be prepared based on the primary analysis of safety and immunogenicity data, which is based on cleaned and locked data collected from participants from Day 0 through the Day 57 visit (Section 4.7.1). Reports after Part B and Part C interim analyses may be generated. All data collected up to OL-D29 and/or OL-D57 in each Part B and Part C, will be cleaned (i.e., data that are as clean as possible) and locked. Results of these analysis may be presented in a CSR.

The EOS CSR will be based on an analysis of all data collected from Day 0 through Day 394 (Blinded Part A), OL-M6 or OL-M7 (Open-Label Part B), and OL-M6 (Open-Label Part C). The Sponsor will provide the investigator(s) with the final approved EOS CSR.

7 APPENDICES

7.1 Appendix 1: Schedule of Events

The Schedules of Events are presented in Table 9 for Part A, and Table 11 and Table 12 for Part B, and Table 12 for Part C. Reasons and procedures for possible unscheduled visits are provided in Section 3.4 and Section 3.4.1.

If a participant cannot attend a study site visit (scheduled or unscheduled) with the exception of Screening, Day 1, and Day 29 visits, a home visit is acceptable if performed by appropriately delegated study site staff or a home healthcare service provided by the Sponsor. If neither a participant visit to the study site nor a home visit to the participant is possible (with the exception of Screening, Day 1, and Day 29 visits), a safety telephone call should be performed that includes the assessments scheduled for the safety telephone calls.

After the Participant Decision Clinic Visit, all participants will proceed with Part B SOEs as described in (Table 10).

Table 9: Schedule of Events (Part A, Blinded Phase only)

**Visit Number**	0	1	2	3	4	5	6	7			8			9
**Type of Visit**	C	C	C	C	C	C	C	C	SFU		C	SFU		C
**Month Timepoint**		M0			M1			M2	eDiary	SC	M7	eDiary	SC	M13
**Study Visit Day**	D0[1] (Screening)	D1 (Baseline)	D8[2]	D15[2]	D29[3]	D36[2, 3]	D43[2, 3]	D57[2, 3]	Every 4 weeks D71 – D183[3, 4]	Every 4 weeks D85– D197[3, 5]	D209[2, 3]	Every 4 weeks D223– D363[3, 4]	Every 4 weeks D237– D377[3, 5]	D394[2, 3]
**Window Allowance (Days)**	-28		+3	±3	+7	+3	±3	-3/ +7	±2	±3	±14	±2	±3	±14
**Days Since Most Recent Injection**	-	0	7	14	28/0	7	14	28	-	-	180	-	-	365
ICF, demographics, concomitant medications, medical history	X													
Confirm participant meets inclusion and exclusion criteria	X	X												
Blood for safety laboratory tests[6]	X				X[4]			X[4]						
Blood for viral serology (hepatitis B, hepatitis C, HIV [1 and 2])	X													
Physical examination including vital signs[7]	X	X	X	X	X	X	X	X			X			X
Pregnancy testing[8]	X	X			X									
Randomization		X												
Study injection (including 30-minute post-dosing observation period)		X			X									
Blood for vaccine immunogenicity[9]		X		X	X		X	X			X			X
Nasopharyngeal swab sample for SARS-CoV-2[10]		X			X			X						
eDiary activation for recording solicited adverse reactions (7 days)[11]		X			X									
Review of eDiary			X			X								

**Visit Number**	0	1	2	3	4	5	6	7			8			9
**Type of Visit**	C	C	C	C	C	C	C	C	SFU		C	SFU		C
**Month Timepoint**		M0			M1			M2	eDiary	SC	M7	eDiary	SC	M13
**Study Visit Day**	D0[1] (Screening)	D1 (Baseline)	D8[2]	D15[2]	D29[3]	D36[2, 3]	D43[2, 3]	D57[2, 3]	Every 4 weeks D71 – D183[3, 4]	Every 4 weeks D85– D197[3, 5]	D209[2, 3]	Every 4 weeks D223– D363[3, 4]	Every 4 weeks D237– D377[3, 5]	D394[2, 3]
**Window Allowance (Days)**	-28		+3	±3	+7	+3	±3	-3/ +7	±2	±3	±14	±2	±3	±14
**Days Since Most Recent Injection**	-	0	7	14	28/0	7	14	28	-	-	180	-	-	365
Follow-up safety calls[12]										X			X	
Recording of unsolicited AEs		X	X	X	X	X	X	X						
Recording of MAAEs and concomitant medications relevant to or for the treatment of the MAAE		X	X	X	X	X	X	X	X[12]	X	X	X[12]	X	X
Recording of SAEs and concomitant medications relevant to or for the treatment of the SAE		X	X	X	X	X	X	X	X[12]	X	X	X[12]	X	X
Recording of concomitant medications and non-study vaccinations[13]		X	X	X	X	X	X	X						
Study completion														X

Abbreviations: AE = adverse event; ALT = alanine aminotransferase; AST = aspartate aminotransferase; BUN = blood urea nitrogen; C = clinic visit; CBC = complete blood count; D = day; HIV = human immunodeficiency virus; ICF = informed consent form; M = month; MAAE = medically attended AE; SC = safety (telephone) call; SFU = Safety Follow Up; PCR = polymerase chain reaction; PT = prothrombin time; PTT = partial thromboplastin time; SAE = serious adverse event.

Note: In accordance with "FDA Guidance on Conduct of Clinical Trials of Medical Products during COVID-19 Public Health Emergency" (DHHS 2020), investigators may convert study site visits to telemedicine visits with the approval of the Sponsor.

1. The Day 0 visit may be performed over multiple visits if within the 28-day screening window.
2. All scheduled study visits should be completed within the respective visit windows. If the participant is not able to come on site for a study site visit as a result of the COVID-19 pandemic (self-quarantine or disruption of study site activities following business continuity plans and/or local government mandates for "stay at home" or "shelter in place"), a safety call to the participant should be made in place of the study site visit. The safety call should encompass all scheduled visit assessments that can be completed remotely, such as assessment for adverse events and concomitant medications (eg, as defined in scheduled safety telephone calls). Home visits will be permitted for all non-dosing visits except for Screening if a participant cannot come to the study site as a result of

the COVID-19 pandemic. Home visits must be permitted by the site IRB and the participant via informed consent and have prior approval from the Sponsor (or its designee).

3. If the visit for the second dose (Day 29) is disrupted and cannot be completed at Day 29 +7 days as a result of the COVID-19 pandemic (self-quarantine or disruption of clinical site activities following business continuity plans and/or local government mandates for "stay at home" or "shelter in place"), the window may be extended to Day 29 + 21 days. When the extended window is used, the remaining study visits should be rescheduled to follow the inter-visit interval from the actual date of the second dose.
4. Safety follow-up via eDiary questionnaire will be performed every 4 weeks starting at Day 71 to Day 183 and again starting at Day 223 to Day 363. These study days are relative to Day 1 vaccine administration and are not affected by the timing of the second vaccine administration.
5. Safety follow-up via a safety telephone call will be performed every 4 weeks starting at Day 85 to Day 197 and again starting at Day 237 to Day 377. There is potential overlap of the Day 197 safety telephone call and Visit 8 due to their respective visit windows. As such, the safety telephone call on Day 197 only needs to be performed if the participant is scheduled to complete Visit 8 after Day 200.
6. Safety laboratory tests include the following: CBC with differential, AST, ALT, total and direct bilirubin, ALP, BUN/creatinine, PT/PTT. Safety laboratory tests are to be repeated at Day 29 and Day 57 **only for Cohort 2 (≥ 55 years old).**
7. Physical examination: A full physical examination, including height and weight, will be performed at Day 1, Day 29, and Day 57. Symptom-directed physical examinations may be performed at other time points at the discretion of the investigator. On each injection day before injection and again 7 days after injection, the arm receiving the injection should be examined and the associated lymph nodes should be evaluated. Any clinically significant finding identified during a study visit should be reported as an MAAE. Vital signs are to be collected pre- and post-dosing on days of injection (Day 1 and Day 29). When applicable, vital sign measurements should be performed before blood collection. Participants who are febrile (body temperature ≥ 38.0°C/100.4°F) before injection on Day 1 or Day 29 must be rescheduled within the relevant window period to receive the injection. Afebrile participants with minor illnesses can be administered investigational product at the discretion of the investigator.
8. Pregnancy test at Screening and Day 1 and before the second study injection will be a point-of-care urine test. At the discretion of the investigator a pregnancy test either via blood or point-of-care urine test can be performed. Follicle-stimulating hormone level may be measured to confirm menopausal status at the discretion of the investigator.
9. Sample must be collected prior to dosing on days of injection (Day 1 and Day 29).
10. The nasopharyngeal swab sample, collected prior to vaccination on days of injection (Day 1 and Day 29), will be used to ascertain the presence of SARS-CoV-2 via PCR.
11. Diary entries will be recorded by the participant starting approximately 1 hour after injection while at the study site with instruction provided by study staff. Study participants will continue to record in the eDiary each day after they leave the study site, preferably in the evening and at the same time each day, on the day of injection and for 6 days following injection. Any solicited AR that is ongoing beyond Day 7 will be reported in the eDiary until resolution. Adverse reactions recorded in eDiaries beyond Day 7 should be reviewed either during the next telephone call or at the next study site visit.
12. Trained site personnel will call all participants to collect information relating to any AEs, MAAEs, SAEs, AEs leading to study withdrawal, information on concomitant medications associated with those events, and any non-study vaccinations. In addition, study personnel will collect information on known participant exposure to someone with known COVID-19 or SARS-CoV-2 infection and on participant experience of COVID-19 symptoms. Sites will collect this information for diary days only if diary responses indicate the need for follow-up via telephone.
13. All concomitant medications and non-study vaccinations will be recorded through 28 days after each injection; all concomitant medications relevant to or for the treatment of an SAE or MAAE will be recorded from Day 1 through the final visit (Day 394).

Table 10: Part B: Participant Decision Clinic Visit

	**All Participants**
Return to clinic for Participant Decision Clinic Visit	X
Sign revised Informed Consent Form	X
Confirm participant's choice to be unblinded or not to be unblinded	X
Confirm participant's choice to receive open-label mRNA-1273	X
Nasopharyngeal swab	X
Blood for immunologic analysis	X
Counsel about social distancing practices[1]	X
**Participant Status at end of Participant Decision Clinic Visit**	**Part B Schedule of Events**
**Consents to unblinding and to receiving either 2 doses of mRNA-1273, 28 days apart, or a single booster of mRNA-1273 (50 µg) at OL-D1 in Part B**	
Received placebo in Part A	Table 11
Received 1 dose of mRNA-1273 in Part A	Table 12
Received 2 doses of mRNA-1273 in Part A	Table 12
**Consents to unblinding but declines to receiving either 2 doses of mRNA-1273, 28 days apart, or a single booster of mRNA-1273 (50 µg) in Part B**	
Received 1 or 2 doses of placebo in Part A	Table 12
Received 1 dose of mRNA-1273 in Part A[2]	Table 12
Received 2 doses of mRNA-1273 in Part A	Table 12
**Declines unblinding and mRNA-1273 or booster in Part B**	Table 12

1. All participants are counselled about the importance of continuing other public health measures to limit the spread of disease including physical-social distancing, wearing a mask, and hand-washing.
2. Applies to participants in Part A who received 50 µg or 100 µg mRNA-1273.

Table 11: Part B: Open-label Schedule of Events: ONLY for Participants Receiving 2 Doses of mRNA-1273 28 Days Apart

**Visit Number**	0	OL-1	OL-2	OL-3	OL-4		OL-5			OL-6
**Type of Visit**	C	C	C	C	C	SC	C	SFU		C
**Month Timepoint**		OL-M0			OL-M1		OL-M2	eDiary	SC	OL-M7
**Study Visit Day**	OL-D1[1] Participant Decision Visit		OL-D8	OL-D15	OL-D29	OL-D36	OL-D57	Every 4 weeks OL-D71–OL-D155[2]	Every 4 weeks OL-D85–OL-D169	OL-D209
**Window Allowance (Days)**	7		+3	±3	+7	+3	-3/+7	±2	±3	±14
**Days Since Most Recent Injection**	0		7	14	28	7	28	--	--	180
Informed consent form	X									
Confirm participant meets inclusion and exclusion criteria	X									
Physical examination including vital signs[3]	X				X		X			X
Pregnancy testing	X				X					
Study injection (including 30-minute post-dosing observation period)	X				X					
Blood for serology[9] and immunogenicity[4]	X		X	X	X		X			X
Nasopharyngeal swab sample for SARS-CoV-2[5]	X				X		X			
eDiary activation for recording solicited adverse reactions (7 days)[6]	X				X					
Review of eDiary			X		X	X				
Follow-up safety calls[7]						X			X	
Recording of unsolicited AEs	X		X	X	X	X	X			
Recording of MAAEs and concomitant medications relevant to or for the treatment of the MAAE[8]	X		X	X	X	X	X	X	X	X

**Visit Number**	0	OL-1	OL-2	OL-3	OL-4		OL-5			OL-6
**Type of Visit**	C	C	C	C	C	SC	C	SFU		C
**Month Timepoint**		OL-M0			OL-M1		OL-M2	eDiary	SC	OL-M7
**Study Visit Day**	OL-D1[1] Participant Decision Visit		OL-D8	OL-D15	OL-D29	OL-D36	OL-D57	Every 4 weeks OL-D71–OL-D155[2]	Every 4 weeks OL-D85–OL-D169	OL-D209
**Window Allowance (Days)**	7		+3	±3	+7	+3	-3/+7	±2	±3	±14
**Days Since Most Recent Injection**	0		7	14	28	7	28	--	--	180
Recording of SAEs and concomitant medications[8] relevant to or for the treatment of the SAE	X		X	X	X	X	X	X[8]	X	X
Recording of concomitant medications and non-study vaccinations[8]	X		X	X	X	X	X			
Study Completion										X

Abbreviations: AE = adverse event; C = clinic visit; M = month; MAAE = medically attended AE; SC = safety (telephone) call; SFU = Safety Follow Up; SAE = serious adverse event.

Note: In accordance with “FDA Guidance on Conduct of Clinical Trials of Medical Products during COVID-19 Public Health Emergency” (DHHS 2020), investigators may convert study site visits to telemedicine visits with the approval of the Sponsor

1. The Participant Decision Visit may be performed over multiple visits if within the given window.
2. Safety follow-up via eDiary questionnaire will be performed every 4 weeks starting at OL-Day 71 to OL-Day 155. These study days are relative to OL-Day 1 vaccine administration and are not affected by the timing of the second vaccine administration.
3. Physical examination: A symptom-directed physical examination, including height and weight, will be performed at OL-Day 1, OL-Day 29, and OL-Day 57. Symptom-directed physical examinations may be performed at other time points at the discretion of the investigator. Any clinically significant finding identified during a study visit should be reported as an MAAE. Vital signs are to be collected pre- and post-dosing on days of injection (Day 1 and Day 29). When applicable, vital sign measurements should be performed before blood collection. Participants who are febrile (body temperature ≥ 38.0°C/100.4°F) before injection on OL-Day 1 or OL-Day 29 must be rescheduled within the relevant window period to receive the injection. Afebrile participants with minor illnesses can be administered investigational product at the discretion of the investigator.
4. Sample must be collected prior to dosing on days of injection.
5. The nasopharyngeal swab sample, collected prior to vaccination on days of injection, will be used to ascertain the presence of SARS-CoV-2 via PCR.
6. Diary entries will be recorded by the participant starting approximately 30 minutes after injection while at the study site with instruction provided by study staff. Study participants will continue to record in the eDiary each day after they leave the study site, preferably in the evening and at the same time each day, on the day of injection and for 6 days following injection. Any solicited AR that is ongoing beyond Day 7 will be reported in the eDiary until resolution. Adverse reactions recorded in eDiaries beyond Day 7 should be reviewed either during the next telephone call or at the next study site visit.

7. Trained site personnel will call all participants to collect information relating to any AEs, MAAEs, SAEs, AEs leading to study withdrawal, information on concomitant medications associated with those events, and any non-study vaccinations. In addition, study personnel will collect information on known participant exposure to someone with known COVID-19 or SARS-CoV-2 infection and on participant experience of COVID-19 symptoms. Sites will collect this information for diary days only if diary responses indicate the need for follow-up via telephone.
8. All concomitant medications and non-study vaccinations will be recorded through 28 days injection; all concomitant medications relevant to or for the treatment of an SAE or MAAE will be recorded from Day 1 through the final visit (OL-Day 209).
9. Blood sample for serology will be collected only at the Participant Decision Visit.

Table 12: Part B and Part C: Open-label Schedule of Events (Participants Receiving a Booster Dose)[9]

**Visit Number**	0	OL-1	OL-2	OL-3	OL-4	OL-5				OL-6
**Type of Visit**	C	C	C	C	C	C	SC	SFU		C
**Month Timepoint**		OL-M0			OL-M1	OL-M2		eDiary	SC	OL-M6
**Study Visit Day**	OL-D1/ Participant Decision Visit[1,2]		OL-D8	OL-D15	OL-D29	OL-D57	OL-D64[12]	Every 4 weeks OL-D71–OL-D155[3]	Every 4 weeks OL-D85–OL-D169	OL-D181
**Window Allowance (Days)**			+3	±3	+7	-3/+7	+3	±2	±3	±14
**Days Since Most Recent Injection**	0		7	14	28	56	7	--	--	180
Informed consent form	X									
Confirm participant meets inclusion and exclusion criteria	X									
Physical examination including vital signs[4]	X				X	X				X
Pregnancy testing	X									
Study injection (including 30-minute post-dosing observation period)	X					X[11]				
Blood for serology[10] and immunogenicity[5]	X		X	X	X	X				X
Nasopharyngeal swab sample for SARS-CoV-2[6]	X				X	X				
eDiary activation for recording solicited adverse reactions (7 days)[7]	X					X[12]				
Review of eDiary			X				X[12]			
Follow-up safety calls[8]									X	
Recording of unsolicited AEs	X		X	X	X	X[12]	X[12]			

Visit Number	0	OL-1	OL-2	OL-3	OL-4	OL-5				OL-6
**Type of Visit**	C	C	C	C	C	C	SC	SFU		C
**Month Timepoint**		OL-M0			OL-M1	OL-M2		eDiary	SC	OL-M6
**Study Visit Day**		OL-D1/ Participant Decision Visit[1,2]	OL-D8	OL-D15	OL-D29	OL-D57	OL-D64[12]	Every 4 weeks OL-D71–OL-D155[3]	Every 4 weeks OL-D85–OL-D169	OL-D181
**Window Allowance (Days)**			+3	±3	+7	-3/+7	+3	±2	±3	±14
**Days Since Most Recent Injection**		0	7	14	28	56	7	--	--	180
Recording of MAAEs and concomitant medications relevant to or for the treatment of the MAAE[9]		X	X	X	X	X	X	X	X	X
Recording of SAEs and concomitant medications relevant to or for the treatment of the SAE[9]		X	X	X	X	X	X	X	X	X
Recording of concomitant medications and non-study vaccinations[9]		X	X	X	X	X[12]	X[12]			
Study Completion										X

Abbreviations: AE = adverse event; C = clinic visit; ICF = informed consent form; M = month; MAAE = medically attended AE; SC = safety (telephone) call; SFU = Safety Follow Up; SAE = serious adverse event.

Note: In accordance with "FDA Guidance on Conduct of Clinical Trials of Medical Products during COVID-19 Public Health Emergency" (DHHS 2020), investigators may convert study site visits to telemedicine visits with the approval of the Sponsor.

1. The Participant Decision Visit is only applicable to Part B and may be performed over multiple visits if within the given window.
2. Participants who received 1 dose of mRNA-1273 (50 or 100ug) in Part A and agree to receive 1 dose of mRNA-1273 in Part B, will follow Table 11 Schedule of Events.
3. Safety follow-up via eDiary questionnaire will be performed every 4 weeks starting at OL-Day 71 to OL-Day 155. These study days are relative to OL-Day 1 vaccine administration.
4. Physical examination: A symptom-directed physical examination, including height and weight, will be performed at OL-Day 1, OL-Day 29, and OL-Day 57. Symptom-directed physical examinations may be performed at other time points at the discretion of the investigator. On the day of injection before injection and again 7 days after injection, the arm receiving the injection should be examined and the associated lymph nodes should be evaluated. Any clinically significant finding identified during a study visit should be reported as an MAAE. Vital signs are to be collected pre- and post-dosing on days of injection (OL-Day 1). When applicable, vital sign measurements should be performed before blood collection. Participants who are febrile (body

temperature ≥ 38.0°C/100.4°F) before injection on OL-Day 1 must be rescheduled within the relevant window period to receive the injection. Afebrile participants with minor illnesses can be administered investigational product at the discretion of the investigator.

5. Sample must be collected prior to dosing on days of injection.
6. The nasopharyngeal swab sample, collected prior to vaccination on days of injection, will be used to ascertain the presence of SARS-CoV-2 via PCR.
7. Diary entries will be recorded by the participant starting approximately 30 minutes after injection while at the study site with instruction provided by study staff. Study participants will continue to record in the eDiary each day after they leave the study site, preferably in the evening and at the same time each day, on the day of injection and for 6 days following injection. Any solicited AR that is ongoing beyond Day 7 will be reported in the eDiary until resolution. Adverse reactions recorded in eDiaries beyond Day 7 should be reviewed either during the next telephone call or at the next study site visit.
8. Trained site personnel will call all participants to collect information relating to any AEs, MAAEs, SAEs, AEs leading to study withdrawal, information on concomitant medications associated with those events, and any non-study vaccinations. In addition, study personnel will collect information on known participant exposure to someone with known COVID-19 or SARS-CoV-2 infection and on participant experience of COVID-19 symptoms. Sites will collect this information for diary days only if diary responses indicate the need for follow-up via telephone.
9. All concomitant medications and non-study vaccinations will be recorded through 28 days injection; all concomitant medications relevant to or for the treatment of an SAE or MAAE will be recorded from OL-Day 1 through the final visit (OL-Day 181).

NOTE: The following participants will complete the described assessments in this SOE at OL-Day 1, and will skip OL-D8, OL-D15, OL-Day 29, and OL-D57:

- Participants who received 1 or 2 doses of mRNA-1273 in Part A and decline receiving booster mRNA-1273 in Part B.
- Participants who decline unblinding
- Participants who received placebo in Part A, but decline to receive 2 doses of mRNA-1273 in Part B

Participants who decline receiving booster mRNA-1273, decline unblinding, or decline to receive 2 doses of mRNA-1273 in Part B will not receive study vaccination at OL-D1 (or OL-D29), will not perform a pregnancy test, or eDiary activation at OL-D1.

10. Blood sample for serology will be collected only at the Participant Decision Visit (OL-D1).
11. In Part C, an additional booster dose may be added approximately 56 days after the first boost at OL-D1. This additional booster dose will be triggered following review of immunogenicity data up to OL-D15 of the initial mRNA1273.351 (20 µg and 50 µg) and mRNA-1273/mRNA-1273.351 mixture injections.
12. If the additional booster dose is given at OL-D57, an eDiary for reactogenicity will be completed for 7 days post-injection. Additionally, unsolicited AEs will continue to be collected for 28 days post last injection.

7.2 Appendix 2: Toxicity Grading Scale Tables

The toxicity grading scales for clinical and laboratory abnormalities are presented in Table 13 and Table 14, respectively. Note that for laboratory abnormalities, grading only occurs if the values are outside of the normal values established by the clinical laboratory. For study-specific laboratory normal ranges and associated toxicity grades, refer to the laboratory manual and provided toxicity grade communications.

Table 13: Tables for Clinical Abnormalities

Local Reaction to Injectable Product	Mild (Grade 1)	Moderate (Grade 2)	Severe (Grade 3)	Potentially Life-threatening (Grade 4)
Pain	Does not interfere with activity	Repeated use of non-narcotic pain reliever > 24 hours or interferes with activity	Any use of narcotic pain reliever or prevents daily activity	ER visit or hospitalization
Tenderness	Mild discomfort to touch	Discomfort with movement	Significant discomfort at rest	ER visit or hospitalization
Erythema/Redness *	2.5 – 5 cm	5.1 – 10 cm	> 10 cm	Necrosis or exfoliative dermatitis
Induration/Swelling **	2.5 – 5 cm and does not interfere with activity	5.1 – 10 cm or interferes with activity	> 10 cm or prevents daily activity	Necrosis

Abbreviation: ER = emergency room.

* In addition to grading the measured local reaction at the greatest single diameter, the measurement should be recorded as a continuous variable.

** Induration/swelling should be evaluated and graded using the functional scale as well as the actual measurement.

Source: Guidance for industry – Toxicity Grading Scale for Heathy Adult and Adolescent Volunteers Enrolled in Preventive Vaccine Clinical Trials; tables for clinical abnormalities (DHHS 2007).

Vital Signs *	Mild (Grade 1)	Moderate (Grade 2)	Severe (Grade 3)	Potentially Life-threatening (Grade 4)
Tachycardia (beats per minute)	101 – 115	116 – 130	> 130	ER visit or hospitalization for arrhythmia
Bradycardia (beats per minute)**	50 – 54	45 – 49	< 45	ER visit or hospitalization for arrhythmia
Hypertension (systolic) (mm Hg)	141 – 150	151 – 155	> 155	ER visit or hospitalization for malignant hypertension
Hypertension (diastolic) (mm Hg)	91 – 95	96 – 100	> 100	ER visit or hospitalization for malignant hypertension
Hypotension (systolic) (mm Hg)	85 – 89	80 – 84	< 80	ER visit or hospitalization for hypotensive shock
Respiratory rate (breaths per minute)	17 – 20	21 – 25	> 25	Intubation

Abbreviation: ER = emergency room.

Note that fever is classified under systemic reactions for grading purposes.

* Participant should be at rest for all vital sign measurements.

** When resting heart rate is between 60 – 100 beats per minute. Use clinical judgment when characterizing bradycardia among some healthy participant populations, for example, conditioned athletes.

Source: Guidance for industry – Toxicity Grading Scale for Heathy Adult and Adolescent Volunteers Enrolled in Preventive Vaccine Clinical Trials; tables for clinical abnormalities (DHHS 2007).

Systemic (General)	Mild (Grade 1)	Moderate (Grade 2)	Severe (Grade 3)	Potentially Life-threatening (Grade 4)
Fever (°C) *<br>(°F) *	38.0 – 38.4<br>100.4 – 101.1	38.5 – 38.9<br>101.2 – 102.0	39.0 – 40.0<br>102.1 – 104.0	> 40.0<br>> 104.0
Nausea/vomiting	No interference with activity or 1 to 2 episodes/24 hours	Some interference with activity or > 2 episodes/ 24 hours	Prevents daily activity, requires outpatient IV hydration	ER visit or hospitalization for hypotensive shock
Diarrhea	2 – 3 loose stools or < 400 g/24 hours	4 – 5 stools or 400 – 800 g/ 24 hours	6 or more watery stools or > 800 g/ 24 hours or requires outpatient IV hydration	ER visit or hospitalization
Headache	No interference with activity	Repeated use of non-narcotic pain reliever > 24 hours or some interference with activity	Significant; any use of narcotic pain reliever or prevents daily activity	ER visit or hospitalization
Fatigue/malaise (unusual tiredness)	No interference with activity	Some interference with activity	Significant; prevents daily activity	ER visit or hospitalization
Generalized myalgia (muscle ache or pain)	No interference with activity	Some interference with activity	Significant; prevents daily activity	ER visit or hospitalization
Generalized arthralgia (joint ache or pain)	No interference with activity	Some interference with activity not requiring medical intervention	Prevents daily activity and requires medical intervention	ER visit or hospitalization

Abbreviations: ER = emergency room; IV = intravenous.

* Oral temperature; no recent hot or cold beverages or smoking.

Sources: Guidance for industry – Toxicity Grading Scale for Heathy Adult and Adolescent Volunteers Enrolled in Preventive Vaccine Clinical Trials; tables for clinical abnormalities (DHHS 2007). Division of AIDS Grading the Severity of Adult and Pediatric Adverse Events (DHHS 2014).

Table 14: Tables for Laboratory Abnormalities

Serum Chemistry*	Mild (Grade 1)	Moderate (Grade 2)	Severe (Grade 3)	Potentially Life-threatening (Grade 4)**
BUN (mg/dL)	23 – 26	27 – 31	> 31	Requires dialysis
Creatinine (mg/dL)	1.5 – 1.7	1.8 – 2.0	2.1 – 2.5	> 2.5 or requires dialysis
ALP; increase by factor	1.1 – 2.0 × ULN	2.1 – 3.0 × ULN	3.1 – 10 × ULN	> 10 × ULN
Liver function tests – ALT and AST; increase by factor	1.1 – 2.5 x ULN	2.6 – 5.0 × ULN	5.1 – 10 × ULN	> 10 × ULN
Bilirubin – when accompanied by any increase in liver function test; increase by factor	1.1 – 1.25 × ULN	1.26 – 1.5 × ULN	1.51 – 1.75 × ULN	> 1.75 × ULN
Bilirubin – when liver function test is normal; increase by factor	1.1 – 1.5 × ULN	1.6 – 2.0 × ULN	2.0 – 3.0 × ULN	> 3.0 × ULN

Abbreviations: ALP = alkaline phosphatase; ALT = alanine aminotransferase; AST = aspartate aminotransferase; BUN = blood urea nitrogen; ULN = upper limit of the normal range.

* The laboratory values provided in the tables serve as guidelines and are dependent upon institutional normal parameters. Institutional normal reference ranges should be provided to demonstrate that they are appropriate.

** The clinical signs or symptoms associated with laboratory abnormalities might result in characterization of the laboratory abnormalities as potentially life-threatening (Grade 4). For example, a low sodium value that falls within a Grade 3 parameter (125 – 129 mEq/L) should be recorded as a Grade 4 hyponatremia event if the participant had a new seizure associated with the low sodium value.

Source: Guidance for industry – Toxicity Grading Scale for Heathy Adult and Adolescent Volunteers Enrolled in Preventive Vaccine Clinical Trials; tables for laboratory abnormalities (DHHS 2007).

**Hematology ***	**Mild (Grade 1)**	**Moderate (Grade 2)**	**Severe (Grade 3)**	**Potentially Life-Threatening (Grade 4)**
Hemoglobin (female) (g/dL)	11.0 – 12.0	9.5 – 10.9	8.0 – 9.4	< 8.0
Hemoglobin (female) change from baseline value (g/dL)	Any decrease – 1.5	1.6 – 2.0	2.1 – 5.0	> 5.0
Hemoglobin (male) (g/dL)	12.5 – 13.5	10.5 – 12.4	8.5 – 10.4	< 8.5
Hemoglobin (male) change from baseline value (g/dL)	Any decrease – 1.5	1.6 – 2.0	2.1 – 5.0	> 5.0
WBC increase (cell/mm^3)	10,800 – 15,000	15,001 – 20,000	20,001 – 25,000	> 25,000
WBC decrease (cell/mm^3)	2,500 – 3,500	1,500 – 2,499	1,000 – 1,499	< 1,000
Lymphocytes decrease (cell/mm^3)	750 – 1,000	500 – 749	250 – 499	< 250
Neutrophils decrease (cell/mm^3)	1,500 – 2,000	1,000 – 1,499	500 – 999	< 500
Eosinophils (cell/mm^3)	650 – 1,500	1,501 – 5,000	> 5,000	Hypereosinophilic
Platelets decreased (cell/mm^3)	125,000 – 140,000	100,000 – 124,000	25,000 – 99,000	< 25,000
PT; increase by factor	> 1.0 – 1.10 × ULN	1.11 – 1.20 × ULN	1.21 – 1.25 × ULN	> 1.25 × ULN
PTT; increase by factor	> 1.0 – 1.2 × ULN	1.21 – 1.4 × ULN	1.41 – 1.5 × ULN	> 1.5 × ULN

Abbreviations: PT = prothrombin time; PTT = partial thromboplastin time; ULN = upper limit of normal; WBC = white blood cell.

* The laboratory values provided in the tables serve as guidelines and are dependent upon institutional normal parameters. Institutional normal reference ranges should be provided to demonstrate that they are appropriate. Laboratory abnormality grading occurs only when the values fall beyond the normal ranges established by the local laboratory.

Source: Guidance for industry – Toxicity Grading Scale for Heathy Adult and Adolescent Volunteers Enrolled in Preventive Vaccine Clinical Trials; tables for laboratory abnormalities (DHHS 2007). Note that the criteria for Grade 1 PT and PTT have been adjusted from the source table: instead of ≥ 1.0 x ULN, both criteria are > 1.0 x ULN. Grade 1 will not be used for hematology values due to the large overlap with normal values at the central laboratory.

7.3 Appendix 3: Protocol Amendment History

The document history table for this protocol and the Protocol Amendment Summary of Changes table for the current Amendment 5 is located directly before the Table of Contents.

Descriptions of Amendments 1, 2, 3, 4, and 5 are presented in this appendix.

7.3.1 Amendment 5, 19 Feb 2021

Main Rationale for the Amendment:

There is an urgent need for vaccination strategies against SARS-CoV2 that induce broader protection that includes variants such as B.1.351 to decrease morbidity and mortality. ModernaTX, Inc. is developing a mRNA vaccine (mRNA-1273.351) that is similar to the mRNA-1273 vaccine available under the Emergency Use Authorization (EUA), but in which the mRNA encodes for mutations included in the S protein of the B.1.351 variant.

This protocol amendment will add Part C to the protocol, which will be an amendment to investigate the proof of concept of a single dose booster of two dose levels of the mRNA-1273.351 variant and a mixture formulation of mRNA-1273/mRNA-1273.351 administered to approximately 60 participants who received primary vaccination during the mRNA-1273-P301 COVE study. The COVE study participants will be offered enrollment in this new site-specific sub study, Part C of mRNA-1273-P201, based on pre-determined eligibility criteria. If they choose to enroll in this protocol amendment, the participants will be discontinued from the mRNA-1273-P301 COVE study. The participants would have had to be originally randomized to the mRNA-1273 group and have previously received 2 doses of mRNA-1273, 28 days apart, to be enrolled in this amendment. The unblinding visit should also have occurred. In this protocol amendment, enrolled participants will be allocated 1:1:1 to receive a single intramuscular injection of mRNA-1273.351 (20 µg or 50 µg) or mRNA-1273/mRNA-1273.351 mixture (50 µg) as a booster injection.

The summary of changes table provided here describes the major changes made in Amendment 5 relative to Amendment 4, including the sections modified and the corresponding rationales. The synopsis of Amendment 5 has been modified to correspond to changes in the body of the protocol.

Summary of Major Changes in Protocol Amendment 5:

Section # and Name	Description of Change	Brief Rationale
Title Page, Protocol Approval Page, Headers, Protocol Amendment Summary of Changes	Updated the protocol version and date.	To reflect the new version and date of the protocol.
Protocol Synopsis – Objectives and Section 2.7 (Primary Objectives [Part C, Open Label]), Section 2.8 (Secondary Immunogenicity Objective [Part C, Open Label]), and Section 2.9 (Exploratory Objectives [Part C, Open Label]	The description of the Part C primary, secondary, and exploratory objectives was added to the Protocol Synopsis. The description of Part C was included in Section 2 (Study Objectives). Section 2.7, Section 2.8, and Section 2.9 were added to encompass the addition of Part C primary, secondary, and exploratory objectives.	The addition of Part C was the main purpose of this amendment.
Protocol Synopsis – Study Design and Methodology and Section 3.1 (General Study Design)	A description of the general design of Part C was added to the synopsis. The description was also added to Section 3.1 for consistency.	The addition of Part C was the main purpose of this amendment. Language was included for consistency and clarity.
Protocol Synopsis – Study Design and Methodology and Section 3.1.3 (Part C, Open-Label Interventional Phase of mRNA-1273.351 and mRNA-1273/mRNA-1273.351 Mixture Booster Vaccines)	A further description of the methodology for Part C was included in the synopsis and Section 3.1.3 for consistency and clarity. Figure 4 was added to Section 3.1.3 as a visual aid for the structure of Part C.	The addition of Part C was the main purpose of this amendment. Language was included for consistency and clarity.
Protocol Synopsis – Safety Assessments and Section 3.4.7 (Safety Assessments)	Applicable safety assessments for Part C were indicated in the safety sections of the synopsis and body for consistency.	The addition of Part C was the main purpose of this amendment.
Protocol Synopsis – Immunogenicity assessments, Section 3.4.5 (Immunogenicity Assessments)	Part C immunogenicity assessments were included for clarity.	The addition of Part C was the main purpose of this amendment. Language added for clarity.
Protocol Synopsis – Investigational Product, Dosage, and Route of Administration and Section 3.3.3 (Identity of Investigational Product)	The investigational product, dosage, and route of administration information was provided for Part C.	The addition of Part C was the main purpose of this amendment. Language was included for clarity.
Protocol Synopsis – Investigational Product, Dosage, and Route of Administration and Section 3.3.5 (Blinding)	Specified that Part B and Part C of the study will be open label.	The addition of Part C was the main purpose of this amendment. Language was added for clarity and consistency.

Section # and Name	Description of Change	Brief Rationale
Protocol Synopsis – Sample Size and Section 4.5 (Sample Size Determination)	Clarified that patients in Part A will have the opportunity to enter Part B provided they meet the eligibility criteria.<br>A description of the Part C sample size and enrollment process was provided.	Language was updated for clarity. The description for Part C was included as the addition of Part C was the main purpose of this amendment.
Protocol Synopsis – Statistical Methods and Section 4.6 Statistical Methods.	Indicated that Part C data may be presented separately, as appropriate.<br>Specified when analyses were specific to Part A and Part B.<br>Indicated that Part C will be similarly analyzed (when not specific to Part A and Part B).	Language was included as the addition of Part C was the main purpose of this amendment. Other language updated for clarity and consistency.
Protocol Synopsis – Study Analyses and Section 4.7.3 (Interim Study Analysis for Open-Label Part C Only)	Indicated that an interim analysis of the safety and immunogenicity data in Part C of the study may be performed after participants have completed OL-D8, OL-D15, OL-Day 29 and/or the OL-Day 57 study procedures.<br>Section 4.7.3 added, and language included for consistency and clarity.	The addition of Part C was the main purpose of this amendment. Language was included for clarity.
Section 3.1.6 (Inclusion Criteria)	Section 3.1.6 heading was created.<br>Inclusion criterion #7 was updated to #6 as there is no inclusion criterion #7.<br>A separate set of inclusion criteria for Part C was included. Headers separating Parts A and B from Part C were created.	Headings were created for clarity and readability.<br>Numbering was updated for clarity and accuracy.<br>Part C inclusion criteria was added to clarify what participants will be eligible for Part C.
Section 3.1.7 (Exclusion Criteria)	A separate set of exclusion criteria for Part C was included. Headers separating Parts A and B from Part C were created.	Part C exclusion criteria was added to clarify which participants will not be eligible for Part C. Headers were created for clarity and readability.
Section 3.3.1 (Method of Assigning Participants to Dosing Groups [Part A and Part C])	Table 2 – Dose Group Assignment (Part C) added to clarify groups, investigational product, and number of participants.	The addition of Part C was the main purpose of this amendment. The table was added for clarity.

Section # and Name	Description of Change	Brief Rationale
Section 3.3.2 (Investigational Product Administration)	Indicated that the Part C investigational product will be administered as a single intramuscular injection into the deltoid muscle on the day of the participants consent. Each injection will have variable dose volumes; the 20-µg dose of mRNA-1273.351 is 0.2 mL, the 50-µg dose of mRNA-1273.351 is 0.5 mL, and the 50-µg dose of pharmacy prepared mixture of mRNA-1273/mRNA-1273.351 is 0.5 mL.	The addition of Part C was the main purpose for this amendment. This language was included for clarity.
Section 3.3.2.1 (Pause Rules)	Indicated that the pause rules described in this section are not applicable to Part C. Clarified for Part C, that the sponsor will continue to inform the SMC of any occurrence of the events in Table 3.	The addition of Part C was the main purpose for this amendment. This language was included for clarity.
Section 3.3.2.2 (Contraindications to Subsequent Injection)	Updated how long the patient will be encouraged to continue study participation from 12 months to 6 months.	Existing safety data across mRNA-1273 studies suggest that a safety follow up of 6 months after last vaccination is acceptable.
Section 3.3.4.2 (Packaging and Labeling)	Additional packaging and labeling information for Part C was included.	The addition of Part C was the main purpose of this amendment.
Section 3.3.4.3 (Storage)	Confirmed that the mRNA-1273 vaccine in Part C must be stored at -25°C to -15°C. The mRNA-1273.351 vaccine used in Part C must be stored at -90°C to -60°C (-130°F to -76°F).	The addition of Part C was the main purpose of this amendment and other additional language was for clarity.
Section 3.8.5.6 (Definition and Reporting of Adverse Events Consistent With Anaphylaxis)	Section 3.8.5.6 describes the definitions and reporting procedures for Adverse Events (AEs) consistent with anaphylaxis.	This text is being added to all mRNA-1273 protocols based on recent reports of anaphylaxis in the post-Emergency Use Authorization setting.
Section 4.3.3 (Part C, Open Label)	Updated Section 4.3 to include Part C primary safety endpoints, primary immunogenicity endpoints, secondary immunogenicity endpoint, and exploratory endpoints.	The addition of Part C was the main purpose of this amendment.

Section # and Name	Description of Change	Brief Rationale
Section 7.1 (Appendix 1: Schedule of Events) – Table 11 (Part B: Open-label Schedule of Events: ONLY for Participants Receiving 2 Doses of mRNA-1273 28 Days Apart)	"Blood for vaccine immunogenicity[4]" updated to "Blood for serology[9] and immunogenicity[4]"<br>Added Footnote 9 "Blood sample for serology will be collected only at the Participant Decision Visit."	Language updated and footnote added for clarity and specificity of serology timing.

Section # and Name	Description of Change	Brief Rationale
Section 7.1 (Appendix 1: Schedule of Events) – Table 12 (Part B and Part C: Open-label Schedule of Events (Participants Receiving a Booster Dose)[9]	Table title updated to remove “ONLY for” and “single.”<br>“Blood for vaccine immunogenicity[5]” updated to “Blood for serology[10] and immunogenecity[5]”<br>Updated note language to indicate the following, “Participants who decline receiving booster mRNA-1273, decline unblinding, or decline to receive 2 doses of mRNA-1273 in Part B will not receive study vaccination at OL-D1 (or OL-D29), will not perform a pregnancy test, or eDiary activation at OL-D1.”<br>Added Footnote 10 “Blood sample for serology will be collected only at the Participant Decision Visit (OL-D1).”<br>Addition of Booster injection for Part C under OL-D57.<br>Added footnote 11 “In Part C, an additional booster injection may be added approximately 56 days after the first boost at OL-D1. This additional booster dose will be triggered following review of immunogenicity data up to OL-D15 of the initial mRNA1273.351(20 and 50 µg) and mRNA-1273/mRNA-1273.351 mixture injections.”<br>Added footnote 12 “If the additional booster dose is given at OL-D57, an eDiary for reactogenicity will be completed for 7 days post-injection. Additionally, unsolicited AEs will continue to be collected for 28 days post last injection.”	Title update made as this schedule of events applies to participants who choose to decline unblinding, but wish to remain in the study, decide to unblind but decline to receive further vaccination (either a single mRNA-1273 booster or 2 doses of mRNA-1273, 28 days apart). Language updates made for clarity and specificity of serology timing.<br>An additional booster injection may be added approximately 56 days after the first boost at OL-D1. This will be determined following review of immunogenicity data up to OL-D15.<br>Additional footnotes included for clarity.

7.3.2 Amendment 4, 15 Jan 2021

Main Rationale for the Amendment:

Following authorization of a COVID-19 vaccine under an Emergency Use Authorization (EUA), this study amendment is designed to transition to Part B, the Open-Label Interventional Phase (Figure 3). Transitioning the study to Part B, Open-Label Interventional Phase permits all ongoing study participants to (a) be informed of the availability and eligibility criteria of any COVID-19 vaccine made available under an EUA, and (b) the option to offer all ongoing study participants who request unblinding an opportunity to schedule a study visit to know their original group assignment (placebo vs. mRNA-1273 [50µg or 100µg vaccine]).

Part B, Open-label Interventional Phase, also provides the opportunity for study participants who previously received placebo, to request to receive 2 doses of the mRNA-1273 (100 µg) vaccine. Participants who originally received 1 or 2 doses of mRNA-1273 (50µg or 100µg vaccine) during Part A, will have the opportunity to request to receive a single booster dose of mRNA-1273 (50 µg).

The summary of changes table provided here describes the major changes made in Amendment 4 relative to Amendment 3, including the sections modified and the corresponding rationales. The synopsis of Amendment 4 has been modified to correspond to changes in the body of the protocol.

Summary of Major Changes in Protocol Amendment 4:

Section # and Name	Description of Change	Brief Rationale
Title Page, Protocol Approval Page, Headers, Protocol Amendment Summary of Changes	Updated the protocol version and date	To reflect the new version and date of the protocol
Synopsis (Objectives) and Section 2 (Study Objectives)	Added objectives for Part B, Open Label	Added to specifically enumerate the objectives for the Open-Label part of the study, allowing for changes to study design and dosing.

Section # and Name	Description of Change	Brief Rationale
Synopsis (Study Design), Section 3.1.2 (Part B, Open-Label Interventional Phase), Section 7.1 (Appendix 1: Schedule of Events)	Added a "Participant Decision Clinic Visit"; instructions for transitioning to Part B, Open Label; Schedule of Events for the Participant Decision Clinic Visit; and Schedules of Events for Part B, Open-Label procedures.	This Participant Decision Clinic Visit provides the opportunity for study site personnel to discuss with and offer to participants, the choice to be unblinded, as well as offering to participants who originally received placebo, the choice to receive active vaccination with mRNA-1273 and possible vaccination against COVID-19, as well as offering those who received mRNA-1273 during Part A, the choice to receive a booster injection of mRNA-1273.<br>The new Schedules of Events distinguish Part B, Open Label from Part A, Blinded, since all participants will transition to Part B. The Part B Schedules of Events provide procedural instructions for participants who will receive 2 mRNA-1273 injections and for those who will receive 1 mRNA-1273 injection in Part B.
Section 1.2 (Nonclinical Studies in Development of mRNA-1273) and Section 1.3 (Clinical Studies With Lipid Nanoparticle mRNA Vaccines)	Updated status of nonclinical studies, as well as ongoing clinical studies, including this study (mRNA-1273-P201) and the Phase 3 Study mRNA-1273-P301.	The status of the 3 clinical studies (one Phase 1, one Phase 2a, and the Phase 3 study) has changed since Amendment 3.<br>In addition, the results of the interim analyses in the Phase 3 study of the primary efficacy endpoint (prevention of COVID-19 infection), a major secondary endpoint (prevention of severe COVID-19), and safety and reactogenicity endpoints are now available and are provided here. These results provide the justification for offering participants the opportunity to receive active investigational product (mRNA-1273) and the potential benefit of vaccination against COVID-19.

Section # and Name	Description of Change	Brief Rationale
Section 4.3.2 (Statistical Analyses, Part B, Open Label)	Addition of Part B, open-label statistical analyses	Lists the Part B, open-label endpoints. Major differences from the Part A endpoints/analyses were elimination of assessment of laboratory values, and distinguishing identical endpoints for 50 µg and 100 µg of mRNA-1273.
Section 3.4.5 (Blinding)	The study site staff, investigators, study monitors, and participants will remain blinded until the conclusion of the study.<br>Changed to: The study site staff, investigators, study monitors, and participants will remain blinded until the <u>initiation of Open-Label Part B.</u>	Previous language is no longer applicable, since the study will be unblinded for Part B.
Section 3.4.2.1 (Pause Rules)	The pause-triggering rules that have been in effect for Part A, will not be applicable for Part B; however, participants will continue to be monitored for the pause rule criteria.	In December 2020, after review of both safety and efficacy data observed to date, the FDA granted Emergency Use Authorization (EUA) to Moderna's mRNA-1273 vaccine. Given this, the current pause rules as outlined cannot be applied effectively, as it is more likely that any serious safety signal would emerge from the ongoing large public vaccination campaigns. Moderna will still monitor for safety events in this study and will report to the Safety Monitoring Committee as appropriate.
Section 4.7.2 (Interim Analysis for Open-Label Part B Only)	Added an option IA following completion of OL-Day 29 and/or OL-Day 57 study procedures.	The IA would help inform of the benefit of participant receiving a booster dose.

7.3.3 Amendment 3, 02 Sep 2020

The main purpose of this amendment was to clarify that data can be analyzed in multiple batches based on availability of participants who have reached the Day 57 visit. The summary of changes table describes the major changes made in Amendment 3 relative to Amendment 2, including the sections modified and the corresponding rationales. Minor editorial or formatting changes are not included in this summary table.

Summary of Major Changes in Protocol Amendment 3:

Section # and Name	Description of Change	Brief Rationale
Title page, Signature page, Synopsis, and header	Updated the protocol version and date	Reflect the new version and date of the protocol.
Synopsis, Section 3.1 Study Design	Deleted repeated text about Safety Monitoring Committee review before expansion in Cohort 2.	Editorial removal of redundant text.
Synopsis,<br>Section 3.4.5 Blinding,<br>Section 4.1 Blinding and Responsibility for Analyses,<br>Section 4.7.1 Primary Study Analysis	Added information about potential participant populations to be included in the primary analysis of safety and immunogenicity after completion of Day 57 procedures.	Clarification that data can be analyzed in multiple batches based on availability of participants who have reached the Day 57 visit.
Synopsis, Section 4.6.2 Safety Analyses	Revisions to clarify that separate summaries of Grade 3 or higher solicited ARs are not planned.	Clarification of planned safety analyses.
Section 3.5.2 Use of Electronic Diaries,<br>Section 7.1 Appendix 1: Schedule of Events (Table 7)	Added clarification about site follow-up of relevant safety events from eDiary entries (includes revisions to Footnote 12).	Clarification that follow-up by telephone of relevant safety events from eDiary entries is not the same as scheduled safety follow-up telephone calls.
Section 7.1 Appendix 1: Schedule of Events (Table 7)	The acceptable window around the Day 29 visit has been clarified as + 7 days with no negative visit window.	Correction to reflect the minimum interval between vaccine administrations of 28 days.
Section 7.1 Appendix 1: Schedule of Events (Table 7)	The footnotes and footnote numbering have been updated to accommodate the footnotes that were added with Amendment 2.	Editorial clarification.
Section 7.1 Appendix 1: Schedule of Events (Table 7)	Footnote 4 has been revised to clarify that study days for safety follow-up are relative to Day 1 vaccine administration.	Editorial clarification.
Section 7.1 Appendix 1: Schedule of Events (Table 7)	Footnote 5 has been revised to explain how to handle potential visit window overlap related to Visit 8.	Editorial clarification.
Section 7.1 Appendix 1: Schedule of Events (Table 7)	Footnote 10 has been revised to clarify the timing of nasopharyngeal swab samples on vaccination days.	Editorial clarification.

Abbreviations: AE = adverse event; AR = adverse reaction; MAAE = medically attended adverse event; SAE = serious adverse event.

7.3.4 Amendment 2, 01 Jul 2020

Main Rationale for the Amendment:

The main purpose of this amendment was to change the statistical analysis plan by removing interim analyses and defining the Primary Study Analysis and EOS Analysis. The summary of changes table describes the major changes made in Amendment 2 relative to Amendment 1, including the sections modified and the corresponding rationales. Minor editorial or formatting changes are not included in this summary table.

Summary of Major Changes in Protocol Amendment 2:

Section # and Name	Description of Change	Brief Rationale
Title page, Signature page, and header	Updated the protocol version and date	Reflect the new version and date of the protocol.
Synopsis, Section 3.1 Study Design, Section 3.5.2 Use of Electronic Diaries, Section 3.5.3 Safety Telephone Calls, Section 7.1 Appendix 1: Schedule of Events (including text, Table 7, and footnotes to Table 7)	Added eDiary questionnaires to the procedure for safety follow-up after the Day 57 visit, with completion of eDiary questionnaires alternating with safety telephone calls approximately every 2 weeks after the Day 57 visit.	Reduce the burden on study site personnel of completing safety follow-up by telephone.
Synopsis, Section 3.1 Study Design, Section 3.5.3 Safety Telephone Calls, Section 7.1 Appendix 1: Schedule of Events (footnote 12)	Added exposure to someone with known COVID-19 or SARS-CoV-2 infection and participant experience of COVID-19 symptoms to the list of events queried during scheduled safety telephone calls.	Improve surveillance for incidence of COVID-19 during the study.
Synopsis, Section 3.1 Study Design	End of Study definition was amended.	Minor clarification to define the End of Study.
Synopsis, Section 3.4.5 Blinding, Section 4.1 Blinding and Responsibility for Analyses, Section 4.7 Study Analyses, Section 4.7.1 Primary Study Analysis, Section 4.7.2 End of Study Analysis, Section 6.4 Clinical Study Reports	Added descriptions of the Primary Study Analysis and End of Study Analysis and respective clinical study reports, replacing descriptions of interim analyses and reports. The synopsis contains a new section.	Eliminate interim analyses in favor of a focus on the primary analysis.
Synopsis, Section 4.6 Statistical Methods	Stated that all analyses will be performed by treatment group overall (for the 2 cohorts combined) and for the 2 cohorts separately, unless specified otherwise.	Previous versions of the protocol had not included the overall analysis in statement of the standard scope of analysis.
Synopsis, Section 4.6.3 Immunogenicity Analyses	For the primary immunogenicity endpoint, geometric mean titer was changed to geometric mean.	Assays for bAb are under development. The reported values may or may not be titers; hence the protocol wording has been modified.

Section # and Name	Description of Change	Brief Rationale
Section 3.5.1 Assessment for SARS-CoV-2 Infection	Added instructions for asymptomatic patients who have a confirmed SARS-CoV-2 infection.	To clarify the steps for the investigator to follow when a participant is confirmed to have SARS-CoV-2 infection but is asymptomatic.
Section 3.5.8.8 Assessment of Severity	Decoupled life-threatening and Grade 4 in the severity assessment.	To adhere to CDISC guidance and align with case report form page.
Section 3.5.8.8 Assessment of Severity	Added clarification on when an AE is defined as serious.	To clarify when an AE is defined as serious.

Abbreviations: AE = adverse event; bAb = binding antibody; CDISC = Clinical Data Interchange Standards Consortium; eDiary = electronic diary; SARS-Cov-2 = Severe Acute Respiratory Syndrome coronavirus that causes COVID-19.

7.3.5 Amendment 1, 18 May 2020

Main Rationale for the Amendment:

The main purpose of this amendment was to incorporate the following modifications requested by the FDA Center for Biologics Evaluation and Research:

- Enhance monitoring of participants who are confirmed to have SARS-CoV-2 infection.
- Include a convalescent visit for participants with confirmed SARS-CoV-2 infection.
- Explore the mRNA-1273 vaccine efficacy in preventing asymptomatic SARS-CoV-2 infection.
- Update the Month 7 and Month 13 visits to Day 209 and Day 394, respectively, to extend the follow-up to a full 12-month period after the second injection on Day 29 (Month 1).
- Decrease the highest dose of mRNA-1273 in the study from 250 µg to 100 µg.

The summary of changes table describes the major changes made in Amendment 1, including the sections modified and the corresponding rationale. Minor editorial or formatting changes are not included in this summary table.

Summary of Major Changes in Protocol Amendment 1:

Section # and Name	Description of Change	Brief Rationale
Title page, Signature page, and header	Updated protocol version and date.	Revised version and date of protocol.
Title page, Signature page, and header	Updated the protocol title.	Revised to reflect the current purpose of the study.
Synopsis and Section 2.3 Exploratory Objectives	Added an exploratory objective to evaluate the effect of the mRNA-1273 vaccine on the incidence of SARS-CoV-2 infection.	Request from the Health Authority.

**Section # and Name**	**Description of Change**	**Brief Rationale**
Synopsis,<br>Section 2.3 Exploratory Objectives, Section 4.3.3 Exploratory Endpoints, Section 4.6.4 Exploratory Analyses	Revised wording for the exploratory objective/endpoint regarding spike protein-specific serum immunoglobulin class and subclass and neutralizing antibody in serum	Editorial change.
Synopsis, Section 3.1 Study Design, Study Flow Schema (Figure 1), Sentinel and Expansion Cohort Schema (Figure 2),<br>Section 3.1.1 Rationale for Dose Selection, 3.4.1 Method of Assigning Participants to Dosing Groups. Dose Group Assignment (Table 1),<br>3.4.2 Investigational Product Administration, 4.5 Sample Size Determination	Decreased the highest dose of mRNA-1273 in the study from 250 µg to 100 µg.	Decreased based on the preliminary findings of the Phase 1 DMID study.
Synopsis and<br>Section 3.1 Study Design	Deleted collection of nasopharyngeal swab samples at the Screening Visit (Day 0).	Editorial update for consistency with Schedule of Events (Table 7).
Synopsis and<br>Section 3.1 Study Design	Deleted the number of visits at which participants will have blood samples collected.	Editorial update to avoid confusion as blood samples will be collected at different visits for safety and vaccine immunogenicity assessments.
Synopsis; Section 3.1 Study Design, Section 3.5.6 Blood Sampling Volumes (Table 3), Section 3.5.7 Safety Assessments,<br>Section 3.5.8.6 Eliciting and Documenting Adverse Events,<br>Section 4.3.1.2 Primary Immunogenicity Endpoint, Section 4.3.2 Secondary Endpoints,<br>Section 4.7 Interim Analyses, Section 6.4 Clinical Study Reports, and<br>Section 7.1 Appendix 1: Schedule of Events (Table 7)	Updated Month 7 and Month 13 visits to Day 209 and Day 394, respectively, to allow for 6-month and 12-month intervals, respectively, after the second injection on Day 29 (Month 1).	Request from the Health Authority.
Synopsis, Section 3.1 Study Design, and<br>Section 7.1 Appendix 1: Schedule of Events (Table 7)	Updated the biweekly safety telephone calls from Day 211 through Day 351 to Day 223 through Day 377.	Consequent to the change made to the Day 209 Visit (Request from the Health Authority).

Section # and Name	Description of Change	Brief Rationale
Synopsis, Section 3.1 Study Design,<br>Section 3.1.2 Rationale for Study Design,<br>Section 3.5.1 Assessment for SARS-CoV-2 Infection, and<br>Section 7.1 Appendix 1: Schedule of Events (Table 7)	Updated nasal swab to nasopharyngeal swab.	Clarified the type of swab to be performed.
Section 3.1.1 Rationale for Dose Selection	Updated enrollment and preliminary safety data from the ongoing Phase 1 DMID study.	Updated based on the preliminary findings of the Phase 1 DMID study.
Section 3.2.1 Inclusion Criteria	Updated inclusion criterion #7 to exclude sperm donations through 3 months after the last injection.	Update to align with the informed consent form on refraining male participants from sperm donation through 3 months after the last injection based on IRB feedback to the ICF.
Section 3.3.2 Handling Withdrawal From the Study	Updated the scheduled end of study assessments at Day 394 (Month 13) to allow for a 12-month interval after the second vaccination on Day 29 (Month 1).	Request from the Health Authority.
Section 3.4.5 Blinding	Updated the method to maintain the blind of the dosing assignment from opaque sleeve to blinding label.	Operational change in cases for which opaque sleeves are not used.
Section 3.5.1 Assessment for SARS-CoV-2 Infection	• Added more intense monitoring of participants who are confirmed to have SARS-CoV-2 infection (ie, notification of the participant's primary care physician by the investigator and recording of confirmed SARS-CoV-2 infection as an MAAE along with relevant concomitant medications and details about severity, seriousness, and outcome).<br>• Added a convalescent visit with blood collection after diagnosis of SARS-CoV-2 infection.	Request from the Health Authority.
Section 3.5.1 Assessment for SARS-CoV-2 Infection and Section 3.5.8.2 Medically Attended Adverse Event	Deleted "or COVID-19."	Editorial update for internal consistency.
Section 4.3.3 Exploratory Endpoints	Included a new exploratory endpoint to evaluate the effect of the mRNA-1273 vaccine on the incidence of SARS-CoV-2 infection.	Request from the Health Authority.

Section # and Name	Description of Change	Brief Rationale
Section 7.1 Appendix 1: Schedule of Events (Table 7)	Deleted that Day 0 and Day 1 visits may be combined the same day.	Editorial update of template text, which did not apply to this protocol.
	Corrected sequential footnote numbering in the schedule of events (Table 7).	Editorial update.
	Included a header row titled "Days Since Most Recent Vaccination."	Update to clarify that the visits are relative to the most recent injection.

Abbreviation: DMID = Division of Microbiology and Infectious Diseases; ICF = informed consent form; IRB = Institutional Review Board; MAAE = medically attended adverse event; SARS-Cov-2 = Severe Acute Respiratory Syndrome coronavirus that causes COVID-19.

8 REFERENCE LIST

Callaway E, Mallapaty S. (2021). Novavax offers first evidence that COVID vaccines protect people against variants. Nature. 2021Feb;590(7844):17.

Centers for Disease Control and Prevention (CDC). 2020a. Coronavirus Disease 2019 (COVID-19). Retrieved from: https://www.cdc.gov/coronavirus/2019-ncov/cases-updates/cases-in-us.html. (accessed 2020 Apr 20).

Centers for Disease Control and Prevention (CDC). 2020b. Coronavirus Disease 2019 (COVID-19). Retrieved from: https://www.cdc.gov/coronavirus/2019-ncov/cases-updates/summary.html. (accessed 2020 Apr 20).

Centers for Disease Control and Prevention (CDC). 2020c. Coronavirus Disease 2019 (COVID-19). Retrieved from: https://www.cdc.gov/coronavirus/2019-ncov/symptoms-testing/symptoms.html. (accessed 2020 Apr 14).

Chen Y, Lu S, Jia H, Deng Y, Zhou J, Huang B, et al. A novel neutralizing monoclonal antibody targeting the N-terminal domain of the MERS-CoV spike protein. Emerg Microbes Infect. 2017 May 24;6(5):e37.

Cohen, J. One-dose COVID-19 vaccine offers solid protection against severe disease. Science. 2021 Jan 29 Jan 2021. doi:10.1126/science.abg7115.

Corti D, Zhao J, Pedotti M, Simonelli L, Agnihothram S, Fett C, et al. Prophylactic and postexposure efficacy of a potent human monoclonal antibody against MERS coronavirus. Proc Natl Acad Sci U S A. 2015 Aug 18;112(33):10473-8.

Department of Health and Human Services (DHHS), Food and Drug Administration, Center for Biologics Evaluation and Research (US). Guidance Document. FDA guidance on conduct of clinical trials of medical products during COVID-19 public health emergency. March 2020. Updated on April 16, 2020. [cited 2020 Apr 17]. Available from: https://www.fda.gov/media/136238/download.

Department of Health and Human Services (DHHS), Food and Drug Administration, Center for Biologics Evaluation and Research (US). Guidance for industry: Toxicity grading scale for healthy adult and adolescent volunteers enrolled in preventive vaccine clinical trials. September 2007 [cited 2019 Apr 10] [10 screens]. Available from: https://www.fda.gov/downloads/BiologicsBloodVaccines/GuidanceComplianceRegulatoryInformation/Guidances/Vaccines/ucm091977.pdf.

Department of Health and Human Services (DHHS), National Institutes of Health, National Institute of Allergy and Infectious Diseases, Division of AIDS. Division of AIDS (DAIDS)

table for grading the severity of adult and pediatric adverse events. Version 2.0. November 2014. [cited 2019 April 13]. Available from: https://rsc.niaid.nih.gov/sites/default/files/daids-ae-grading-table-v2-nov2014-highlighted-changes.pdf.

Johnson RF, Bagci U, Keith L, Tang X, Mollura DJ, Zeitlin L, et al. 3B11-N, a monoclonal antibody against MERS-CoV, reduces lung pathology in rhesus monkeys following intratracheal inoculation of MERS-CoV Jordan-n3/2012. Virology. 2016 Mar;490:49-58.

Kim Y, Lee H, Park K, Park S, Lim JH, So MK, et al. Selection and characterization of monoclonal antibodies targeting middle east respiratory syndrome coronavirus through a human synthetic fab phage display library panning. Antibodies (Basel). 2019 Jul 31;8(3).

Pallesen J, Wang N, Corbett KS, Wrapp D, Kirchdoerfer RN, Turner HL, et al. Immunogenicity and structures of a rationally designed prefusion MERS-CoV spike antigen. Proc Natl Acad Sci USA. 2017;114(35):E7348-E7357.

Ruggeberg J, Gold M, Bayas J, Blum M, Bonhoeffer J, Friedlander S, et al. Anaphylaxis: case definition and guidelines for data collection, analysis, and presentation of immunization safety data. Vaccine. 2007 Aug 1; 25(31):5675-5684.

Tegally H, Wilkinson E, Giovanetti M, Iranzadeh A, Fonseca V, Giandhari J, et al. Emergence and rapid spread of a new severe acute respiratory syndrome-related coronavirus 2 (SARS-CoV-2) lineage with multiple spike mutations in South Africa. medRxiv. 2020.12.21.20248640.

Wang L, Shi W, Joyce MG, Modjarrad K, Zhang Y, Leung K, et al. Evaluation of candidate vaccine approaches for MERS-CoV. Nat Commun. 2015 Jul 28;6:7712.

Wang L, Shi W, Chappell JD, Joyce MG, Zhang Y, Kanekiyo M, et al. Importance of neutralizing monoclonal antibodies targeting multiple antigenic sites on the middle east respiratory syndrome coronavirus spike glycoprotein to avoid neutralization escape. J Virol. 2018 Apr 27;92(10).

Wang Z, Schmidt F, Weisblum Y, Muecksch F, Barnes CO, Finkin S, et al. mRNA vaccine-elicited antibodies to SARS-CoV-2 and circulating variants. bioRxiv 2021.01.15.426911.

Widge AT, Rouphael NG, Jackson LA, Anderson EJ, Roberts PC, Chappell JD, et al. Durability of Responses after SARS-CoV-2 mRNA-1273 Vaccination. N Engl J Med. 2021;384:80-2. doi: 10.1056/NEJMc2032195.

Widjaja I, Wang C, van Haperen R, Gutiérrez-Álvarez J, van Dieren B, Okba NMA, et al. Towards a solution to MERS: protective human monoclonal antibodies targeting different

domains and functions of the MERS-coronavirus spike glycoprotein. Emerg Microbes Infect. 2019;8(1):516-30.

World Health Organization (WHO). (Data reported as of 2020, Apr 20). Coronavirus disease 2019 (COVID-19) Situation Report – 34. Retrieved from: https://www.who.int/docs/default-source/coronaviruse/situation-reports/20200420-sitrep-91-covid-19.pdf?sfvrsn=fcf0670b_4. (accessed 2020 Apr 20).

Wrapp D, Wang N, Corbett KS, Goldsmith JA, Hseih CL, Abiona O, et al. Cryo-EM structure of the 2019-nCoV spike in the prefusion conformation. Science. 2020;367:1260-3.

Wu K, Werner AP, Moliva JI, Koch M, Choi A, Stewart-Jones GBE, et. al. mRNA-1273 vaccine induces neutralizing antibodies against spike mutants from global SARS-CoV-2 variants. bioRxiv [Preprint]. 2021

Yu X, Zhang S, Jiang L, Cui Y, Li D, Wang D, et al. Structural basis for the neutralization of MERS-CoV by a human monoclonal antibody MERS-27. Sci Rep. 2015 Aug 18;5:13133.

Signature Page for VV-CLIN-002041 v2.0

Approval	Roderick McPhee<br>Medical<br>04-May-2021 12:56:09 GMT+0000

Approval	Deborah Manzo<br>Clinical<br>04-May-2021 13:56:17 GMT+0000

Approval	Jacqueline Miller<br>Clinical<br>04-May-2021 14:40:46 GMT+0000

Approval	David Martin<br>Pharmacovigilance<br>05-May-2021 16:27:26 GMT+0000

Approval	Charbel Haber<br>Regulatory<br>05-May-2021 18:42:51 GMT+0000

Signature Page for VV-CLIN-002041 v2.0
